# Association between expression of random gene sets and survival is evident in multiple cancer types and may be explained by sub-classification

**DOI:** 10.1371/journal.pcbi.1006026

**Published:** 2018-02-22

**Authors:** Yishai Shimoni

**Affiliations:** IBM Research-Haifa, Haifa, Israel; Baylor College of Medicine, UNITED STATES

## Abstract

One of the goals of cancer research is to identify a set of genes that cause or control disease progression. However, although multiple such gene sets were published, these are usually in very poor agreement with each other, and very few of the genes proved to be functional therapeutic targets. Furthermore, recent findings from a breast cancer gene-expression cohort showed that sets of genes selected randomly can be used to predict survival with a much higher probability than expected. These results imply that many of the genes identified in breast cancer gene expression analysis may not be causal of cancer progression, even though they can still be highly predictive of prognosis. We performed a similar analysis on all the cancer types available in the cancer genome atlas (TCGA), namely, estimating the predictive power of random gene sets for survival. Our work shows that most cancer types exhibit the property that random selections of genes are more predictive of survival than expected. In contrast to previous work, this property is not removed by using a proliferation signature, which implies that proliferation may not always be the confounder that drives this property. We suggest one possible solution in the form of data-driven sub-classification to reduce this property significantly. Our results suggest that the predictive power of random gene sets may be used to identify the existence of sub-classes in the data, and thus may allow better understanding of patient stratification. Furthermore, by reducing the observed bias this may allow more direct identification of biologically relevant, and potentially causal, genes.

## Author summary

Multiple gene sets have been published as predictive of cancer progression and metastasis in several cancer types. Although many of these sets proved to be highly predictive of survival, even gene sets for the same cancer (but from different data-sets or different analyses) exhibit very little overlap and to date did not provide functional therapeutic targets. Recent studies found that in breast cancer, even random gene sets can predict survival much better than would be expected, and on average are better than many published gene sets. Together, these results undermine the causal role of the published gene sets and their potential clinical implications. We show that random gene sets predict survival in many cancer types, and that this property no longer exists after splitting the data into subclasses based on data-driven clusters. This suggests that such sub-classification could increase the likelihood to identify causal genes that are potential therapeutic targets, and that this property can be used as an indication that there may be subclasses within the dataset.

## Introduction

The last two decades have seen a proliferation of papers, each proposing a set of genes that are important for cancer progression, metastasis, or patient stratification [1–7], where most of the findings stem from the computational analysis of gene-expression data from large patient cohorts. While such gene sets have been shown to have good predictive power [8], gene sets obtained by similar analyses of the same cancer type, but using different cohorts, provided poor overlap in the identity of the genes, and in some cases these sets proved to be non-robust [9]. Furthermore, even within a single dataset it has been shown that multiple gene sets can be obtained with equal predictive power [10, 11]. This reduces the significance of the identity of individual genes in the gene set, as they can be replaced by many others with little to no loss in predictive power. From a clinical point of view, most genes obtained from such sets did not prove to be useful therapeutic targets. This may not be surprising given the results presented above, but highlights the importance of identifying genes that are not only predictive but are also causal of disease progression.

Many methods have been developed to address the problems mentioned above, including generating meta-analyses that combine multiple data sets [12]; adding information about interactions between genes [9, 13–15]; checking the robustness of the signature when choosing small sub-cohorts [9]; and adding biologically-relevant expert knowledge [11]. Results obtained from such analyses are more likely to identify causal genes, and some of the predicted genes were even validated in *in vitro* settings [15], but the clinical implications of such analyses remain to be seen.

Recently it was shown by Venet *et al*. [16] that in breast cancer random gene sets can predict survival much better than can be expected by chance, meaning that when using the expression of random genes to split the cohort into two groups the groups have significantly different survival curves. Let us consider the property that random sets of genes can be used to predict survival. Intuitively, we might think that by using random gene sets to split the cohort we should end up with random assignments into groups. However, if that was the case then such a procedure would provide p-values that are uniformly distributed between 0 and 1, and thus, 5% of the p-values should fall below 0.05. In contrast, Venet *et al*. observed that most of the assignments into groups induced by random genes provided a statistically significant separation in survival curves (or a p-value smaller than 0.05). This means that the separation into two groups that is induced by random gene sets is in fact not random, and moreover—that this separation into two groups is related to survival. From here on, when a larger (or smaller) than expected proportion of random gene sets predict some clinical property (e.g., survival) in a statistically significant way, we say that the data exhibits *random bias*.

The discovery of random bias in breast cancer gene expression cohorts further questions the causal relevance of individual genes that are identified in signatures based solely on these gene expression cohorts. Venet *et al*. attributed the phenomenon of random bias to a large proliferation signature that affects a substantial proportion of the genes in the genome. They suggest that most random gene sets are statistically likely to include some genes from this proliferation signature and are thus predictive of proliferations, and by proxy of survival, as well. Therefore, when choosing a random gene set and using it to split the data into two groups these groups are not random, but are separated by the activity of that proliferation signature. They showed that they could remove the random bias by defining a proliferation score (see the Methods section) and then removing the effect of that score from all the genes in the expression data.

The hypothesis that random bias stems from a large proliferation signature is an attractive one. It stands to reason, however, that if it holds true for breast cancer it should also hold true for many, if not all, cancer types. We used data from the cancer genome atlas (TCGA) project to determine the prevalence of random bias in all available cancer types, and whether the removal of a proliferation signature can remove this bias. By removing such random bias, it may be possible to restore some of the causal interpretation for gene signatures discovered by computational analysis.

We present the results of this analysis for 34 datasets downloaded from TCGA and show that 24 out the datasets exhibit significant random bias. We further show that for most of these cases the random bias cannot be removed by using the proliferation score as described in Venet *et al*. We demonstrate how sub-classification by unsupervised clustering (but not sub-classification by grade) can help reduce random bias for cases in which the proliferation score was insufficient to remove random bias. We conclude by discussing the implications of our results on further research into genes that are causal for disease progression.

## Materials and methods

### TCGA data

We downloaded TCGA data from the TCGA data matrix on November 30, 2015 using the TCGA2STAT R package [17]; This gave us a total of 34 RNAseq expression datasets with their adjoining survival and clinical information. We used the level 3 data normalized using RSEM. We used the dataset abbreviations as defined by the TCGA consortium (as defined in https://tcga-data.nci.nih.gov/docs/publications/tcga/). Two of the datasets comprised an agglomeration of two cancer types, specifically, GBMLGG (glioblastoma multiforme and brain low grade glioma) and COADREAD (colon adenocarcinoma and rectum adenocarcinoma), where each of the individual datasets also appears in the data.

### Defining random bias

To define random bias, we follow the methods presented by Venet *et al*. [16] as follows. Given a normalized dataset with *N* samples holding gene expression data for *M* genes, we chose gene sets of an arbitrary size *s* with uniform probability and without substitution. Next, we performed singular value decomposition (also known as principal component analysis) on the new gene-expression sub-matrix to obtain the sample weights for the first principle component. The samples were then split into two groups, depending on whether the weight assigned to each sample was above or below the median value. Finally, we evaluated the separation in survival curves between the two groups using a log-rank test to obtain a p-value. The process was repeated *b* = 5000 times to obtain a set P of p-values.

### The proportion of significant random sets

Given sufficient data, a random assignment of samples into two groups should provide a null distribution of p-values that is uniform between zero and one. This is different from the assignment described above since it does not use the data to choose the random assignment, and is therefore truly random and independent from any clinical outcome. However, since some of the survival data was sparse or had insufficient follow-up time (such as for PRAD, prostate adenocarcinoma), even such a random assignment would have some arbitrary non-uniform distribution of p-values, which served as our null distribution. By performing random assignments of samples into two groups to obtain the p-value for the induced separation in survival curves, and repeating the procedure *b* times, we obtained a set R of p-values that are drawn from this null distribution.

From the null distribution, we obtained the 5th percentile, *r*, and calculated the proportion of p-values that are smaller than *r* in P. We call this value the *proportion of significant random sets of size s*. If no bias exists, this value should be close to 0.05. If the value is significantly larger, we determined the dataset to have a *positive random bias*. If the value is lower than 0.05, we said that the dataset has *negative random bias*.

### Consistency of the predictive power of random gene sets

To check that the predictive power of random gene sets is consistent we divided the data randomly into two groups, where each sample has a 50% chance of being chosen to each group. We then randomly chose 100 random sets and calculated the p-values in both halves of the data for each of the random sets. Since individual divisions into halves may provide biased results we repeat this procedure 50 times. The resulting vectors are then compared by Fisher exact test to estimate the statistical significance of obtaining random sets that are significant in both halves of the data. Additionally, the vectors are used to evaluate the proportion of random sets that are significant in both halves of the data.

### Statistical significance of the proportion of significant random sets

We can determine the p-value of the proportion of significant sets by applying the central limit theorem to the difference in proportions obtained in R and in P, providing the statistic

$$\begin{array}{c} Z=\frac{r-0.05}{\sqrt{\frac{r(1-r)}{N}+\frac{0.05(1-0.05)}{N}}}. \end{array}$$ (1)

Under the null hypothesis, *Z* should be drawn from the normal distribution N(0,1). Thus, we obtain the two-sided significance of the proportion *r*. If the significance was less than 0.05, then the dataset was deemed to exhibit significant random bias.

### Adjusting for the PCNA signature

Our procedure for obtaining and adjusting for the proliferation score follows the method of Venet *et al*. [16]. They determined a proliferation signature by identifying the 1% of genes whose expression is most correlated with the gene PCNA across many datasets downloaded from the Gene Expression Omnibus. For each sample, we determined the expression of these proliferation genes and the median value across these genes to provide a proliferation score for that sample. To remove the proliferation effect, we performed a linear fit between each gene in the dataset and the proliferation score across all samples. We replaced the expression of each gene by the residual from the linear fit.

### Data clustering

To perform clustering of the high dimensional gene-expression data using the least number of parameters, we chose to use phenoGraph [18]. The algorithm follows several steps. First, using the similarity between all samples it identifies the *k*-nearest neighbors of each sample. We used Spearman’s correlation as the distance metric. The algorithm then computes the statistical significance of the overlap in neighbors between each pair of samples. This is done using a Fisher exact test to define a weight for the interaction between each pair of samples, thus creating a weighted network. Finally, phenoGraph applies the Louvain algorithm on the network to find a partitioning into clusters that maximize the modularity of the network [19]. The Louvain algorithm has been successfully used in the context of social networks, and is considered by many to be the state-of-the art [20]. The algorithm is remarkably insensitive to the value of *k*, has no additional parameters, and the number of resulting clusters is data-driven and not given as a parameter. This makes it suitable for our purpose because we do not focus on the specific clusters that were obtained, rather on their general properties.

An implementation of phenoGraph is available in Python by the authors [18]. To perform the analysis, we created an R package implementing phenoGraph, based on the code description. The package is available at https://github.com/yishaishimoni/phenoClust.git.

## Results

We analyzed 34 datasets downloaded from TCGA for the existence of random bias, as described in the Methods section. For each dataset we chose *b* = 5000 random gene sets of sizes *N* = 2^0‥10^. Then, using the first principal component of each random set we split the data into two equal-sized groups and obtained the p-value of the separation in survival curves between the two groups. The results of this analysis are summarized in Table 1, where for each set of 5000 p-values we obtained the following: a) The proportion of significant sets in percentage values (Signif %) b) The significance of the proportion using a proportion test compared to randomly splitting the dataset into two groups (P-value) and c) The proportion of significant sets after adjusting for the PCNA signature in percentage values (PCNA %)

**Table 1:** Analysis of random bias in TCGA datasets.

Dataset	Signif %	P-value	PCNA %	PCNA p-val
ACC	80	0	45	2.7e-122
BLCA	55	2e-187	51	1e-157
BRCA	21	1.3e-27	14	1.1e-12
CESC	6	0.38	7	0.072
CHOL	1	6.7e-10	1	2.2e-09
COAD	3	0.0063	1	5.7e-07
COADREAD	5	0.64	2	0.00012
DLBC	5	0.8	3	0.033
ESCA	0	1.1e-12	0	8.9e-12
GBM	7	0.075	7	0.11
GBMLGG	99	0	88	0
HNSC	25	8.5e-38	28	4.9e-48
KICH	8	0.0095	6	0.4
KIPAN	64	1.9e-279	24	1.3e-35
KIRC	82	0	68	0
KIRP	63	3.4e-260	10	4.1e-05
LAML	0	4.9e-11	0	1.1e-10
LGG	80	0	66	5.9e-302
LIHC	34	5.2e-70	4	0.35
LUAD	45	8.8e-118	19	3.8e-22
LUSC	20	1.9e-25	12	5.5e-09
MESO	53	7.7e-172	20	1.1e-25
OV	4	0.12	4	0.3
PAAD	43	2.1e-107	6	0.37
PCPG	4	0.16	4	0.17
PRAD	1	4.3e-06	1	3.9e-08
READ	2	0.00012	2	0.00061
SKCM	3	0.016	4	0.54
TGCT	0	2e-12	0	4e-13
THCA	3	0.0049	4	0.23
THYM	18	6.2e-21	20	2.7e-25
UCEC	62	1.1e-250	46	2.8e-127
UCS	2	1.7e-05	1	7.9e-06
UVM	38	5.3e-84	21	4e-28

In this table ‘Dataset’ indicates the abbreviation of the dataset as defined by the TCGA consortium; ‘Signif %’ is the proportion of significant random sets of size 64; ‘P-value’ is the significance by a proportion test for obtaining the proportion of significant %; ‘PCNA %’ is the proportion of significant random sets of size 64 after adjusting for the PCNA signature; and ‘PCNA p-val’ is the significance of the value in PCNA %. The analysis was performed with 5000 random sets.

Table 1 exhibits the results of this analysis for each of the datasets using random sets of *N* = 64 genes; 17 out of the 34 datasets exhibit significant positive random bias, while 10 datasets do not exhibit significant random bias. This is somewhat surprising considering the interpretation presented in [16] that random bias stems from a proliferation signature, since such an effect should hold true for most cancer types.

Importantly, seven of the datasets exhibited significant negative random bias, or a significant lack of small p-values. Such a property can only occur when the group assignment induced by random genes consistently provides a separation into groups that do not differ in their survival curves. Note that this cannot be explained by separation into random groups, since the p-values in the null distribution are drawn from just such a random assignment into groups, and so the proportion of a random assignment should be similar to that of the null distribution and not less. It also cannot be explained as separation into groups that are statistically unrelated to survival, since this would provide a uniform distribution of p-values, which is not what is observed in the data (as seen by the cumulative distribution plots in S1 File). One conceivable way to obtain such a consistent separation into groups would be by the existence of sub-classes in the data, where those sub-classes share a similar survival profile. We will explore this option in a later part of the manuscript.

To investigate whether the random genes that are associated with survival are indeed predictive we repeated this analysis where for each random set we checked the p-value obtained on random halves of the dataset, as explained in the Methods section. For example, when we ran this analysis of the BLCA dataset, for which there are sufficient samples and a large proportion of significant random sets, the proportion of significant sets of size 50 in each half was approximately 0.25, while the proportions of sets that were significant in both sets was approximately 0.1, signifying an odds ratio of about 2. We then checked the significance of the proportion of random sets that are predictive in both halves of the data using a Fisher exact test, which provided a p-value of 4.6*e* − 25. The results for other datasets with sufficient sample size and significant random bias are similar, and are summarized in Table 2. This result indicates that these random gene sets are not only predictive of survival in a significant way, but also in a consistent way.

**Table 2:** Consistency of random gene sets.

Dataset	Samples	Signif %	Repeat %	P-value	OR
ACC	79	52	32	5.7e-24	1.8
BLCA	408	28	12	9.9e-40	2.4
BRCA	1093	13	3	3e-3	1.4
GBMLGG	667	99	98	0.5	1.3
HNSC	520	13	3	1e-4	1.5
KIPAN	888	44	21	3.1e-5	1.3
KIRC	533	71	52	0.18	1.1
KIRP	289	32	13	2.0e-20	1.8
LGG	515	66	47	5.4e-5	1.3
LIHC	362	19	6	1.0e-14	1.9
LUAD	514	25	8	2.6e-7	1.4
LUSC	499	12	3	1.8e-12	2.3
MESO	86	33	12	4.5e-10	1.5
PAAD	178	21	7	1.1e-15	1.9
THYM	120	9	2	1.5e-10	2.5
UCEC	369	39	17	1.3e-6	1.3
UVM	80	25	8	3.7e-17	1.8

In this table ‘Dataset’ indicates the abbreviation of the dataset as defined by the TCGA consortium; ‘Samples’ is the number of samples in the dataset; ‘Signif %’ is the average proportion of significant random sets of size 64 in each half of the original dataset; ‘Repeat %’ is the average proportion of random sets that are significantly correlated to survival in both halves of the dataset; ‘P-value’ is the significance by a Fisher exact test for obtaining the proportion of repeat %; ‘OR’ is the odds ratio for obtaining a random set that is significant in both halves compared to random. The analysis was performed for each dataset with a total of 5000 random sets consisting of 50 choices of halves and 100 random set for each such division.

**Fig 1:**
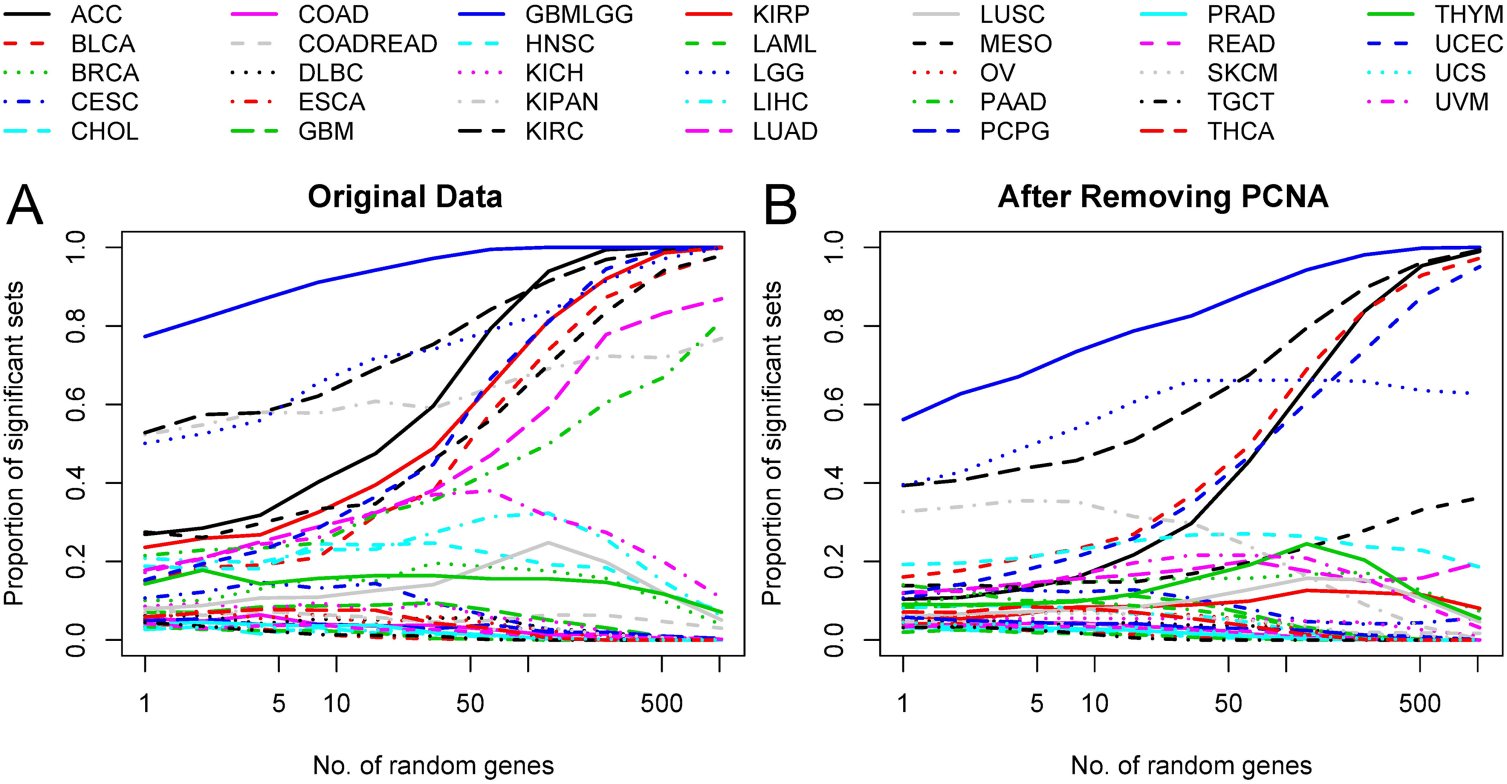
Random bias vs. random set size. The *x* axis represents the size of the random gene sets that were chosen, and the *y* axis represents the proportion of significant sets of that size. A. The results of the analysis using the raw data, and B. The results of the analysis after adjusting for the PCNA meta-gene.

The connection between random bias and the number of genes in the gene set is displayed in Fig 1A, where the *x* axis represents the number of genes in each random gene set and the *y* axis represents the proportion of significant sets of that size. For small set sizes, the results span the range of possible significance ratios (i.e., between zero and one), while large sets tend towards either one, 0.05, or zero. The fact that many of the cohorts exhibit a random bias for even a handful of genes, as seen on the left-most side of the plot, strongly supports the assumption that there is an important biological signature affecting a large proportion of the genes, and that this is the cause for the random bias. The question remains, however, whether or not this biological signature can be associated with proliferation.

To check whether proliferation can account for random bias, we repeated the above analysis after adjusting for the PCNA proliferation signature as described in the Methods section and as suggested by Venet *et al*. [16]. The results of this analysis are presented in the two rightmost columns in Table 1 (PCNA %, and PCNA p-val), as well as in Fig 1B. This analysis shows that the adjustment does decrease the proportion of significant sets for most datasets. However, only two datasets that exhibited significant positive bias lost the statistical significance. For all the other datasets exhibiting random bias, the adjustment to the PCNA signature was insufficient to remove the random bias.

Considering these findings, an explanation other than proliferation is required to account for random bias. We propose that in some cases, random bias may be due to other large-scale biological programs within the dataset. In general, such biological programs can span any biological process that is important for cancer progression or initiation, such as any of the hallmarks of cancer [21], grade, stage, etc. However, these hypotheses cannot explain the phenomena presented earlier, most notably the fact that some datasets exhibit negative random bias, or a lack of small p-values. As explained above, for this to happen the random gene sets must be able to capture a strong and consistent biological difference between subsets, but one that is not associated with survival. Such a difference is unlikely, therefore, to be one of the cancer hallmarks, as those are likely to be highly associated with survival.

Another possibility that may be more general is the biological signature of sub-classification (e.g., indicating susceptibility to some therapy [11]). Such a signature would satisfy both the requirements for consistency and large biological differences, but would not necessarily be associated with survival. To explain this, consider a case where two cancer types arise in the same organ but from two tissues of origin. In general, these two cancer types will differ in their survival profiles. In such a case, a significant difference in gene expression would be observed between the two groups, and a large proportion of the genome will be involved in this difference. When gene-sets are chosen at random, the proportion of differentially expressed genes will be the same in the random set as in the whole genome, and these genes will cause the random gene-set to be predictive of the subclasses, and only by proxy of survival as well. Therefore, having subclasses in the same data-set may easily lead to random bias.

The hypothesis that random bias stems from sub-classification is also supported by the fact that the largest random bias was observed in the agglomerated dataset holding samples from both glioblastoma multiforme (GBM) and from brain low grade glioma (LGG), which are known to have vastly different prognoses. Individually, LGG and GBM exhibit significant ratios of random gene sets of size *N* = 64, with values of 0.84 and 0.14, respectively. In the combined dataset, the significant ratio is almost 1.

It should be noted, however, that the dataset containing samples from both colon adenocarcinoma and rectum adenocarcinoma does not show the same effect; neither the individual data sets nor the combined data set exhibit random bias. This may be caused by the fact that both cancer types share very similar survival dynamics and in fact are often referred to together as colorectal adenocarcinoma [22], and therefore differences in survival dynamics cannot be detected by this method of identifying random bias. This result suggests that, in some cases, merging data sets may not cause random bias.

To test the hypothesis that subclasses in the data can explain random bias we chose to use a data-driven unsupervised clustering algorithm called phenoClust [18], as explained in the Methods section. This algorithm is based on network modularity, which is widely used in social networks, and identifies “neighborhoods” of samples that share nearest neighbors, as determined by similarity. It should be noted that the choice of clustering algorithm is not the focus of this analysis, and that we chose phenoClust as a convenient example. However, to ascertain that the clustering provides biologically relevant results, we compared the clusters provided by phenoClust for the breast cancer dataset (BRCA) to the sub-classification obtained by the PAM50 classification method [23], which has become the state-of-the-art method for computational classification of breast cancer samples using gene-expression data [24]. The clusters that were obtained by phenoClust are in high agreement with the PAM50 classification, as shown in S1 Fig. Specifically, each phenoClust cluster consists mostly of a single PAM50 subclass.

**Fig 2:**
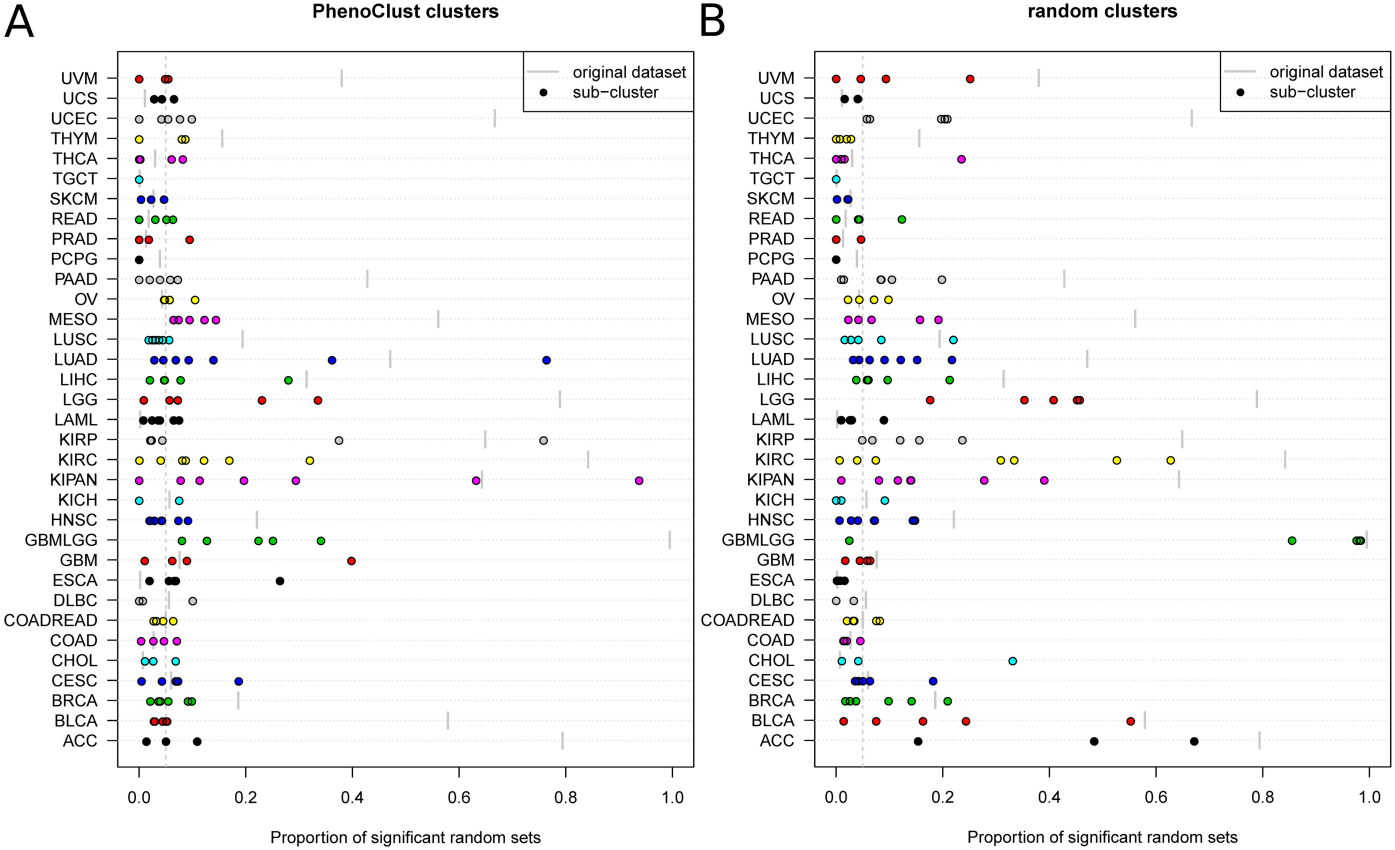
The effect of clustering on random bias. Each horizontal line represents a single TCGA dataset, where the location of each dot along the *x* axis represents the proportion of significant sets in a single cluster and the location of the short vertical gray line indicates the proportion of significant sets in the complete dataset. Both proportions were calculated using random sets of size *N* = 64. A. The analysis using the phenoClust clusters B. The analysis using random sub-sampling of the datasets with groups of samples of the same size as the clusters determined by phenoClust.

We next checked the effect of clustering on random bias, by repeating the random bias analysis (as described above) for each cluster separately, and then comparing the proportion of significant sets to the proportion in its original dataset. The results show that in almost all cases, the proportion of significant sets was closer to the expected value of 0.05, as shown in Fig 2A. This is true both for datasets that exhibited positive random bias and for datasets that exhibited negative random bias. Specifically, out of 106 clusters that originated from datasets exhibiting significant random bias, 65 lost their bias.

It should be noted that the proportions of significant random sets in the BRCA sub-classes by phenoClust (0.021, 0.036, 0.04, 0.055, 0.091, and 0.099), were very similar to the proportions in the PAM50 classes (LumA: 0.036, LumB: 0.074, Basal: 0.038, Her2: 0.05, and Normal: 0.046). This indicates that, as expected, biologically driven sub-classification achieves results that are similar to or better than the data-driven approach.

We performed similar analyses on random sub-samples of the datasets of sizes identical to those of the cluster obtained by phenoClust. We observed some reduction in random bias (as expected due to the reduction in statistical power), but the effect was less pronounced than when using the phenoClust clusters, as shown in Fig 2B. As a comparison, out of the 106 clusters that originated from datasets exhibiting significant random bias, only 32 lost their bias. We performed the above analysis without removing the PCNA proliferation signature, since the effect of the adjustment had a minor impact on the results, as shown in S2 Fig. This result shows that random bias may be used to detect the existence of sub-classes in a dataset.

To examine the possibility that clustering by clinical features may provide a similar explanation to random bias we evaluated the effect of grouping samples according to cancer stage for all data-sets for which this clinical information exists. The results of this analysis are shown in S3 Fig, and show that in most cancer type exhibiting large proportions of significant sets the grade does not allow removal of random bias.

## Discussion

The results presented above suggest that in many cancer types random gene sets are predictive of survival, and that this predictive power is real and robust, and is not a result of some property of the dataset or of the analysis. The proposition that this is due to a large proliferation signature that affects many genes is an attractive one. While this may be true in some cases, the fact that not all cancer types exhibit this property casts a pall over it, and suggests the need for a larger, more over-arching explanation. The proliferation signature suggested by Venet *et al*. did not provide the effect of removing the random bias that was reported in the original paper. It is possible that the signature that was provided was platform-specific or that the effect was database-specific, but we can only speculate. To avoid such potential problems, we attempted to compare our results as much as possible to random ones, rather than focusing on some biological signature. This allowed us to show in a robust way that we could remove the bias more than could be done using random assignments. This does not preclude the option that in some cases a biologically relevant signature may provide the best explanation for random bias.

The method we used here is not the only method to identify random bias. One can create any conceivable method to extract a score for each sample in the data from these random sets, and use it to split the samples into sub-groups. If a statistically significant result emerges, this is still random bias. Similarly, the test can be based on any desired property of the data that needs to be predicted, not necessarily survival. Furthermore, the method employed here is biased towards a dataset that is composed of two equal-sized sub-groups with different survival dynamics. If one of the groups is large compared to the other, or if the difference in survival is small, then splitting the data into two equal-sized groups may make it difficult to detect a meaningful difference in the survival curves.

Our approach is somewhat weakened by the fact that splitting the data into random sub-classes also decrease the proportion of significant sets. Although the data-driven clustering provides a much more significant reduction, splitting the data reduces the statistical power of any method. It remains to be seen whether this reduction in statistical power will disallow identification of the predictive and causal genes within each subclass. The fact that in some of the subclasses the proportion of significant sets increased to significant levels (a phenomenon that was not observed in random subclasses) indicates that these subclasses harbor additional information that is highly predictive of survival; therefore clearly, at least some of the subclasses still harbor relevant information. It is possible (or even likely) that these cases are the ones in which a biologically relevant signature will provide a strong association with survival. These signatures may arise from an association with proliferation, grade, stage, or with one of the hallmarks of cancer. Furthermore, it should be pointed out that not all the random bias was removed by sub-classification. This is yet additional indication that further investigation of random bias and its origin is needed.

Related to the above, it should be noted that some subclass or data-set that exhibits no random bias may still consist of additional subclasses. One possible example of this is the basal-like PAM50 classification in the BRCA data-set. This is a class that is highly related to the triple-negative subtype, and it is well established that this is a highly heterogeneous disease and it was suggested that it consists of several subclasses [25–27]. In our data-driven approach this subtype was identified as a single subclass with very little random bias. Finally, as mentioned in the Results section, we chose to use the phenoClust clustering algorithm because it is almost purely data-driven and has very few parameters. It is likely that adding biological information and performing the clustering in a more supervised way will result in more consistent clusters that are not dataset-specific and are platform independent (e.g., pre-defining the number of clusters or sub-selecting the biologically relevant features). As a result, we chose not to focus on the sub-clusters that were obtained or their biological relevance and implications. The complete set of results and the associated R-code are available to anyone who wishes to further explore these clusters at https://github.com/yishaishimoni/Random_genes.

### Conclusion

We have shown here that gene expression data from random gene sets has a predictive power for survival in cancer, and is a property that spans many cancer types. The phenomenon was first reported by Venet *et al*. [16] in breast cancer, in a dataset where expression was measured using microarrays. We demonstrated that the same phenomenon is exhibited by most of the 34 gene-expression datasets appearing in the TCGA data matrix, which were obtained using RNAseq. This implies that this is not an artifact of the platform we used, nor is it specific to the data-set that we used.

This result raises concerns regarding the causal significance of genes obtained from analyses using such datasets, and explains the lack of reproducibility in identifying predictive and causal genes. In general, as shown previously, computational analyses can provide sets of genes that are highly predictive of survival, and can provide valuable insights into the biological mechanisms driving cancer survival, progression, and metastasis. We observe that only a handful of genes are truly causal, in the sense that altering their expression or activity will influence survival; however, the fact that many random subsets may arise with similar predictive power hides their identity. Subsequently, our analysis and the proposed changes in methodology may have far-reaching implications for follow-up research on potential therapeutic targets.

The cautionary conclusion that can be drawn from these results is that each data set must be examined for random bias. Moreover, this bias must be removed prior to performing any analysis aimed at obtaining genes that are causal for cancer progression or survival, and perhaps for disease progression in general. Clearly, like any analysis of real-world evidence done using static data, even after removing the random bias it is still impossible to guarantee that the genes resulting from such an analysis are causal and not just correlated with survival. However, since a truly causal gene will remain correlated with survival in their relevant context, by removing confounding factors and focusing on specific biological contexts we increase the probability that the remaining genes that are survival related may be biologically relevant and causal.

Another result that emerged from our analysis is that in most cases adjusting for a proliferation signature does not remove the predictive power of random gene sets. In fact, even in breast cancer—the very cancer type in which this bias was first observed—the adjustment for proliferation that removed the bias in the NKI dataset [2] no longer removes the bias in the breast cancer dataset provided by TCGA. Rather than giving up or suggesting a dataset-specific gene signature, we suggest that in the cases in which adjusting by the proliferation score (or any other relevant score) is insufficient, sub-classification may be required. We have shown that by performing unsupervised and data-driven clustering, we were able to remove most of the random bias. Since individual subclasses do not exhibit random bias, analysis performed on each of those subclasses is much more likely (but not guaranteed) to produce genes that are not only predictive but also causal.

Since sub-classification of cancer is an active field of research (e.g. [28–30]), this allows for a more positive and pro-active conclusion that the predictive power of random gene sets (or significant lack thereof) indicates the existence of confounding factors in the data, such as sub-classification or some biological signature.

In conclusion, the analysis presented here can lead to a more refined approach for computational methods aimed at identifying causal genes. It can also guide further efforts to define and identify sub-classes in cancer cohorts that have important biological and clinical implications.

## Supporting information

S1 File P-value distributions for all datasets. This pdf file contains a representation of the distributions of p-values obtained from the analysis random gene sets of size *n* = 64 of all data sets. For each dataset the file contains four plots: a) A histogram of the p-values, where the x-axis represents the p-value and the y-axis represents the frequency at which this p-value was observed; b) A quantile-quantile plot in which the x-axis represents the p-values observed in the null distribution and the y-axis represents the p-values observed by the analysis. The red line represents a distribution that is identical to the null distribution, and the gray line corresponds to a p-value of 0.05; c) A quantile-quantile plot similar to b), where the axes are in log-scale; d) The cumulative distribution function of the p-values in the analysis (black) and the null p-values (gray).
(PDF)

S1 Fig Comparison of PAM50 and phenoClust classification of the BRCA dataset. A. The proportion of each phenoClust cluster within the PAM50 sub-classes. The Basal class is composed almost solely of class 6, the HER2 subclass is mostly composed of class 2, the Luminal A subclass mostly consists of classes 3 and 4, and the Luminal B subclass is mostly composed of class 1. B. The reciprocal view of the proportion of PAM50 subclasses within each phenoClust cluster. Each of the phenoClust clusters consists mostly of a single PAM50 subclass.
(TIF)

S2 Fig The effect of adjusting for the PCNA proliferation signature in the phenoClust clusters. The *x*-axis represents the proportion of significant genes in each phenoClust cluster without adjusting for the PCNA signature, and the *y*-axis represents the proportion of significant genes in the same phenoClust clusters after adjusting for the PCNA signature.
(TIF)

S3 Fig The effect of clustering by grade on random bias. Each horizontal line represents a single TCGA dataset, where the location of each dot along the *x* axis represents the proportion of significant sets in a single cluster consisting of samples labeled with the same grade, and the location of the short vertical gray line indicates the proportion of significant sets in the complete dataset. Both proportions were calculated using random sets of size *N* = 64. Classes with less than 10 samples were not analyzed. The analysis was only performed for cancer types for which grade information is available in the clinical information provided by TCGA.
(TIF)
