# Supplementary material for: Association between expression of random gene sets and survival is evident in multiple cancer types and may be explained by sub-classification
Source: PLoS Comput Biol. 2018 Feb 22;14(2):e1006026. doi: 10.1371/journal.pcbi.1006026 (PMC5839591; doi:10.1371/journal.pcbi.1006026)
Supplement: S1 File — This pdf file contains a representation of the distributions of p-values obtained from the analysis random gene sets of size n = 64 of all data sets. For each dataset the file contains four plots: a) A histogram of the p-values, where the x-axis represents the p-value and the y-axis represents the frequency at which this p-value was observed; b) A quantile-quantile plot in which the x-axis represents the p-values observed in the null distribution and the y-axis represents the p-values observed by the analysis. The red line represents a distribution that is identical to the null distribution, and the gray line corresponds to a p-value of 0.05; c) A quantile-quantile plot similar to b), where the axes are in log-scale; d) The cumulative distribution function of the p-values in the analysis (black) and the null p-values (gray). (PDF) [file pcbi.1006026.s001.pdf]

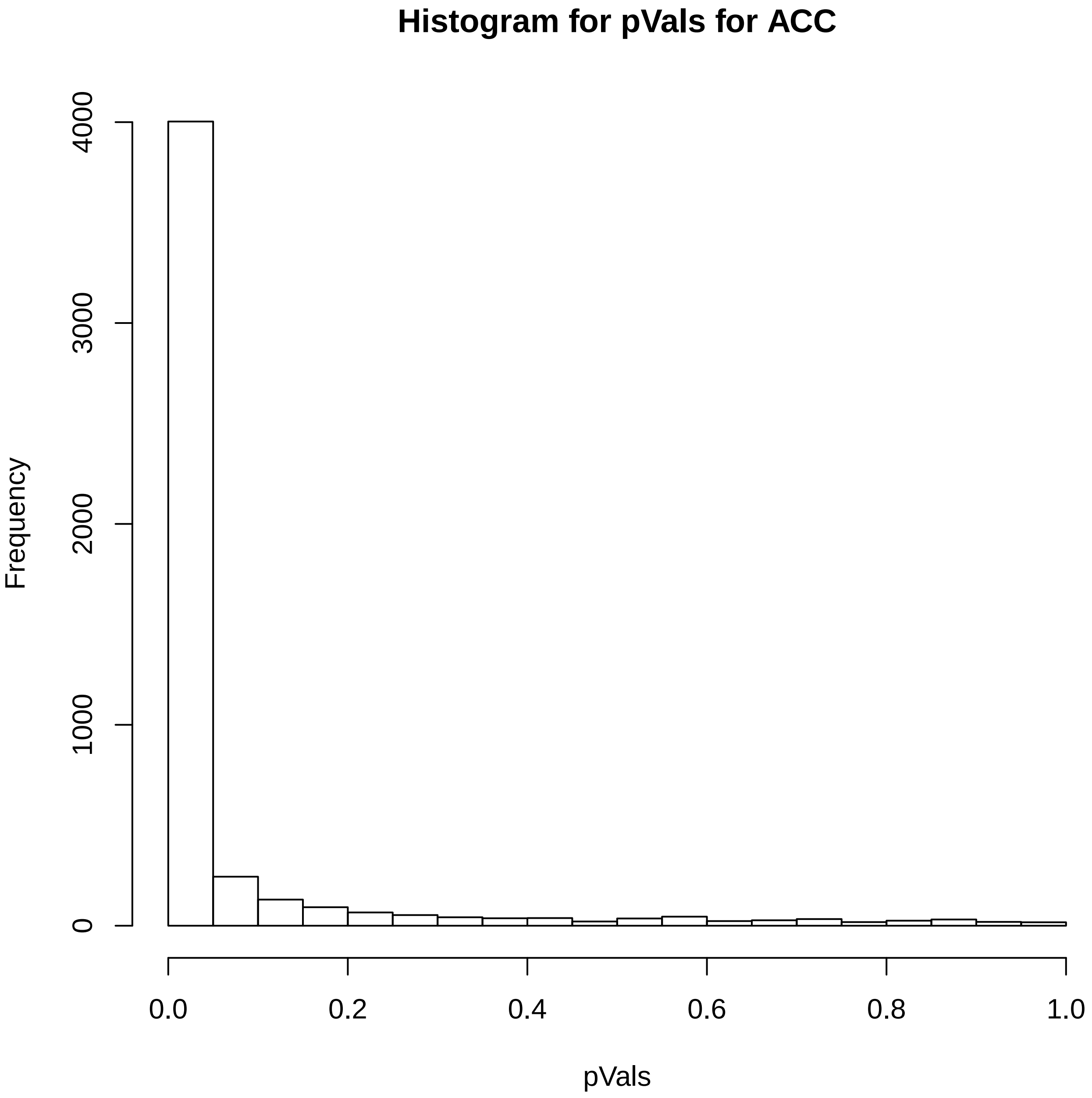
Histogram for pVals for ACC
Frequency
0
1000
2000
3000
4000
0.0
0.2
0.4
0.6
0.8
1.0
pVals

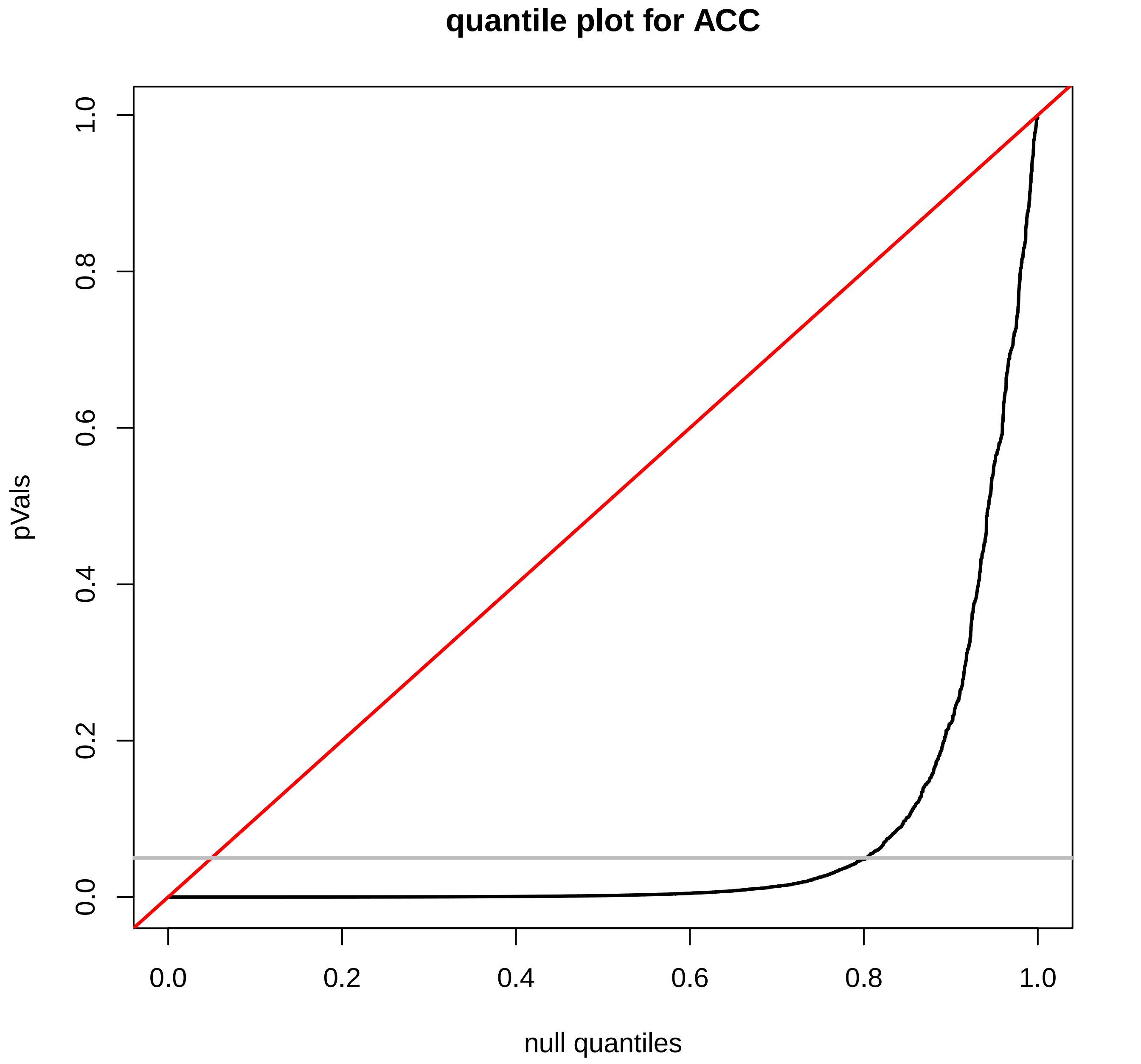
quantile plot for ACC
pVals
0.0
0.2
0.4
0.6
0.8
1.0
null quantiles

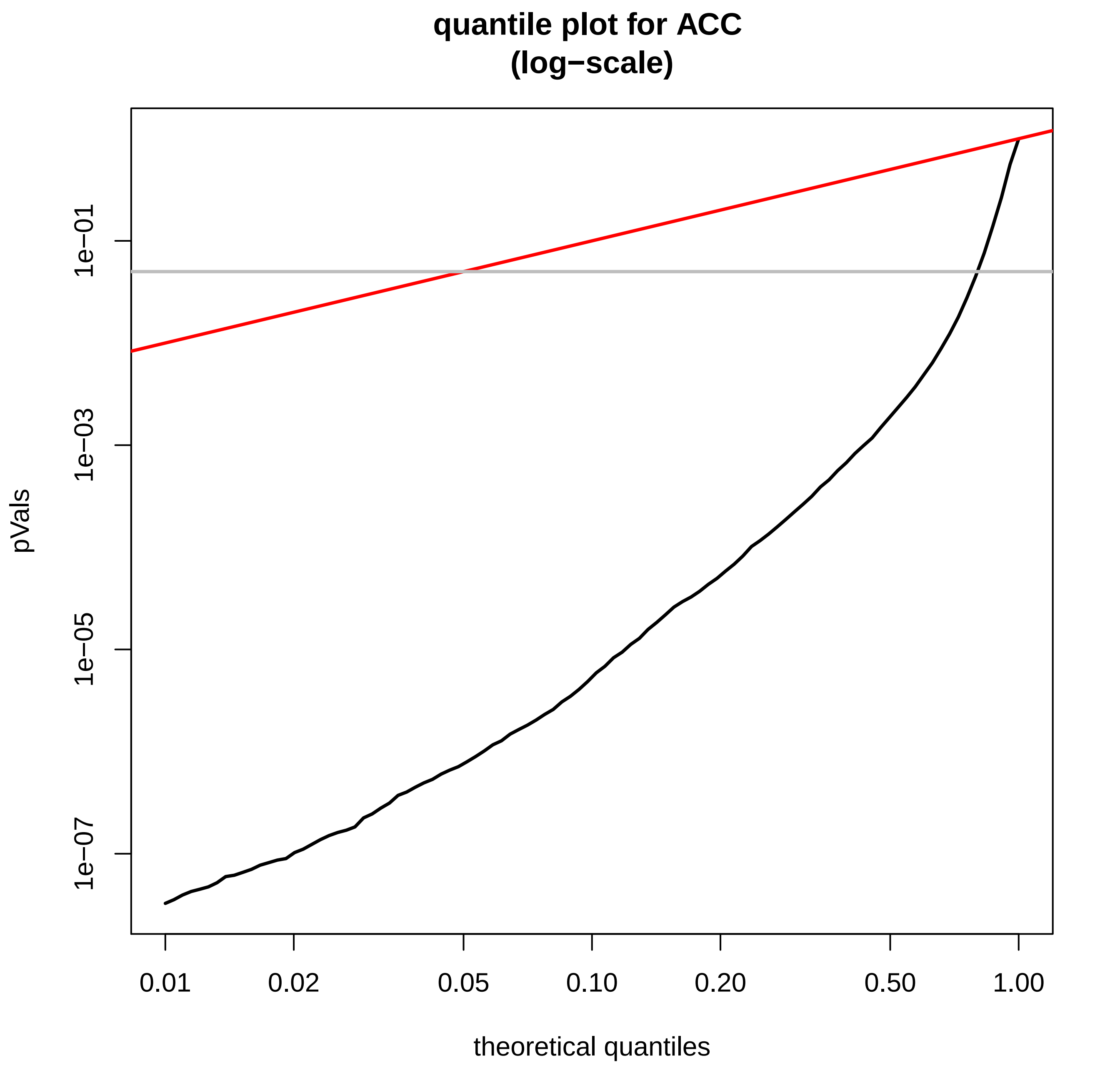

quantile plot for ACC
(log−scale)
pVals
1e−01
1e−03
1e−05
1e−07
0.01
0.02
0.05
0.10
0.20
0.50
1.00
theoretical quantiles

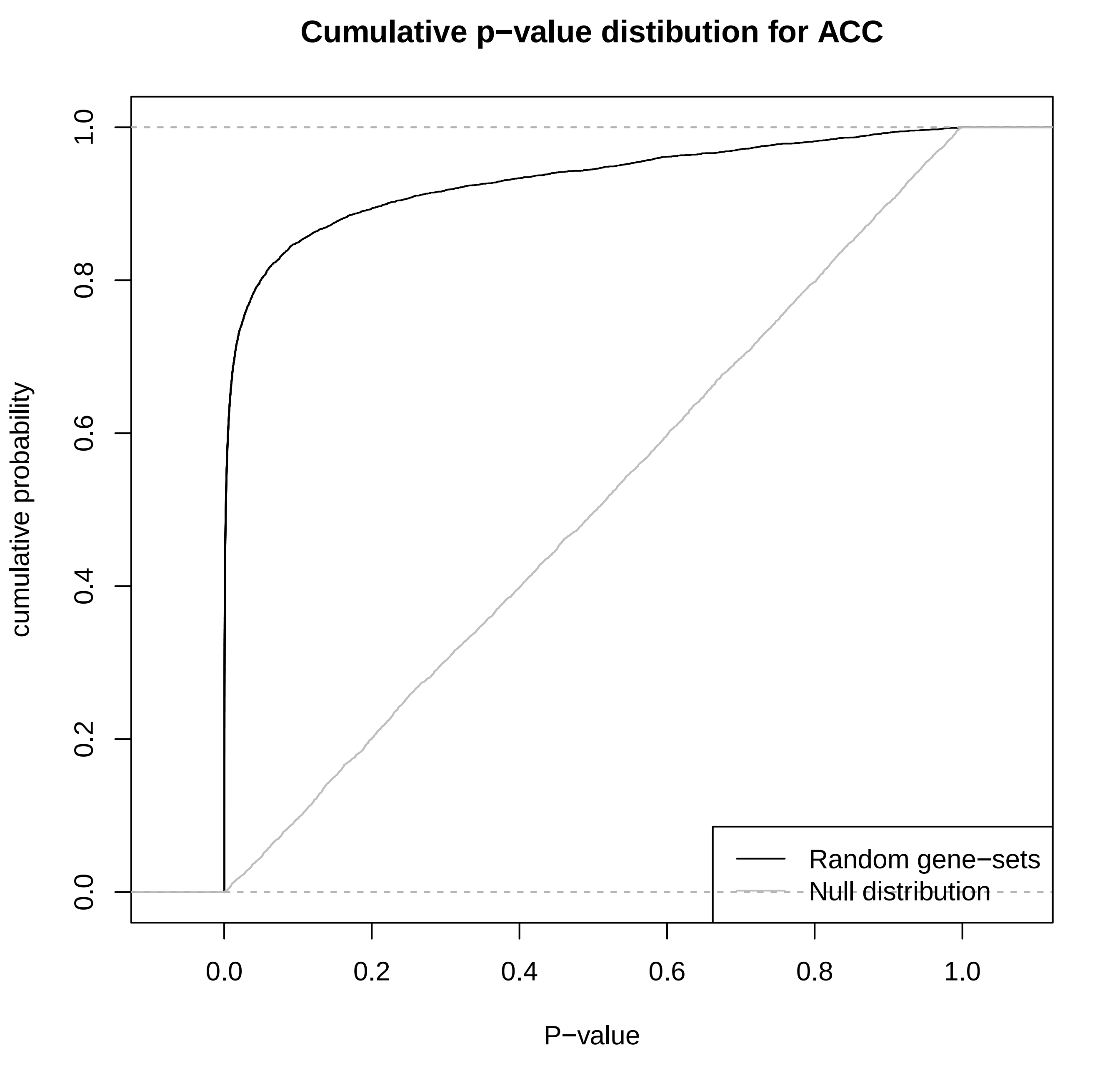

Cumulative p−value distibution for ACC
cumulative probability
P−value
0.0
0.2
0.4
0.6
0.8
1.0
Random gene−sets
Null distribution

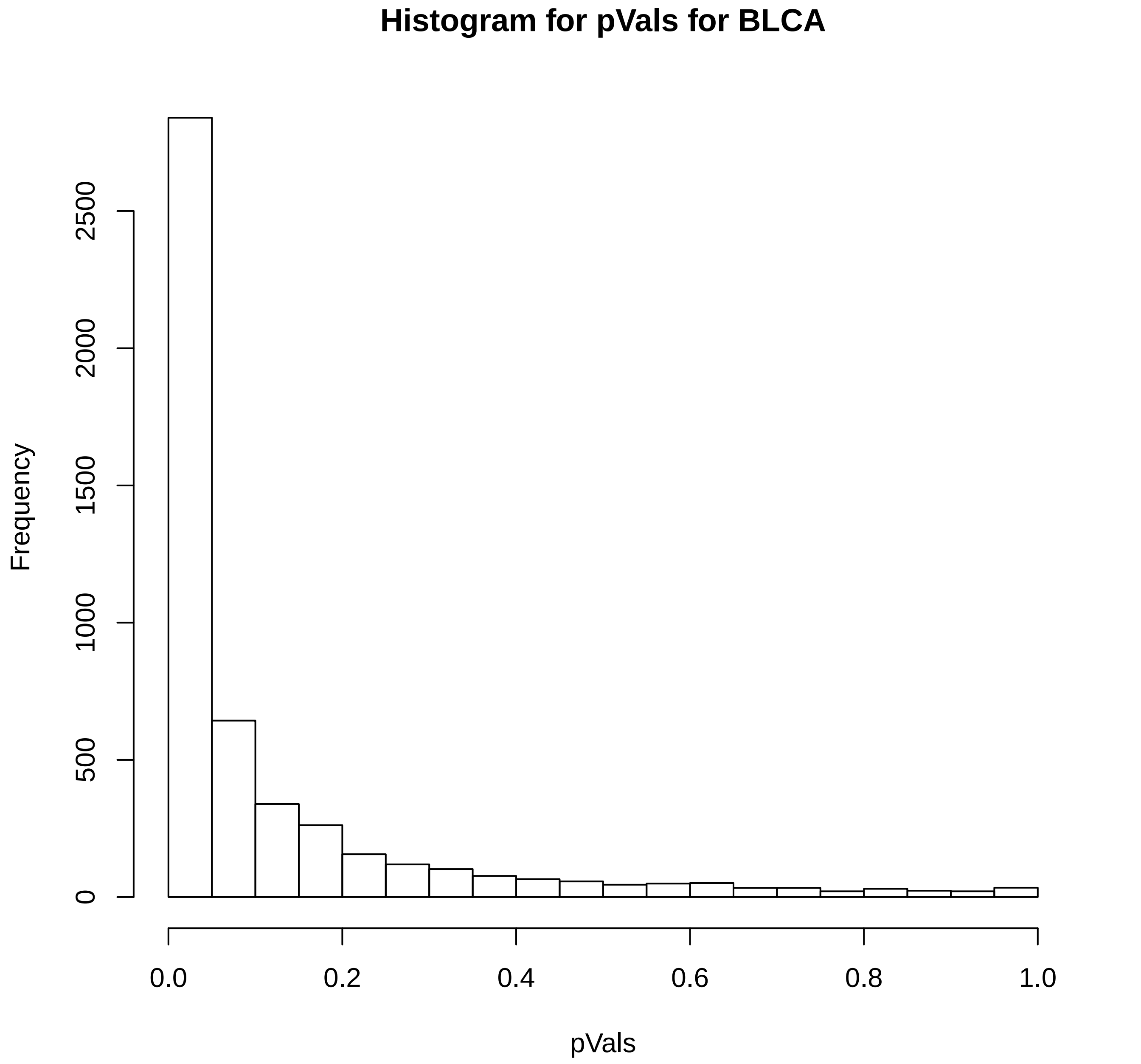
Histogram for pVals for BLCA
Frequency
0
500
1000
1500
2000
2500
0.0
0.2
0.4
0.6
0.8
1.0
pVals

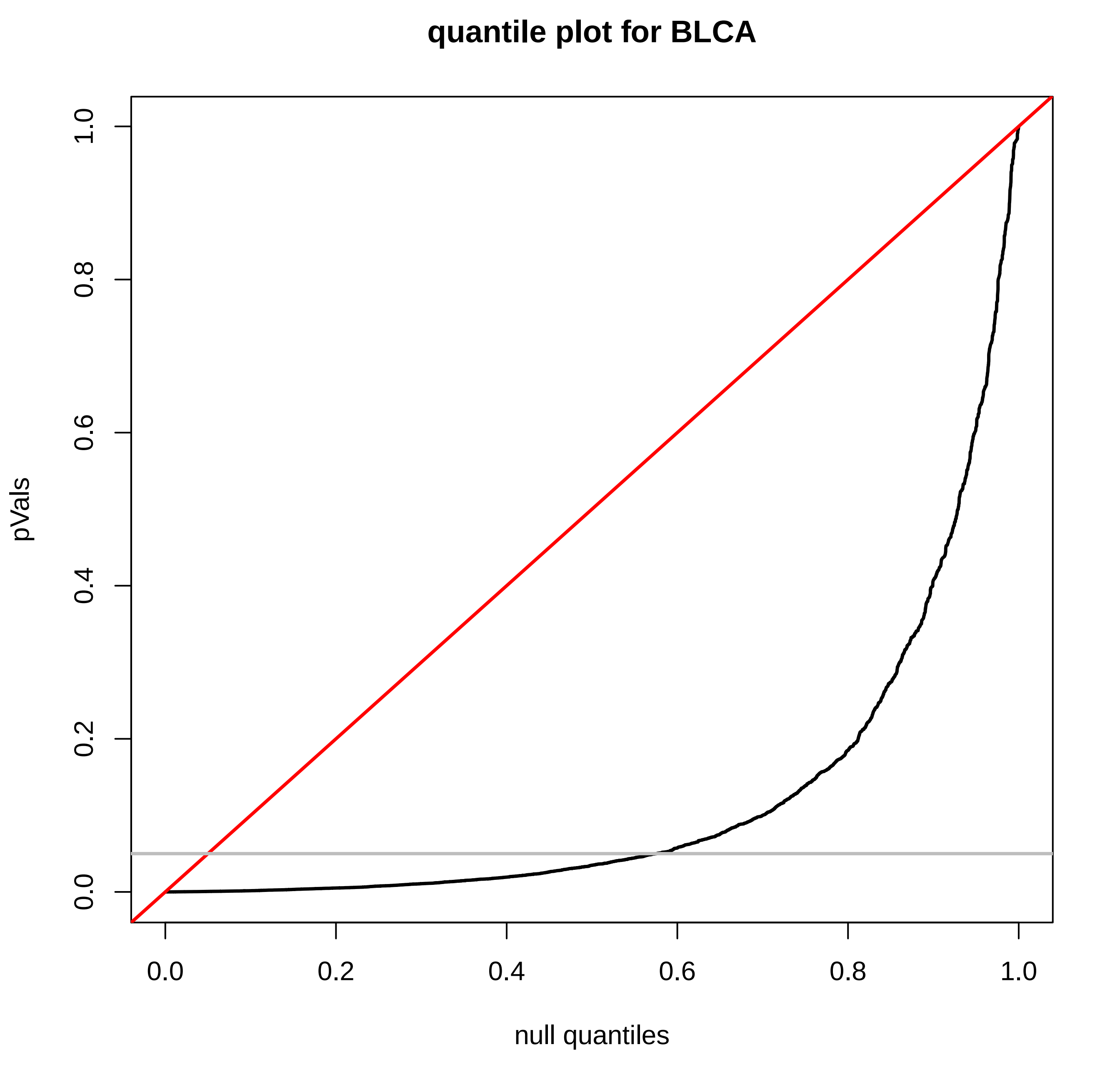
quantile plot for BLCA
pVals
0.0
0.2
0.4
0.6
0.8
1.0
0.0
0.2
0.4
0.6
0.8
1.0
null quantiles

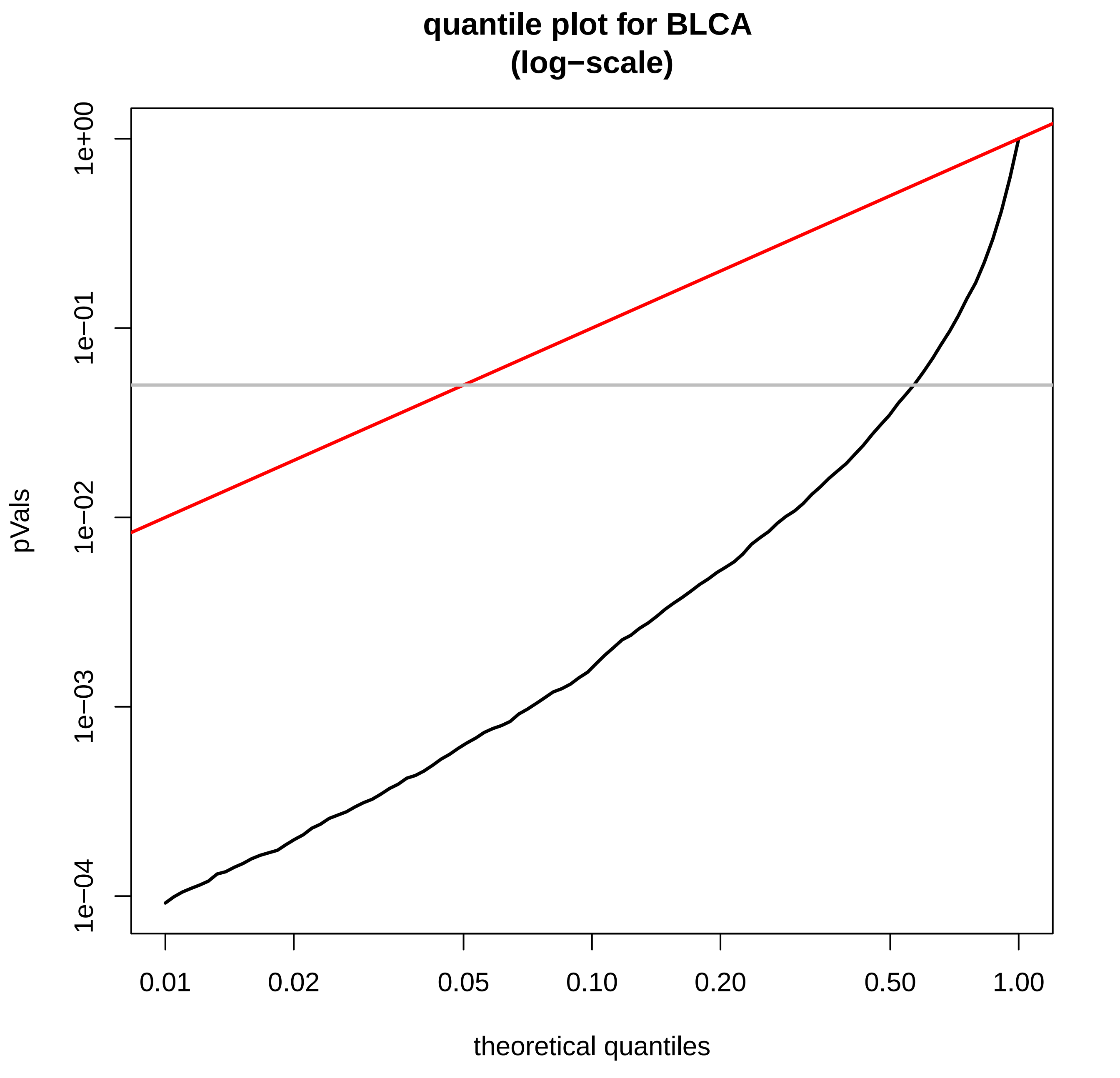
quantile plot for BLCA
(log−scale)
pVals
1e+00
1e−01
1e−02
1e−03
1e−04
0.01
0.02
0.05
0.10
0.20
0.50
1.00
theoretical quantiles

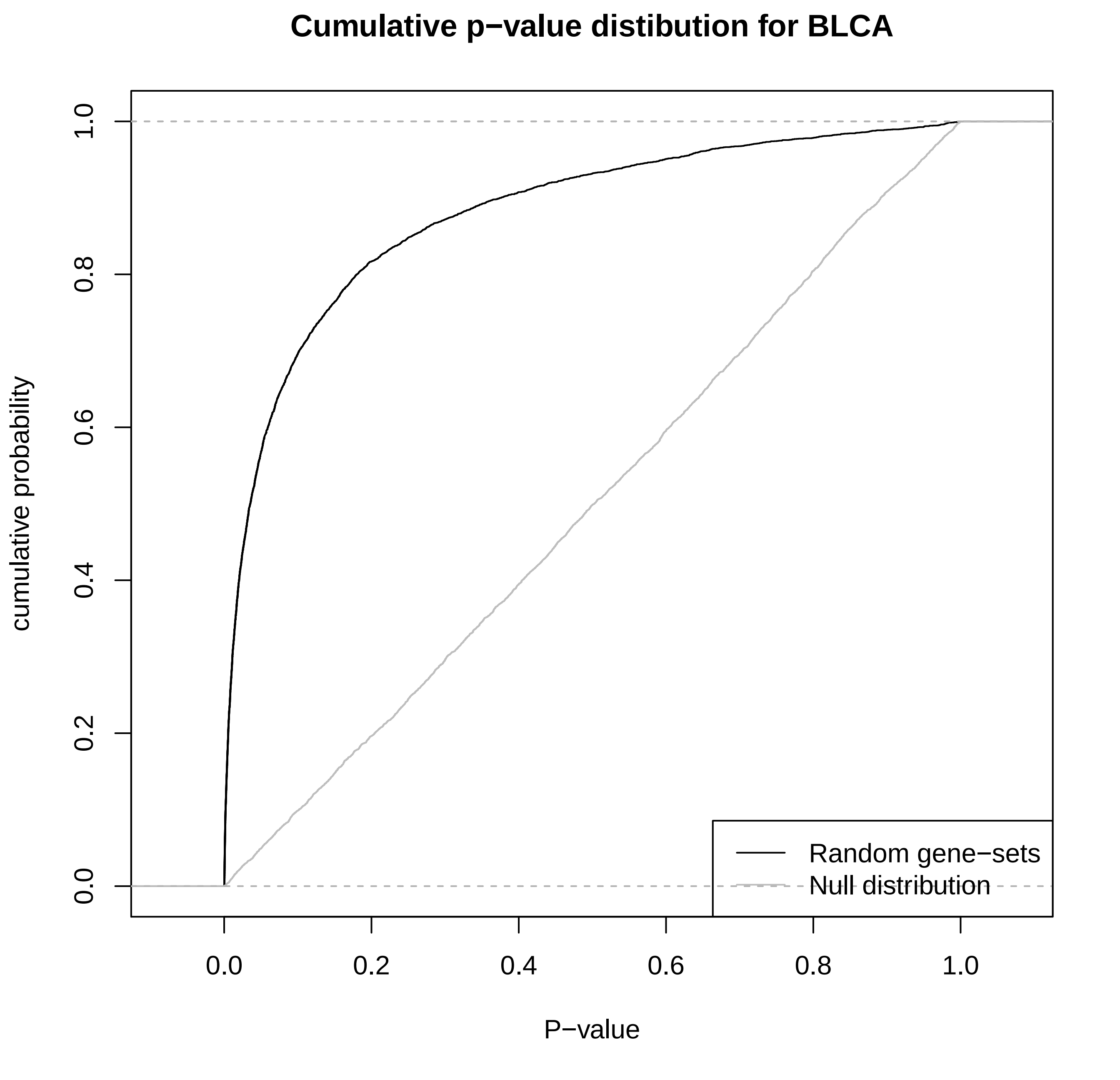
Cumulative p−value distibution for BLCA
cumulative probability
P−value
0.0
0.2
0.4
0.6
0.8
1.0
Random gene−sets
Null distribution

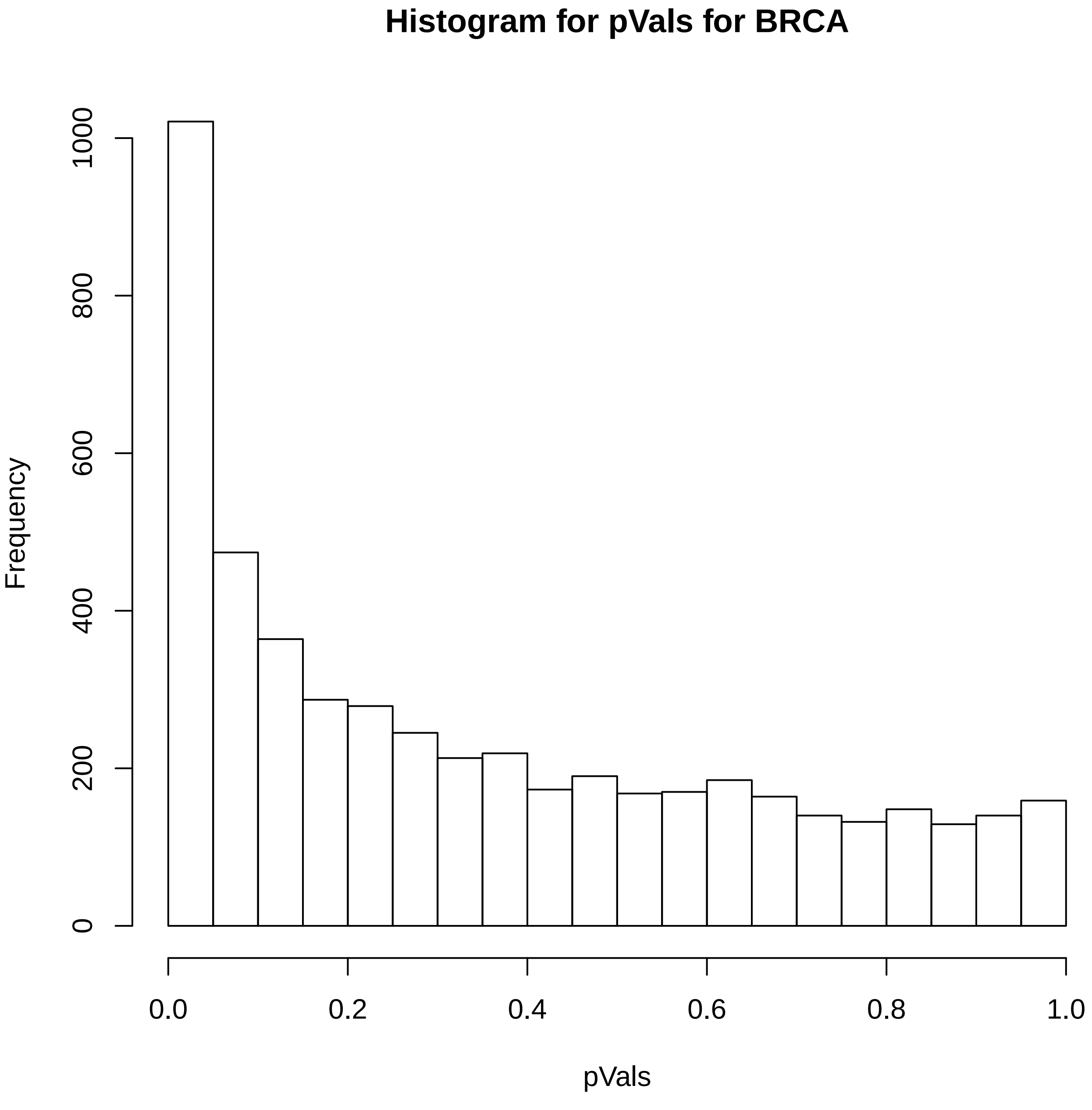
Histogram for pVals for BRCA
Frequency
0
200
400
600
800
1000
0.0
0.2
0.4
0.6
0.8
1.0
pVals

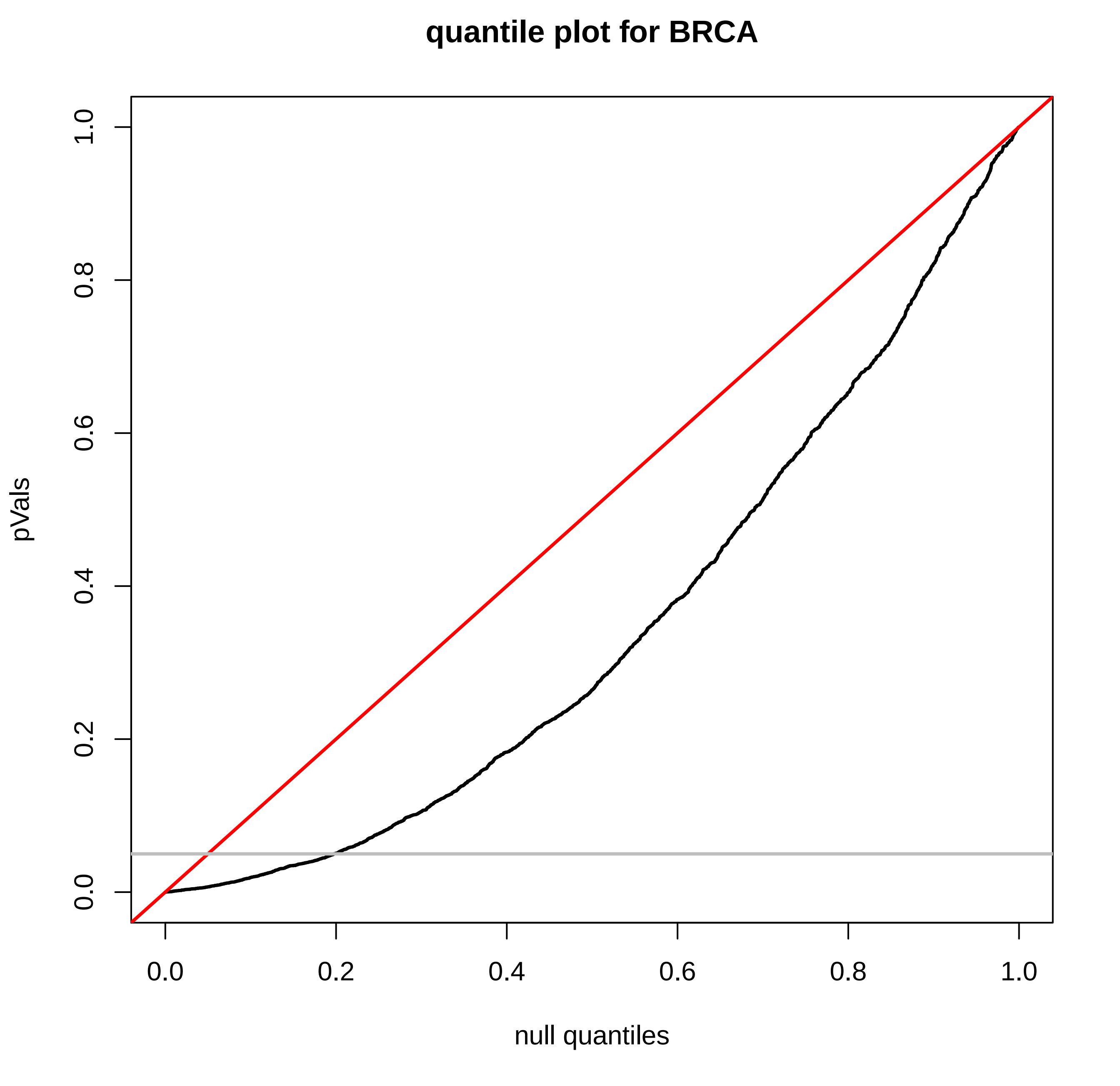
quantile plot for BRCA
pVals
null quantiles
0.0
0.2
0.4
0.6
0.8
1.0
0.0
0.2
0.4
0.6
0.8
1.0

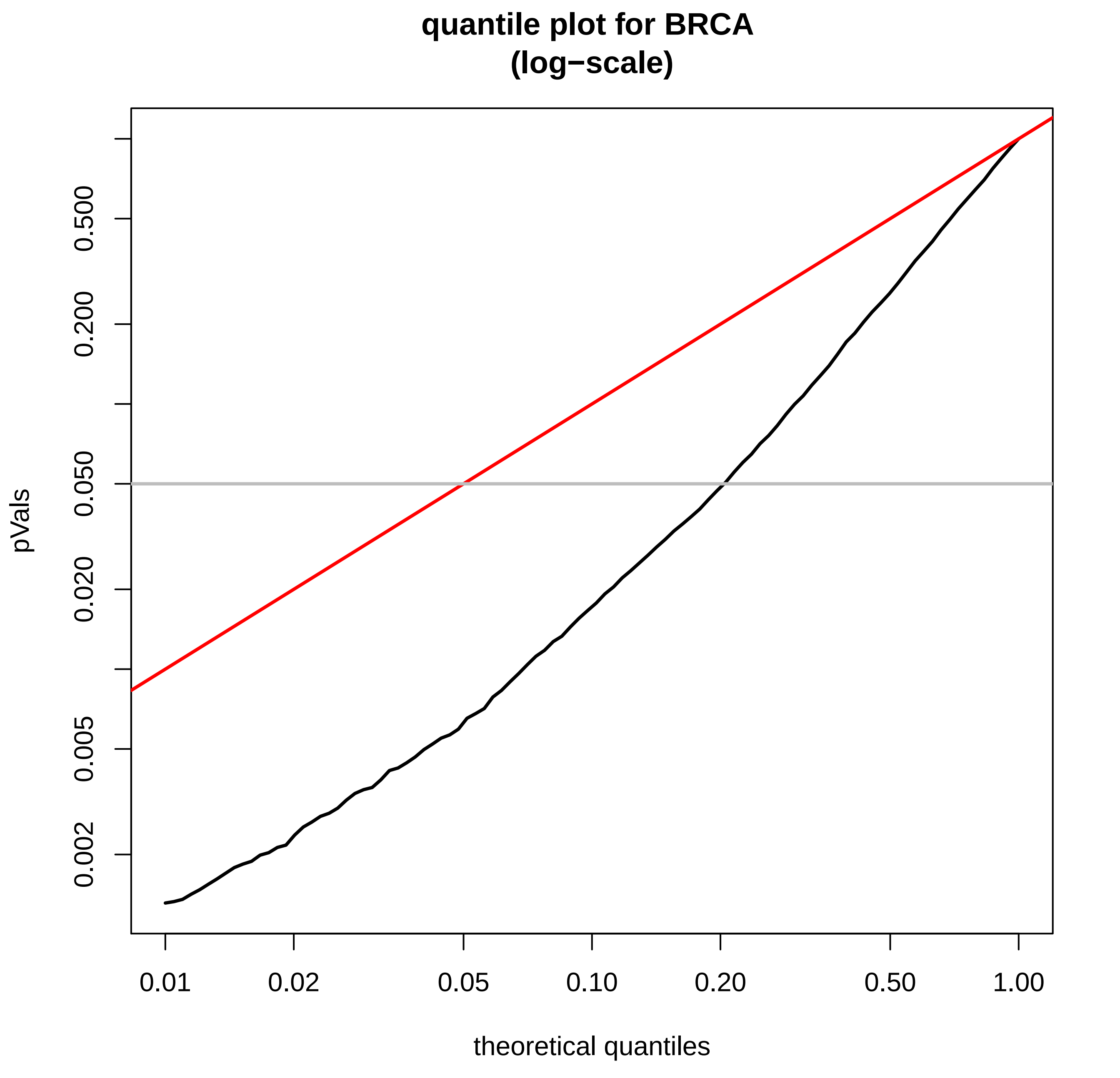

quantile plot for BRCA
(log−scale)
pVals
0.002
0.005
0.020
0.050
0.200
0.500
0.01
0.02
0.05
0.10
0.20
0.50
1.00
theoretical quantiles

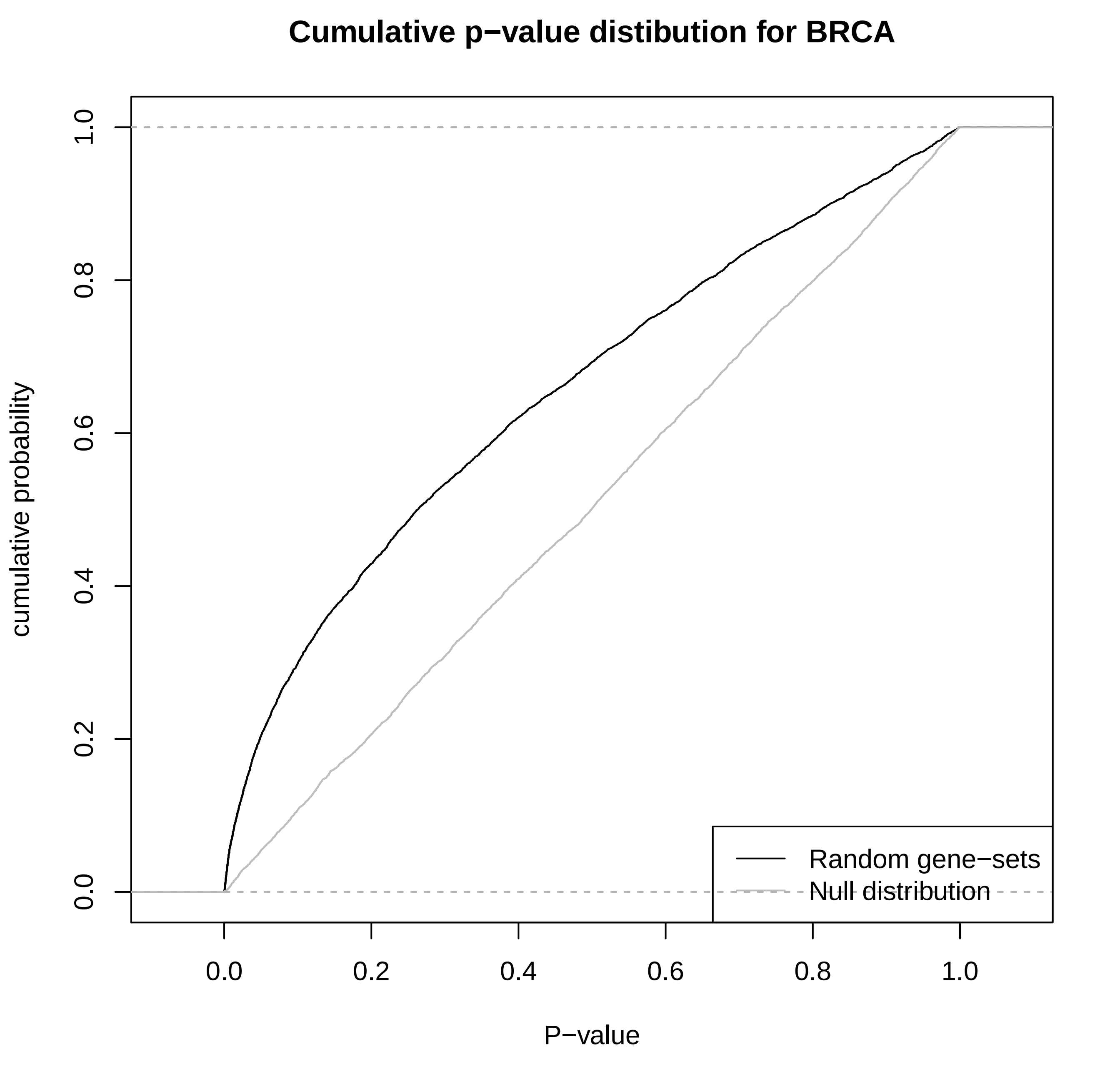

Cumulative p−value distibution for BRCA
cumulative probability
0.0
0.2
0.4
0.6
0.8
1.0
P−value
0.0
0.2
0.4
0.6
0.8
1.0
Random gene−sets
Null distribution

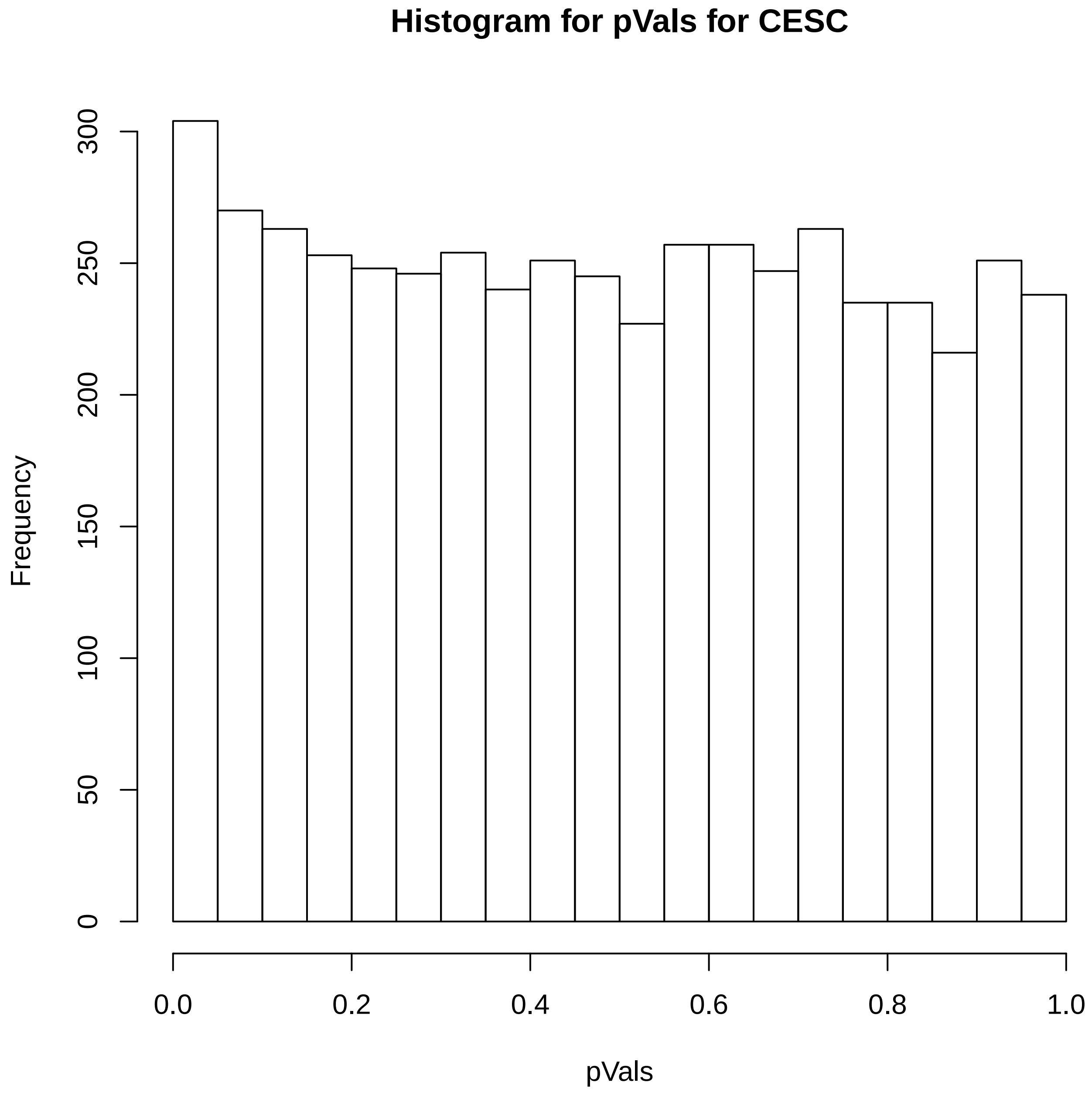
Histogram for pVals for CESC
Frequency
0
50
100
150
200
250
300
0.0
0.2
0.4
0.6
0.8
1.0
pVals

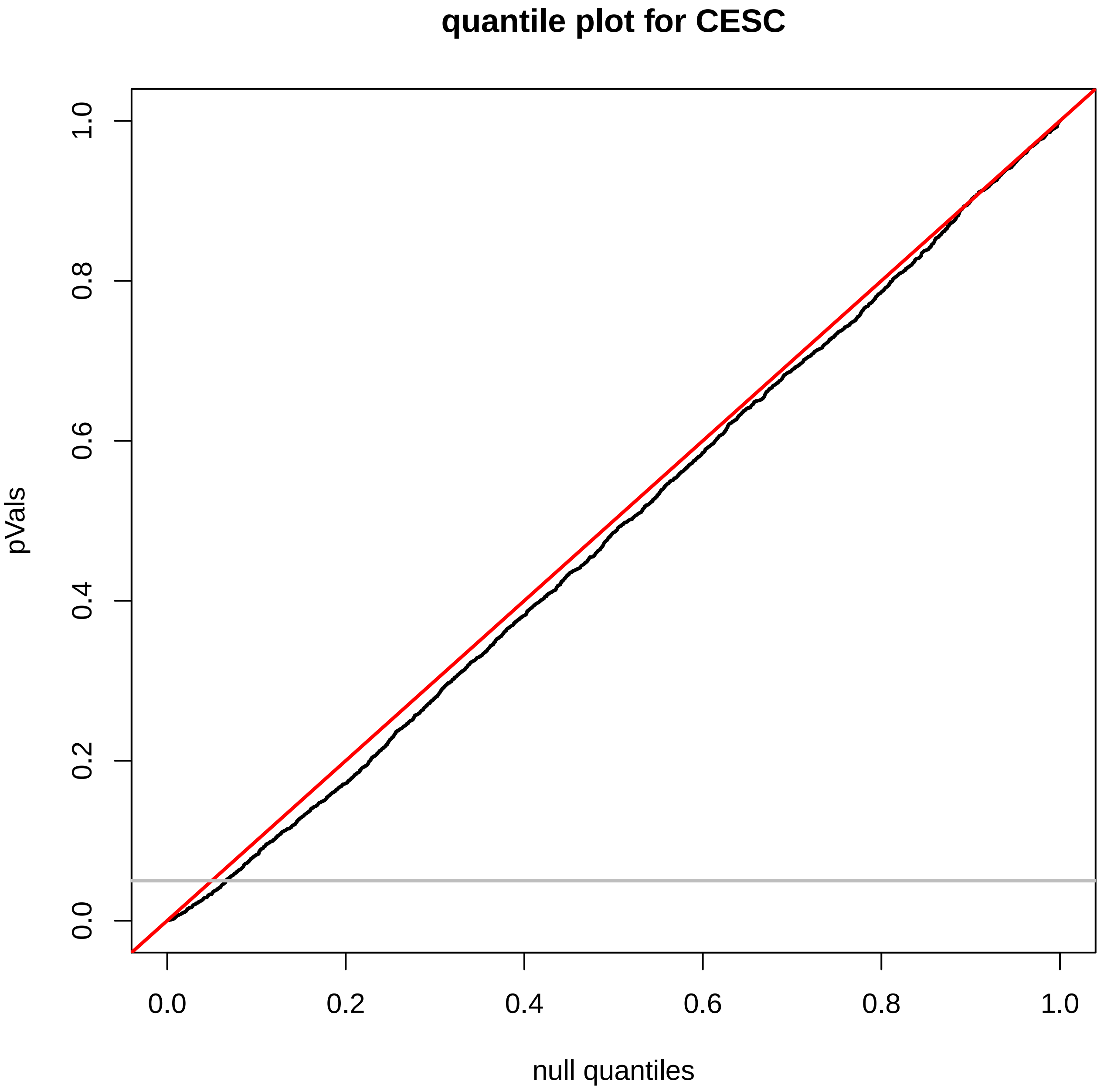
quantile plot for CESC
pVals
null quantiles
0.0
0.2
0.4
0.6
0.8
1.0

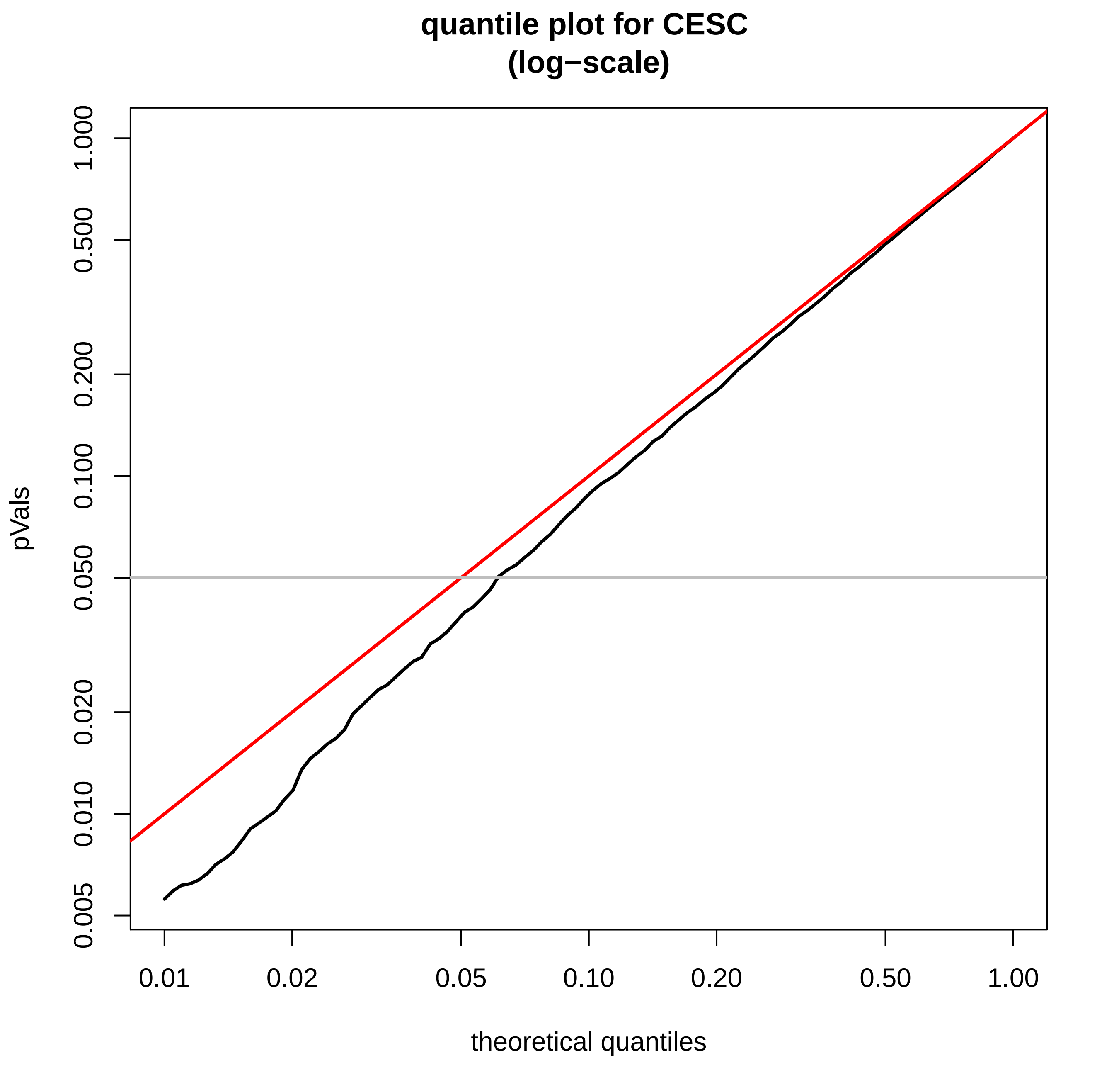
quantile plot for CESC
(log−scale)
pVals
1.000
0.500
0.200
0.100
0.050
0.020
0.010
0.005
0.01
0.02
0.05
0.10
0.20
0.50
1.00
theoretical quantiles

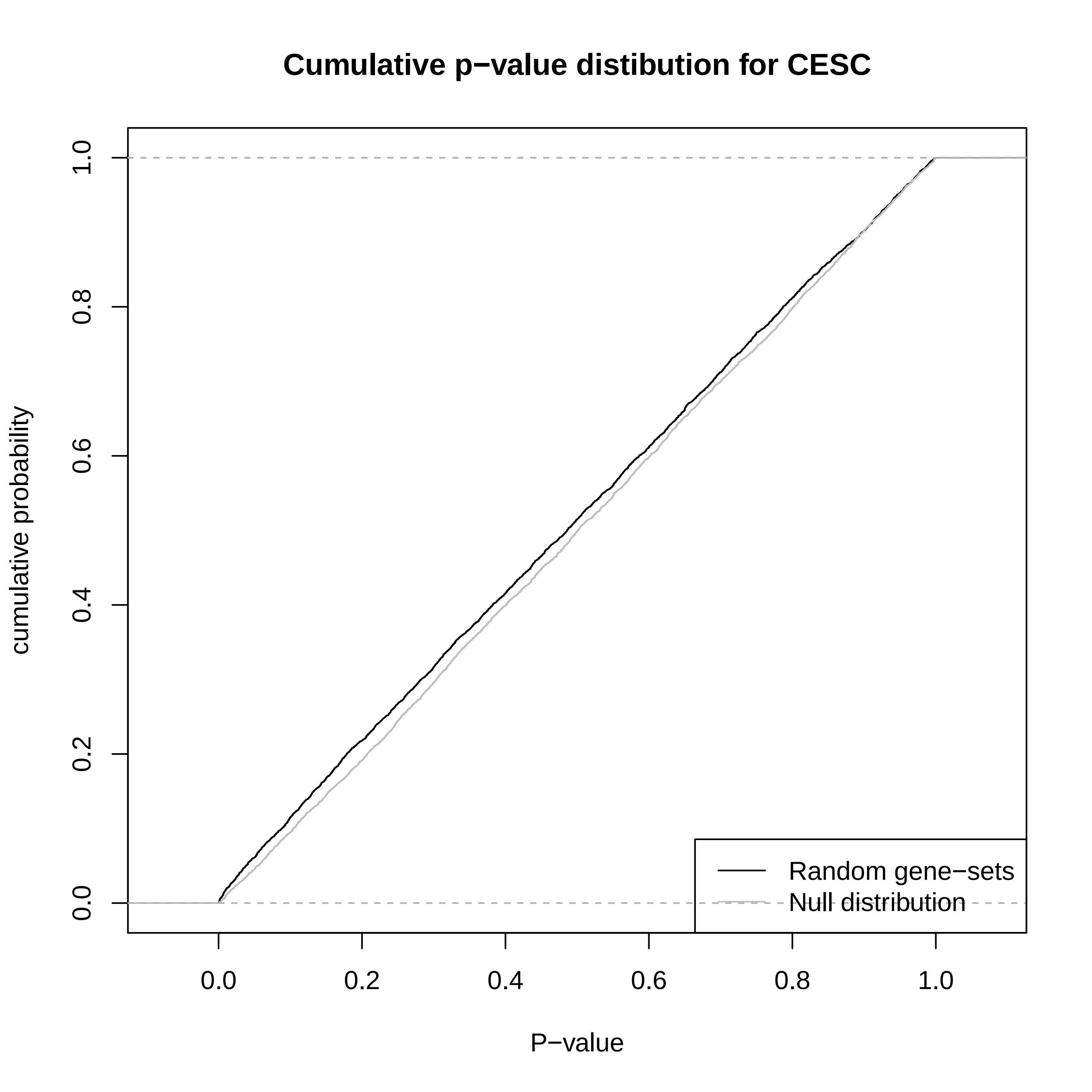
Cumulative p−value distibution for CESC
cumulative probability
0.0
0.2
0.4
0.6
0.8
1.0
P−value
Random gene−sets
Null distribution

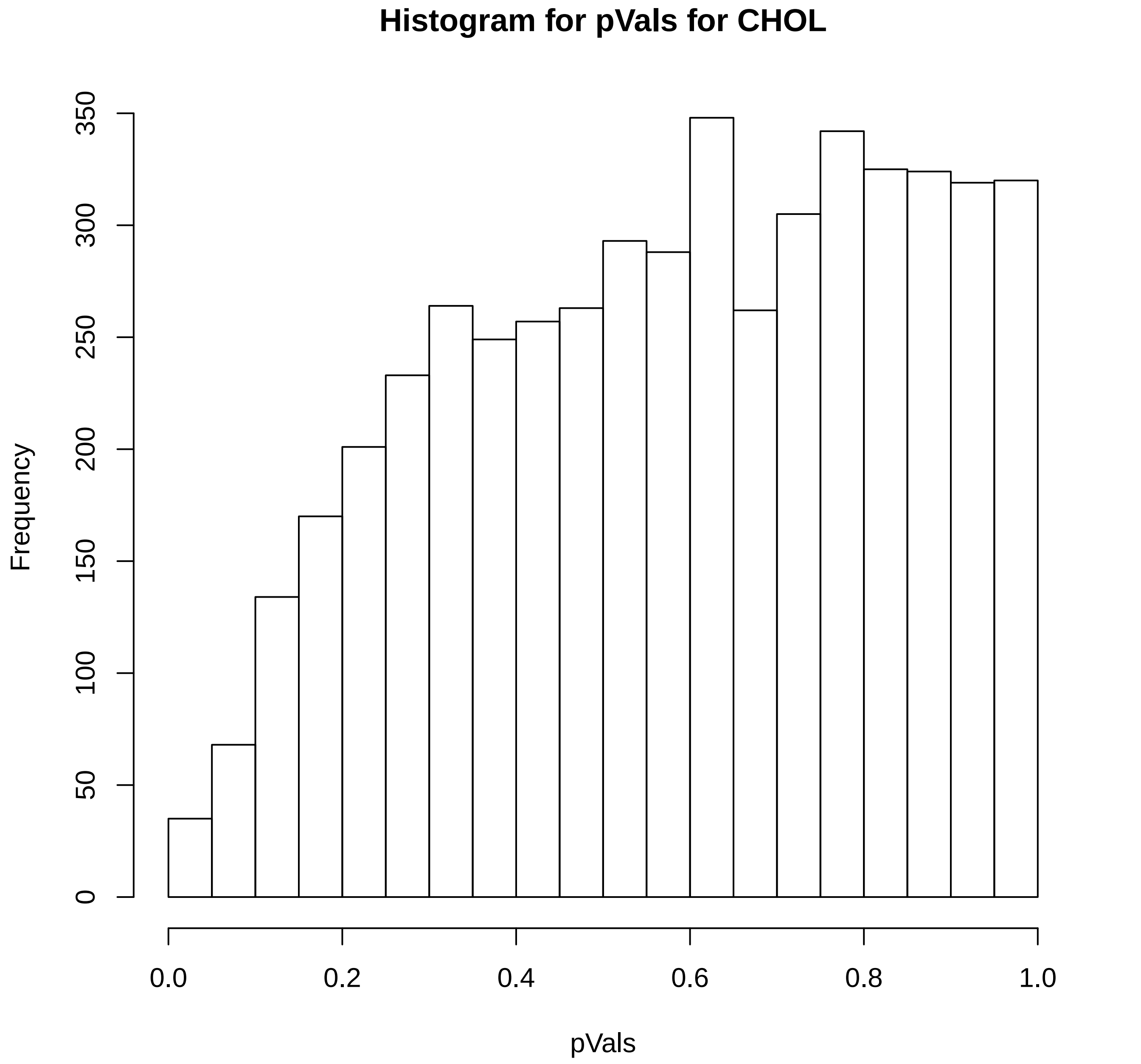
Histogram for pVals for CHOL
Frequency
0
50
100
150
200
250
300
350
0.0
0.2
0.4
0.6
0.8
1.0
pVals

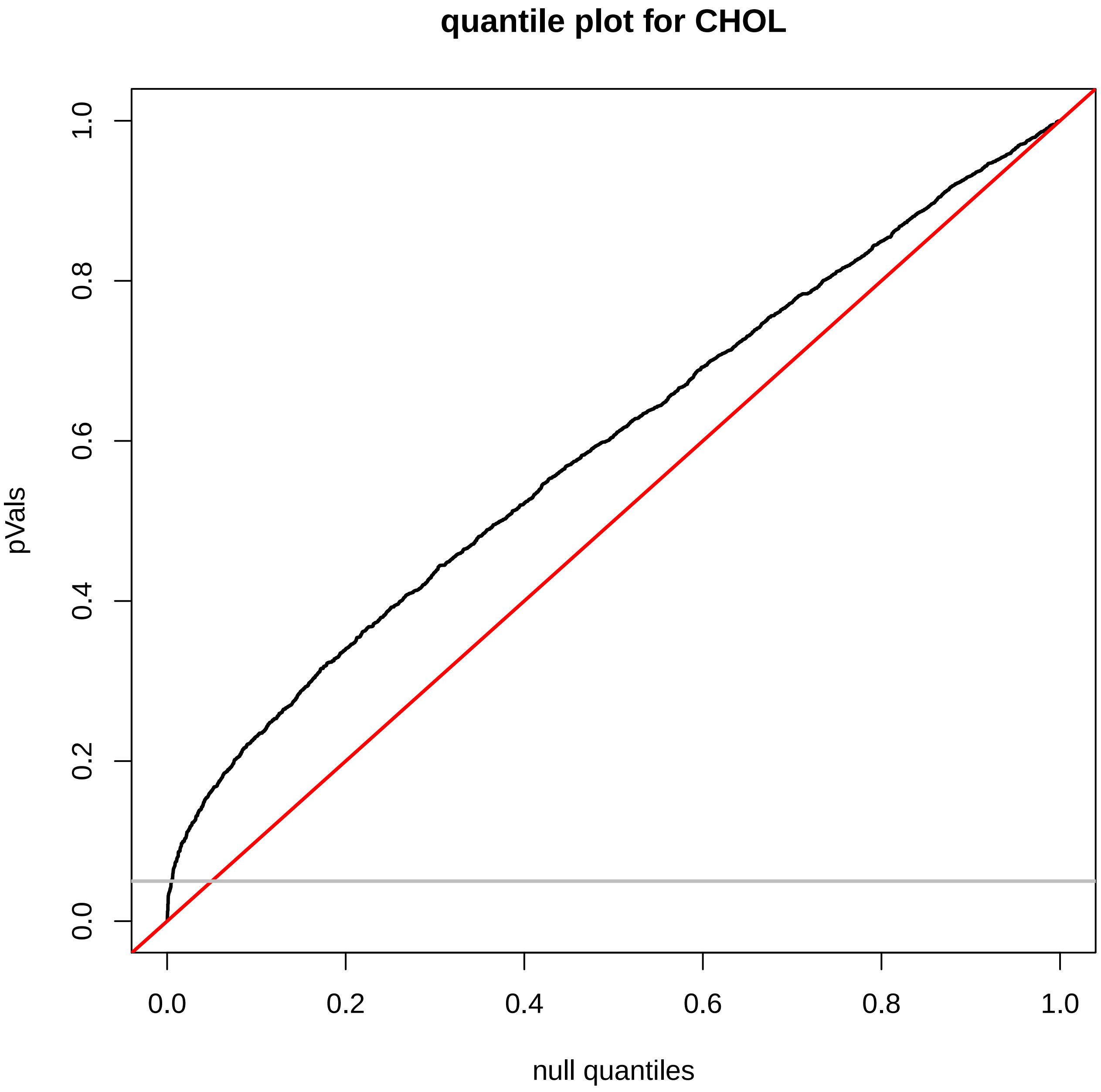
quantile plot for CHOL
pVals
null quantiles
0.0
0.2
0.4
0.6
0.8
1.0
0.0
0.2
0.4
0.6
0.8
1.0

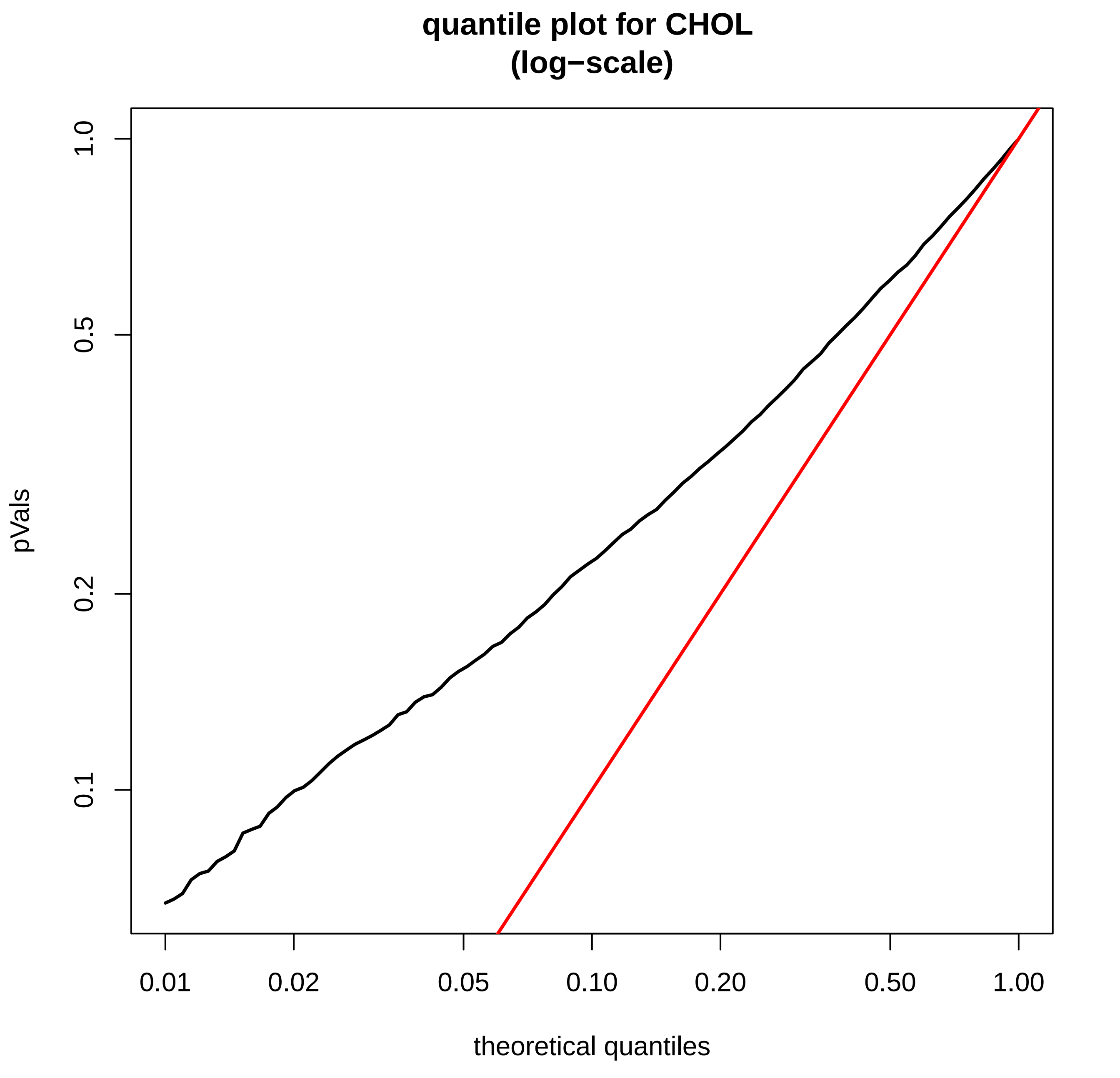
quantile plot for CHOL
(log−scale)
pVals
1.0
0.5
0.2
0.1
0.01
0.02
0.05
0.10
0.20
0.50
1.00
theoretical quantiles

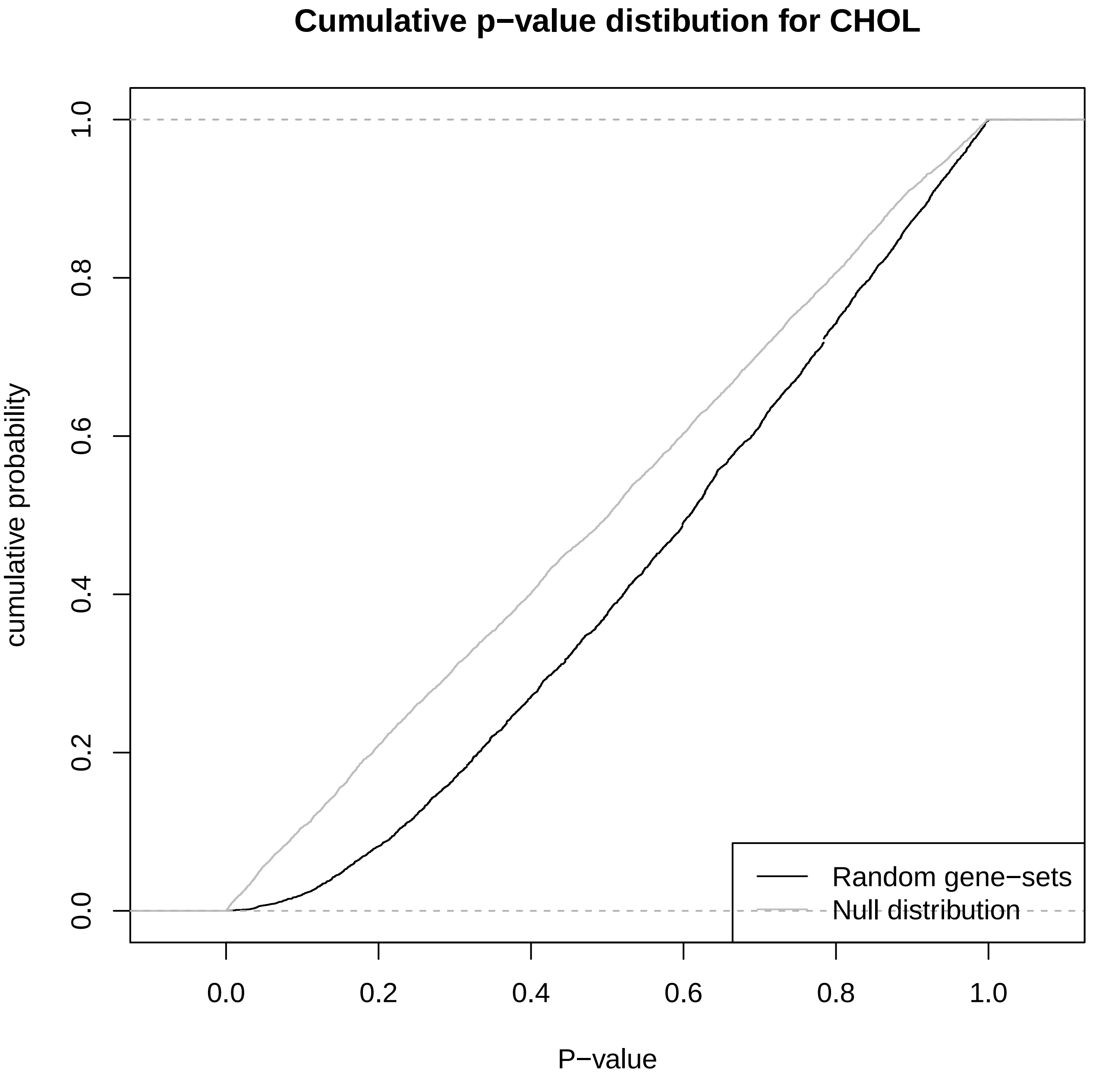

Cumulative p−value distibution for CHOL
cumulative probability
P−value
0.0
0.2
0.4
0.6
0.8
1.0
Random gene−sets
Null distribution

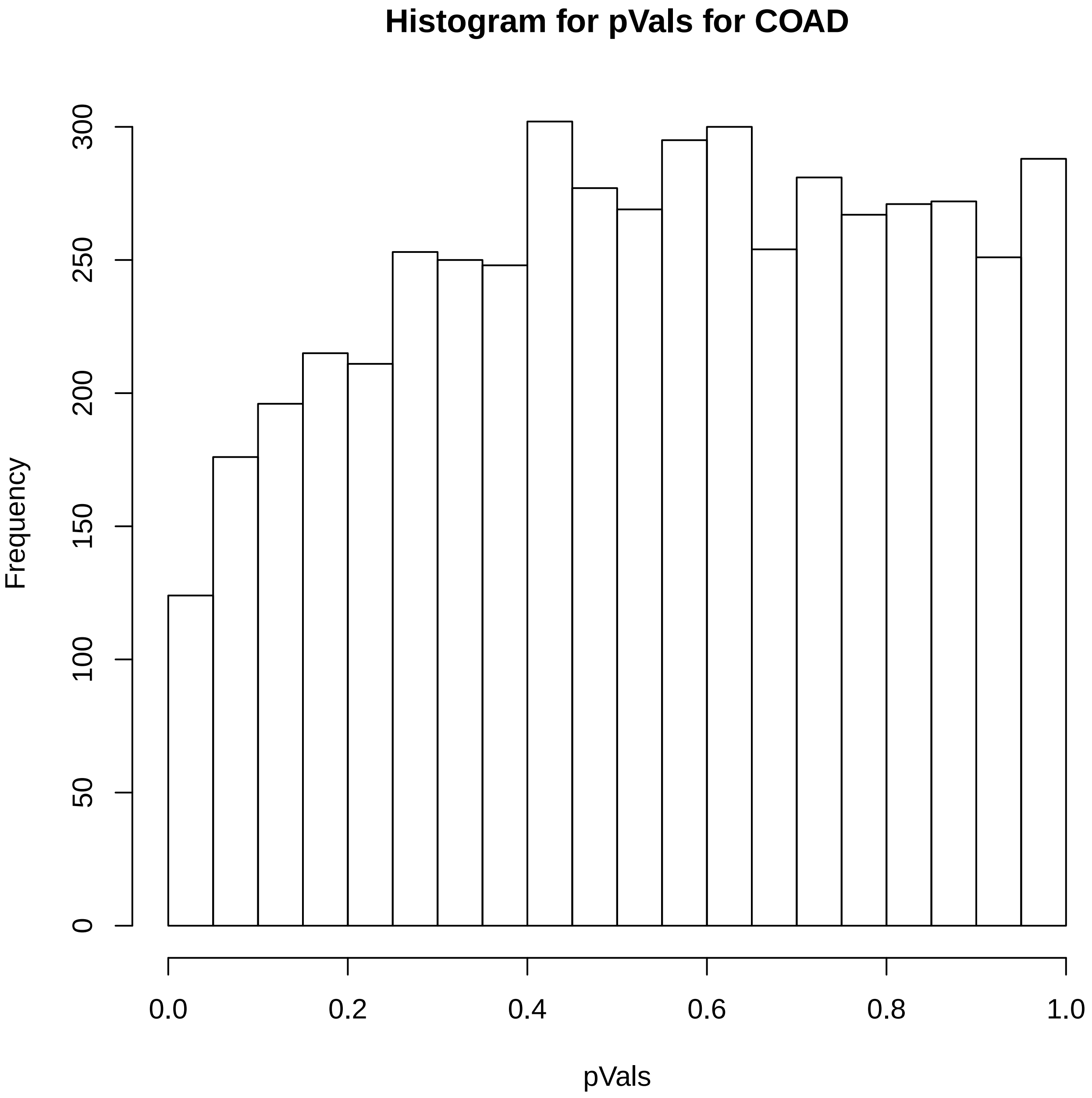

Histogram for pVals for COAD
Frequency
0
50
100
150
200
250
300
0.0
0.2
0.4
0.6
0.8
1.0
pVals

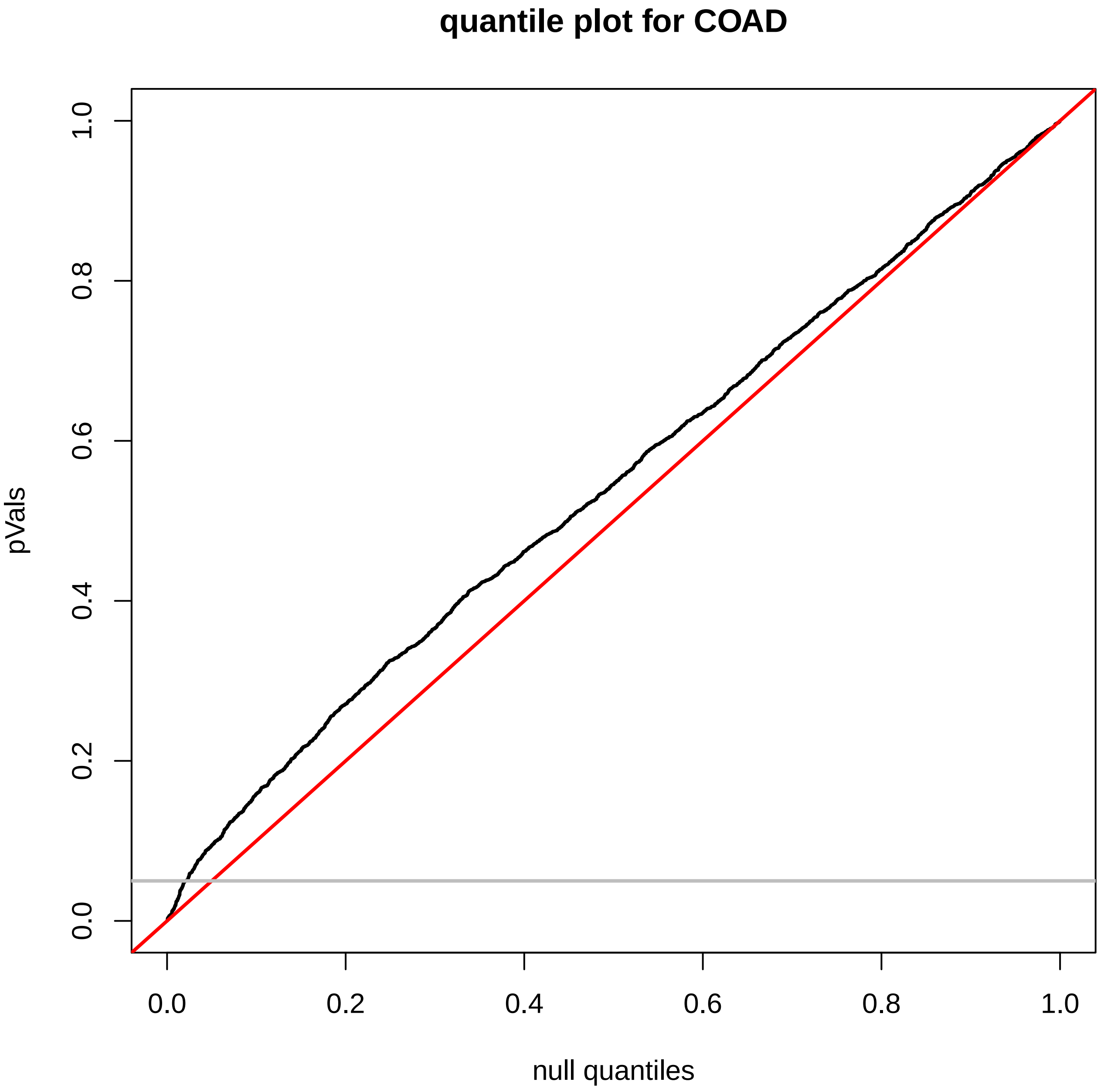

quantile plot for COAD
pVals
null quantiles
0.0
0.2
0.4
0.6
0.8
1.0

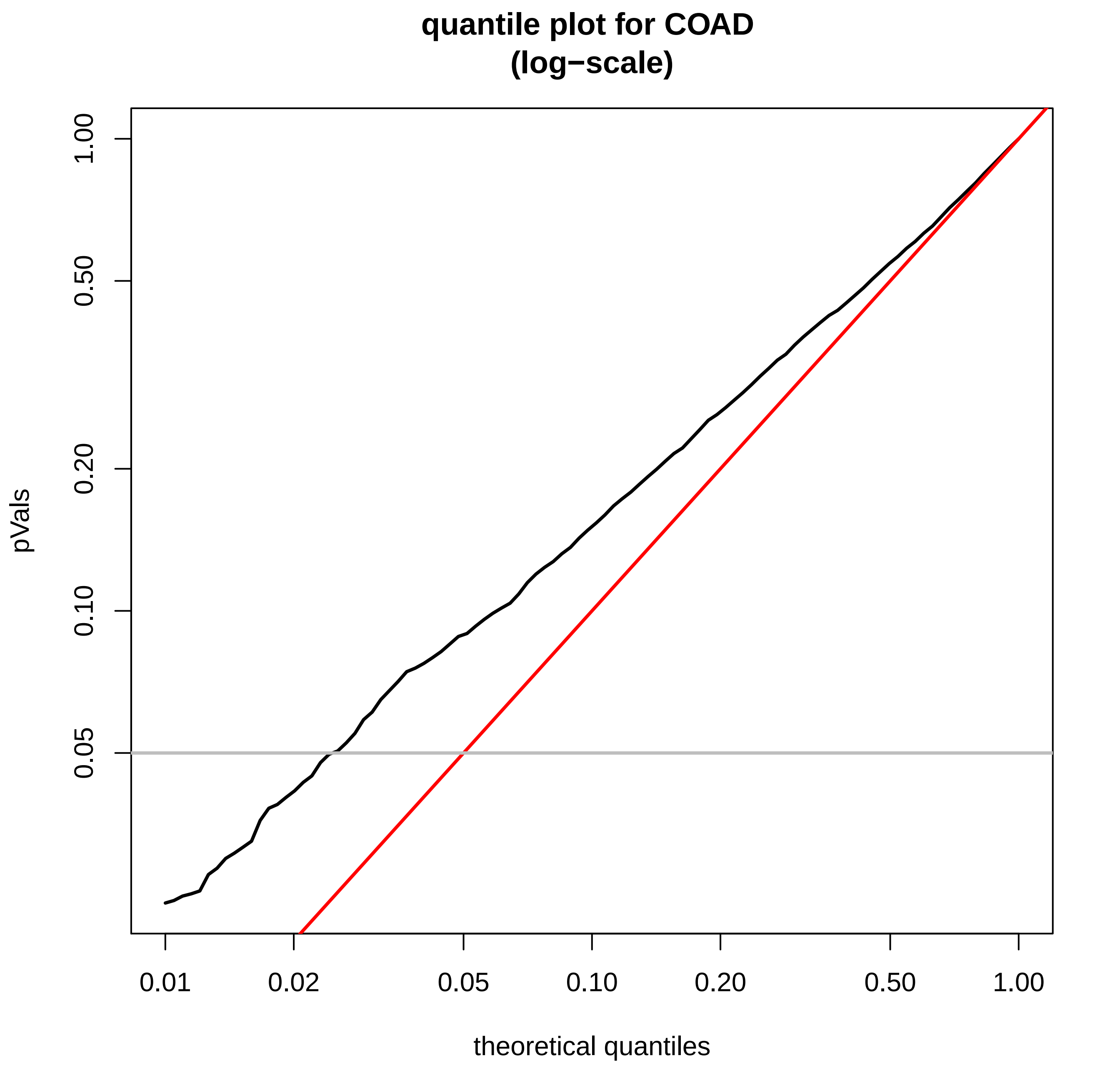
quantile plot for COAD
(log−scale)
1.00
0.50
0.20
0.10
0.05
pVals
0.01
0.02
0.05
0.10
0.20
0.50
1.00
theoretical quantiles

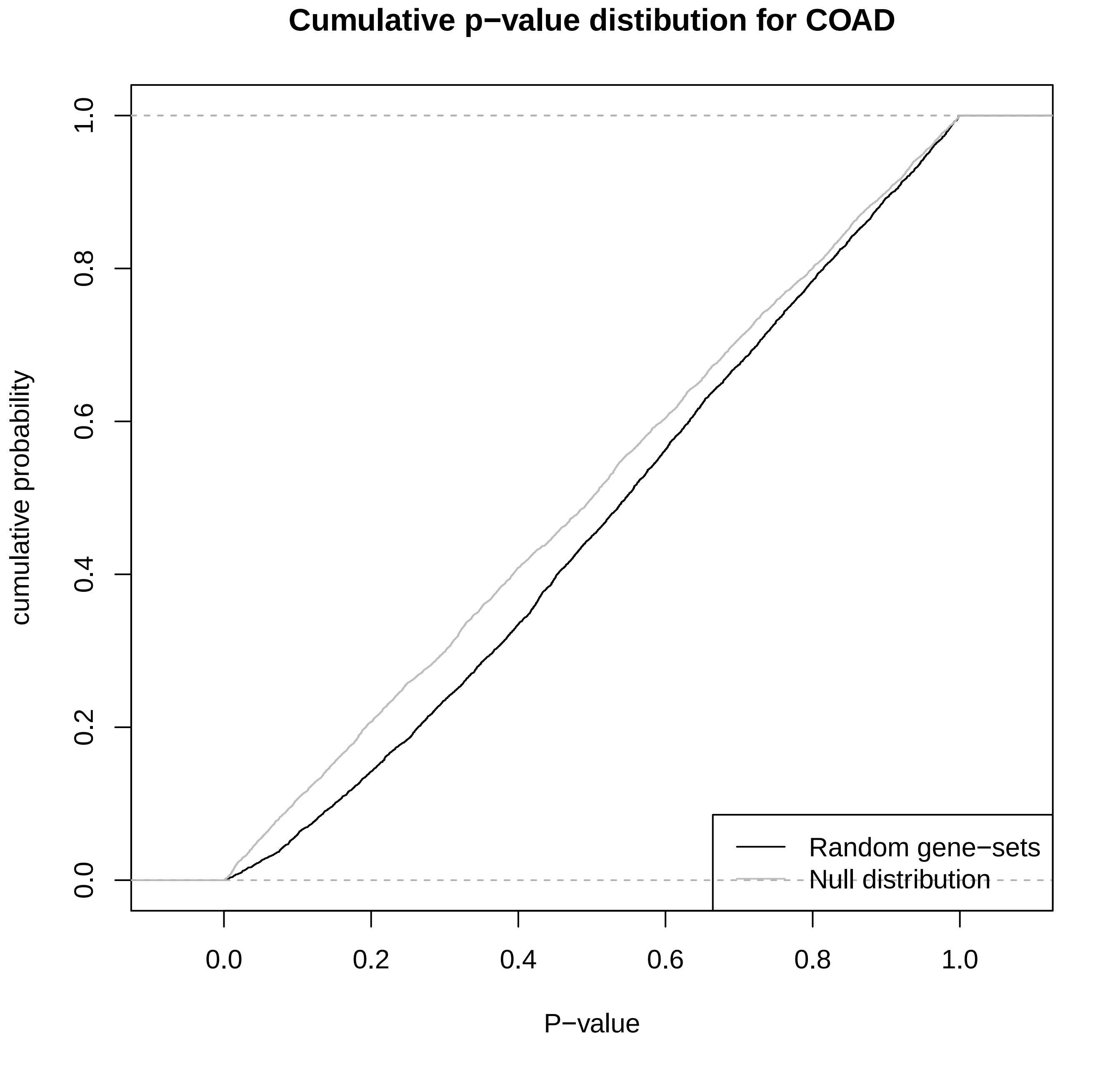
Cumulative p−value distibution for COAD
cumulative probability
0.0
0.2
0.4
0.6
0.8
1.0
P−value
0.0
0.2
0.4
0.6
0.8
1.0
Random gene−sets
Null distribution

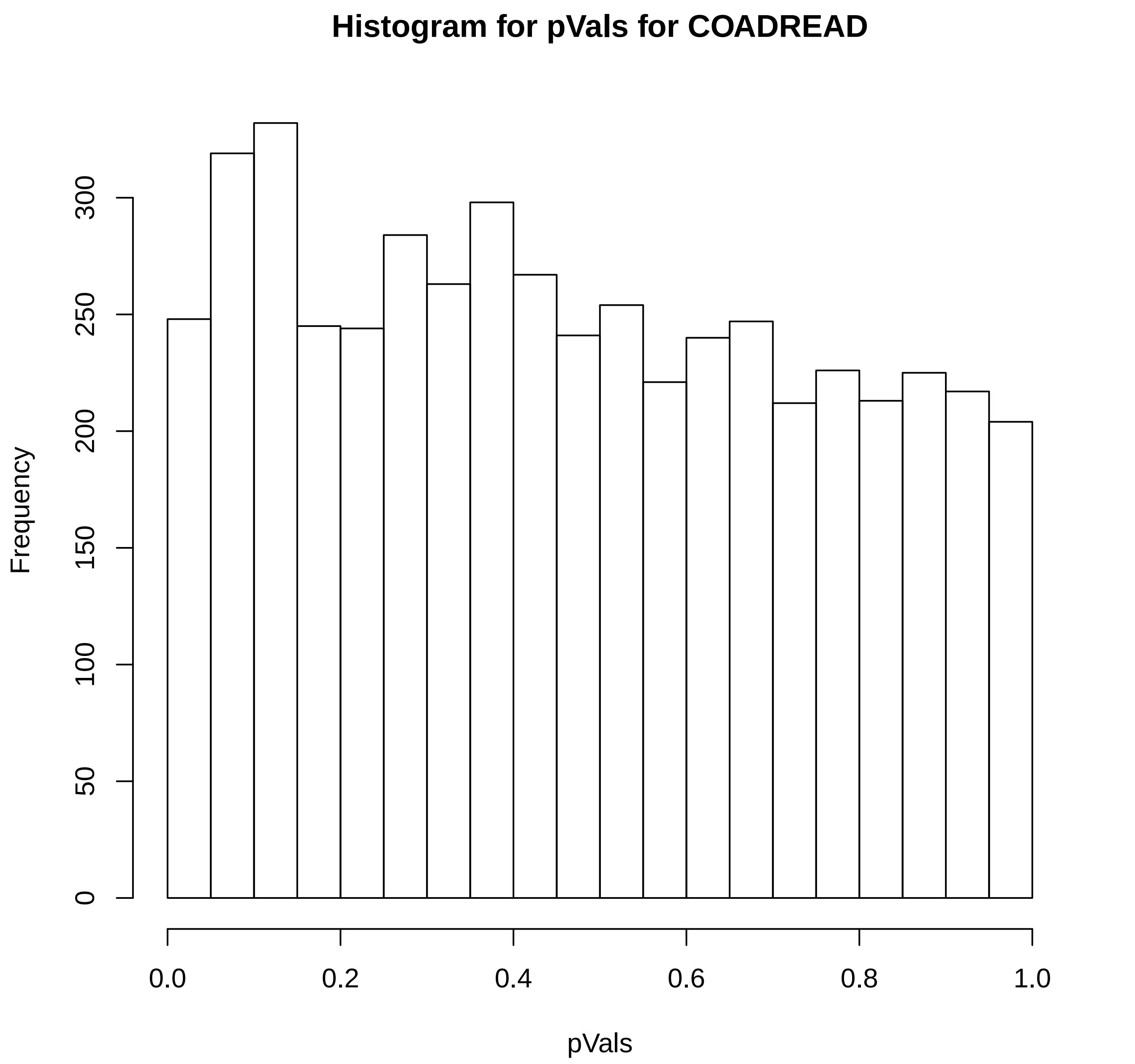
Histogram for pVals for COADREAD
Frequency
0
50
100
150
200
250
300
0.0
0.2
0.4
0.6
0.8
1.0
pVals

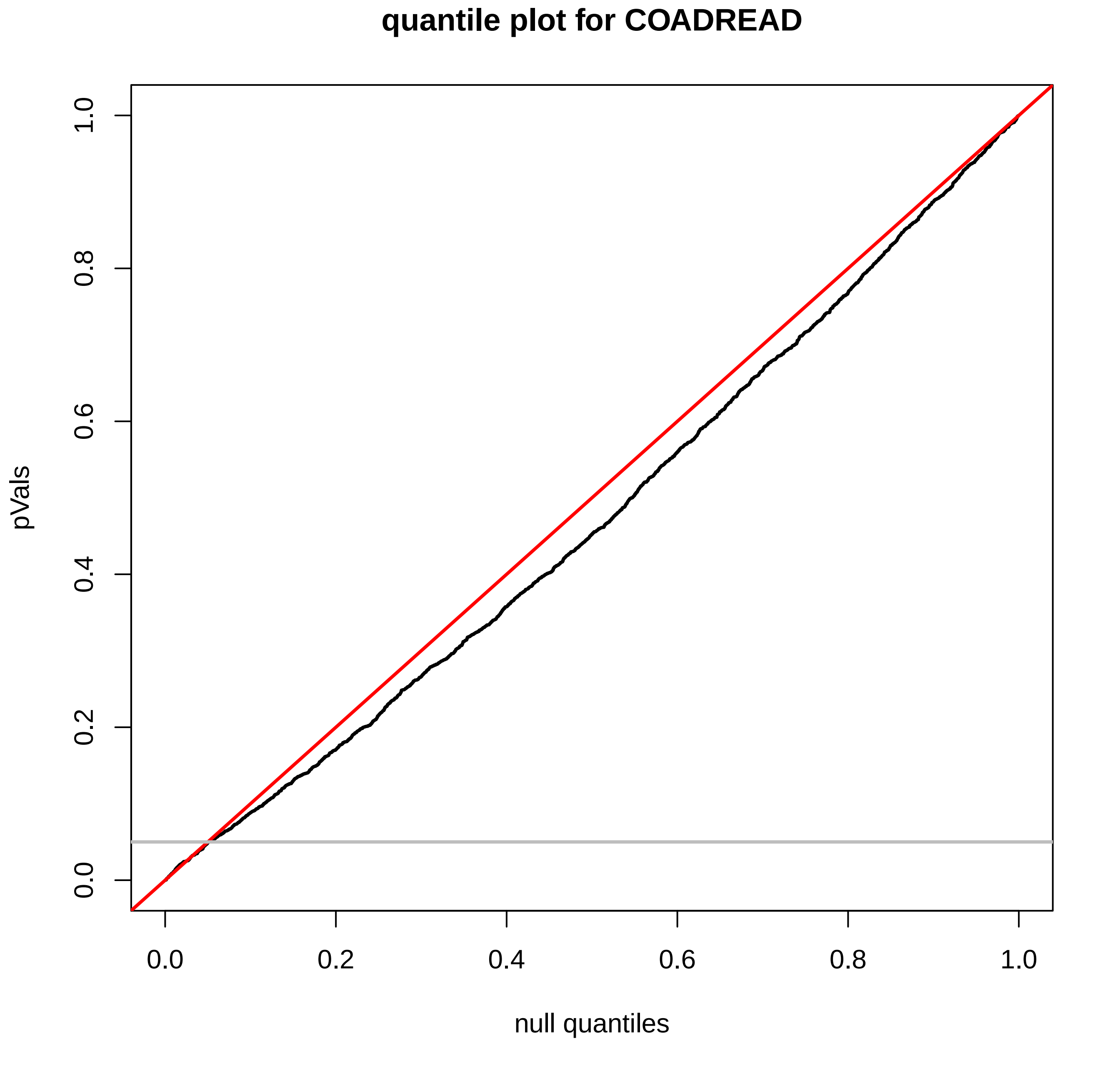
quantile plot for COADREAD
pVals
0.0
0.2
0.4
0.6
0.8
1.0
0.0
0.2
0.4
0.6
0.8
1.0
null quantiles

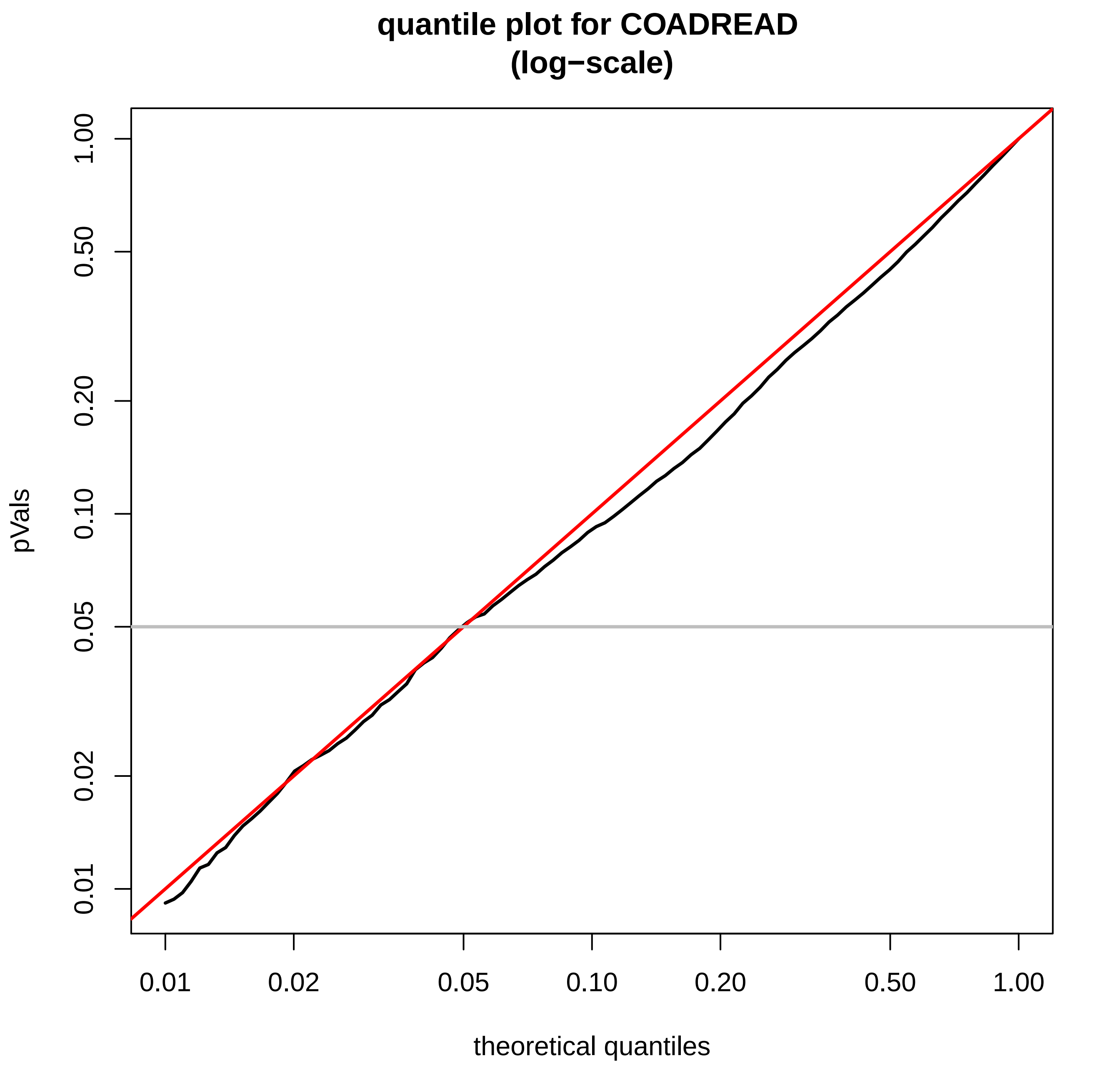
quantile plot for COADREAD
(log−scale)
pVals
1.00
0.50
0.20
0.10
0.05
0.02
0.01
0.01
0.02
0.05
0.10
0.20
0.50
1.00
theoretical quantiles

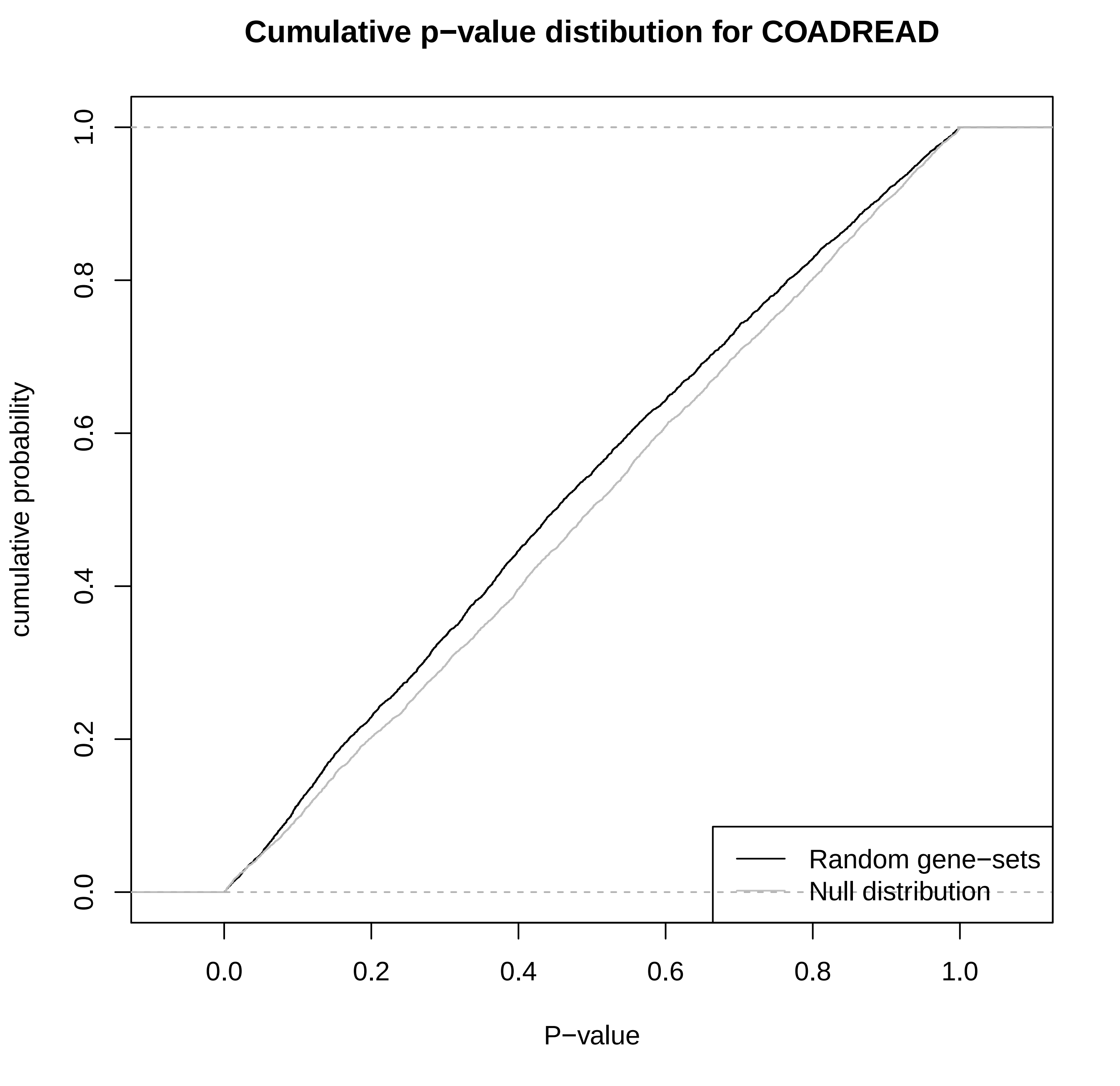

Cumulative p−value distibution for COADREAD
cumulative probability
P−value
0.0
0.2
0.4
0.6
0.8
1.0
Random gene−sets
Null distribution

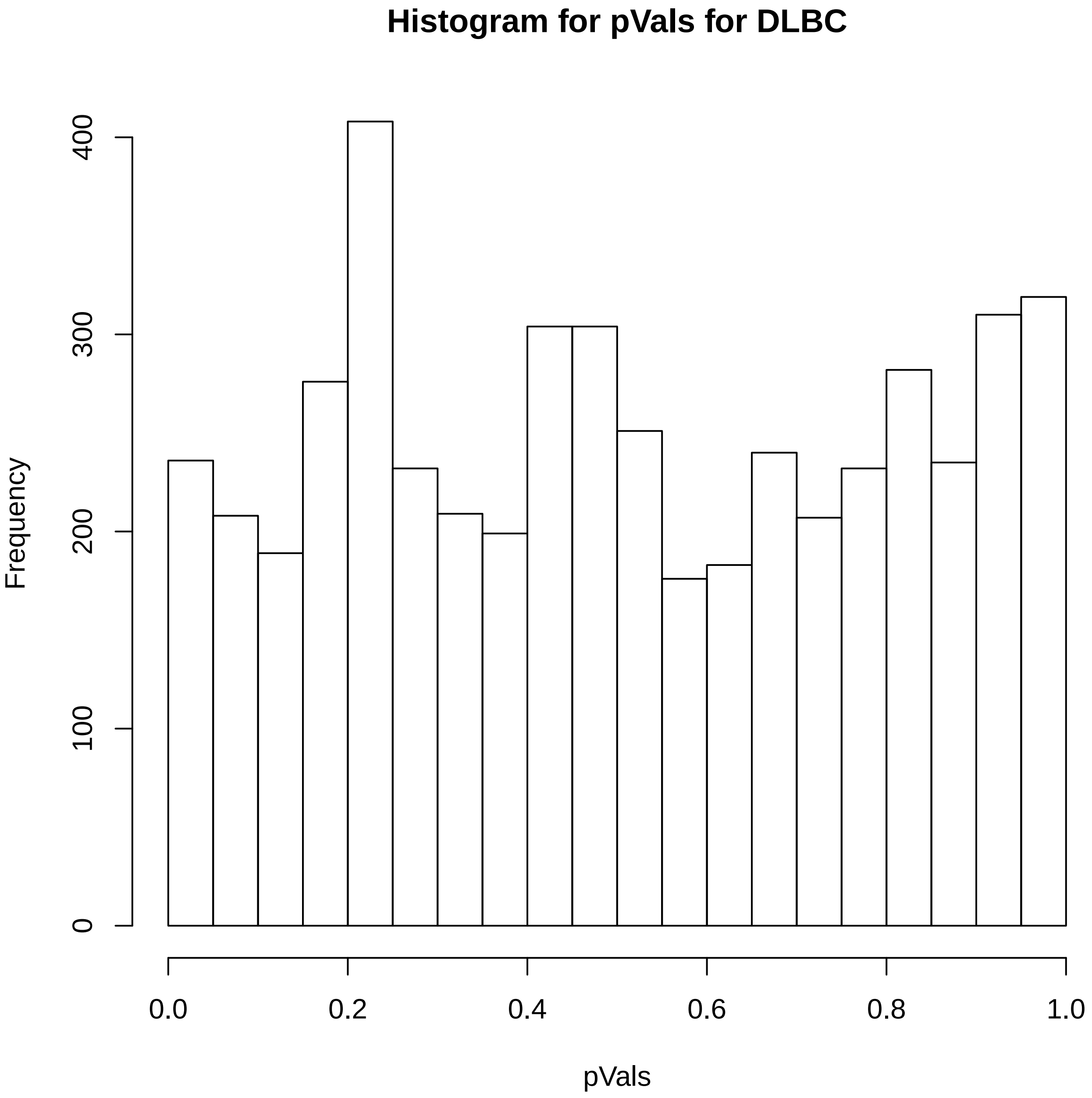
Histogram for pVals for DLBC
Frequency
0
100
200
300
400
0.0
0.2
0.4
0.6
0.8
1.0
pVals

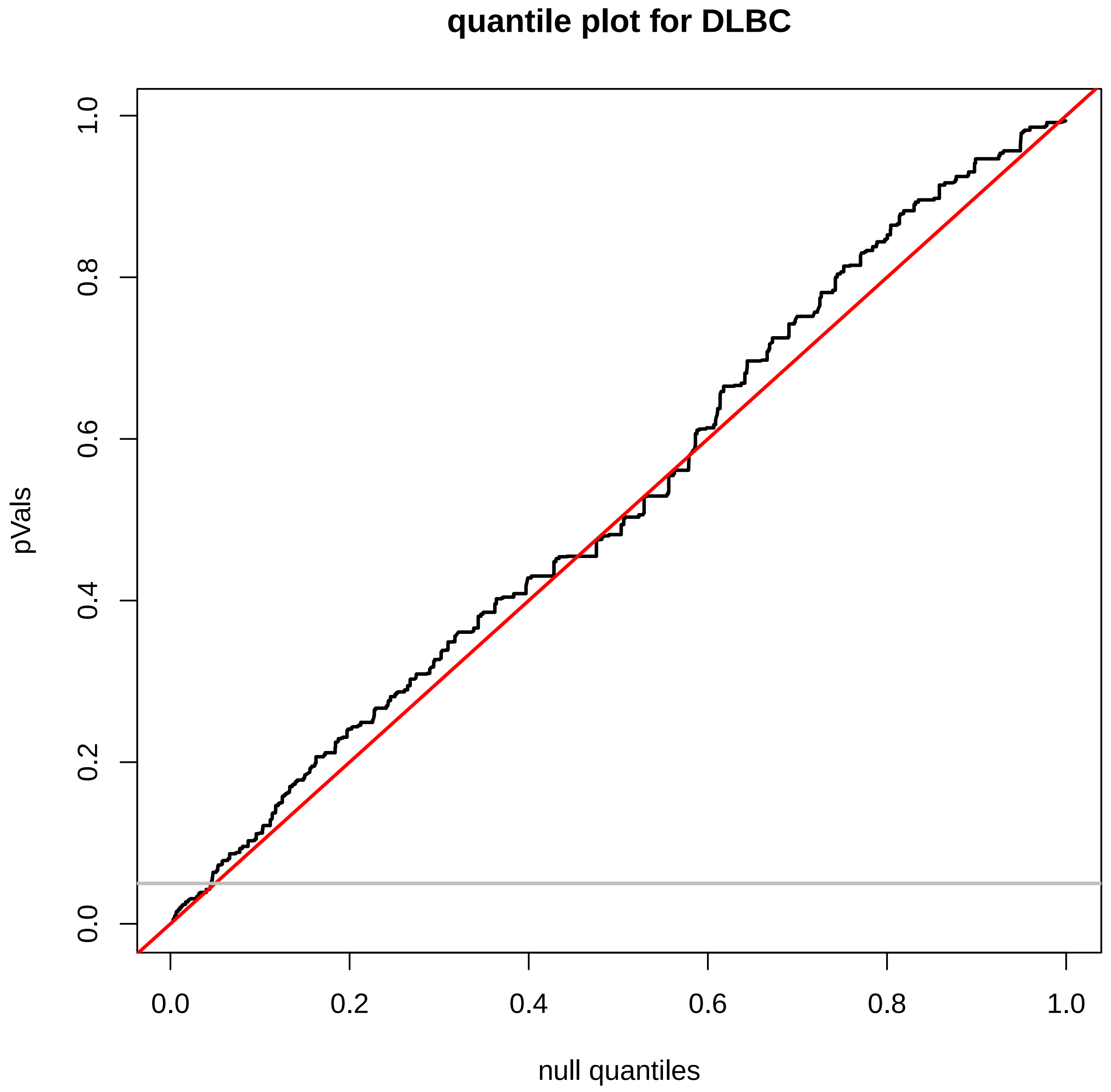

quantile plot for DLBC
pVals
0.0
0.2
0.4
0.6
0.8
1.0
null quantiles
0.0
0.2
0.4
0.6
0.8
1.0

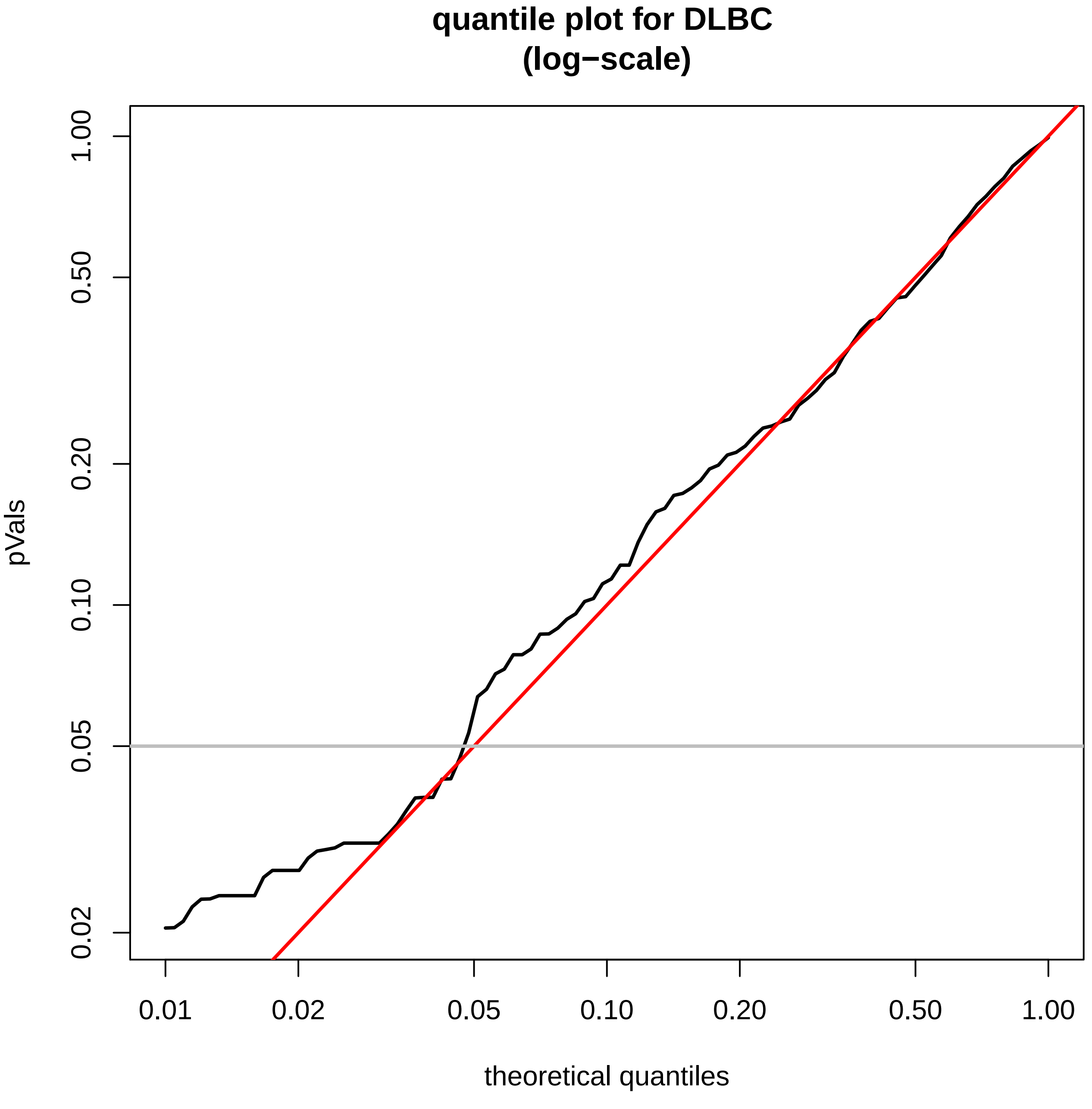
quantile plot for DLBC
(log−scale)
pVals
1.00
0.50
0.20
0.10
0.05
0.02
0.01
0.02
0.05
0.10
0.20
0.50
1.00
theoretical quantiles

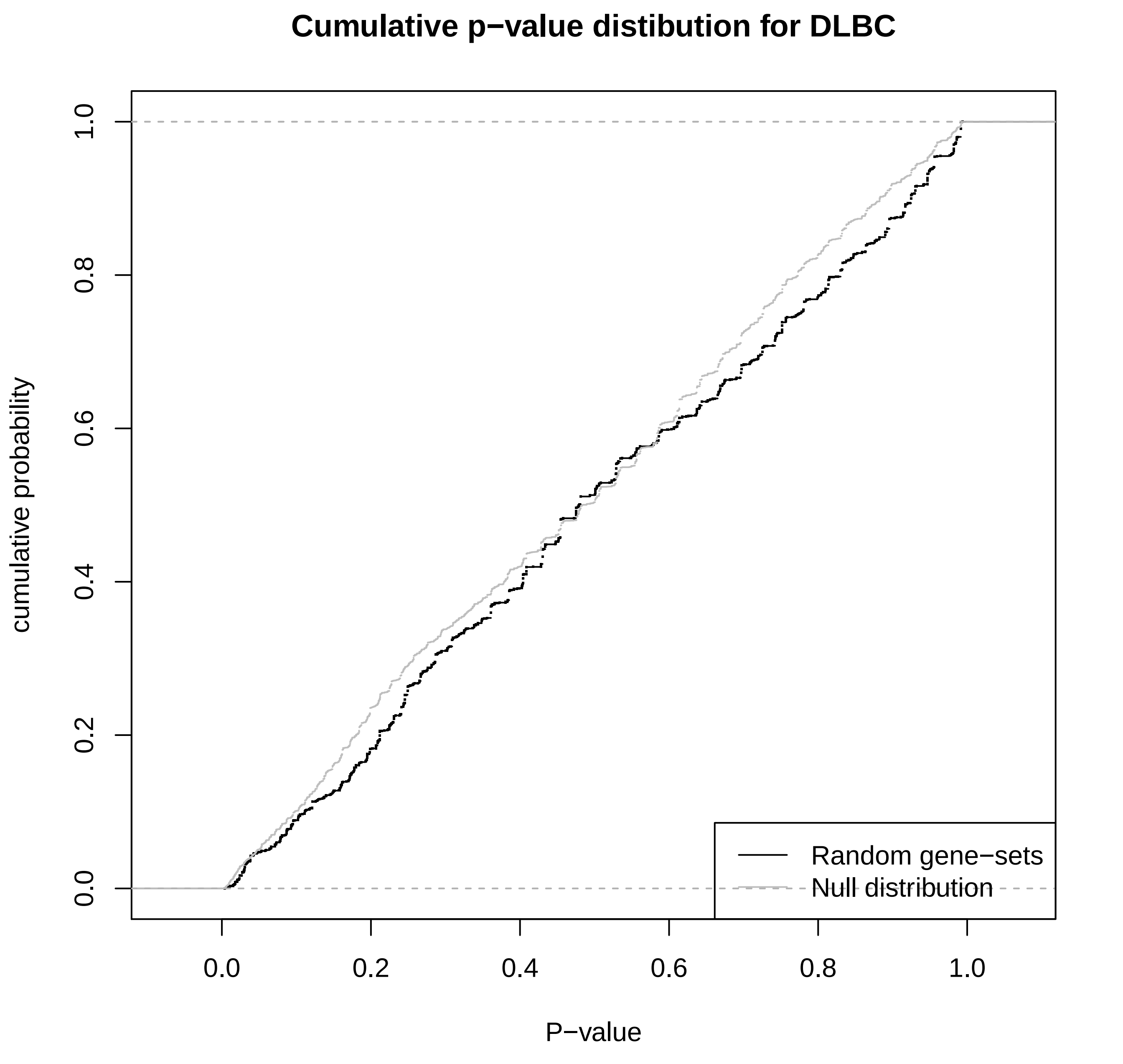
Cumulative p−value distibution for DLBC
cumulative probability
1.0
0.8
0.6
0.4
0.2
0.0
0.0
0.2
0.4
0.6
0.8
1.0
P−value
Random gene−sets
Null distribution

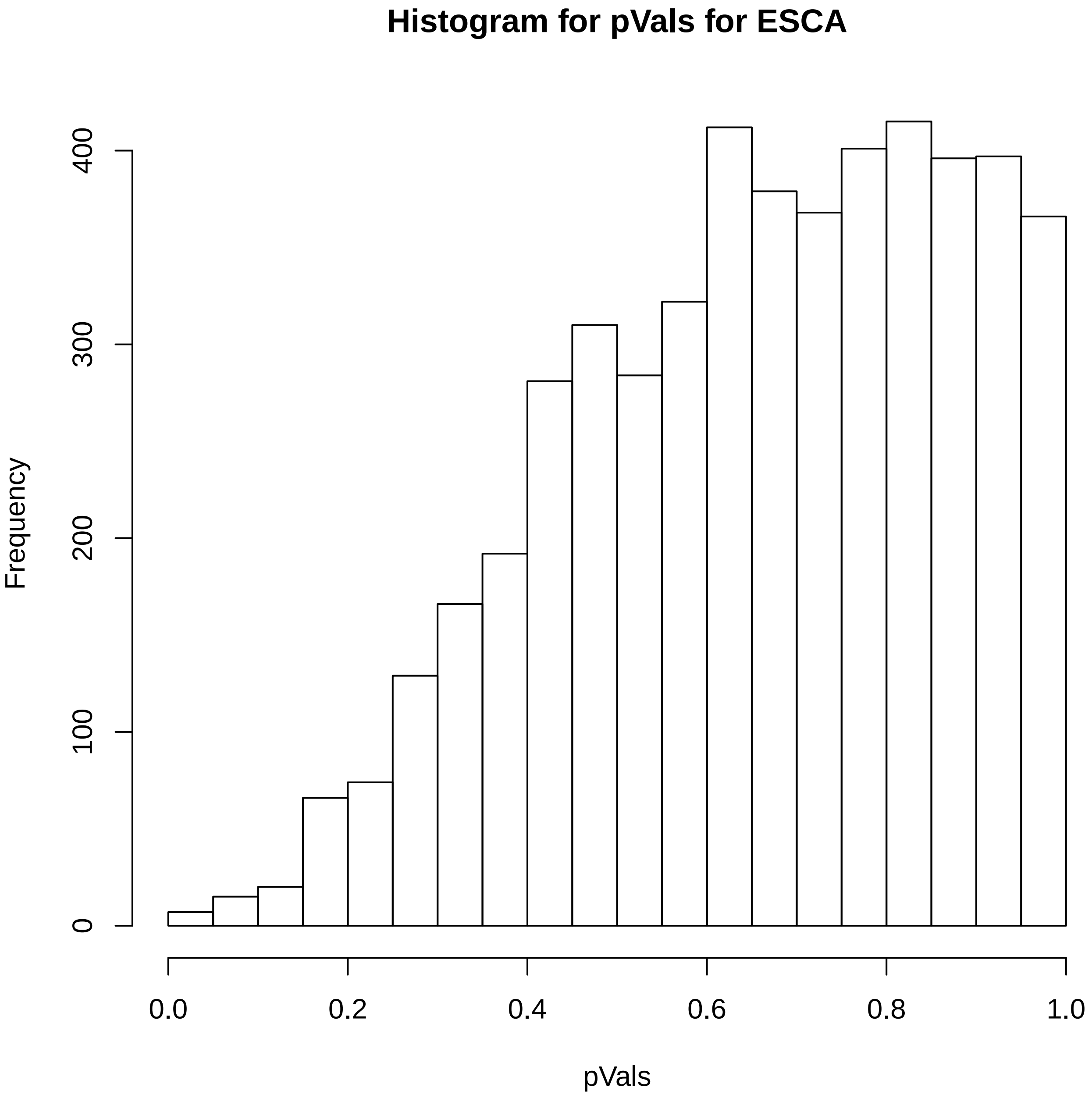

Histogram for pVals for ESCA
Frequency
0
100
200
300
400
0.0
0.2
0.4
0.6
0.8
1.0
pVals

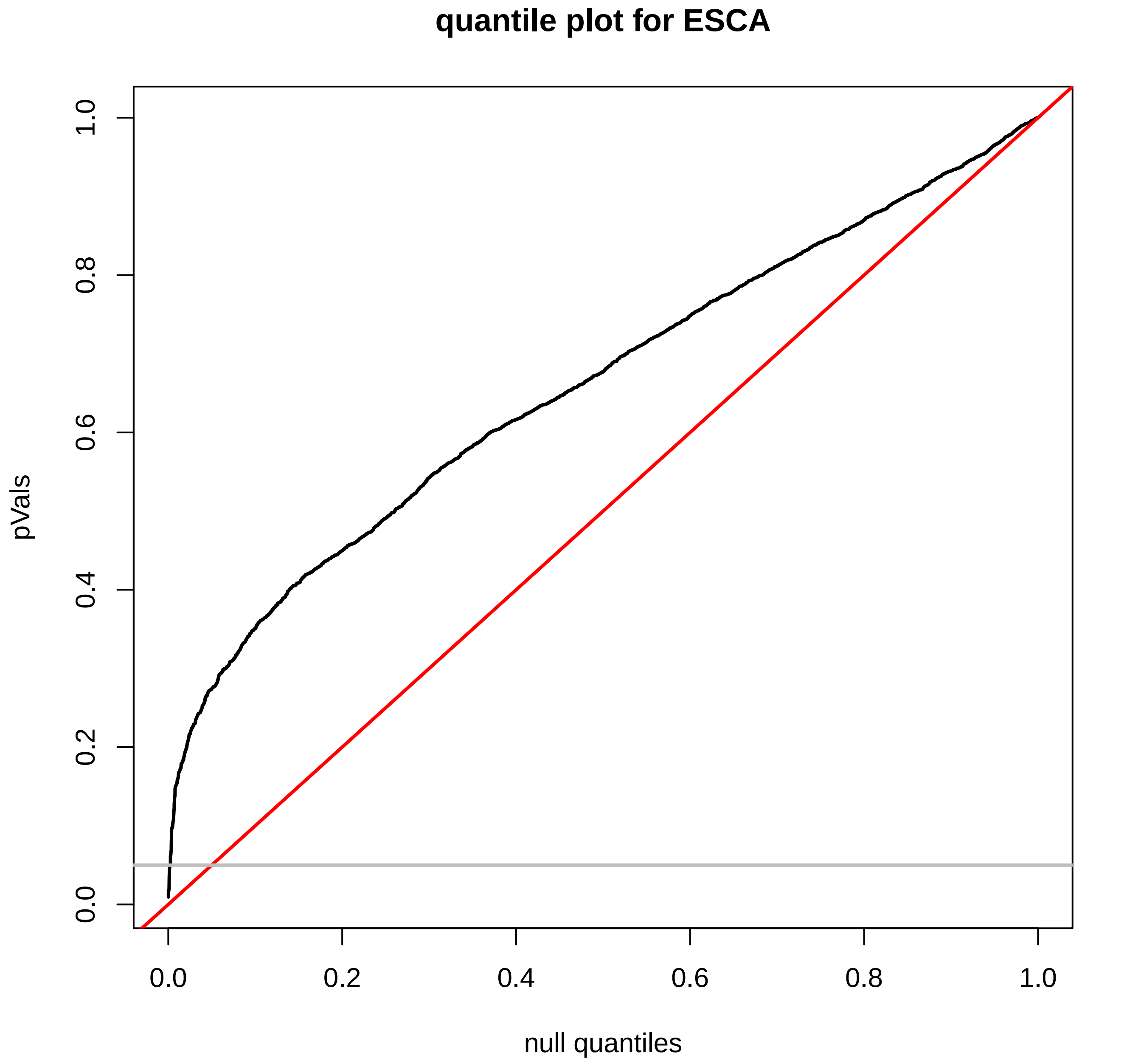
quantile plot for ESCA
pVals
null quantiles
0.0
0.2
0.4
0.6
0.8
1.0

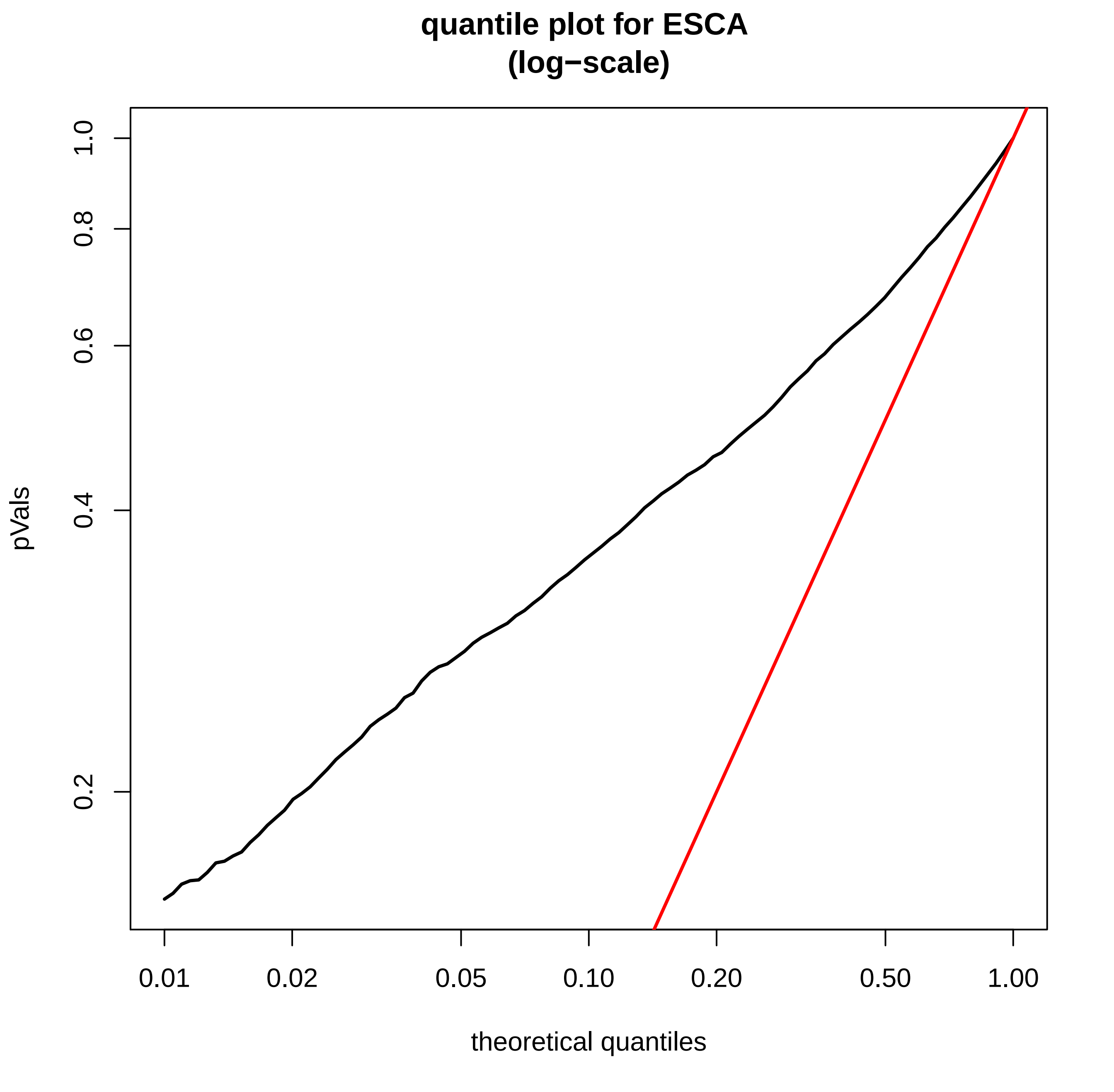
quantile plot for ESCA
(log−scale)
pVals
0.2
0.4
0.6
0.8
1.0
0.01
0.02
0.05
0.10
0.20
0.50
1.00
theoretical quantiles

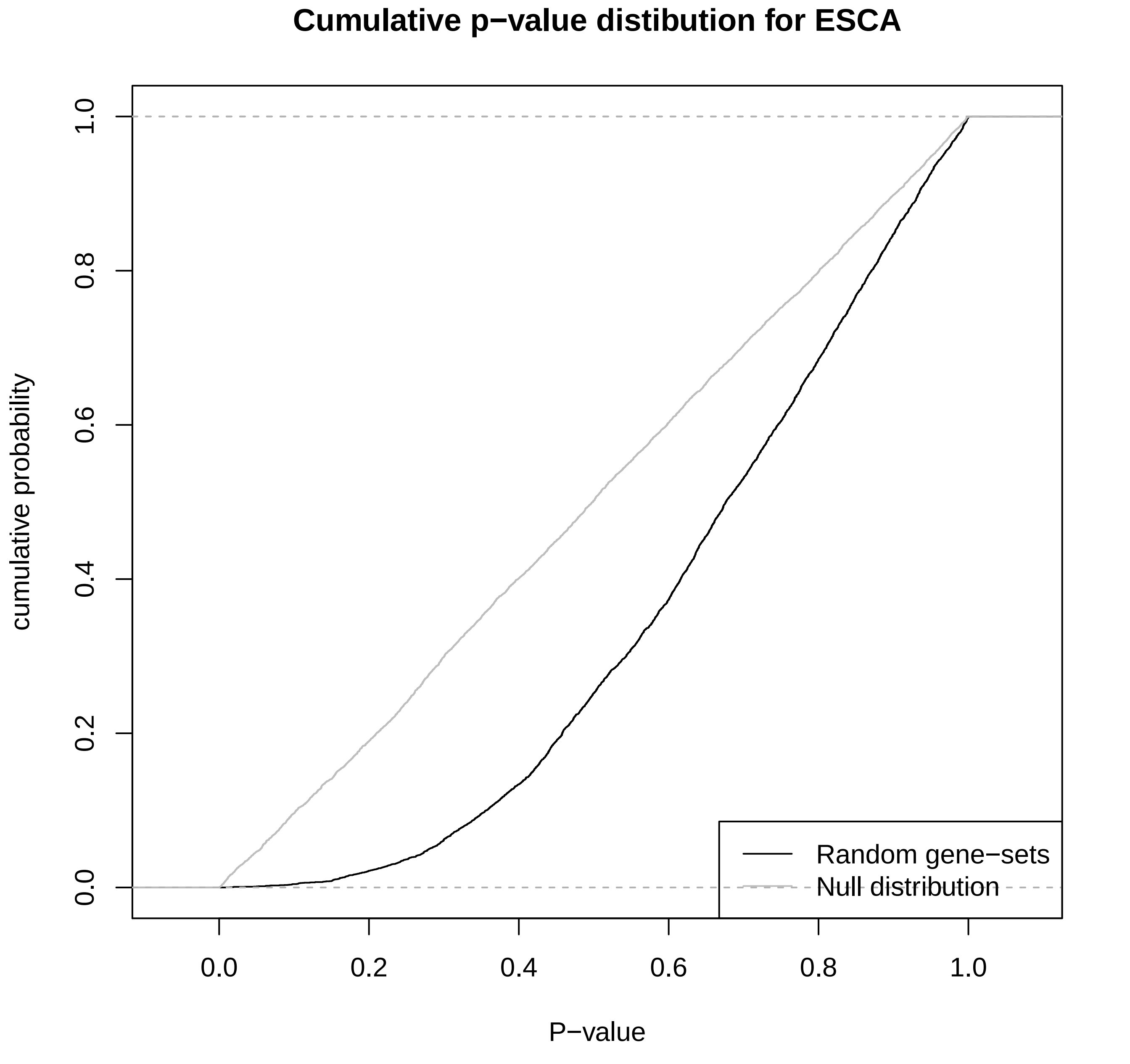
Cumulative p−value distibution for ESCA
cumulative probability
0.0
0.2
0.4
0.6
0.8
1.0
P−value
0.0
0.2
0.4
0.6
0.8
1.0
Random gene−sets
Null distribution

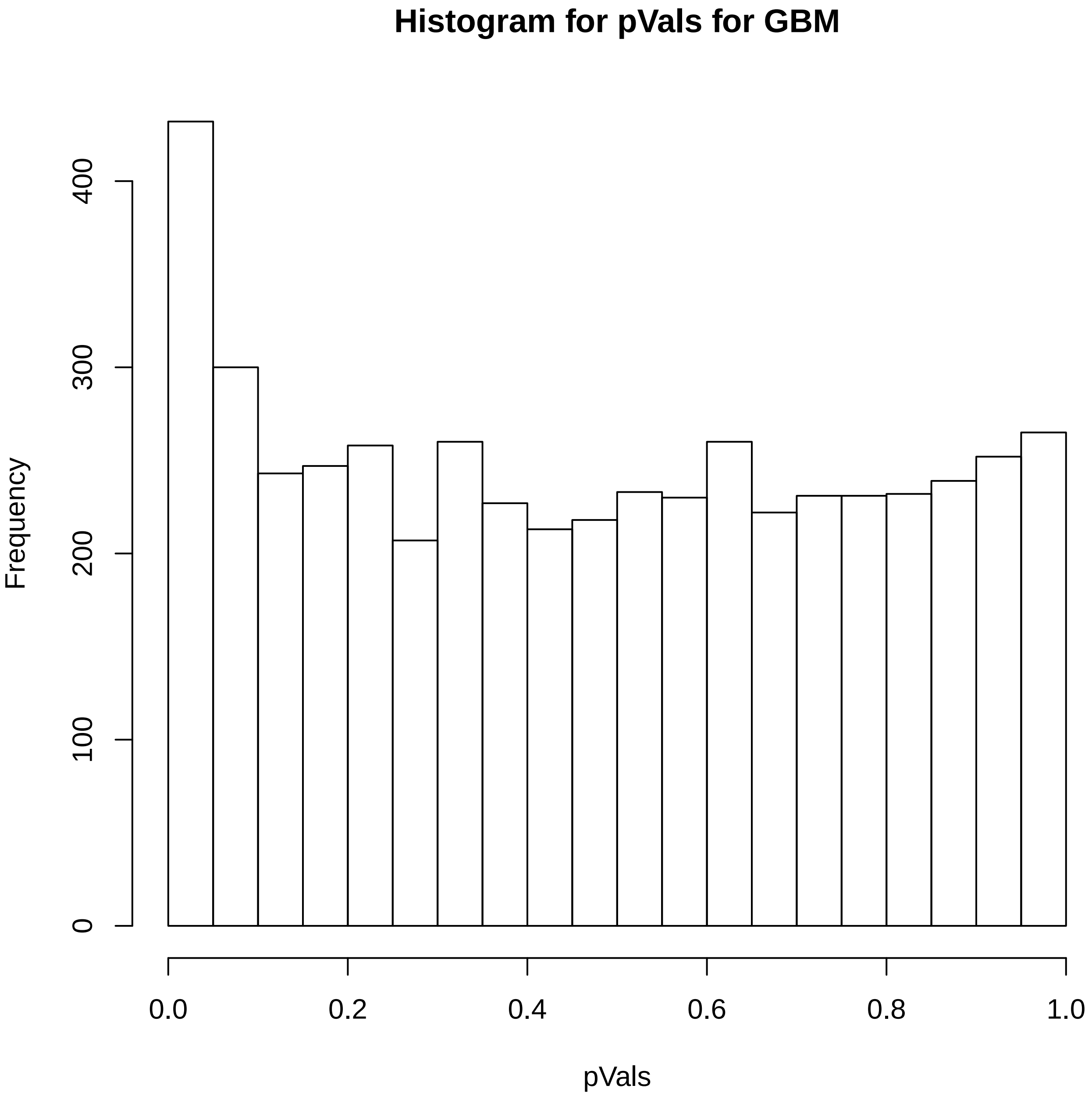

Histogram for pVals for GBM
Frequency
0
100
200
300
400
0.0
0.2
0.4
0.6
0.8
1.0
pVals

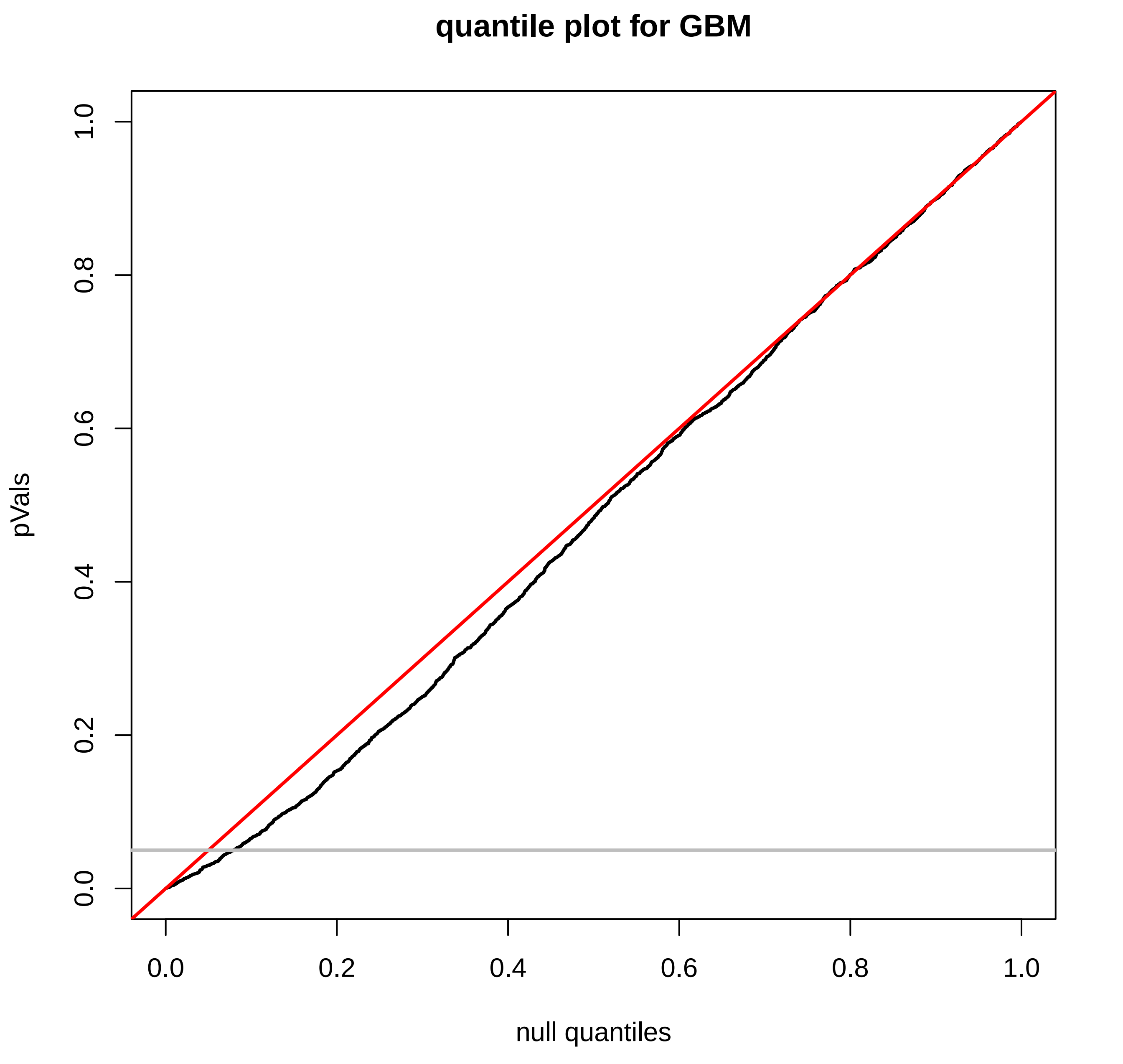
quantile plot for GBM
pVals
null quantiles
0.0
0.2
0.4
0.6
0.8
1.0
0.0
0.2
0.4
0.6
0.8
1.0

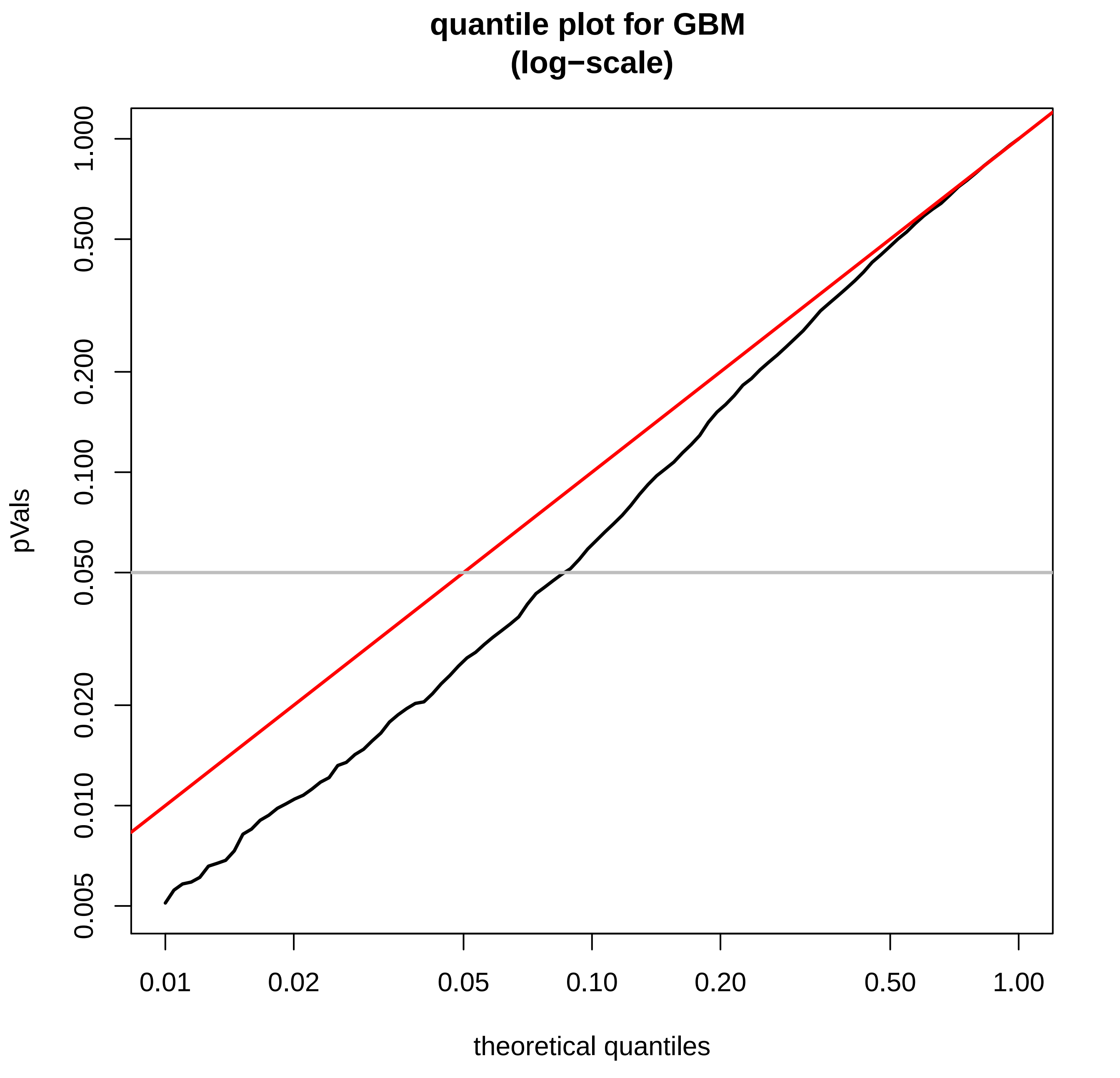
quantile plot for GBM
(log−scale)
pVals
1.000
0.500
0.200
0.100
0.050
0.020
0.010
0.005
0.01
0.02
0.05
0.10
0.20
0.50
1.00
theoretical quantiles

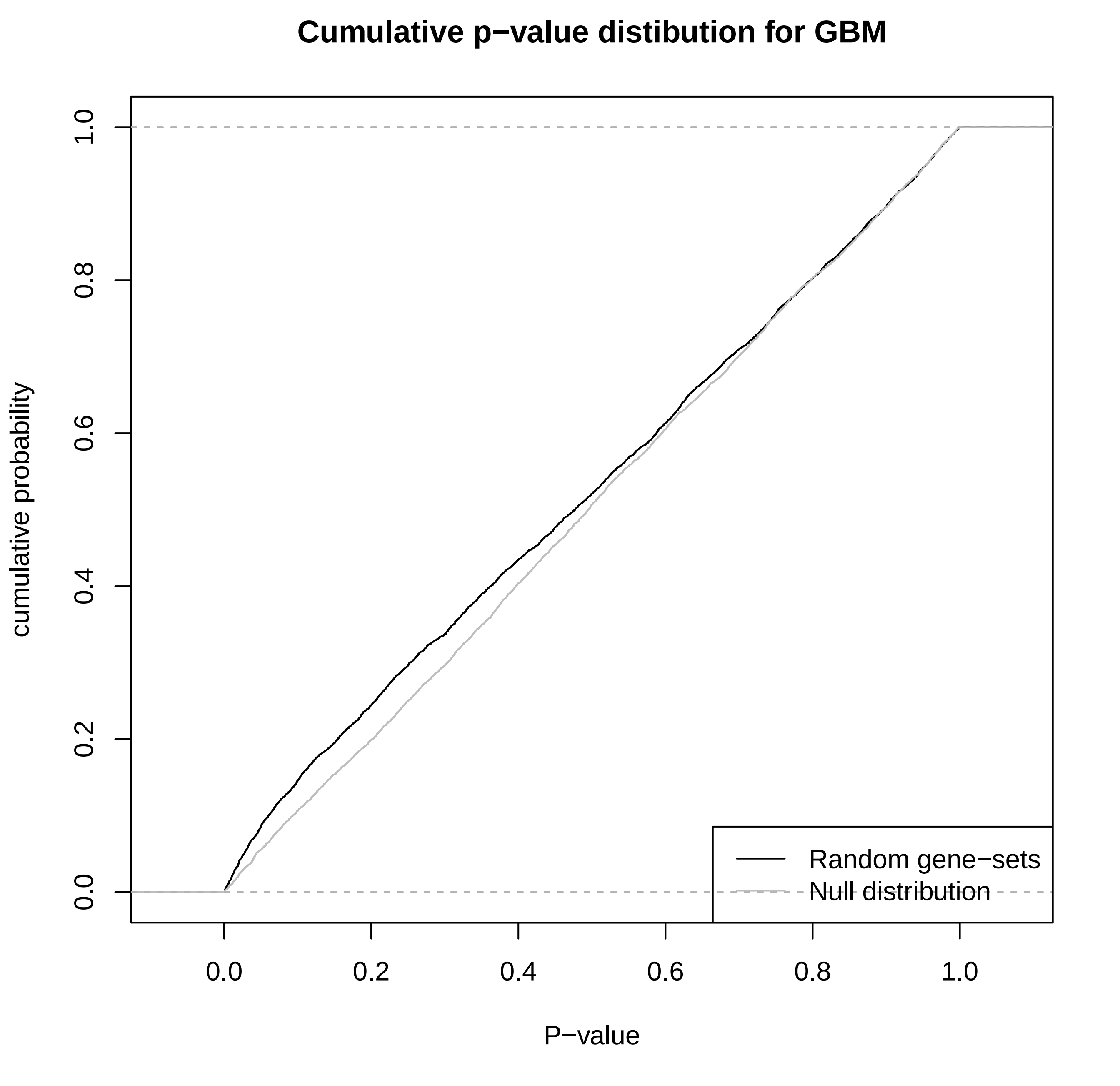
Cumulative p−value distibution for GBM
cumulative probability
P−value
0.0
0.2
0.4
0.6
0.8
1.0
0.0
0.2
0.4
0.6
0.8
1.0
Random gene−sets
Null distribution

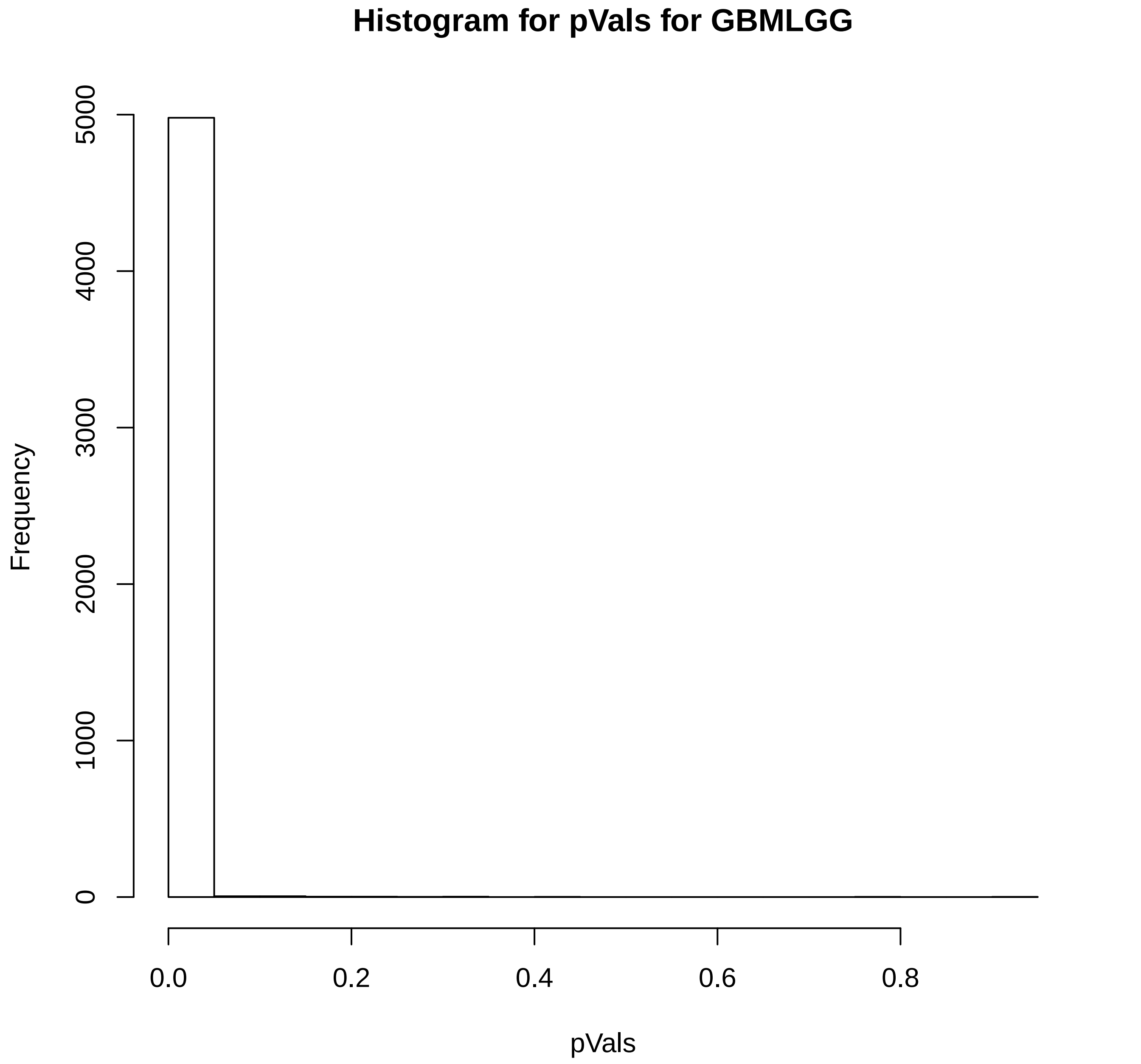
Histogram for pVals for GBMLGG
Frequency
0
1000
2000
3000
4000
5000
0.0
0.2
0.4
0.6
0.8
pVals

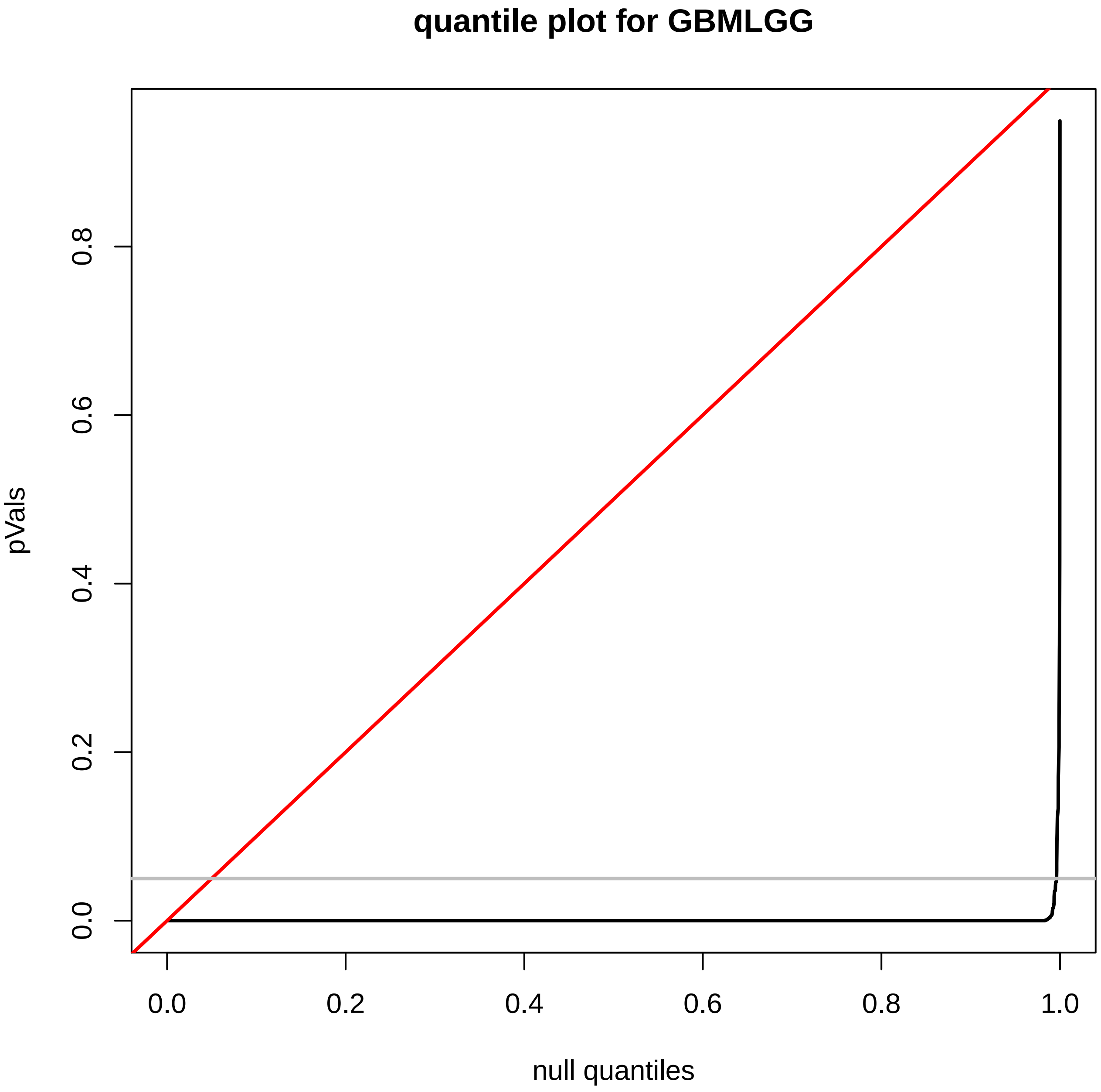
quantile plot for GBMLGG
pVals
null quantiles
0.0
0.2
0.4
0.6
0.8
1.0
0.0
0.2
0.4
0.6
0.8

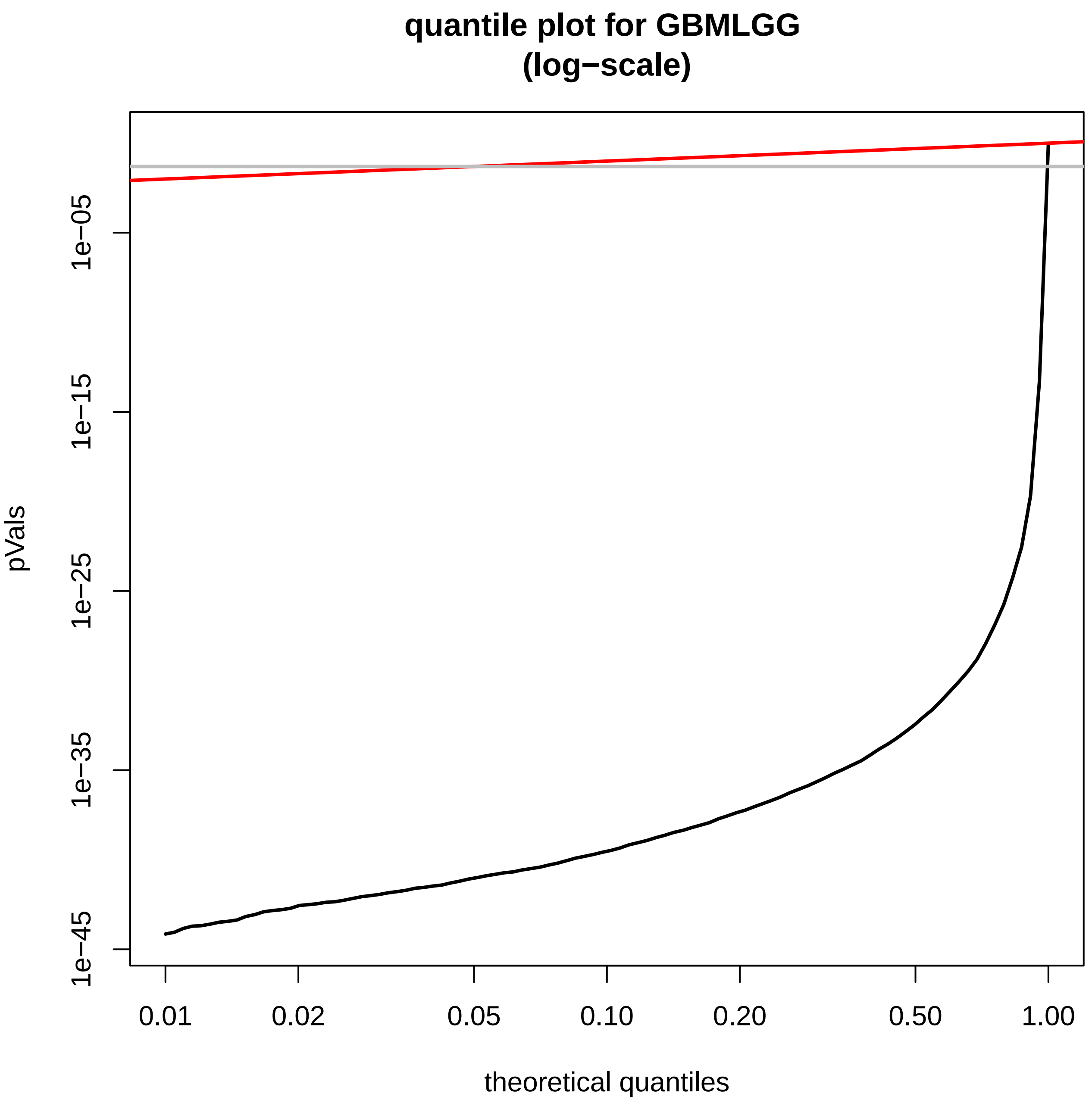

quantile plot for GBMLGG
(log−scale)
pVals
1e−05
1e−15
1e−25
1e−35
1e−45
0.01
0.02
0.05
0.10
0.20
0.50
1.00
theoretical quantiles

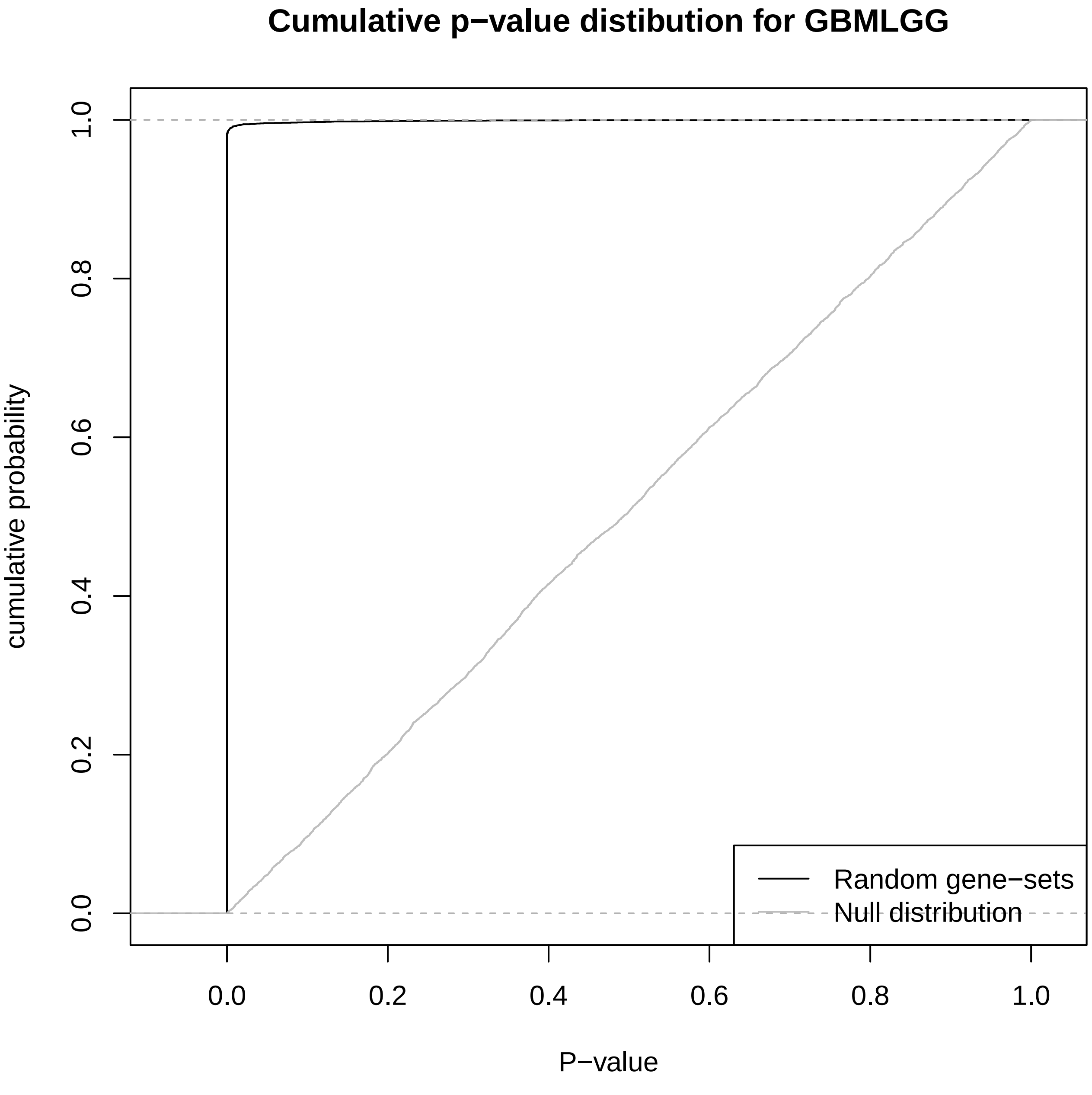
Cumulative p−value distibution for GBMLGG
cumulative probability
P−value
0.0
0.2
0.4
0.6
0.8
1.0
0.0
0.2
0.4
0.6
0.8
1.0
Random gene−sets
Null distribution

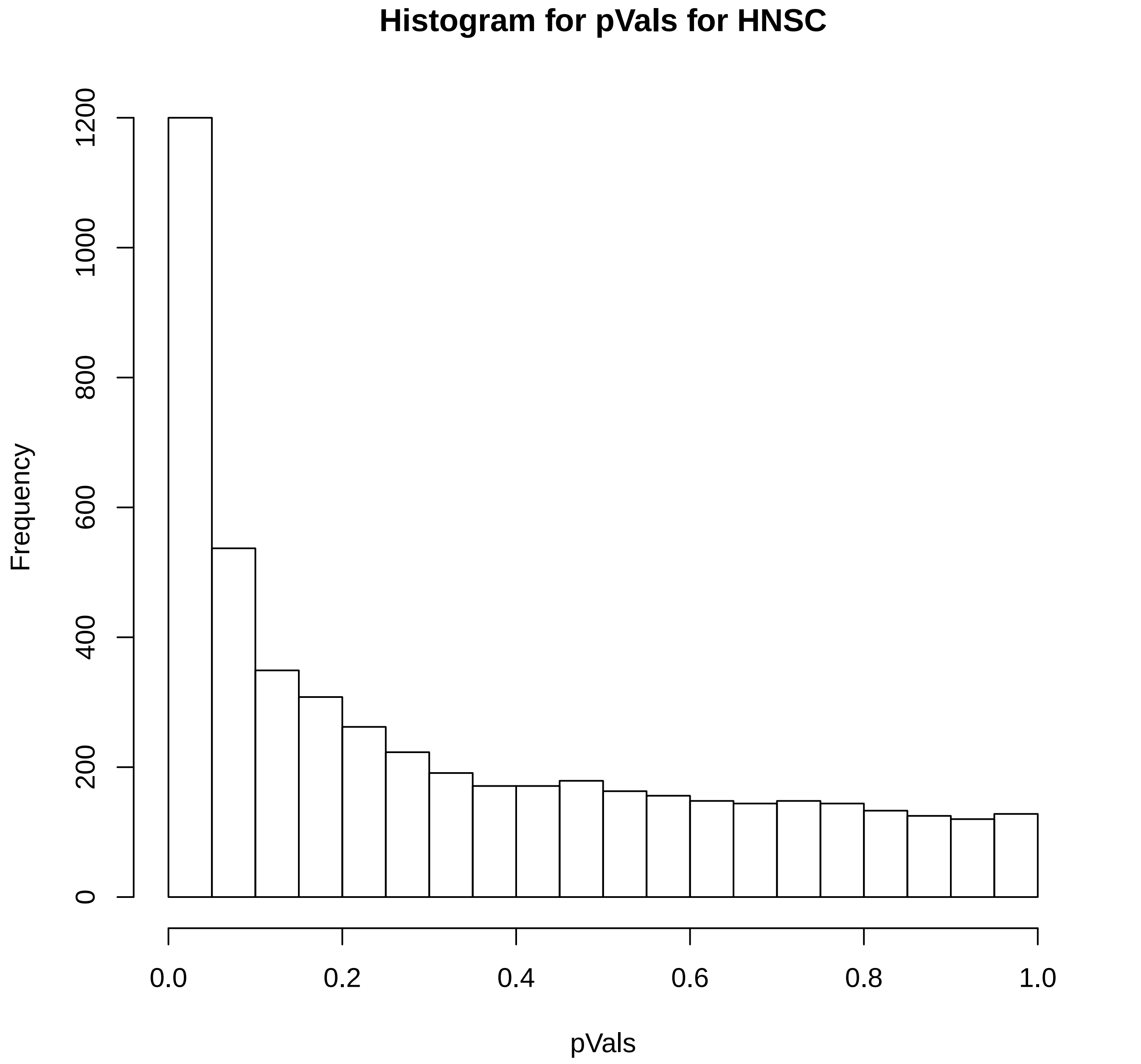
Histogram for pVals for HNSC
Frequency
0
200
400
600
800
1000
1200
0.0
0.2
0.4
0.6
0.8
1.0
pVals

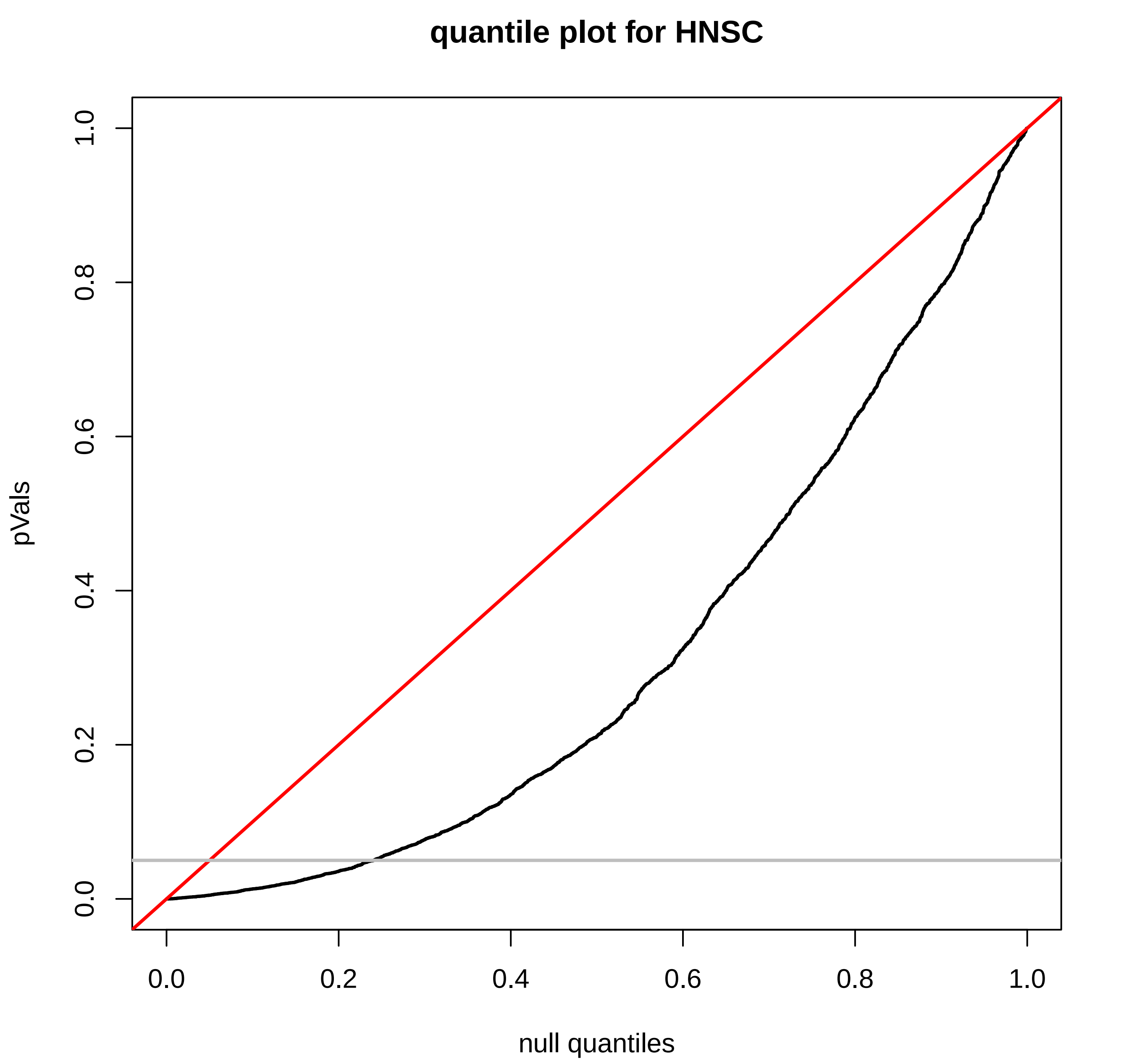
quantile plot for HNSC
pVals
1.0
0.8
0.6
0.4
0.2
0.0
0.0
0.2
0.4
0.6
0.8
1.0
null quantiles

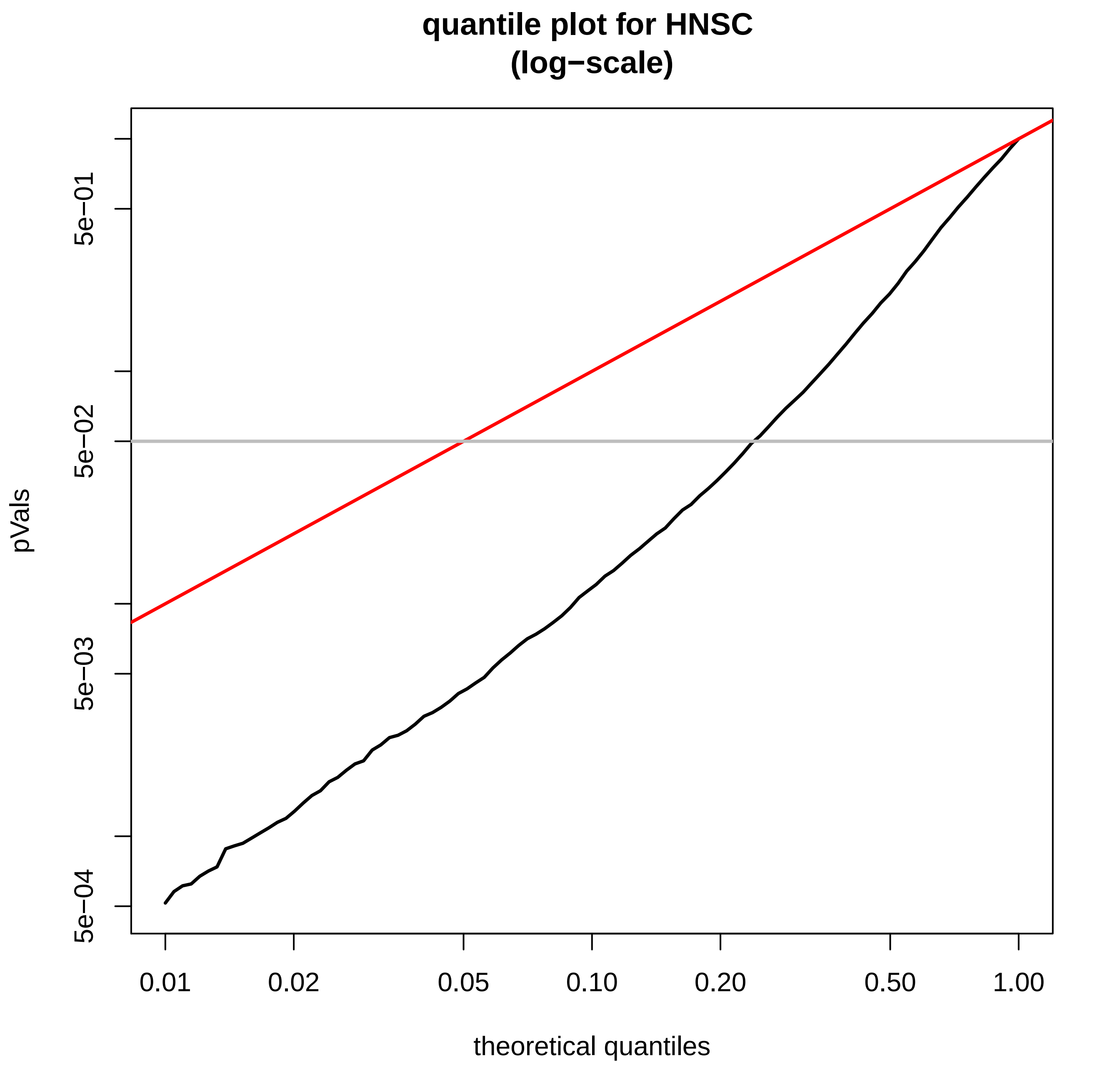
quantile plot for HNSC
(log−scale)
pVals
5e−01
5e−02
5e−03
5e−04
0.01
0.02
0.05
0.10
0.20
0.50
1.00
theoretical quantiles

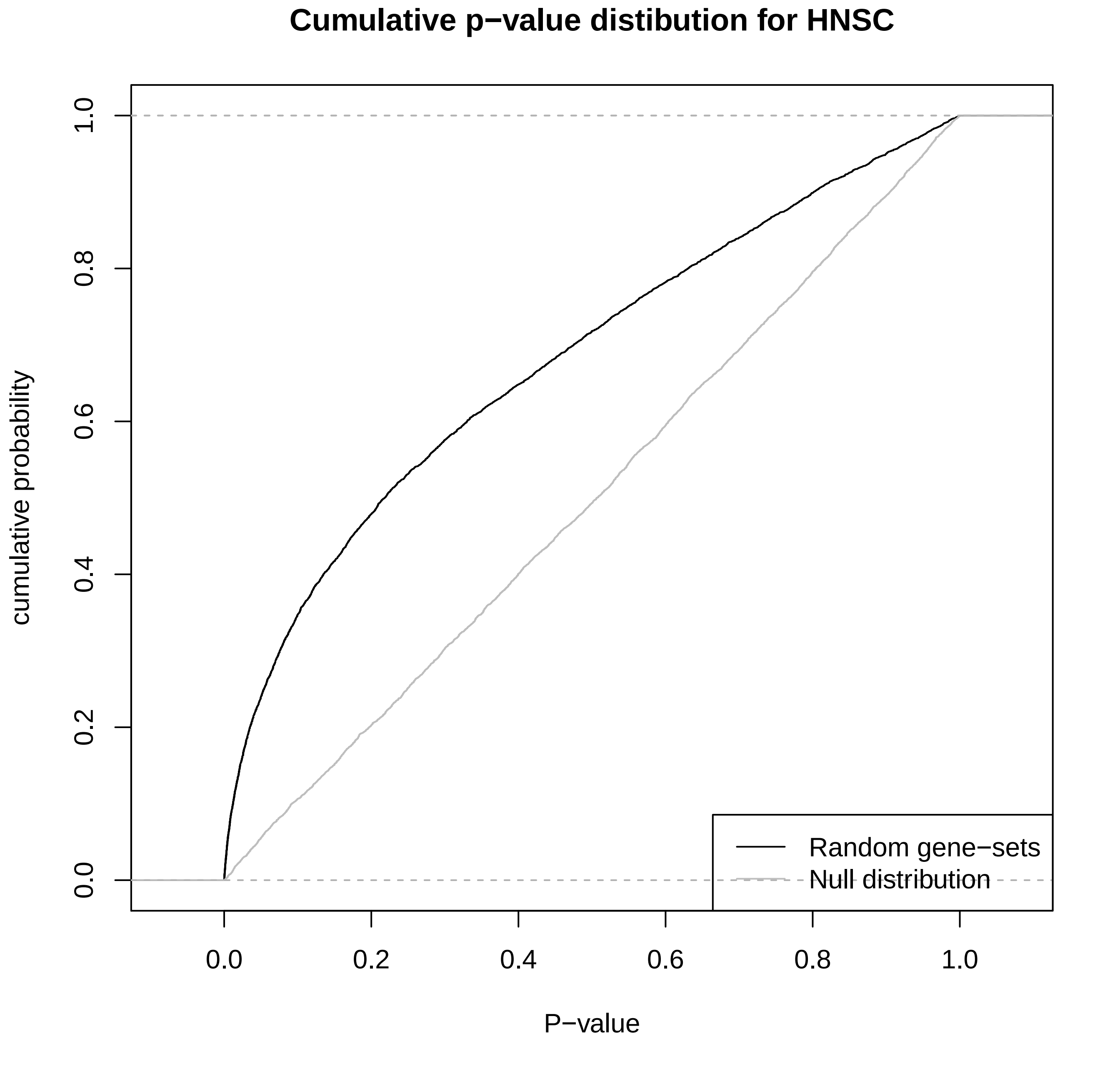
Cumulative p−value distibution for HNSC
cumulative probability
P−value
0.0
0.2
0.4
0.6
0.8
1.0
0.0
0.2
0.4
0.6
0.8
1.0
Random gene−sets
Null distribution

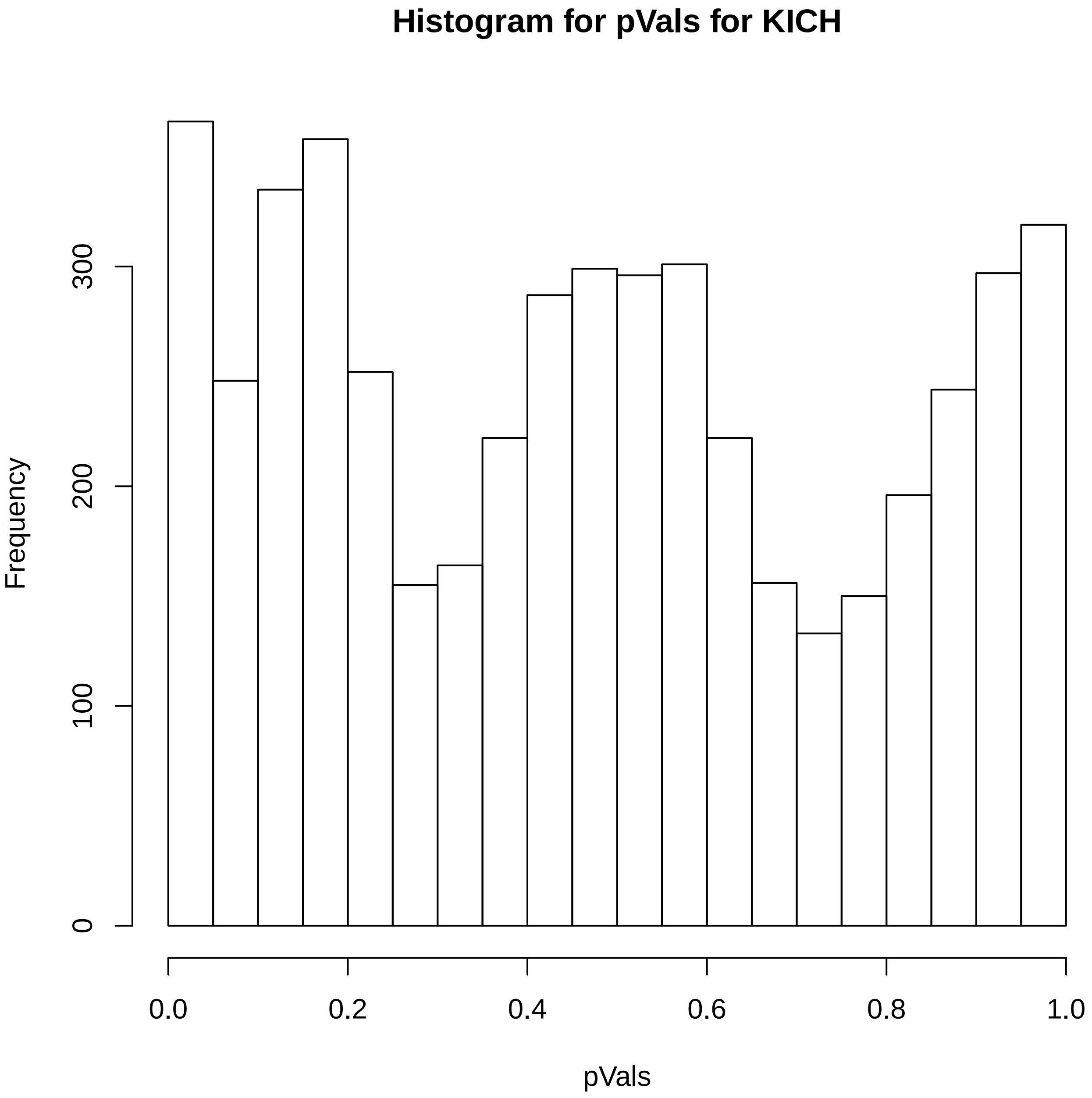
Histogram for pVals for KICH
Frequency
0
100
200
300
0.0
0.2
0.4
0.6
0.8
1.0
pVals

quantile plot for KICH

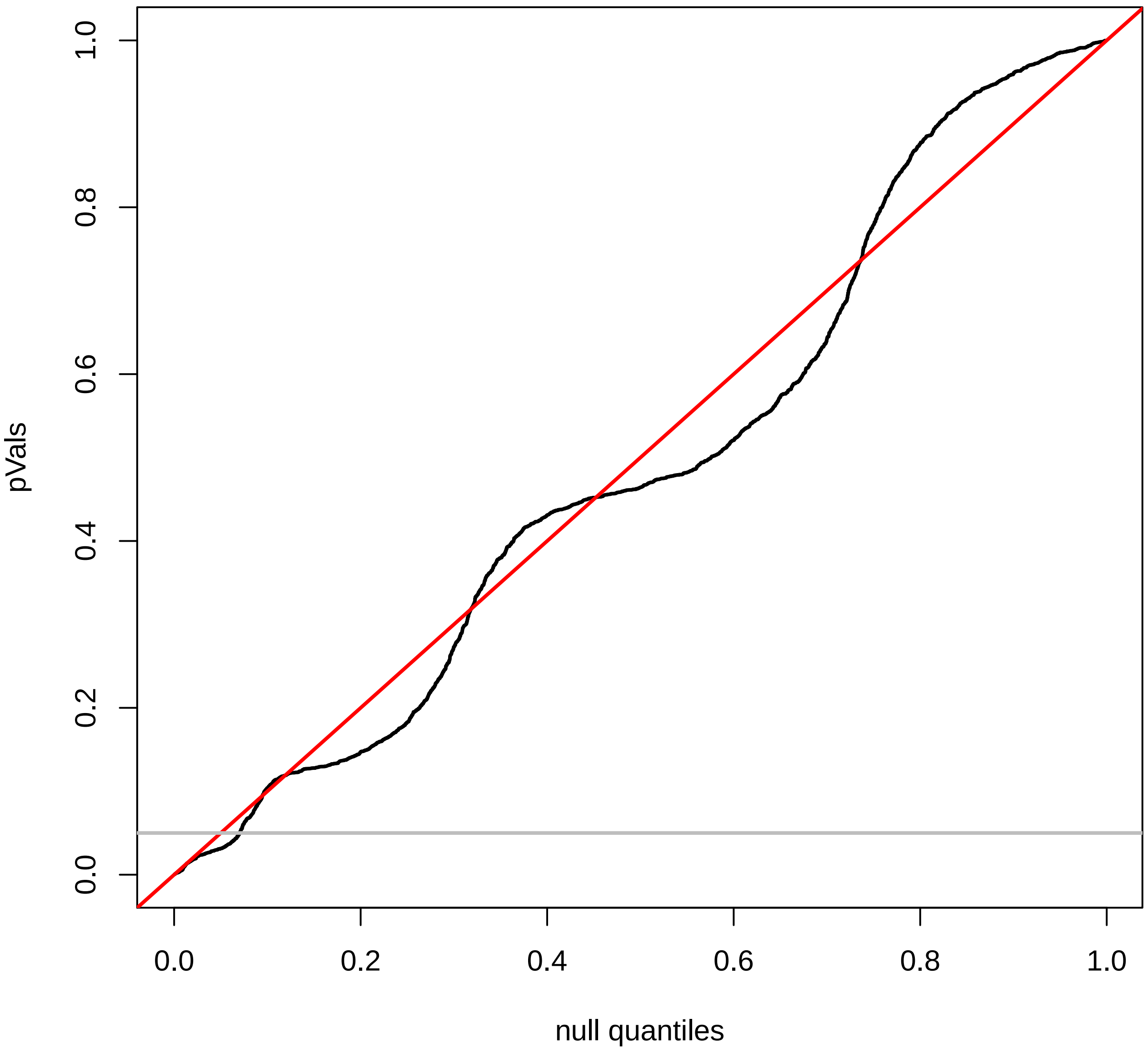

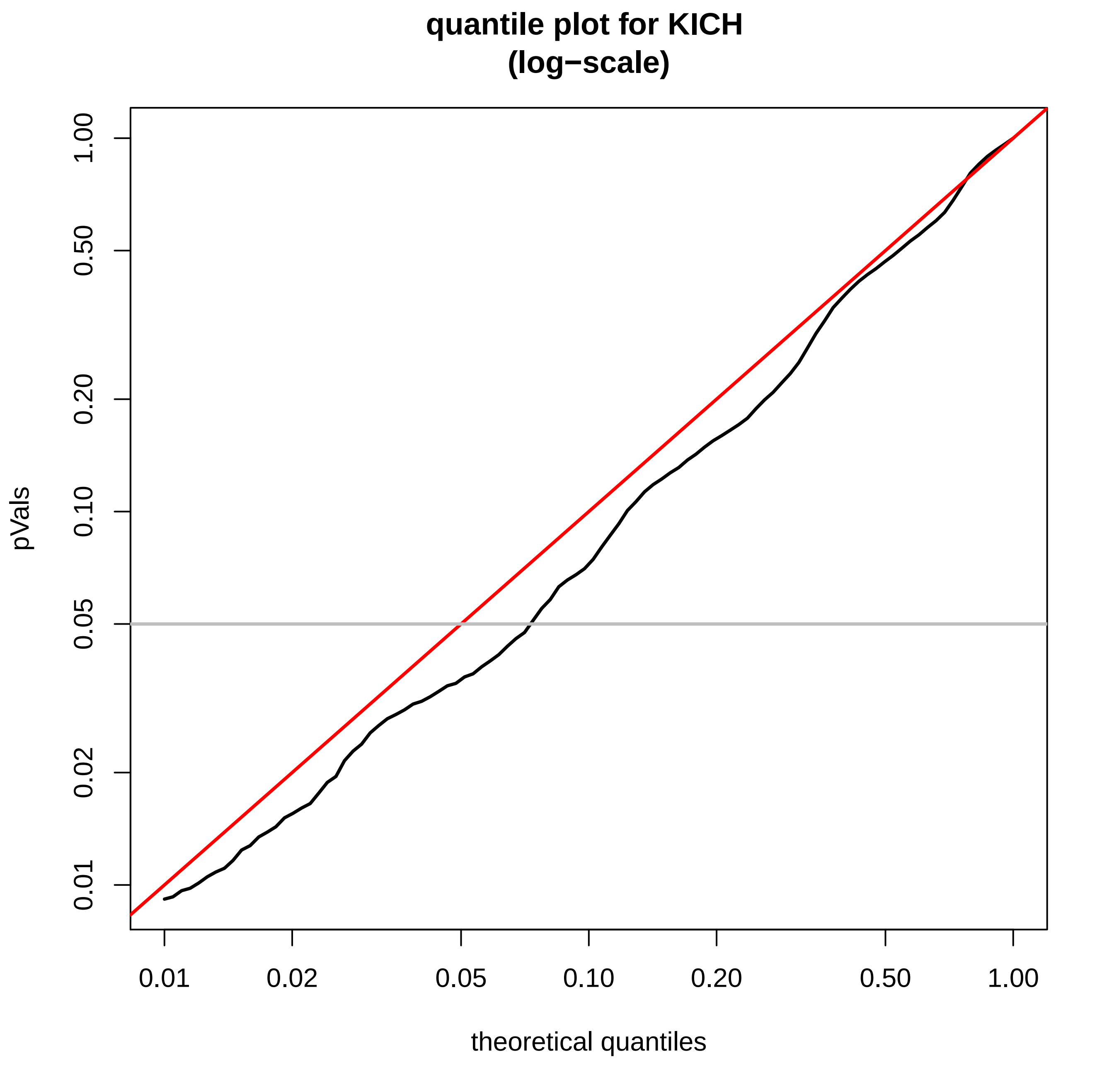

quantile plot for KICH
(log−scale)
pVals
1.00
0.50
0.20
0.10
0.05
0.02
0.01
0.01
0.02
0.05
0.10
0.20
0.50
1.00
theoretical quantiles

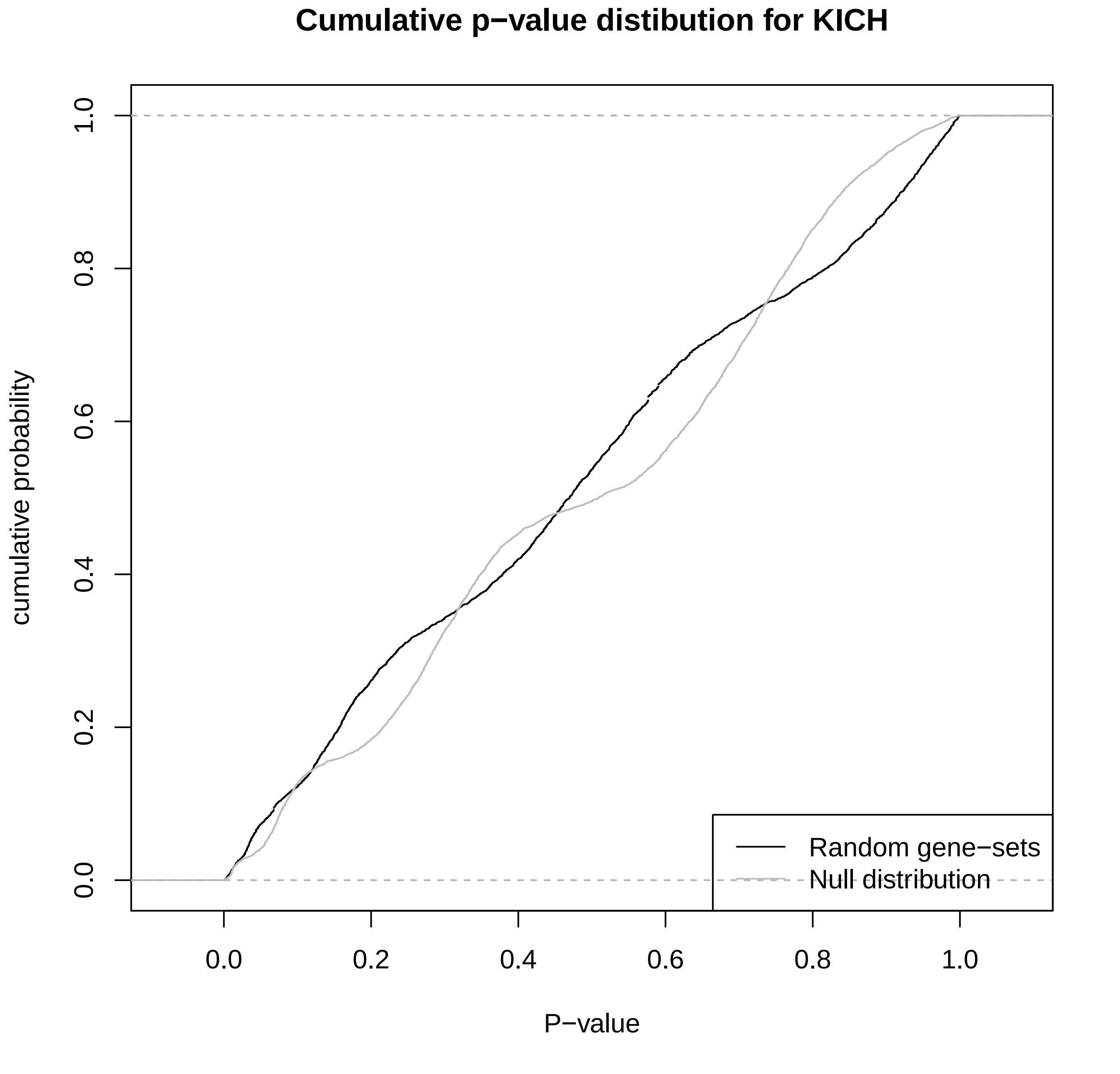
Cumulative p−value distibution for KICH
cumulative probability
P−value
0.0
0.2
0.4
0.6
0.8
1.0
Random gene−sets
Null distribution

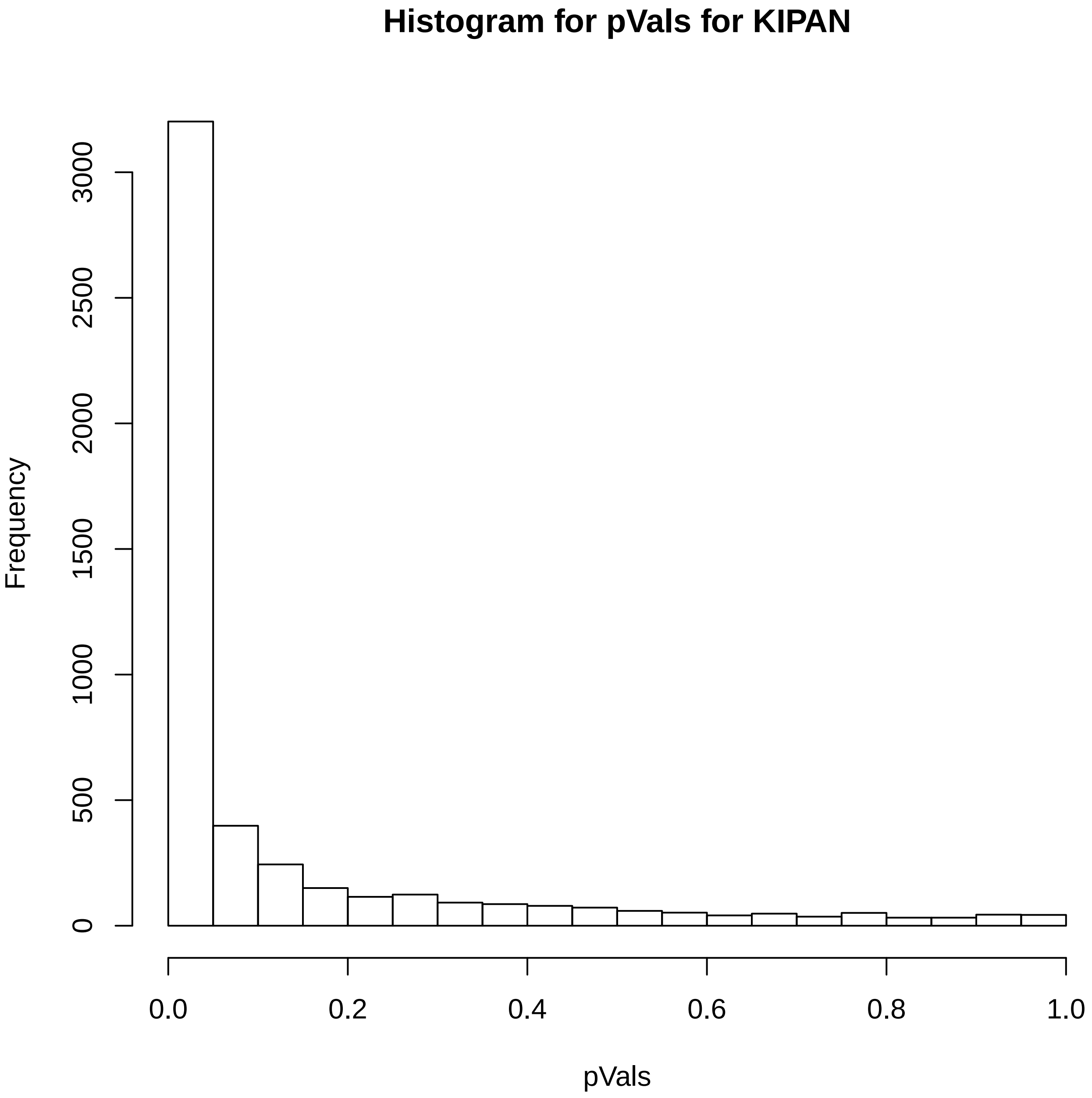

Histogram for pVals for KIPAN
Frequency
0
500
1000
1500
2000
2500
3000
0.0
0.2
0.4
0.6
0.8
1.0
pVals

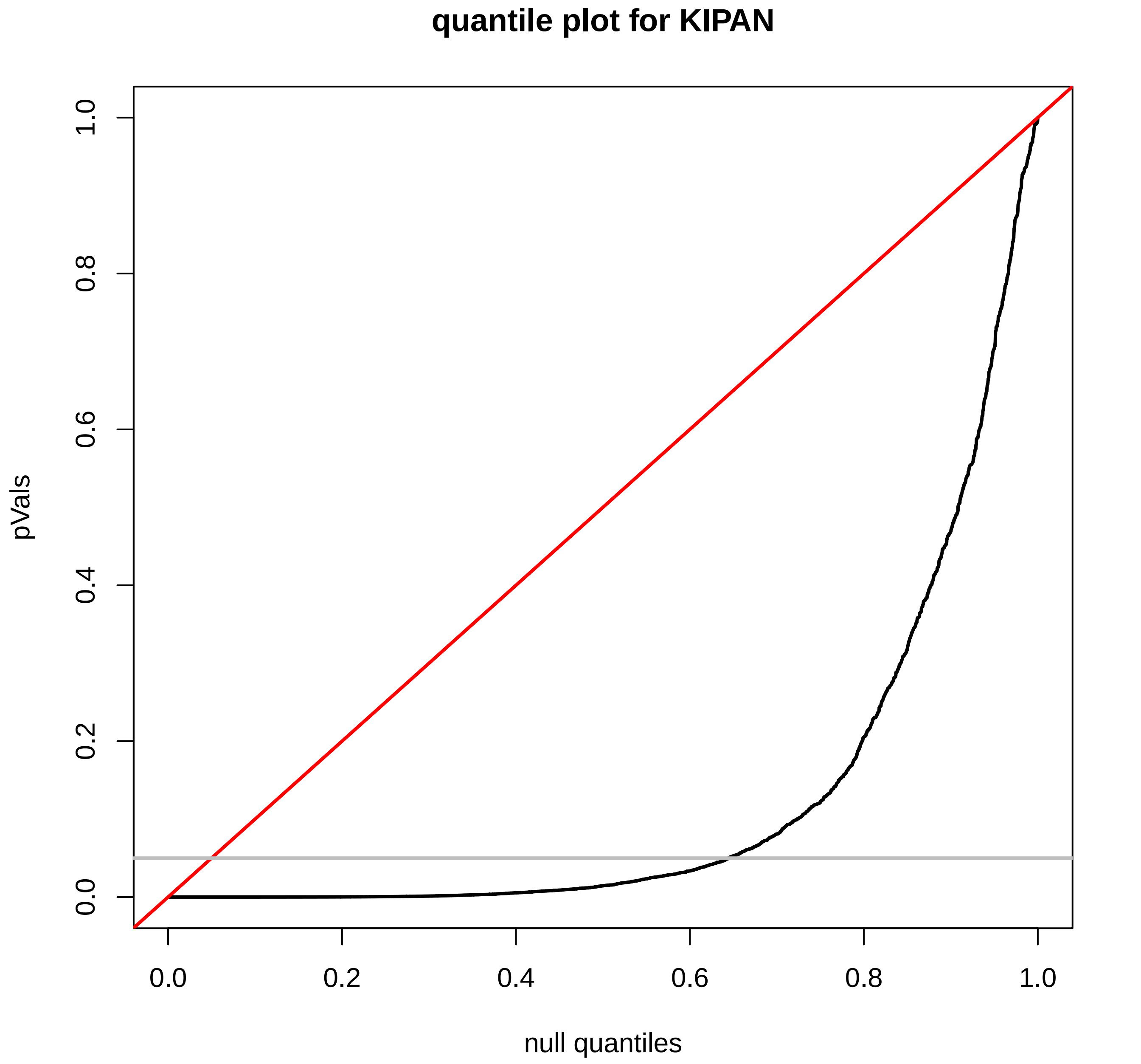
quantile plot for KIPAN
pVals
null quantiles
0.0
0.2
0.4
0.6
0.8
1.0

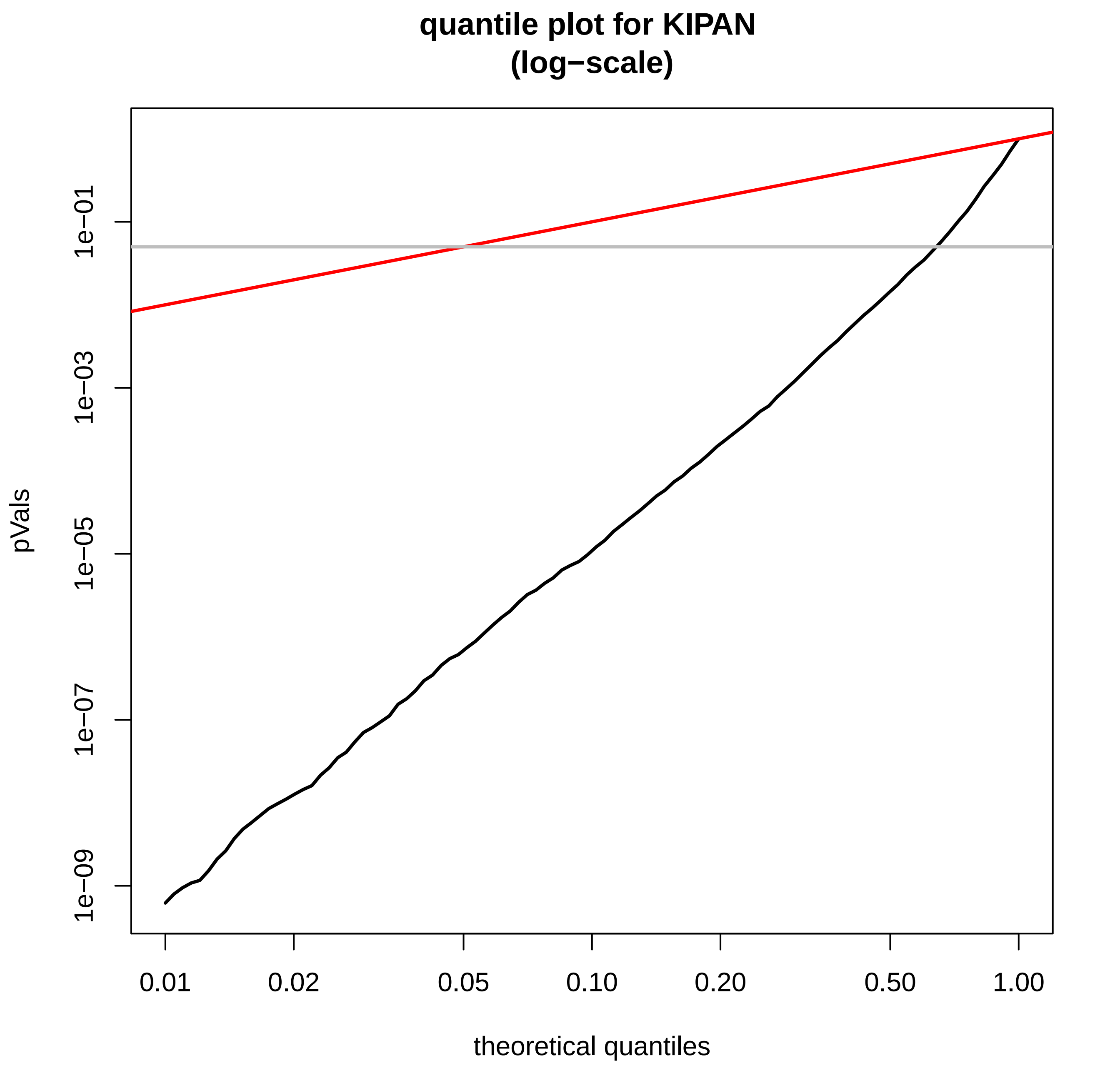
quantile plot for KIPAN
(log−scale)
pVals
1e−01
1e−03
1e−05
1e−07
1e−09
0.01
0.02
0.05
0.10
0.20
0.50
1.00
theoretical quantiles

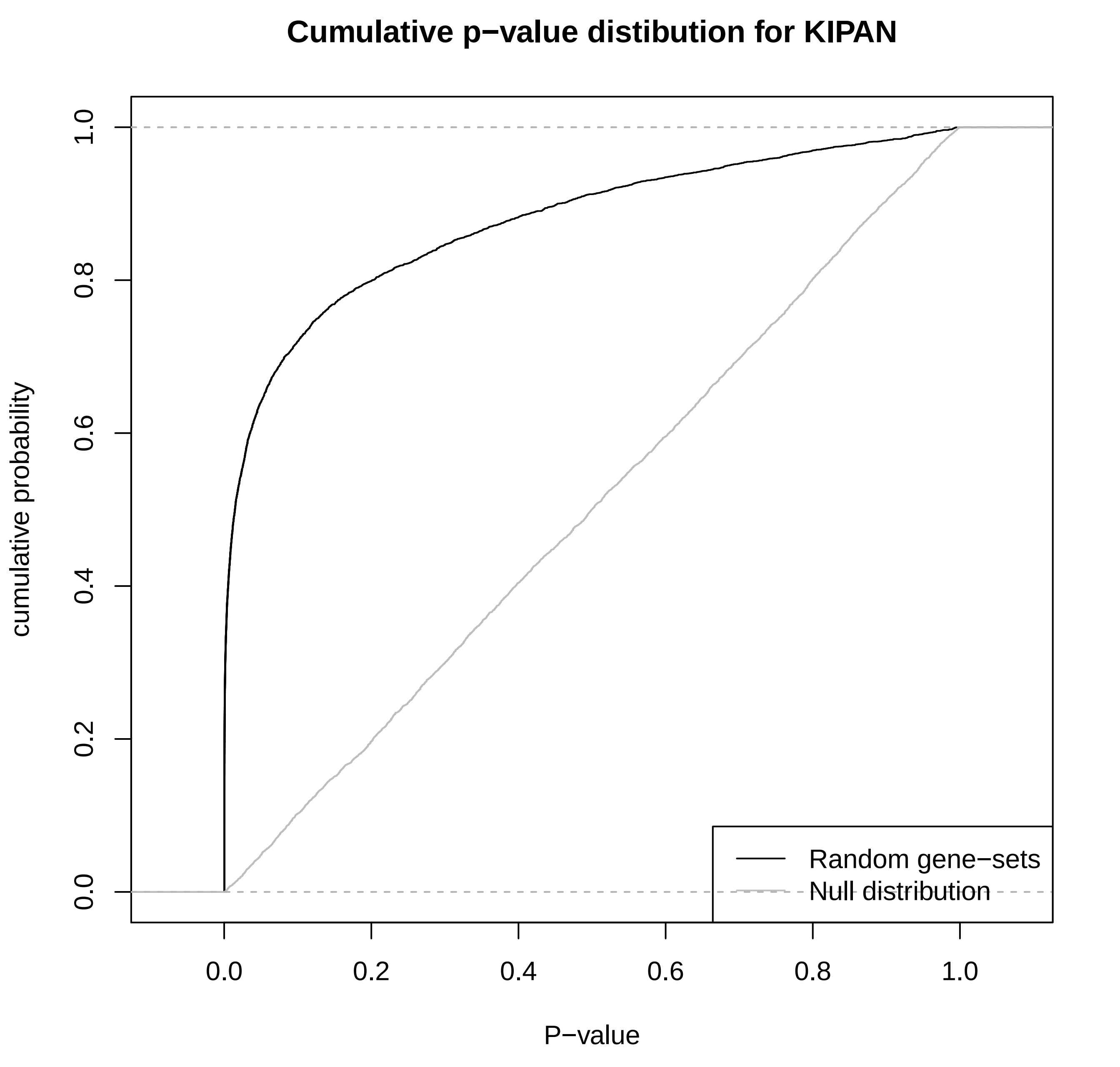
Cumulative p−value distibution for KIPAN
cumulative probability
P−value
0.0
0.2
0.4
0.6
0.8
1.0
Random gene−sets
Null distribution

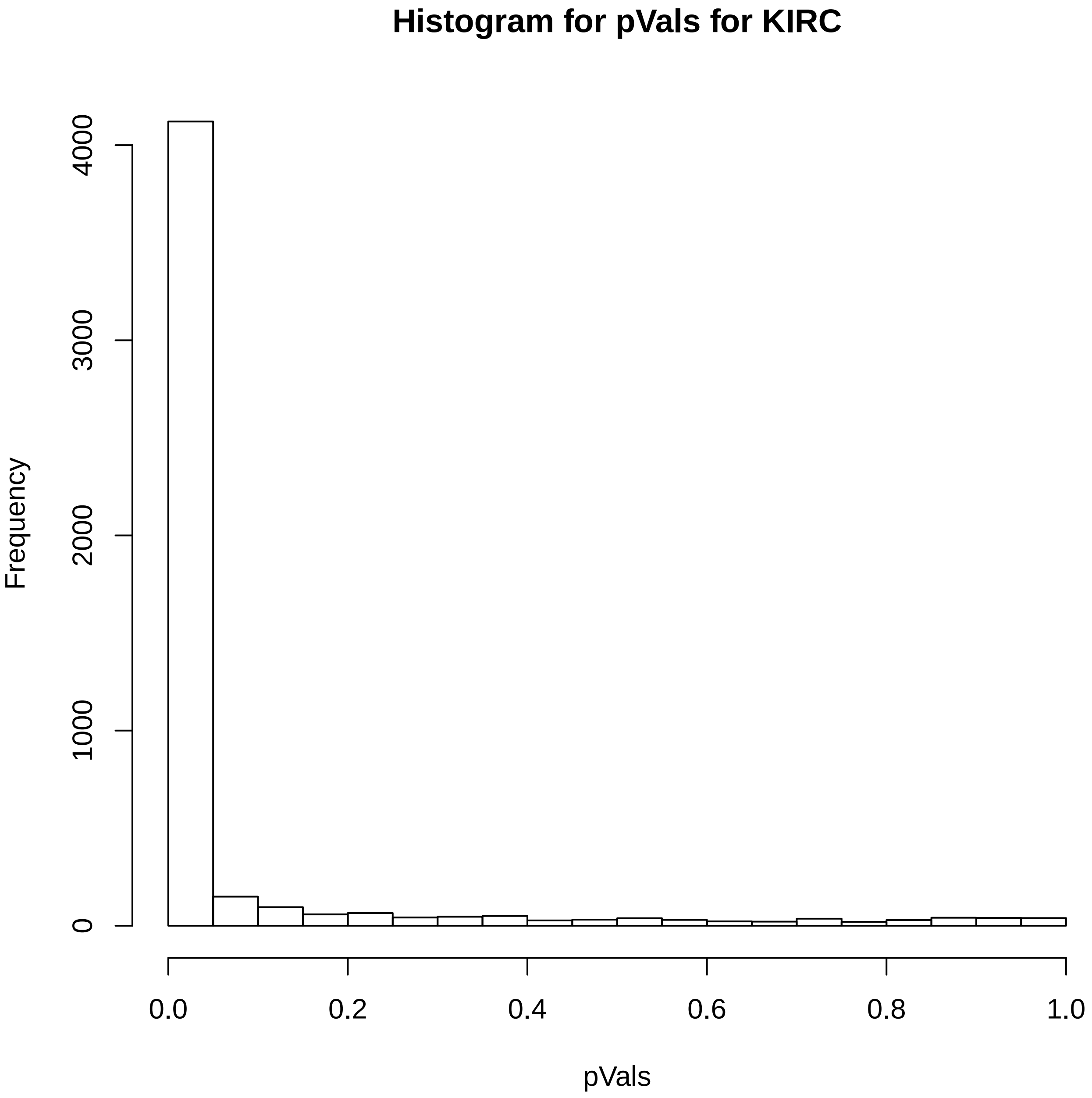
Histogram for pVals for KIRC
Frequency
0
1000
2000
3000
4000
0.0
0.2
0.4
0.6
0.8
1.0
pVals

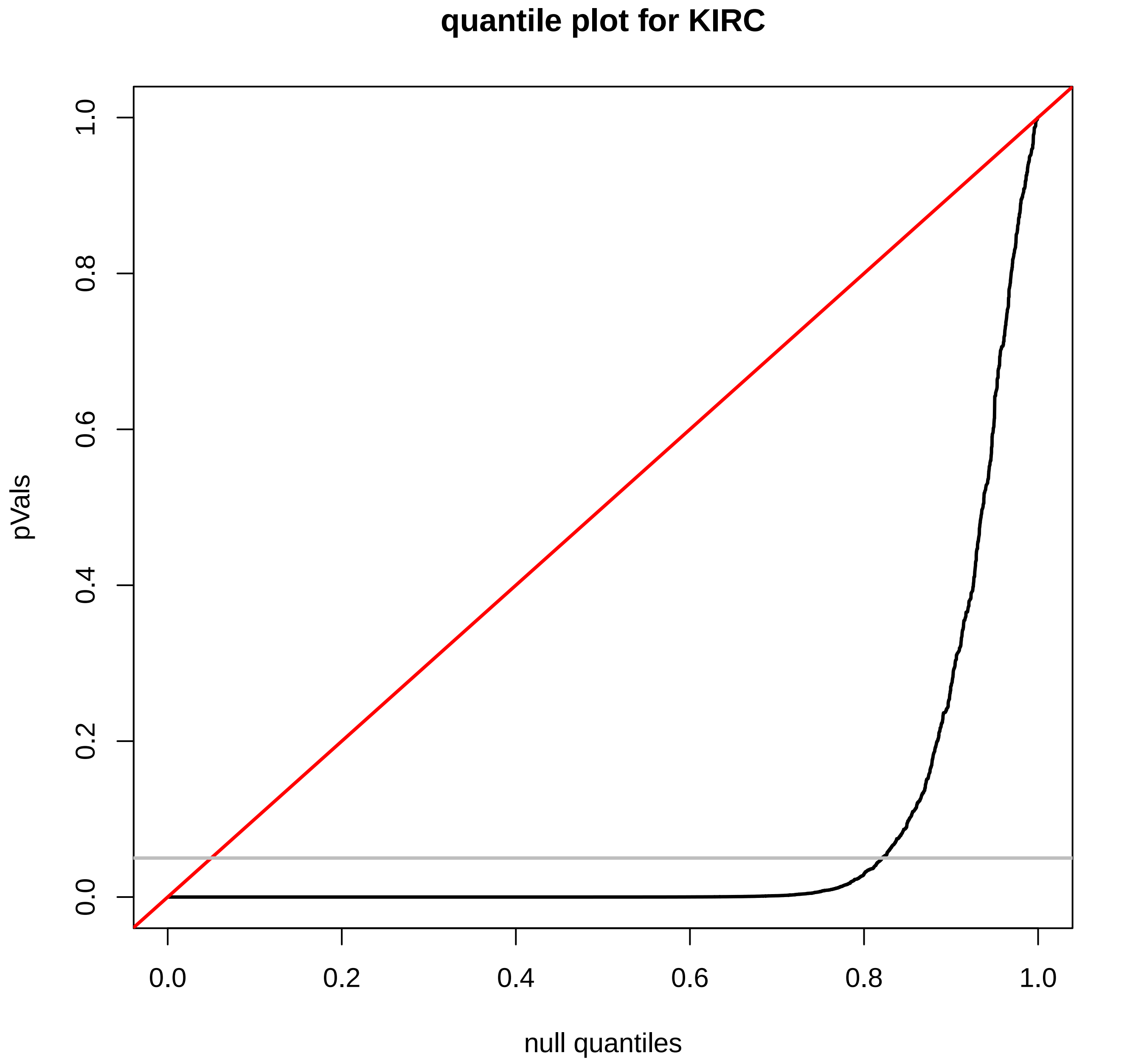

quantile plot for KIRC
pVals
1.0
0.8
0.6
0.4
0.2
0.0
0.0
0.2
0.4
0.6
0.8
1.0
null quantiles

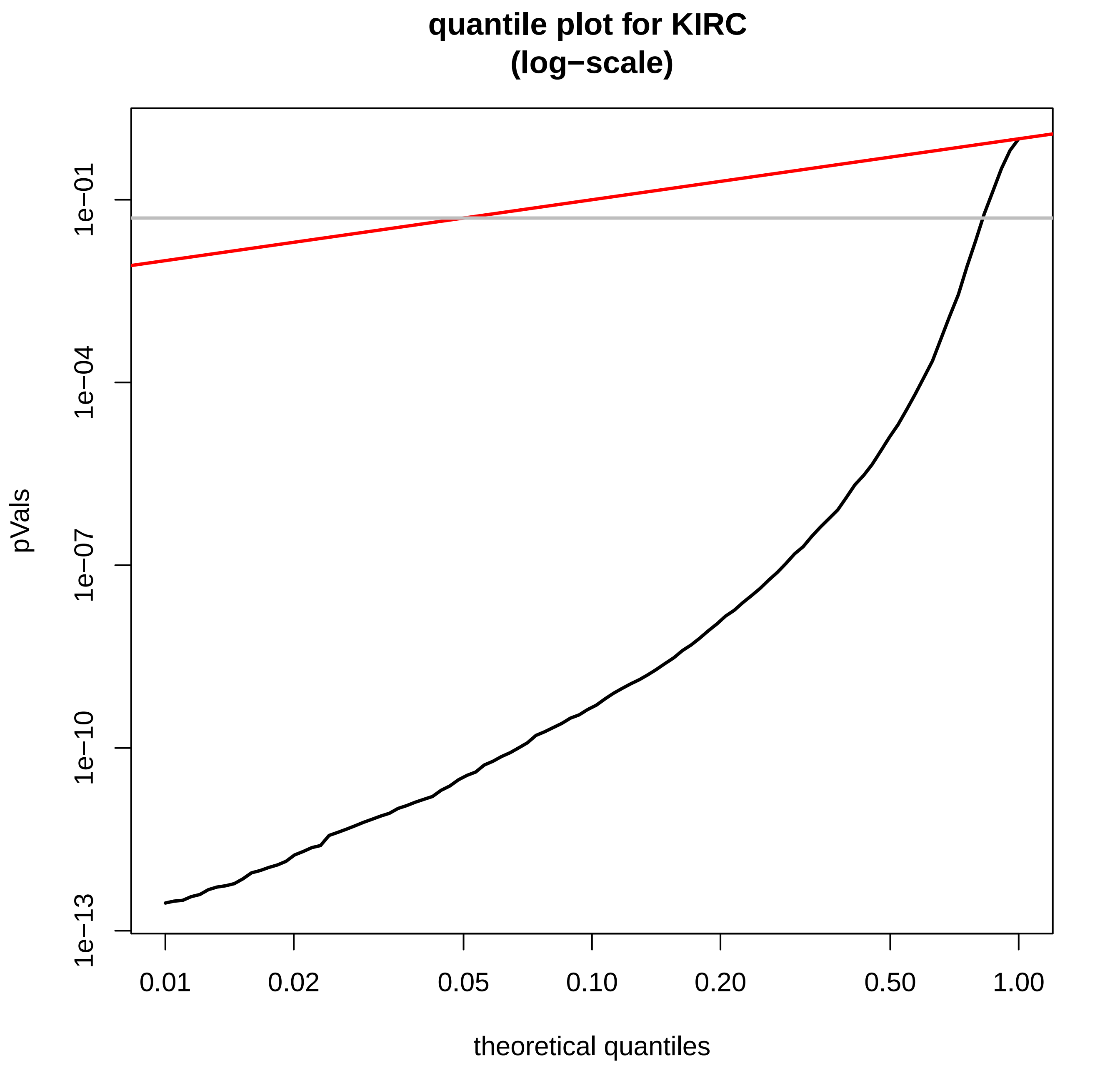
quantile plot for KIRC
(log−scale)
pVals
1e−13
1e−10
1e−07
1e−04
1e−01
0.01
0.02
0.05
0.10
0.20
0.50
1.00
theoretical quantiles

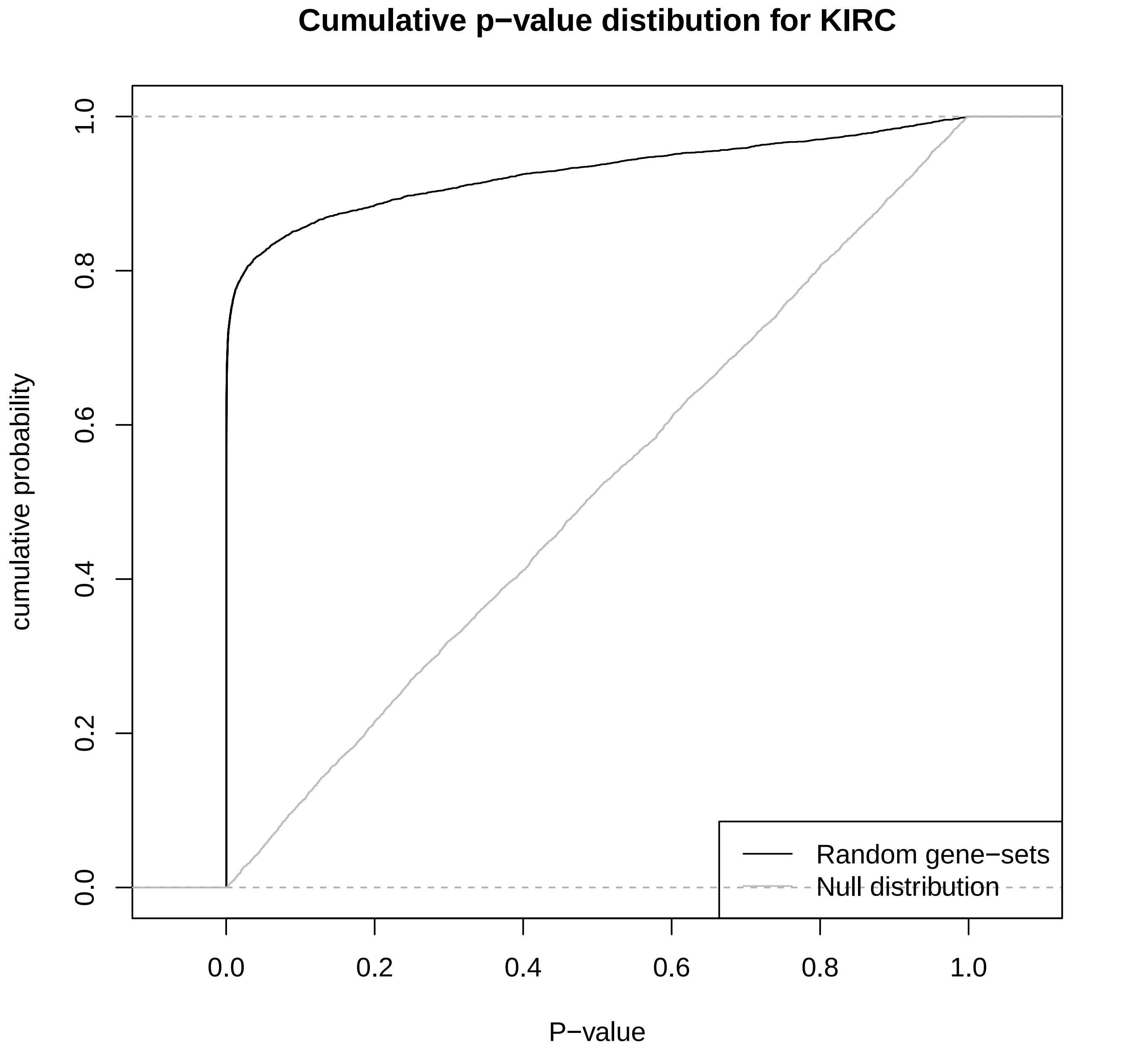
Cumulative p−value distibution for KIRC
cumulative probability
P−value
0.0
0.2
0.4
0.6
0.8
1.0
0.0
0.2
0.4
0.6
0.8
1.0
Random gene−sets
Null distribution

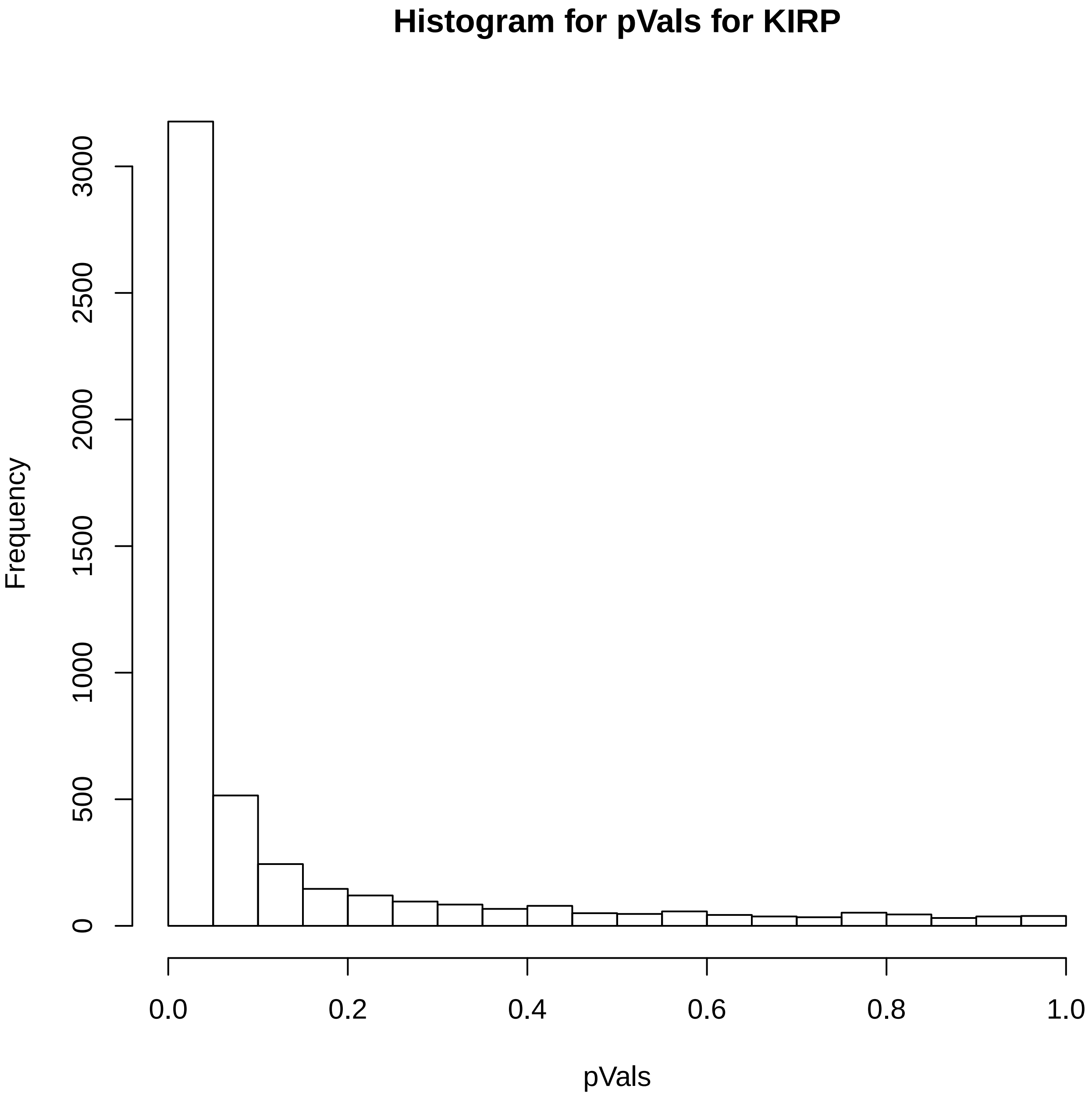
Histogram for pVals for KIRP
Frequency
0
500
1000
1500
2000
2500
3000
0.0
0.2
0.4
0.6
0.8
1.0
pVals

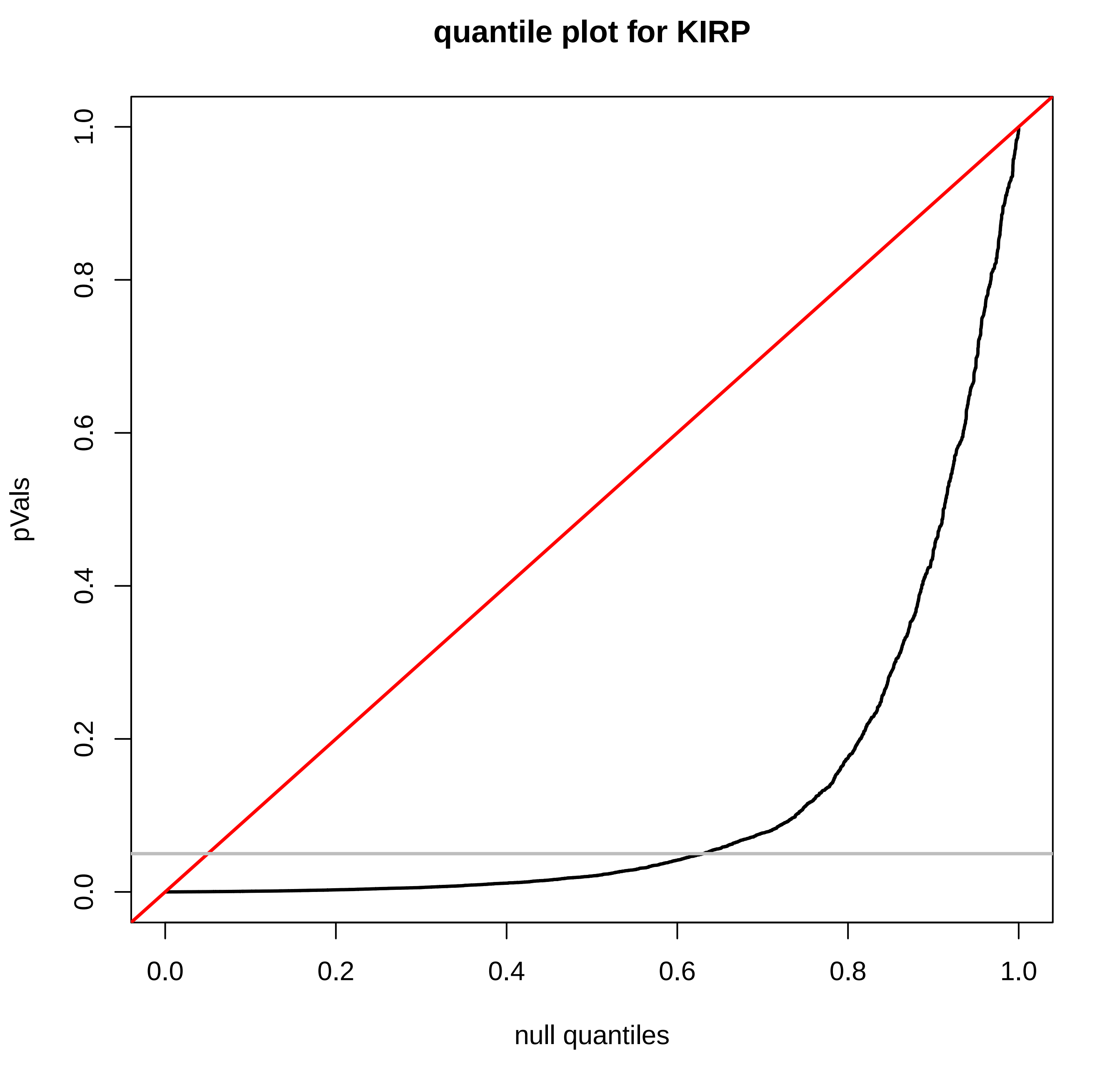
quantile plot for KIRP
pVals
0.0
0.2
0.4
0.6
0.8
1.0
0.0
0.2
0.4
0.6
0.8
1.0
null quantiles

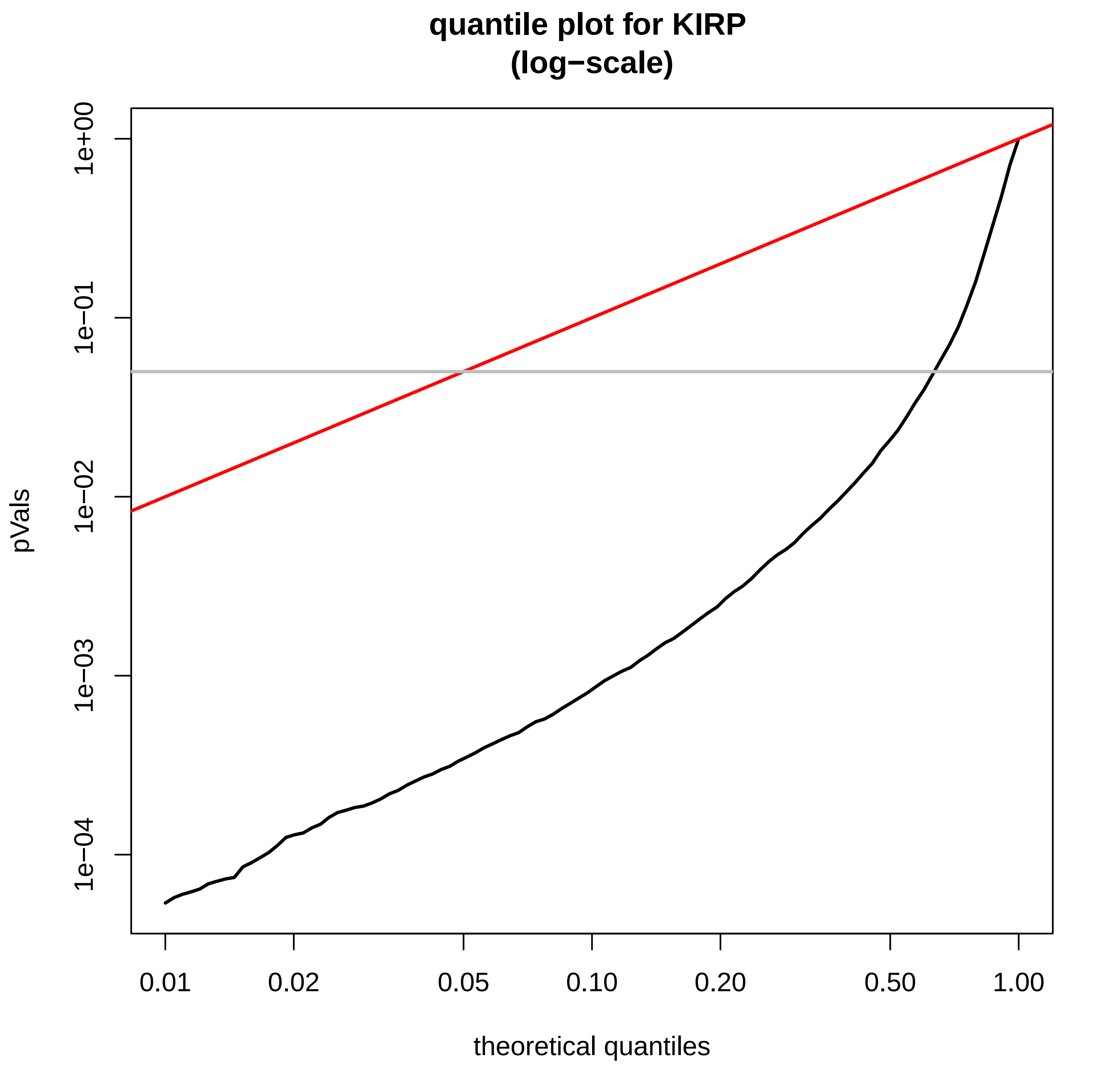
quantile plot for KIRP
(log−scale)
1e+00
1e−01
1e−02
1e−03
1e−04
pVals
0.01
0.02
0.05
0.10
0.20
0.50
1.00
theoretical quantiles

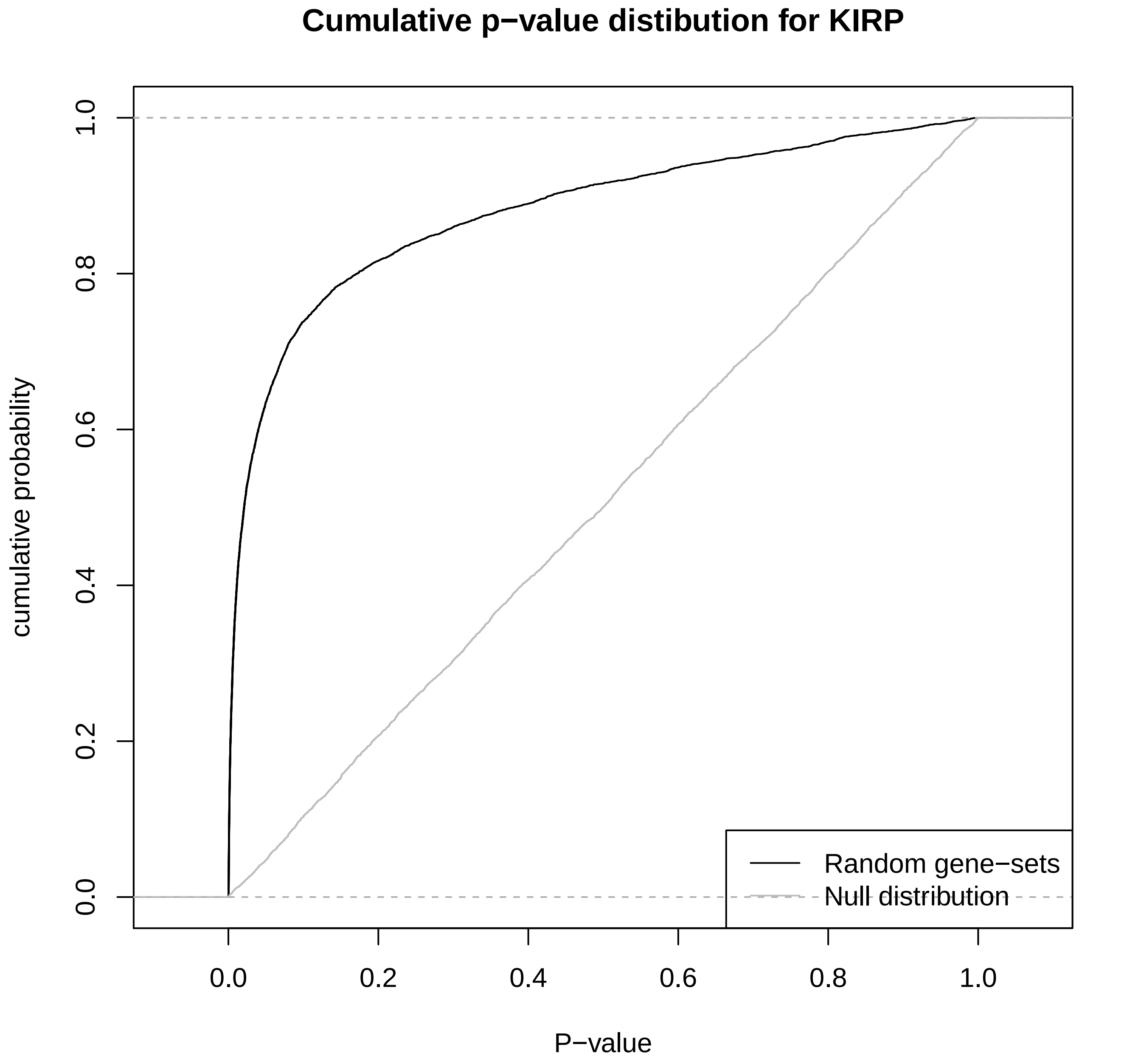
Cumulative p−value distibution for KIRP
cumulative probability
P−value
0.0
0.2
0.4
0.6
0.8
1.0
Random gene−sets
Null distribution

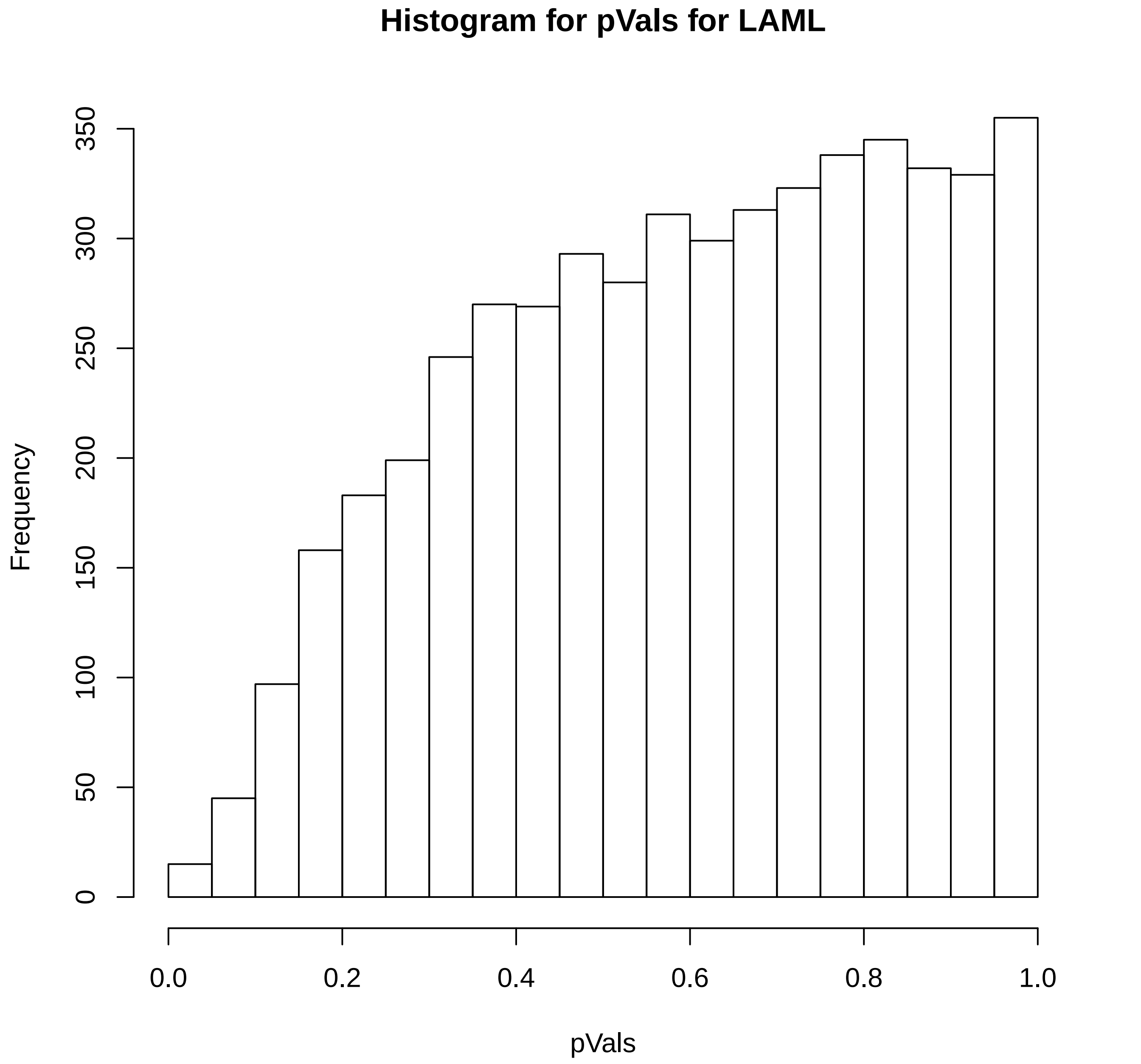

Histogram for pVals for LAML
Frequency
0
50
100
150
200
250
300
350
0.0
0.2
0.4
0.6
0.8
1.0
pVals

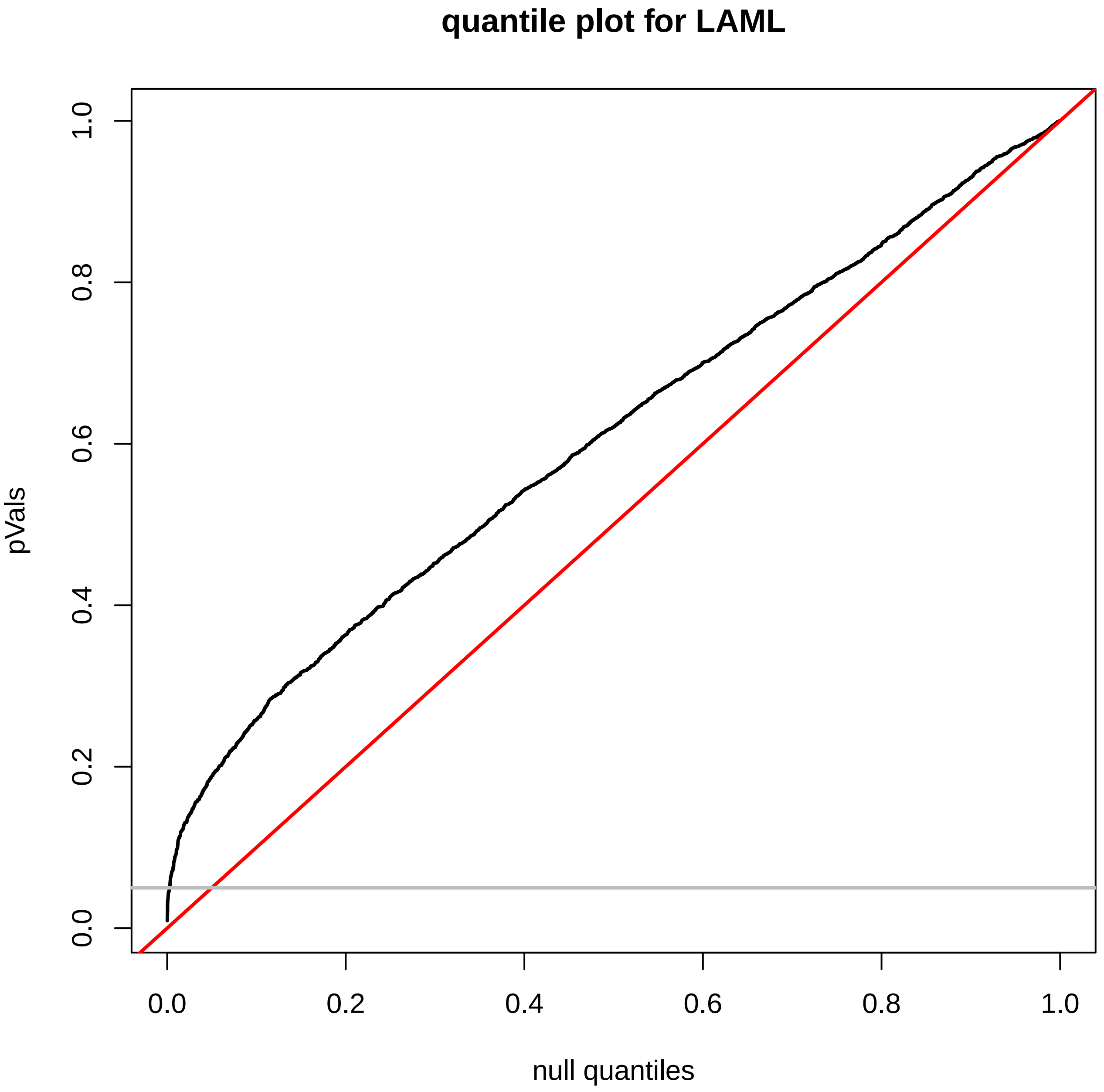
quantile plot for LAML
pVals
null quantiles
0.0
0.2
0.4
0.6
0.8
1.0

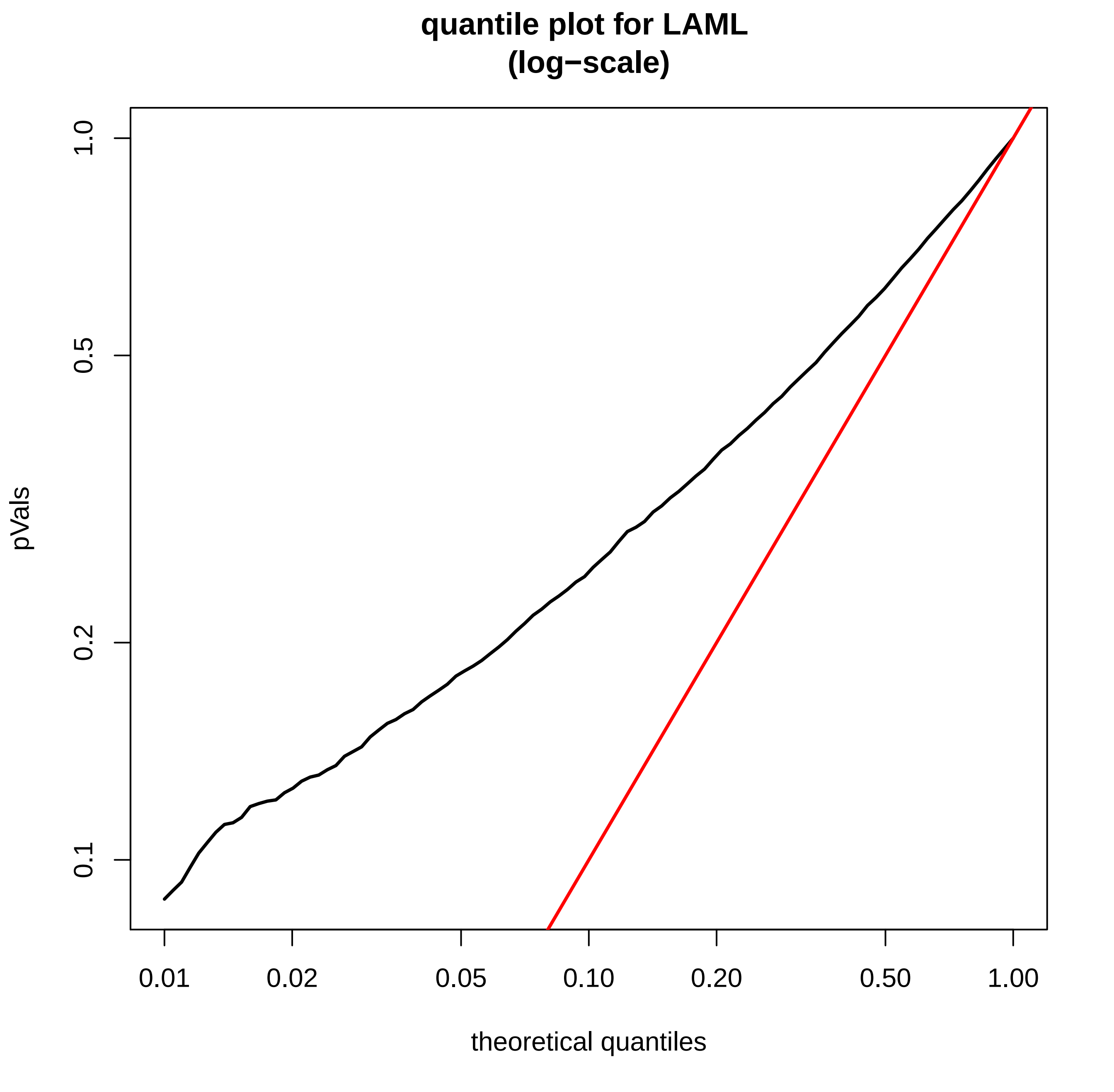

quantile plot for LAML
(log−scale)
pVals
1.0
0.5
0.2
0.1
0.01
0.02
0.05
0.10
0.20
0.50
1.00
theoretical quantiles

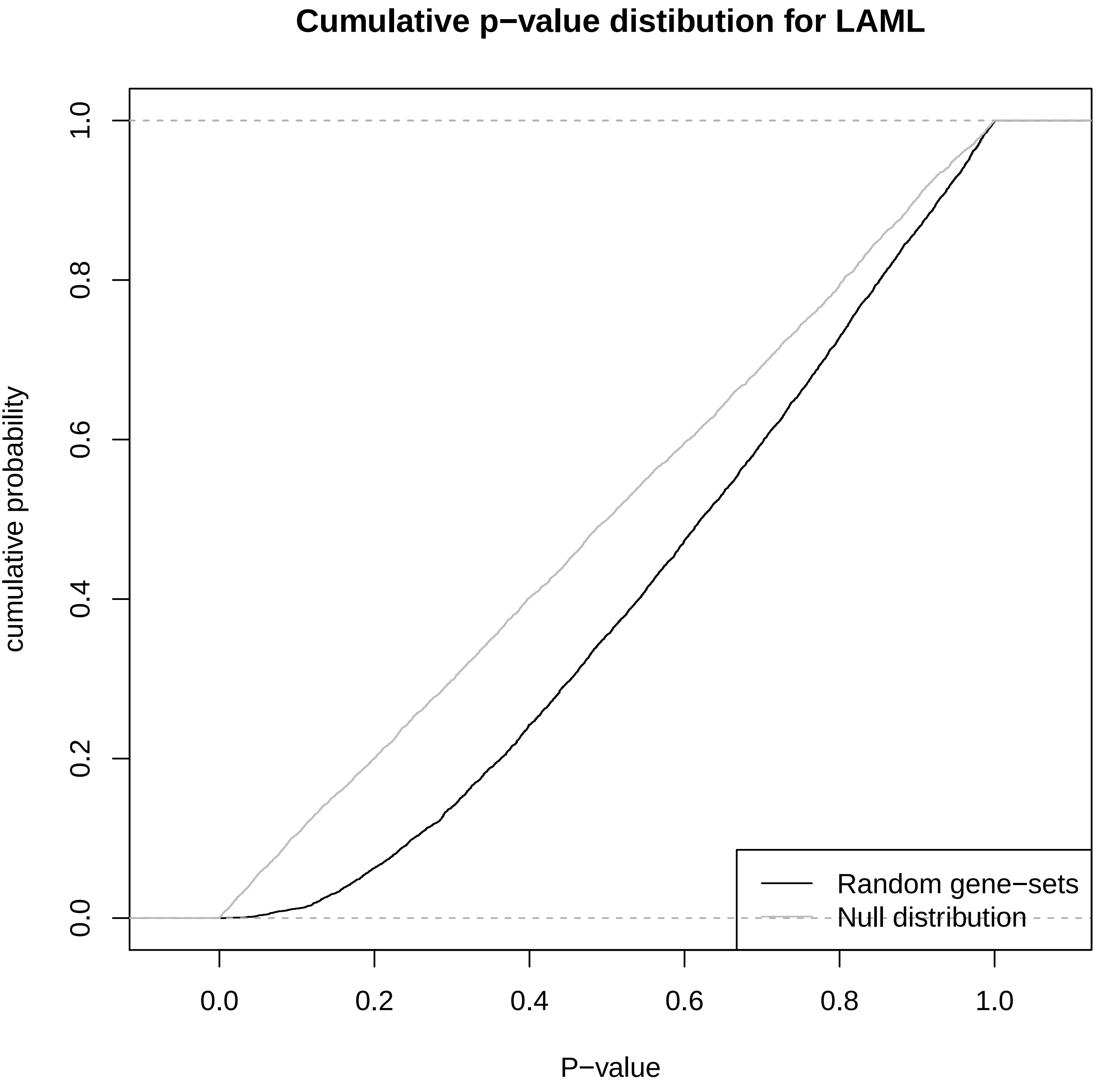
Cumulative p−value distibution for LAML
cumulative probability
0.0
0.2
0.4
0.6
0.8
1.0
P−value
0.0
0.2
0.4
0.6
0.8
1.0
Random gene−sets
Null distribution

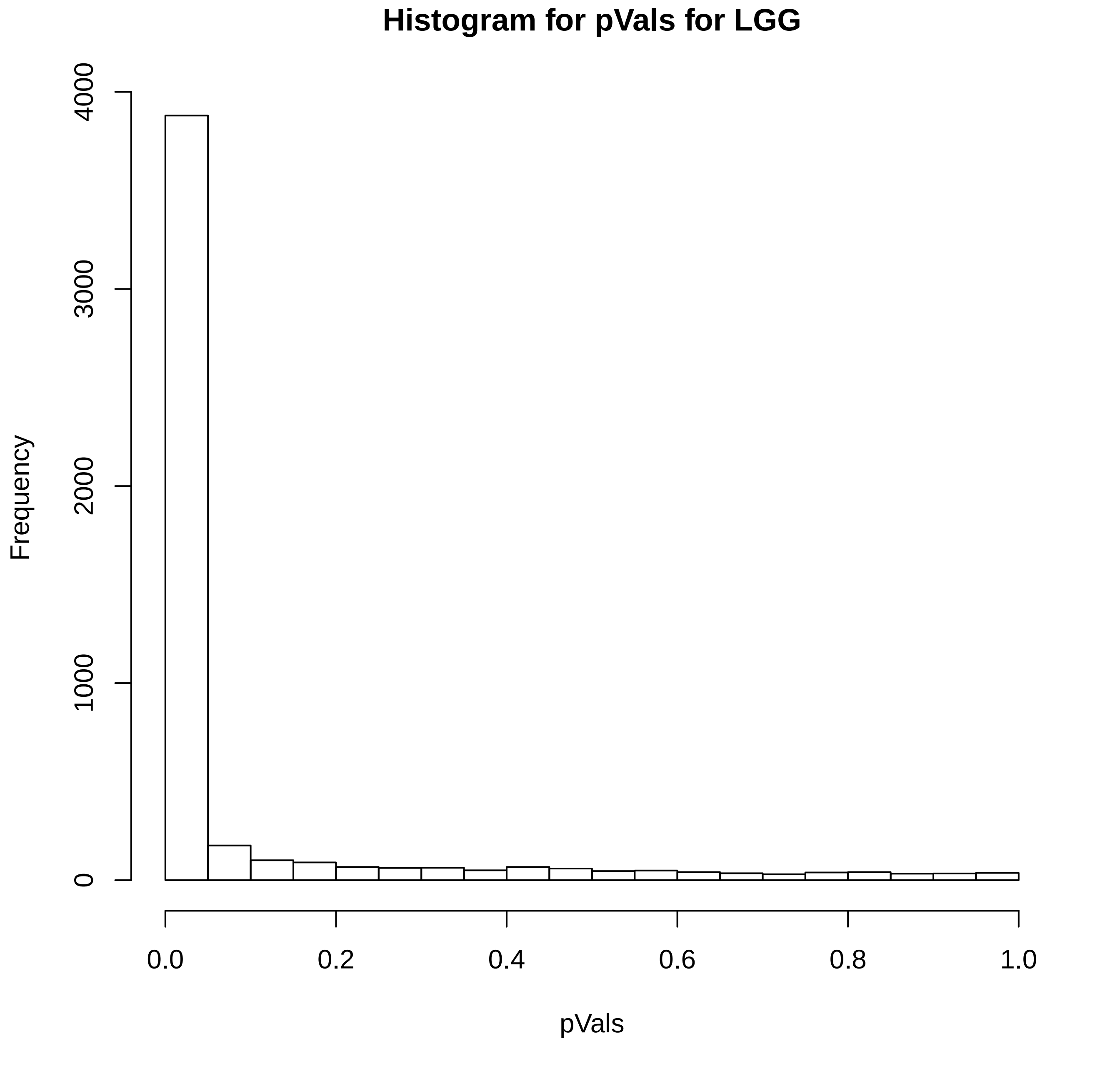

Histogram for pVals for LGG
Frequency
0
1000
2000
3000
4000
0.0
0.2
0.4
0.6
0.8
1.0
pVals

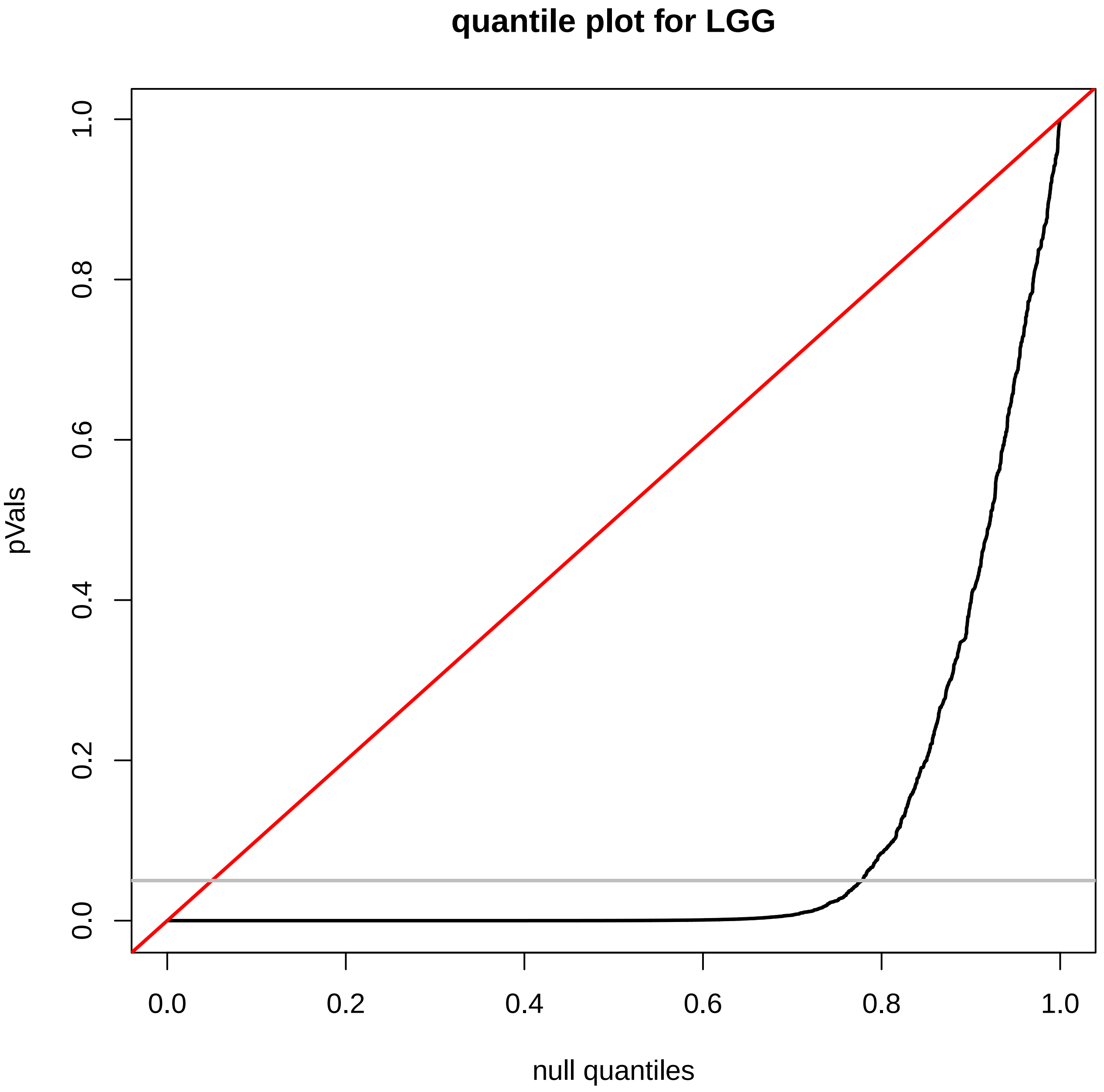
quantile plot for LGG
pVals
0.0
0.2
0.4
0.6
0.8
1.0
null quantiles
0.0
0.2
0.4
0.6
0.8
1.0

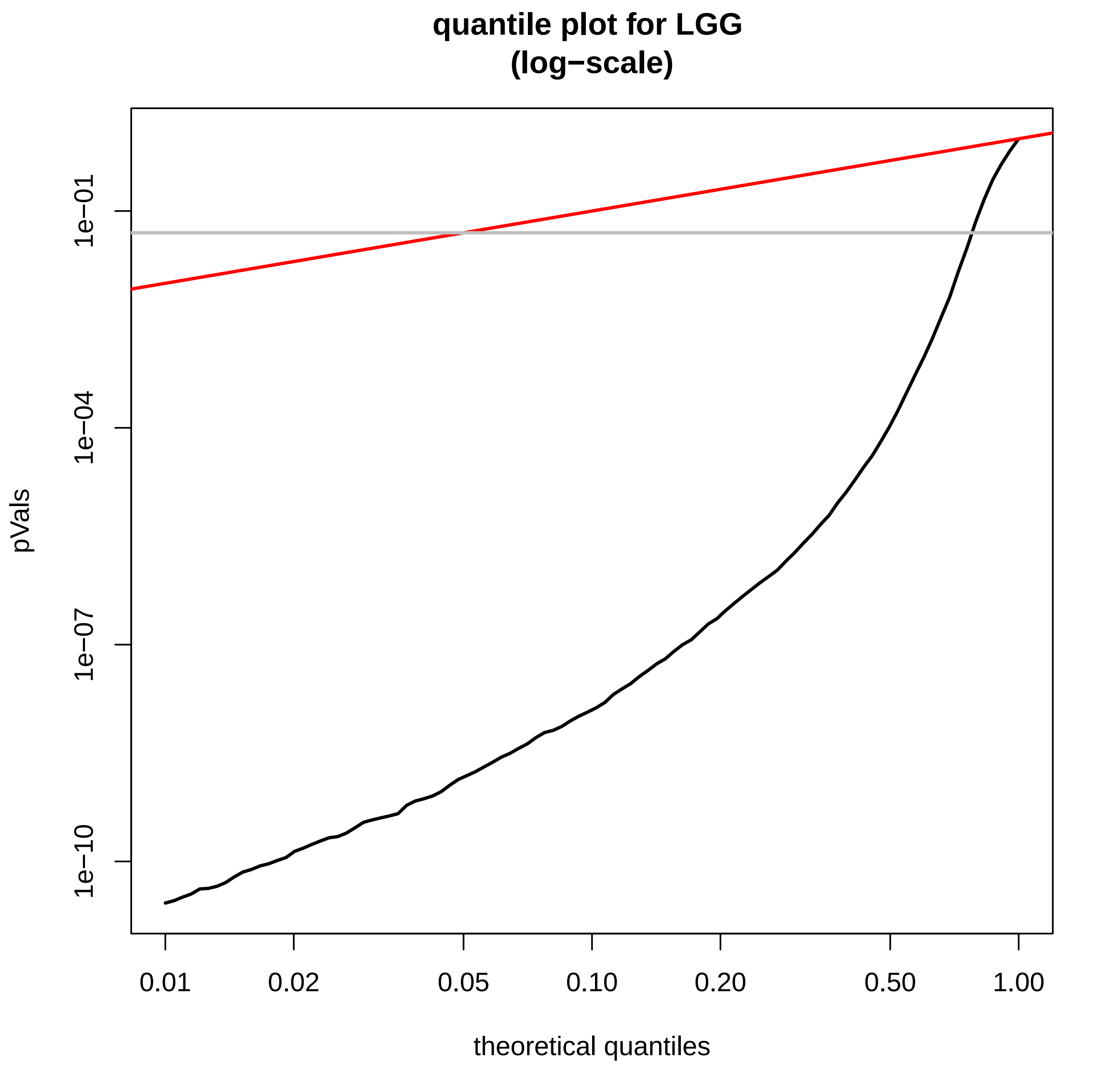

quantile plot for LGG
(log−scale)
pVals
1e−01
1e−04
1e−07
1e−10
0.01
0.02
0.05
0.10
0.20
0.50
1.00
theoretical quantiles

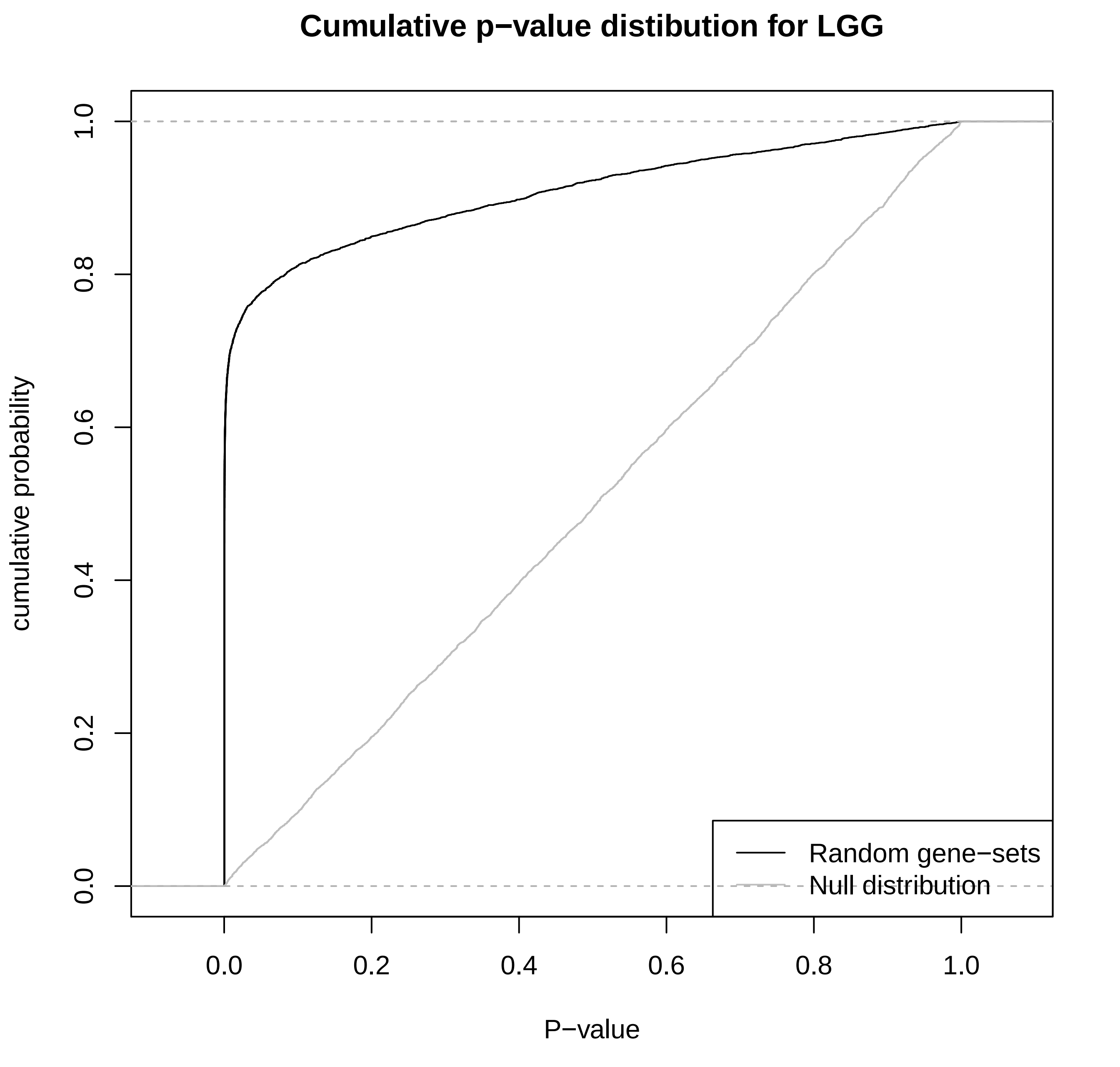
Cumulative p−value distibution for LGG
cumulative probability
P−value
0.0
0.2
0.4
0.6
0.8
1.0
Random gene−sets
Null distribution

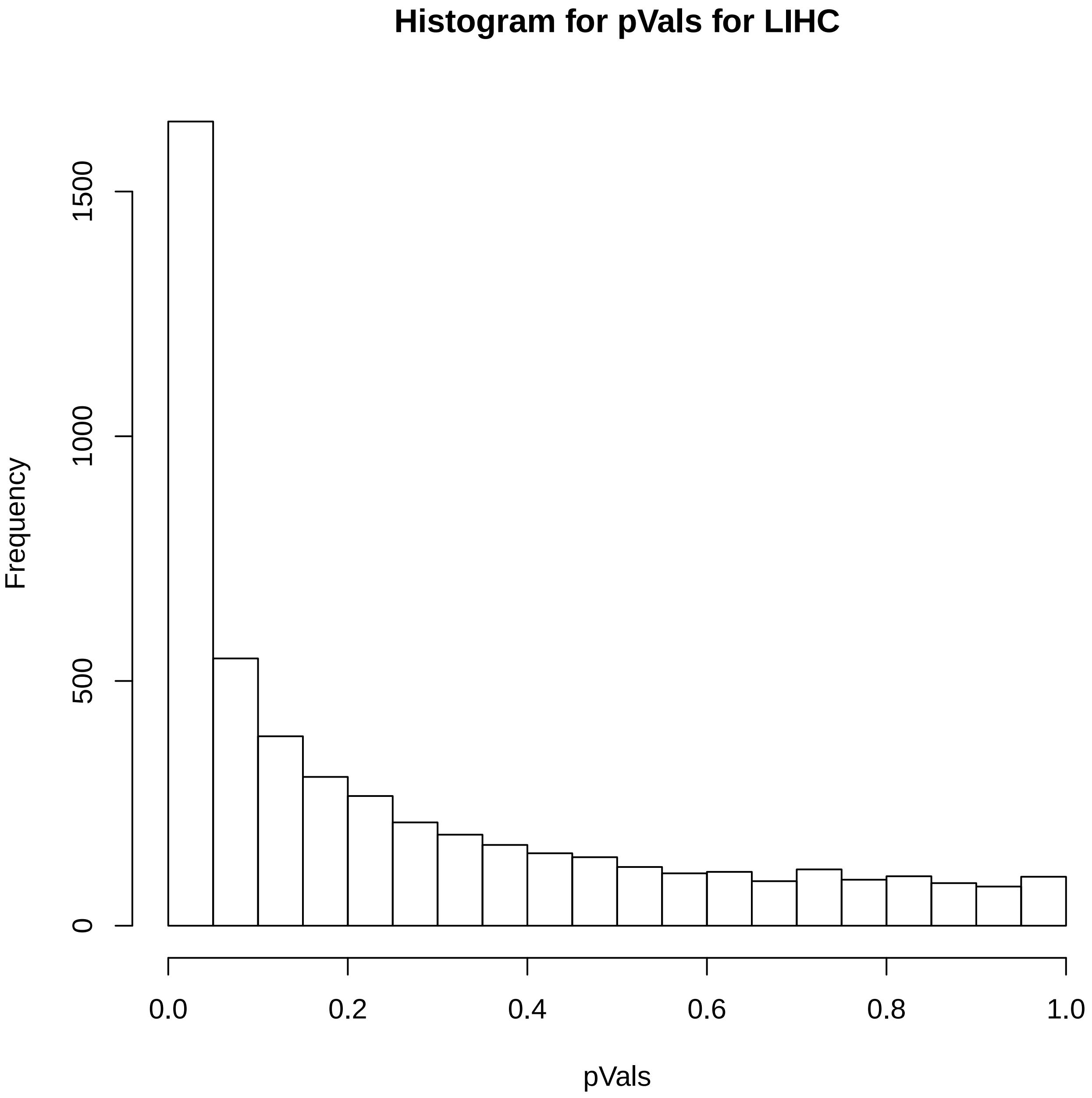
Histogram for pVals for LIHC
Frequency
0
500
1000
1500
0.0
0.2
0.4
0.6
0.8
1.0
pVals

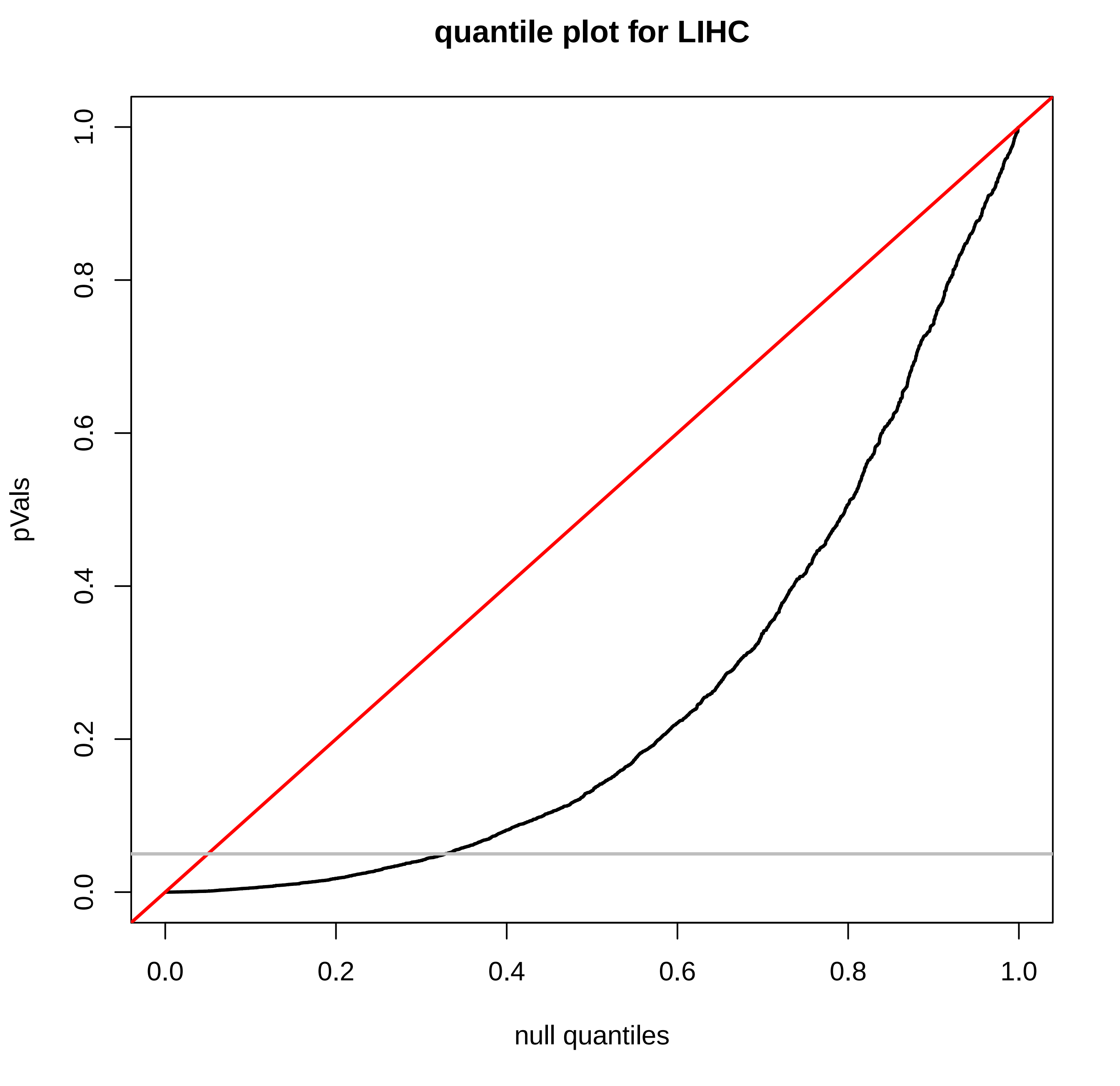

quantile plot for LIHC
pVals
0.0
0.2
0.4
0.6
0.8
1.0
null quantiles

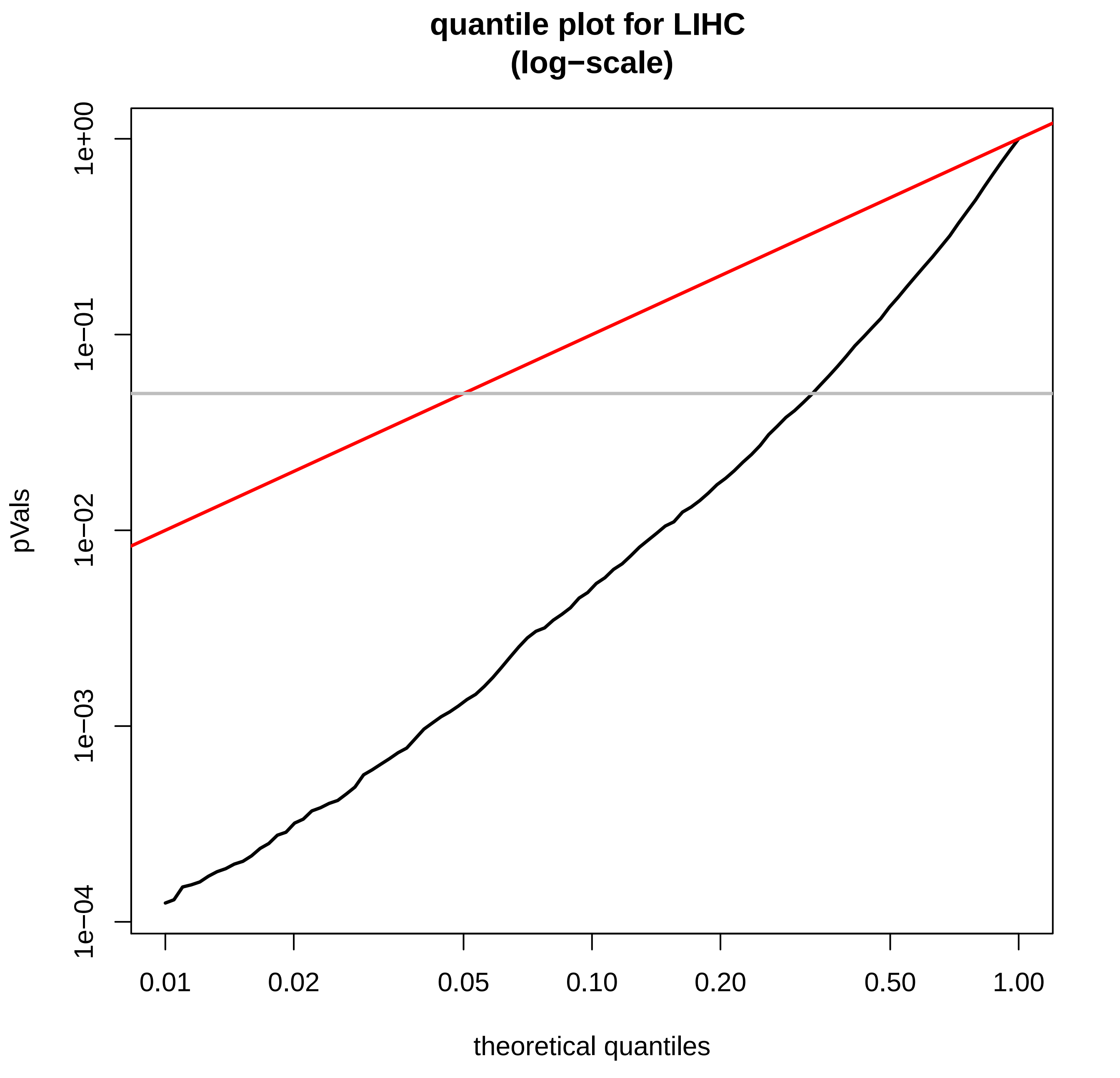
quantile plot for LIHC
(log−scale)
pVals
1e+00
1e−01
1e−02
1e−03
1e−04
0.01
0.02
0.05
0.10
0.20
0.50
1.00
theoretical quantiles

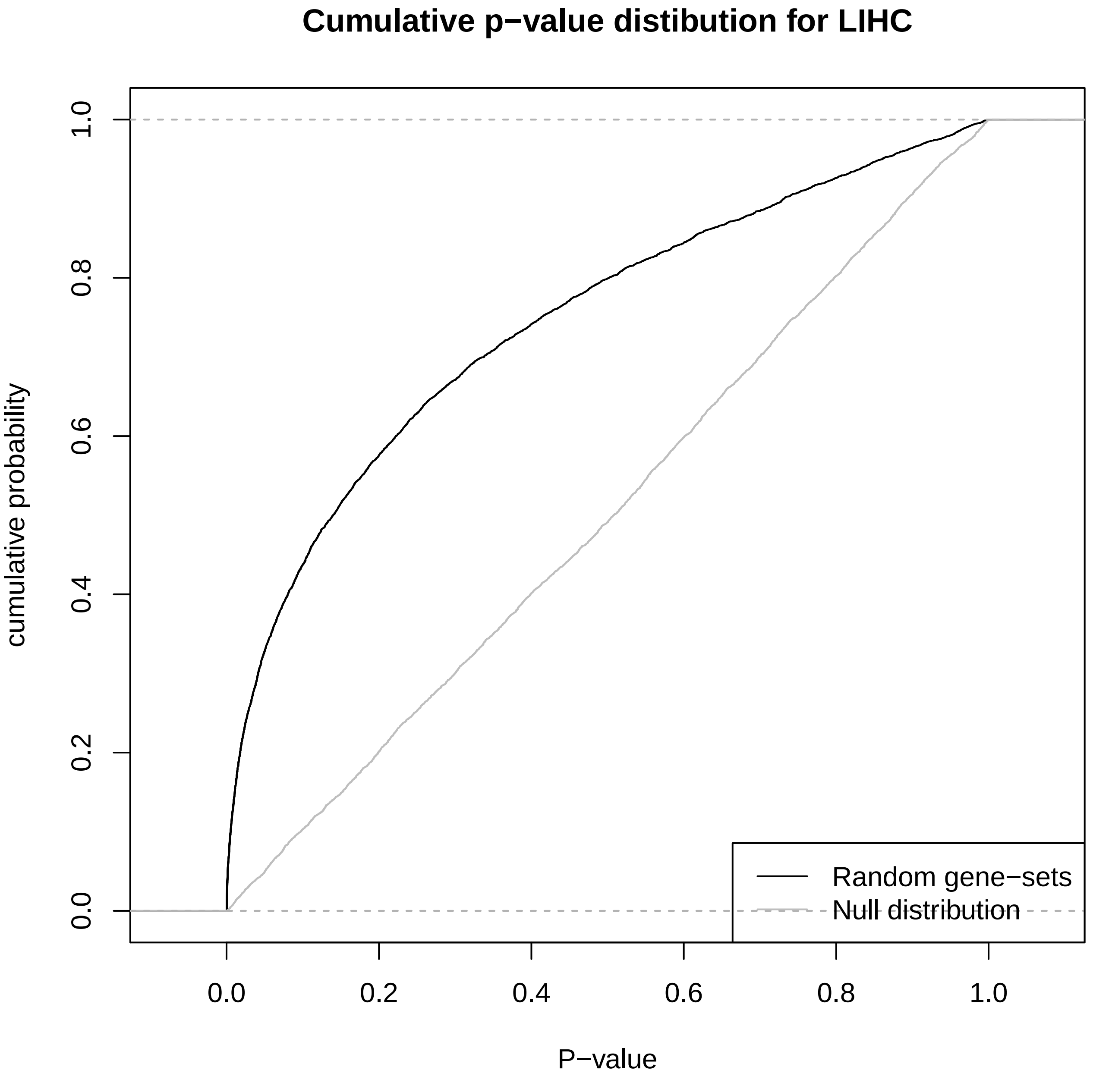
Cumulative p−value distibution for LIHC
cumulative probability
P−value
0.0
0.2
0.4
0.6
0.8
1.0
Random gene−sets
Null distribution

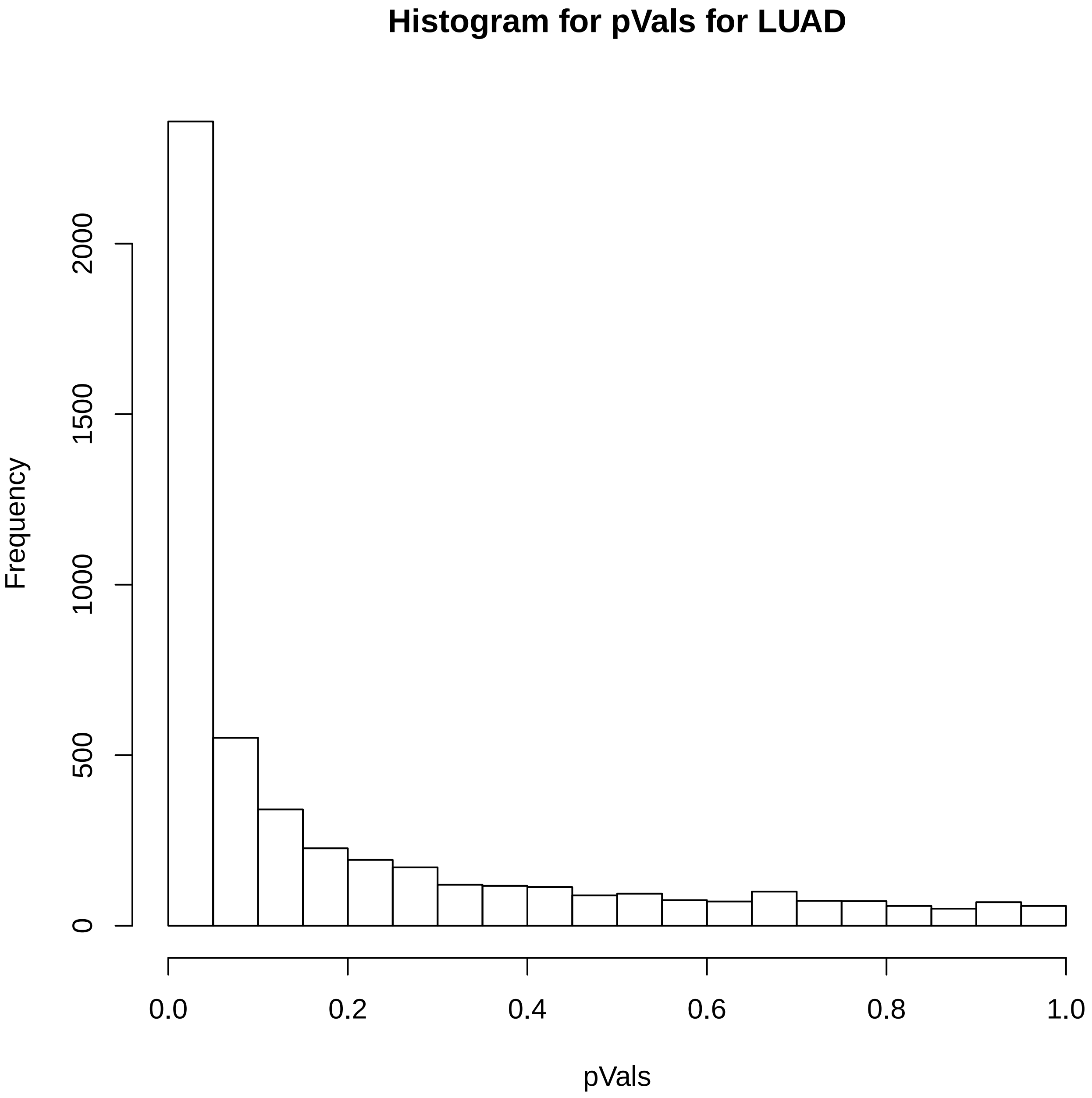
Histogram for pVals for LUAD
Frequency
0
500
1000
1500
2000
0.0
0.2
0.4
0.6
0.8
1.0
pVals

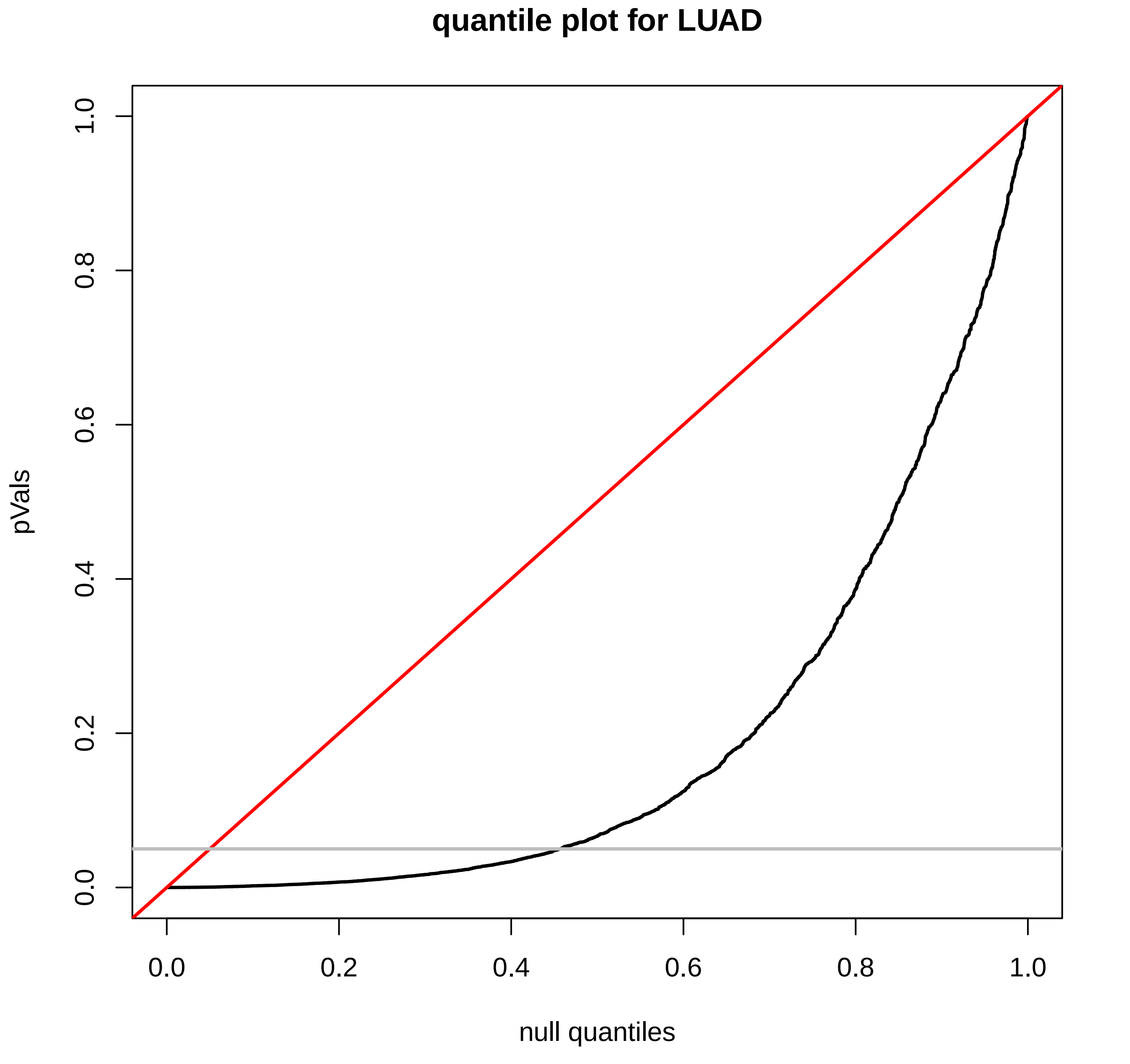
quantile plot for LUAD
pVals
null quantiles
0.0
0.2
0.4
0.6
0.8
1.0
0.0
0.2
0.4
0.6
0.8
1.0

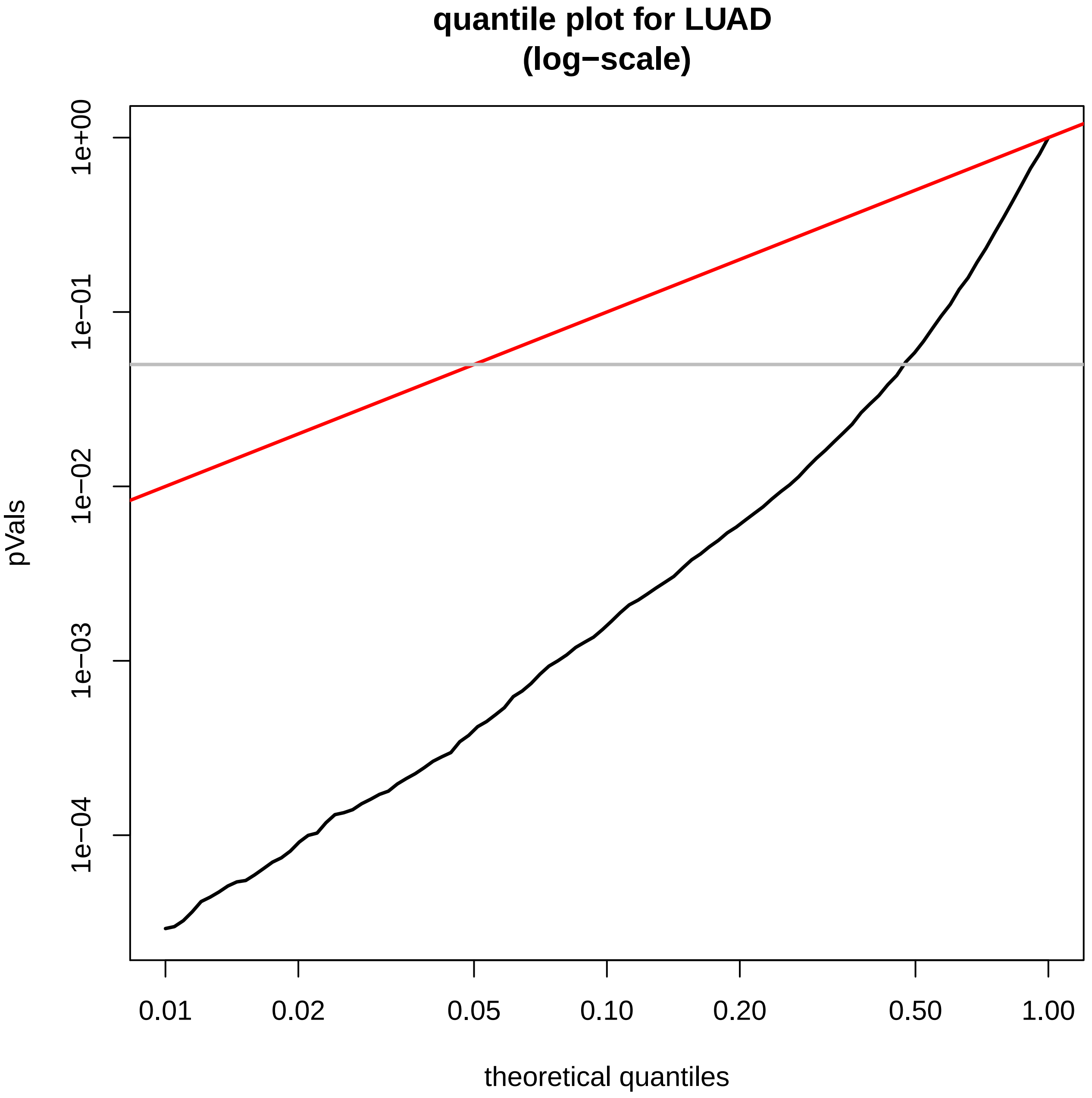

quantile plot for LUAD
(log−scale)
pVals
1e+00
1e−01
1e−02
1e−03
1e−04
0.01
0.02
0.05
0.10
0.20
0.50
1.00
theoretical quantiles

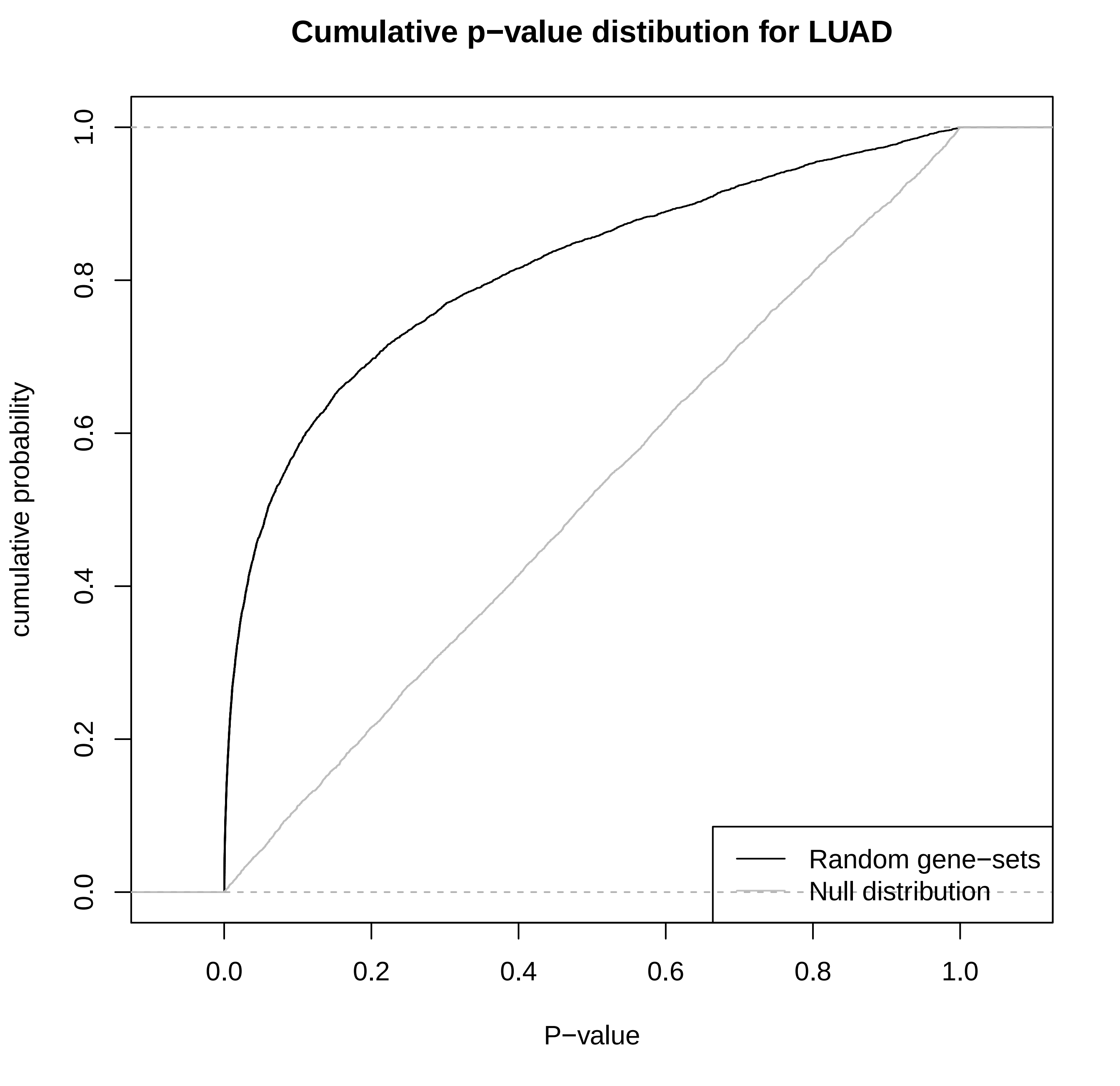

Cumulative p−value distibution for LUAD
cumulative probability
0.0
0.2
0.4
0.6
0.8
1.0
P−value
0.0
0.2
0.4
0.6
0.8
1.0
Random gene−sets
Null distribution

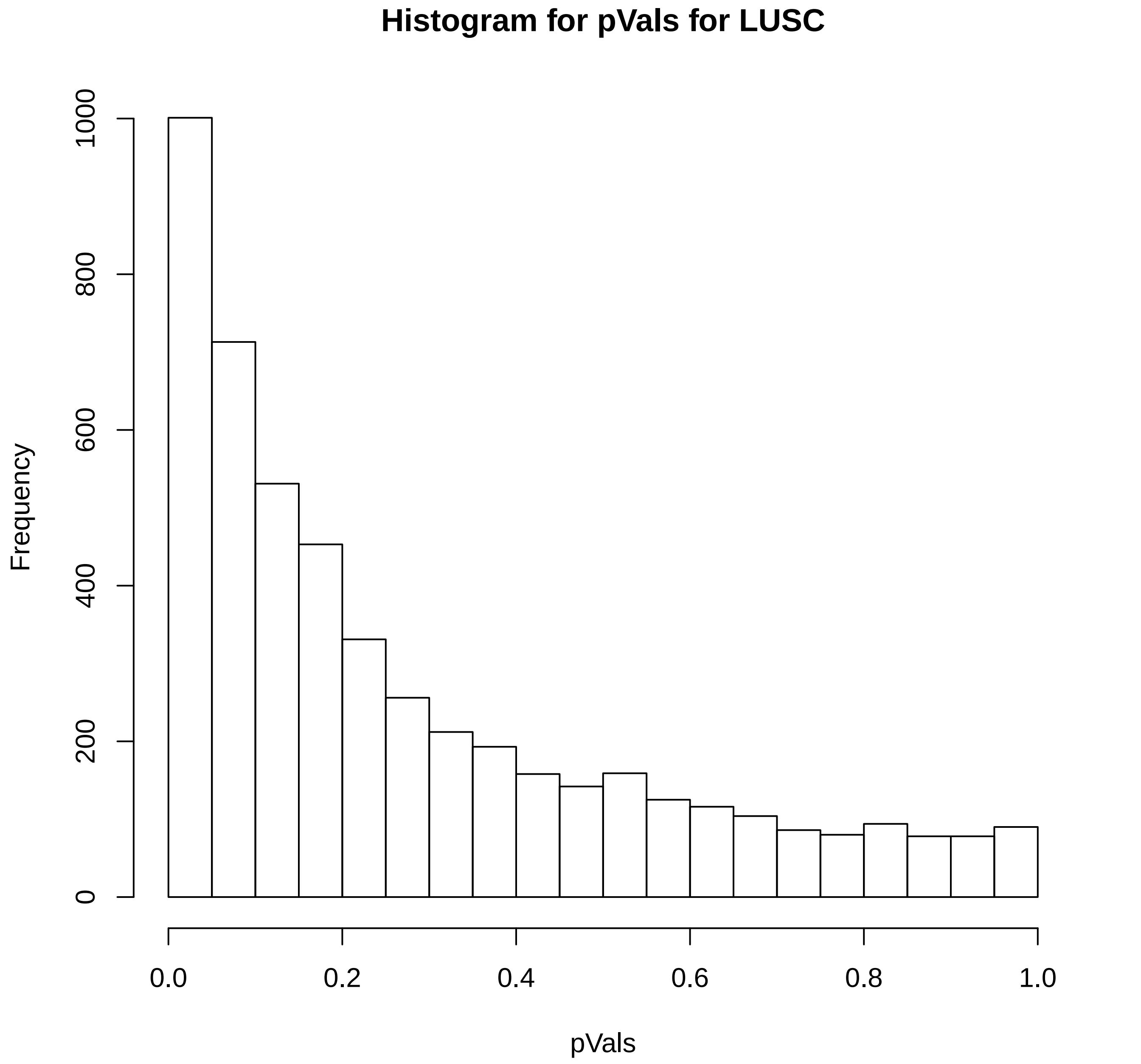
Histogram for pVals for LUSC
Frequency
0
200
400
600
800
1000
0.0
0.2
0.4
0.6
0.8
1.0
pVals

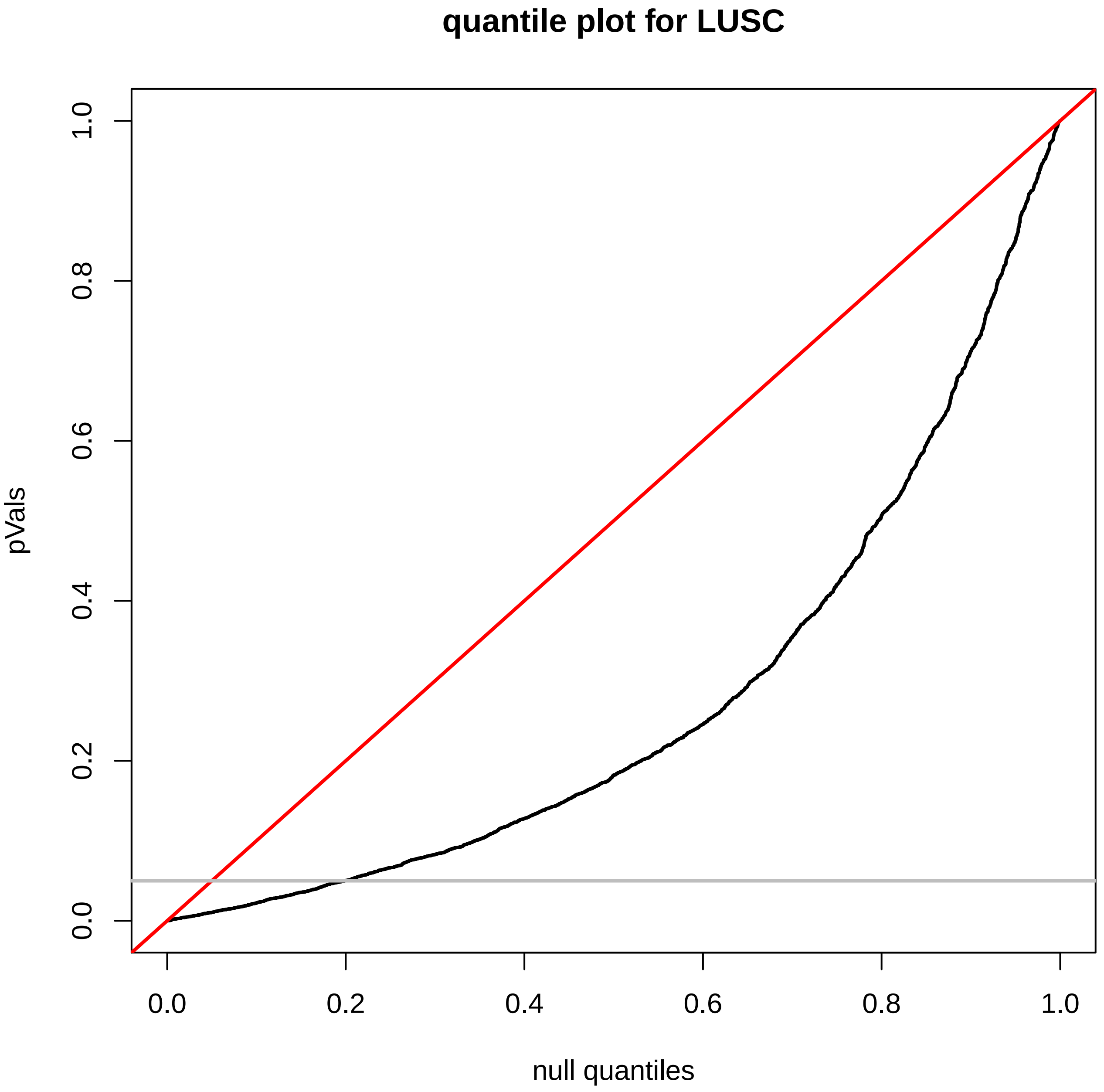

quantile plot for LUSC
pVals
null quantiles
0.0
0.2
0.4
0.6
0.8
1.0

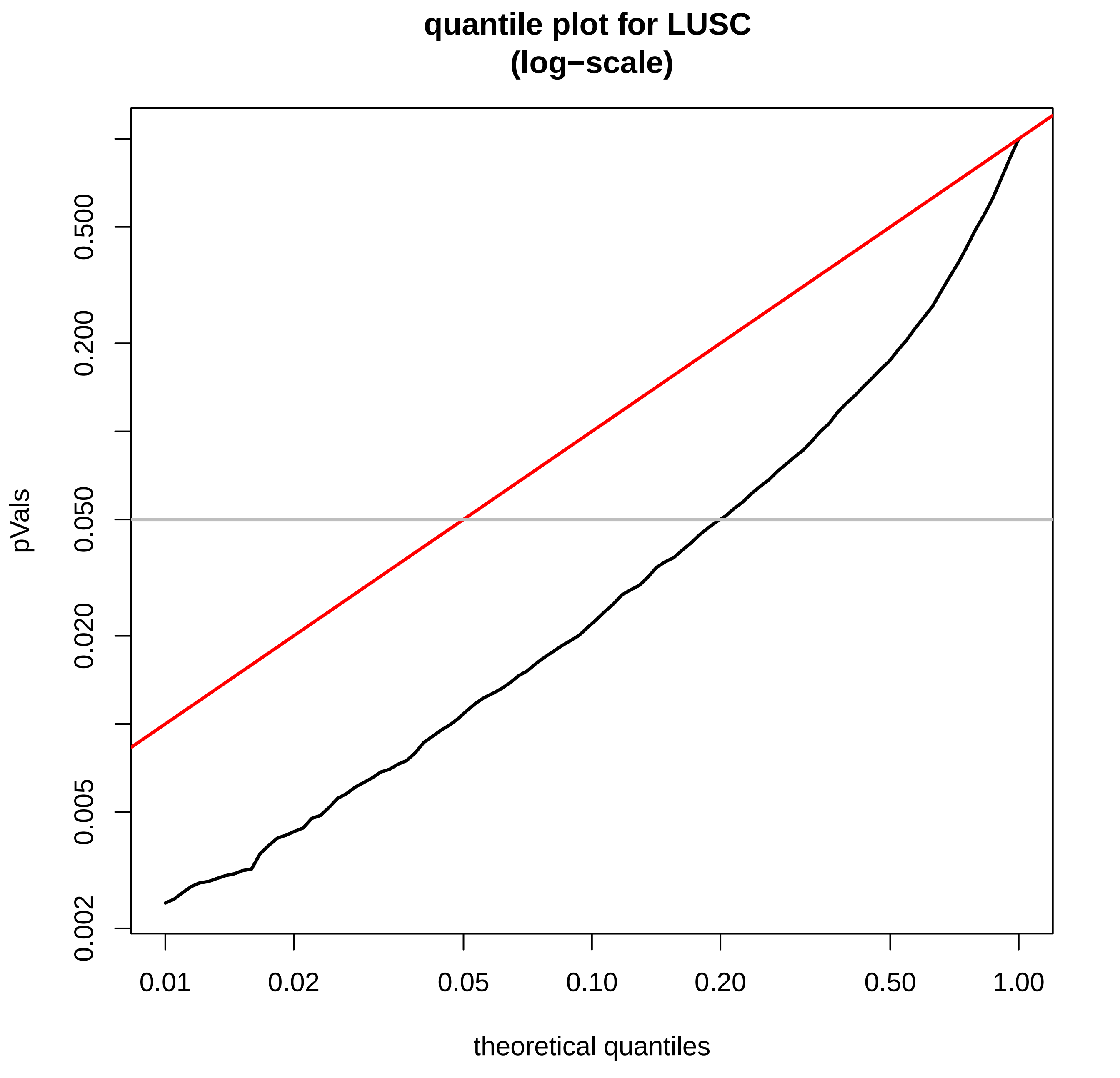

quantile plot for LUSC
(log−scale)
pVals
0.002
0.005
0.020
0.050
0.200
0.500
0.01
0.02
0.05
0.10
0.20
0.50
1.00
theoretical quantiles

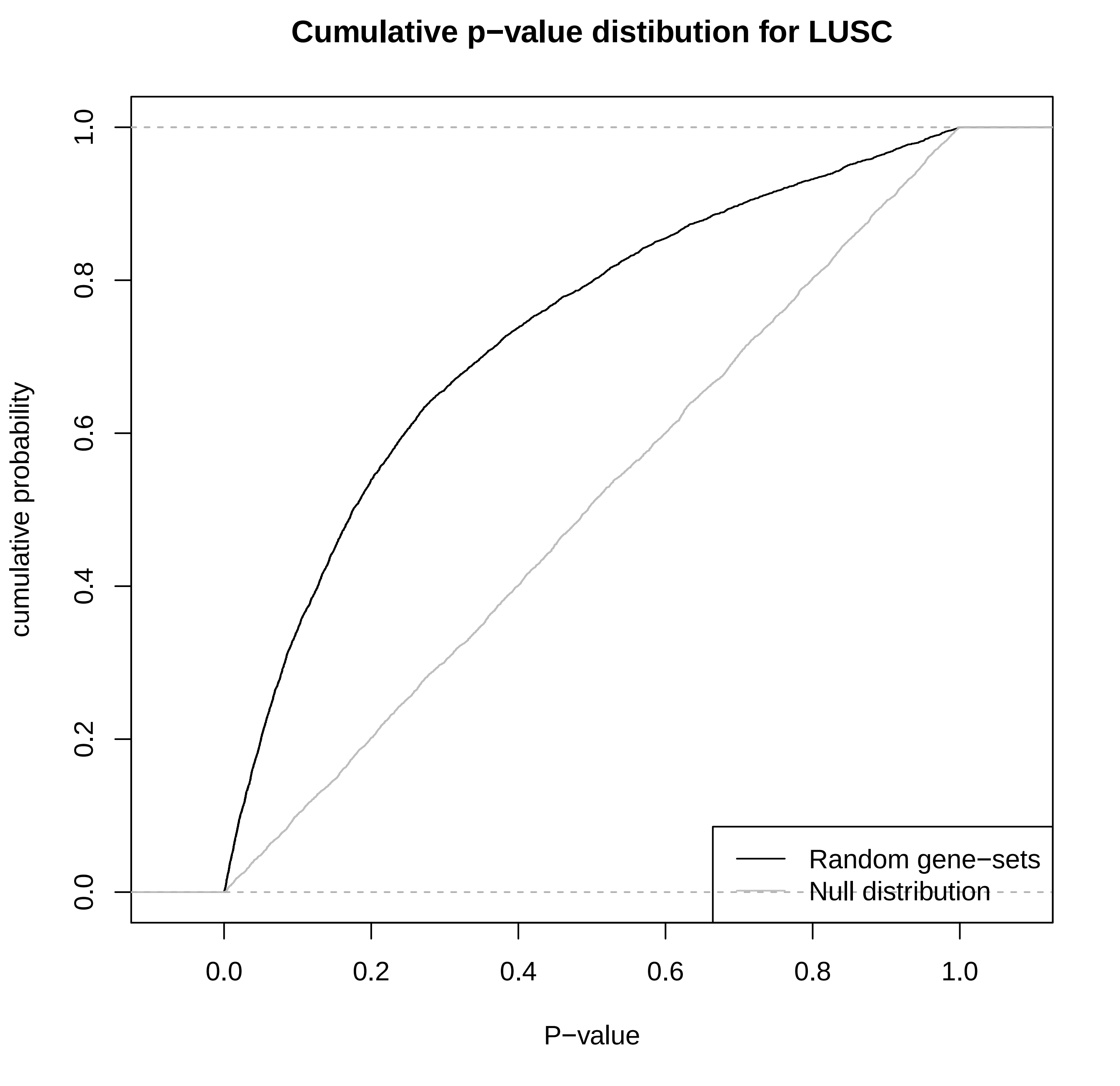
Cumulative p−value distibution for LUSC
cumulative probability
P−value
0.0
0.2
0.4
0.6
0.8
1.0
0.0
0.2
0.4
0.6
0.8
1.0
Random gene−sets
Null distribution

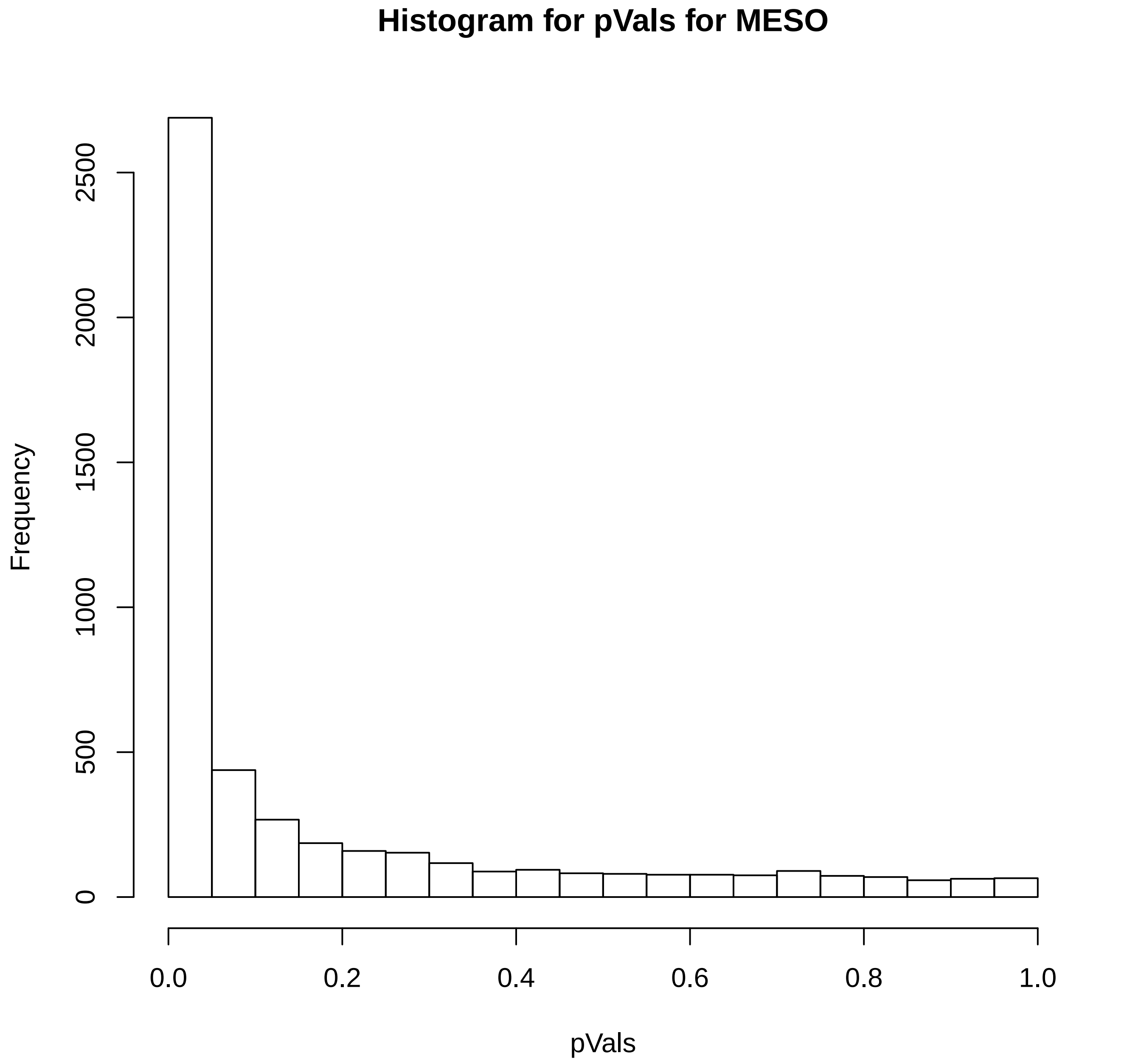
Histogram for pVals for MESO
Frequency
0
500
1000
1500
2000
2500
0.0
0.2
0.4
0.6
0.8
1.0
pVals

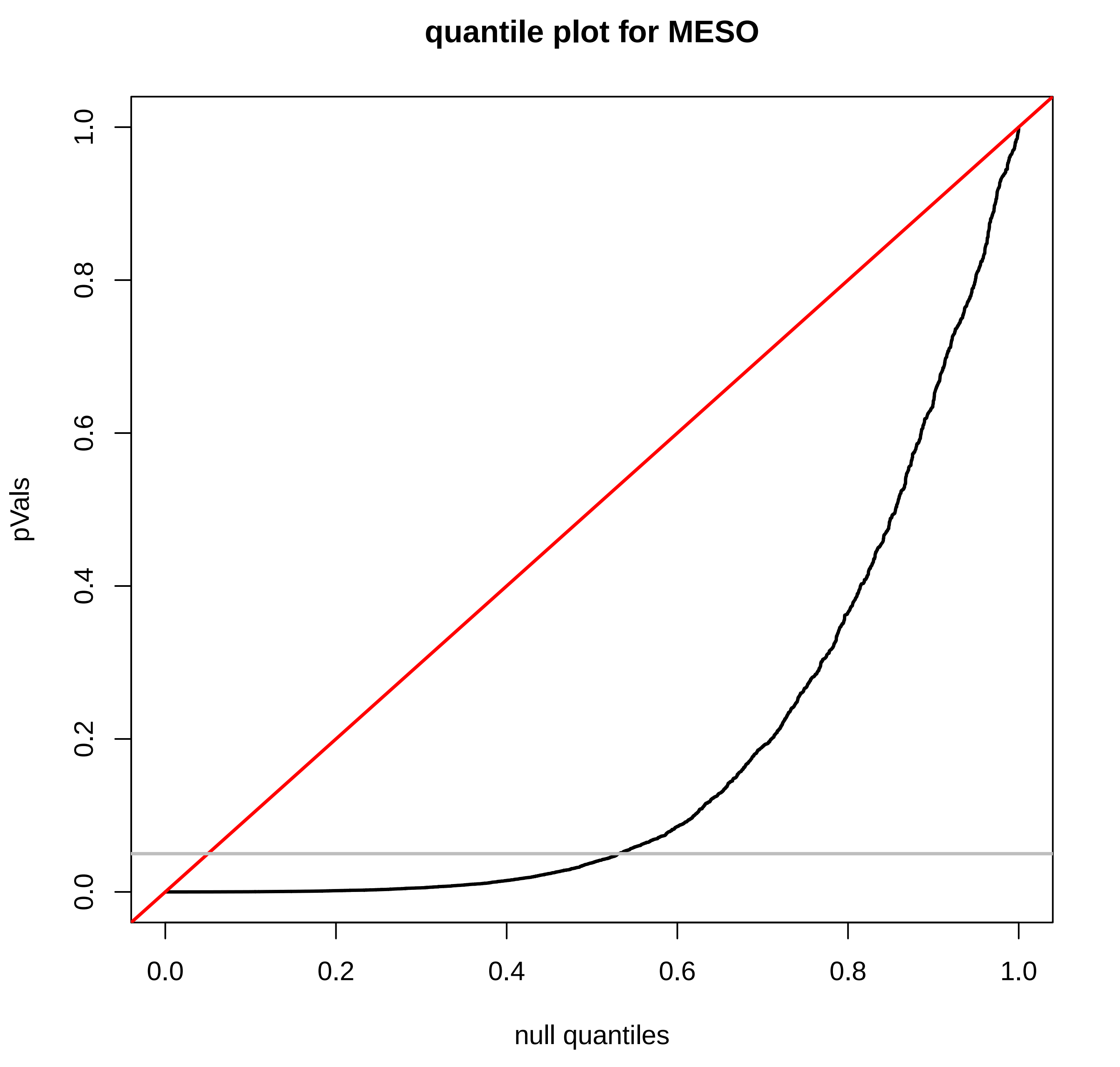
quantile plot for MESO
pVals
0.0
0.2
0.4
0.6
0.8
1.0
null quantiles
0.0
0.2
0.4
0.6
0.8
1.0

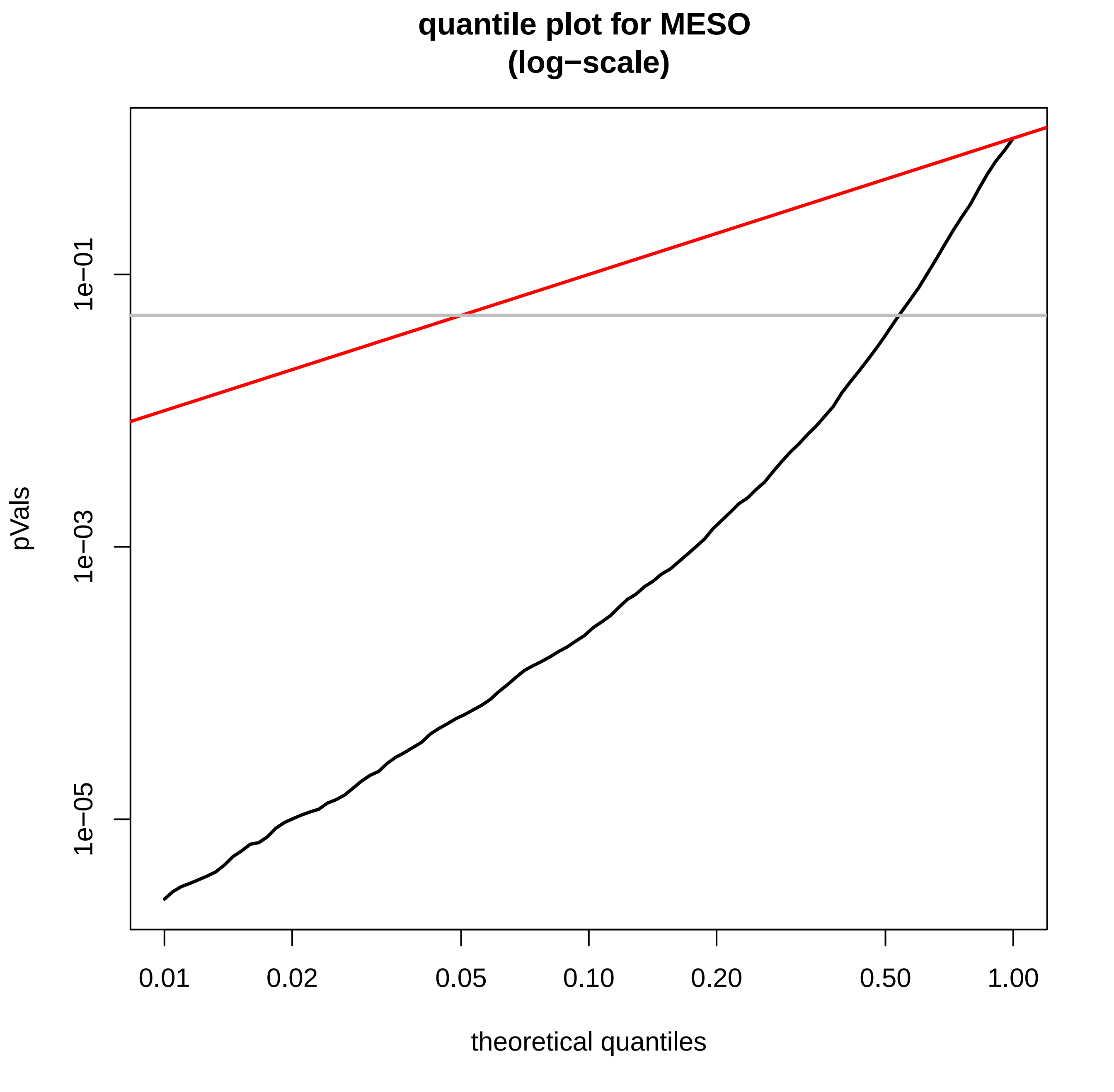

quantile plot for MESO
(log−scale)
pVals
1e−01
1e−03
1e−05
0.01
0.02
0.05
0.10
0.20
0.50
1.00
theoretical quantiles

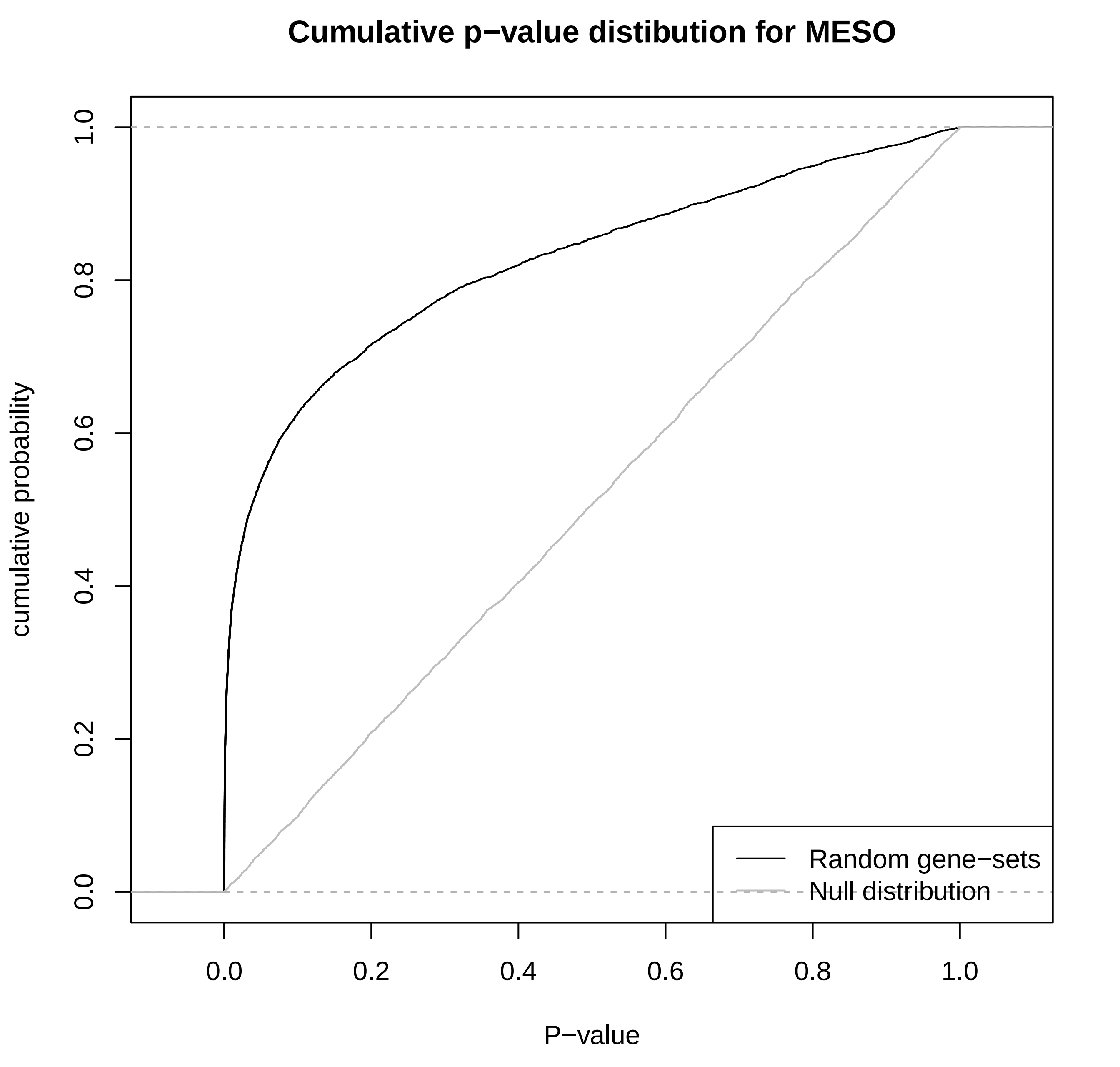

Cumulative p−value distibution for MESO
cumulative probability
0.0
0.2
0.4
0.6
0.8
1.0
P−value
0.0
0.2
0.4
0.6
0.8
1.0
Random gene−sets
Null distribution

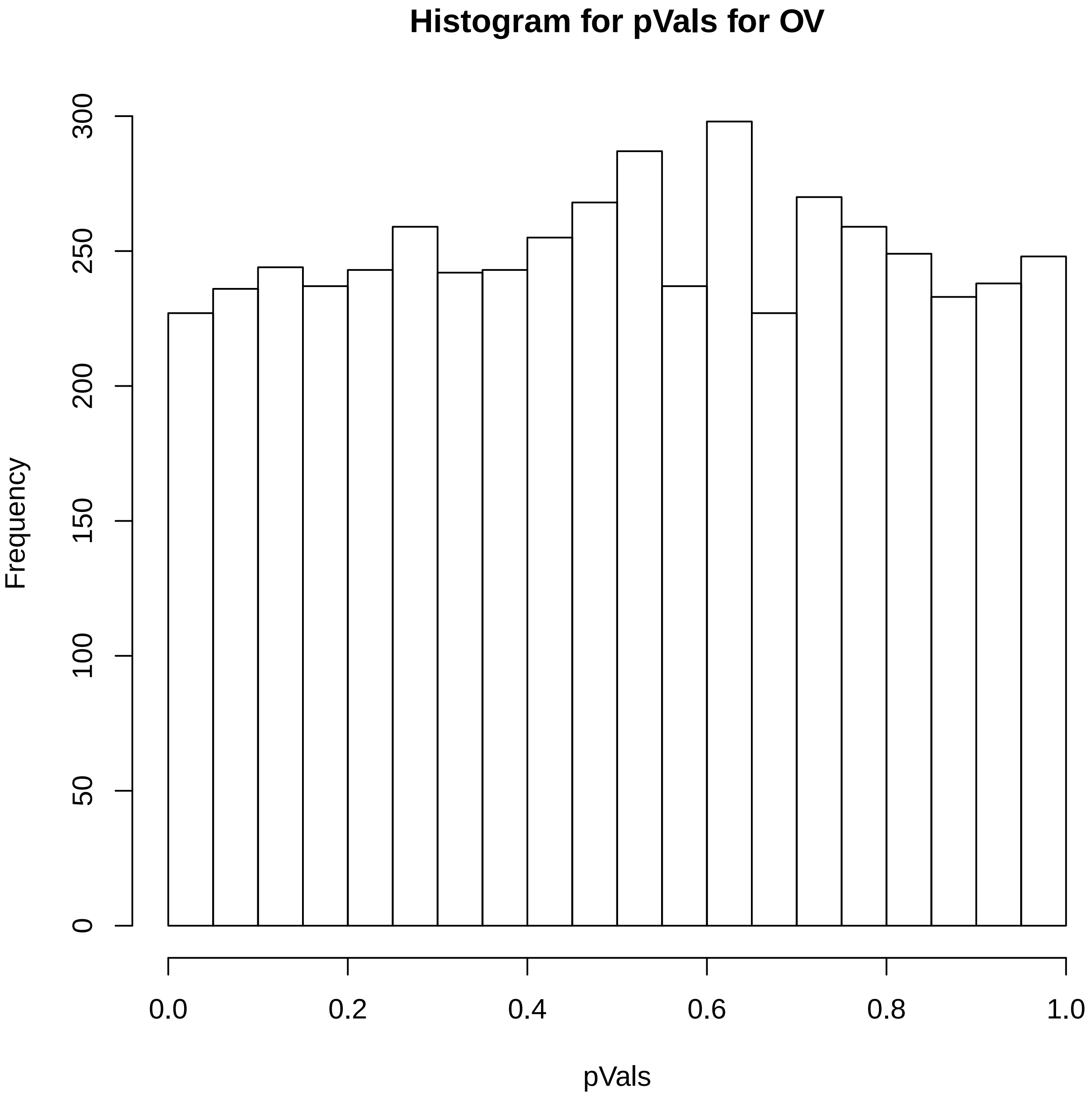
Histogram for pVals for OV
Frequency
0
50
100
150
200
250
300
0.0
0.2
0.4
0.6
0.8
1.0
pVals

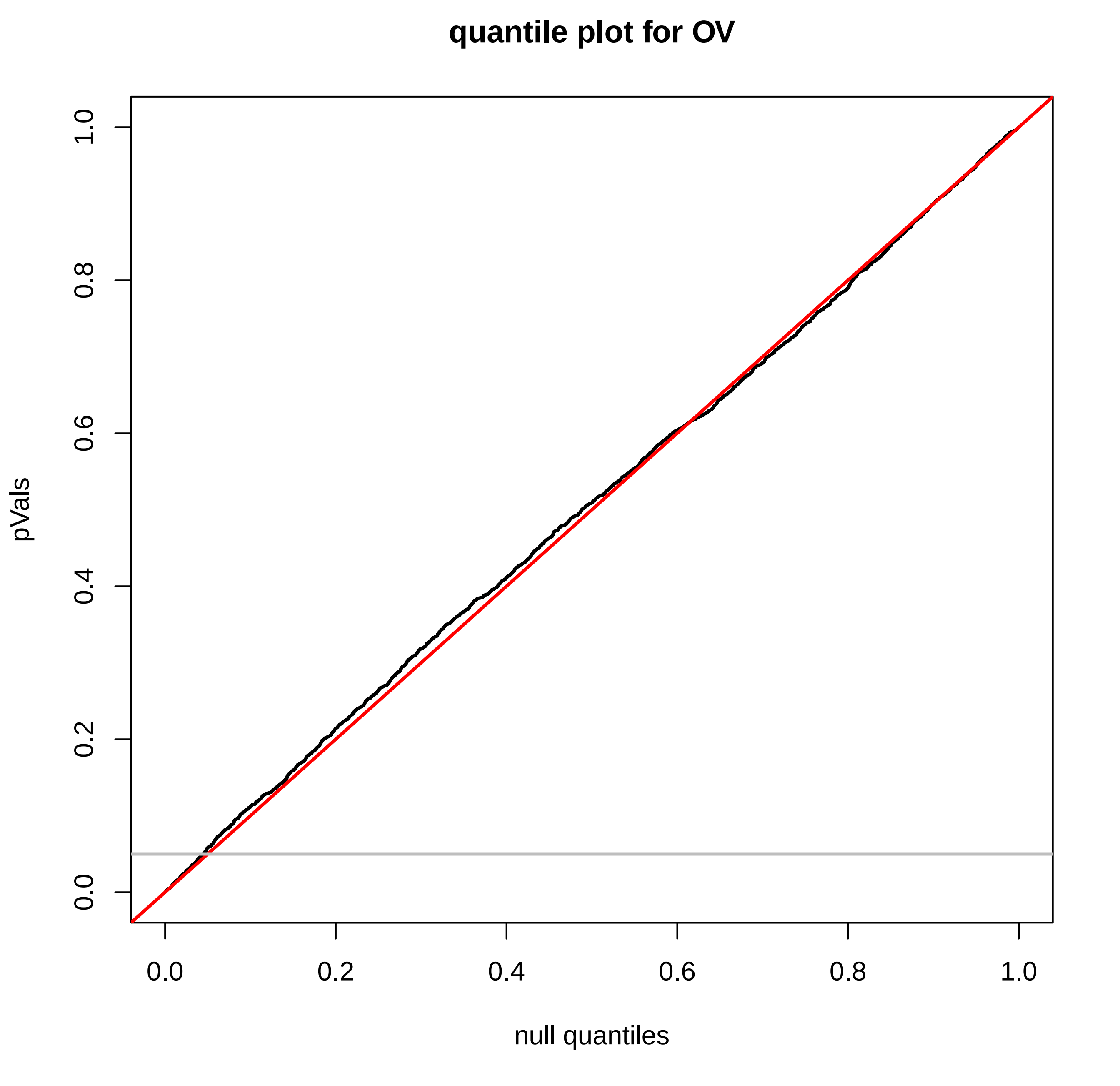
quantile plot for OV
pVals
0.0
0.2
0.4
0.6
0.8
1.0
null quantiles
0.0
0.2
0.4
0.6
0.8
1.0

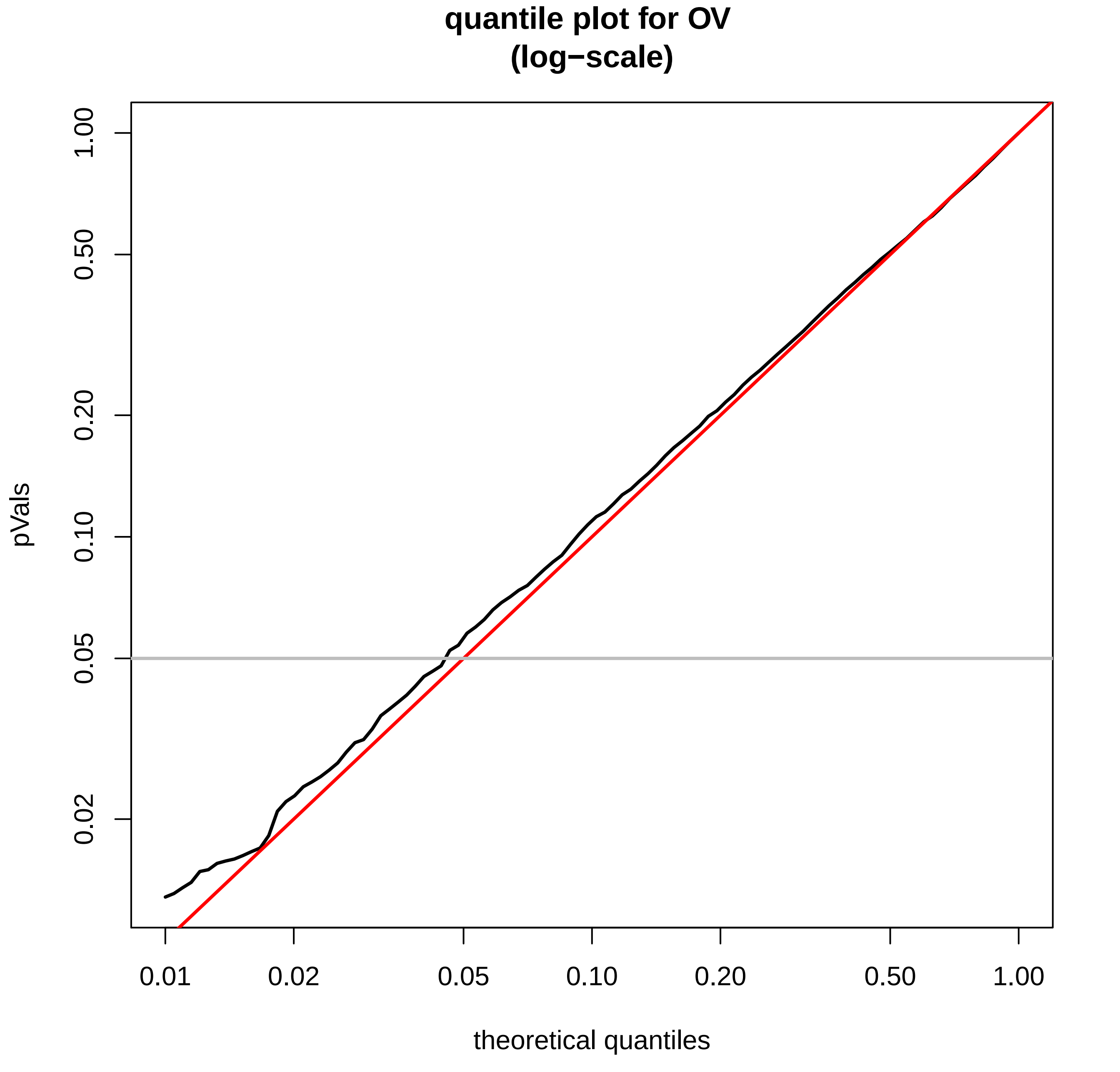
quantile plot for OV
(log−scale)
pVals
1.00
0.50
0.20
0.10
0.05
0.02
0.01
0.02
0.05
0.10
0.20
0.50
1.00
theoretical quantiles

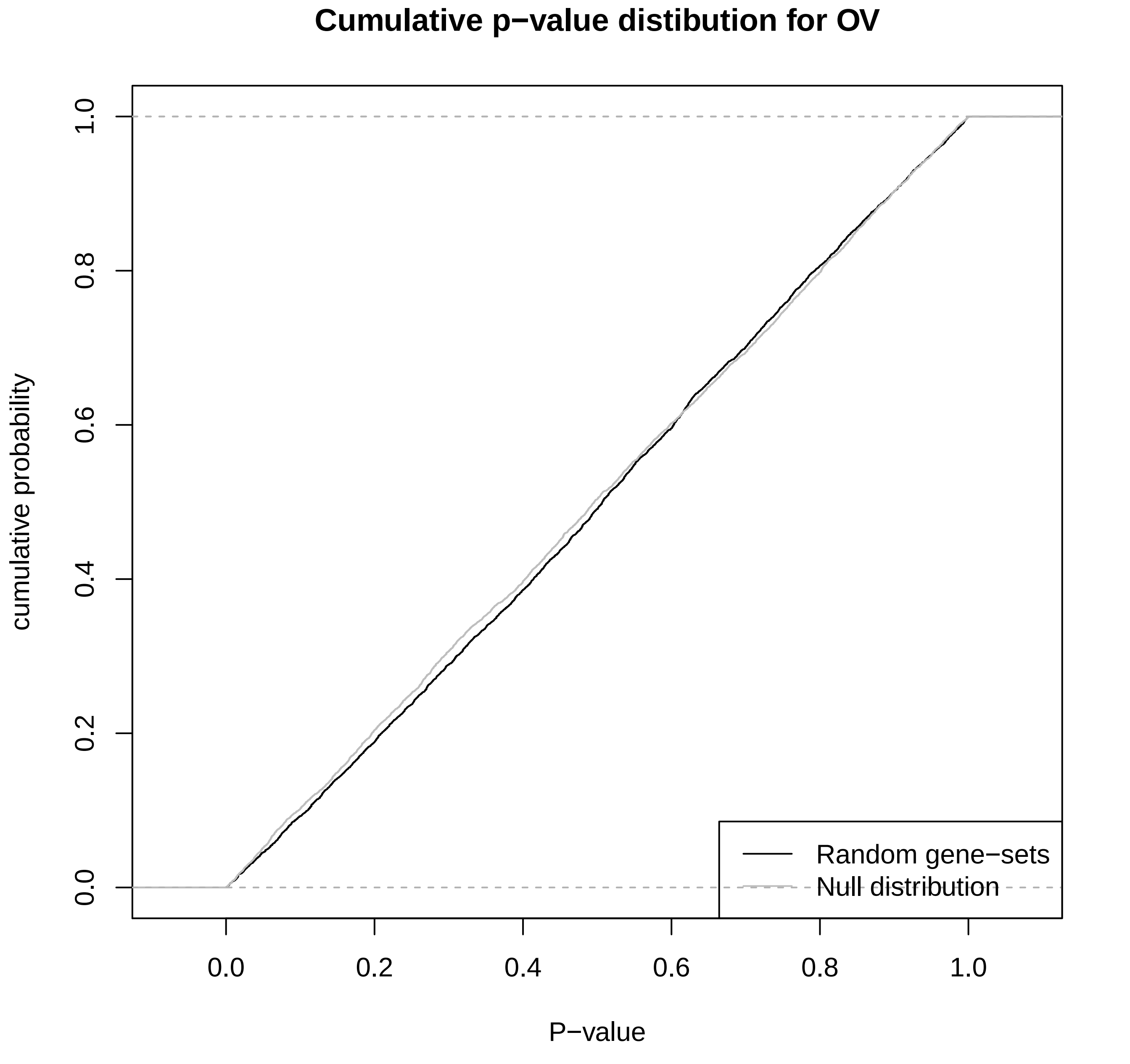
Cumulative p−value distibution for OV
cumulative probability
0.0
0.2
0.4
0.6
0.8
1.0
P−value
Random gene−sets
Null distribution

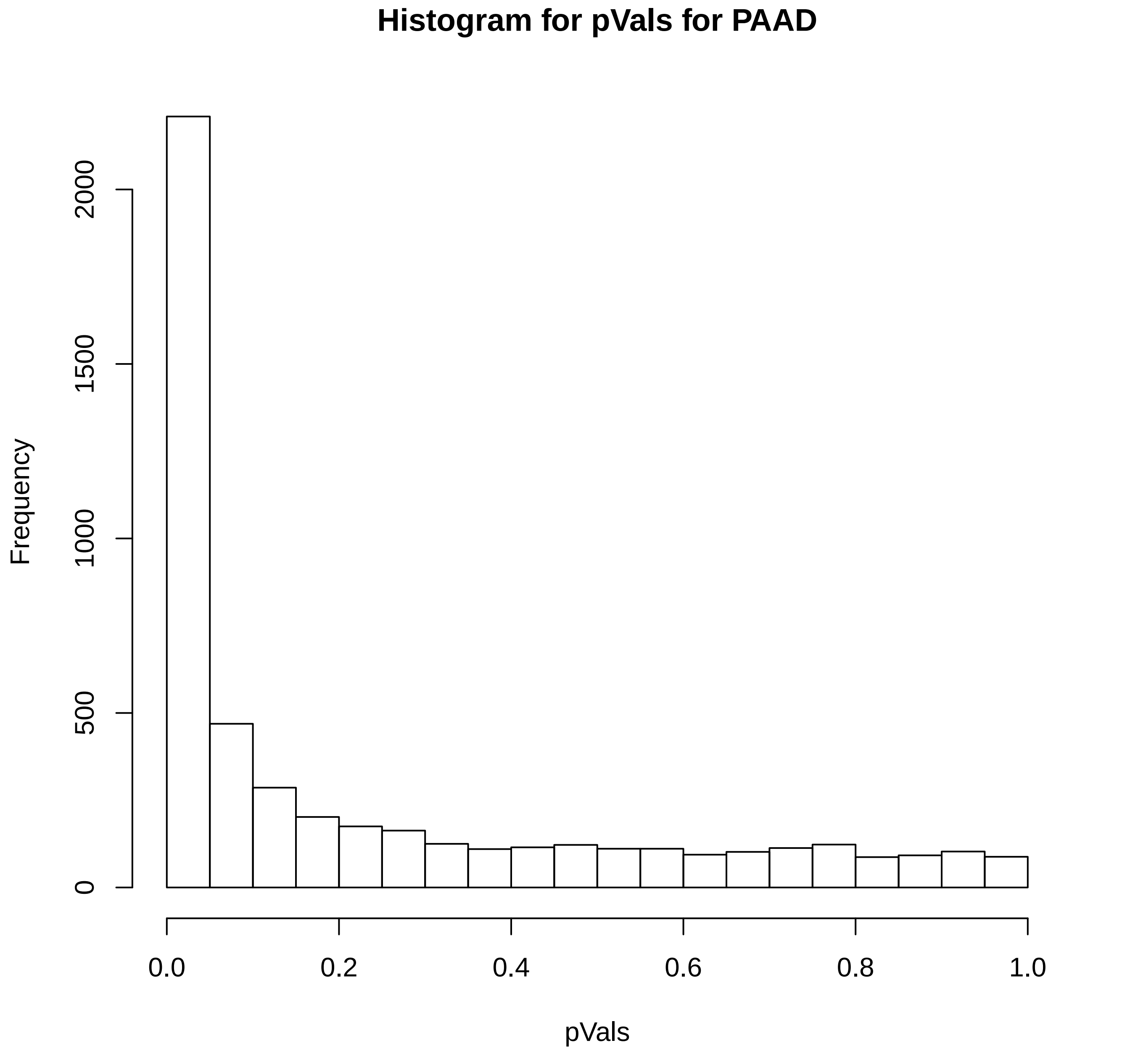
Histogram for pVals for PAAD
Frequency
0
500
1000
1500
2000
0.0
0.2
0.4
0.6
0.8
1.0
pVals

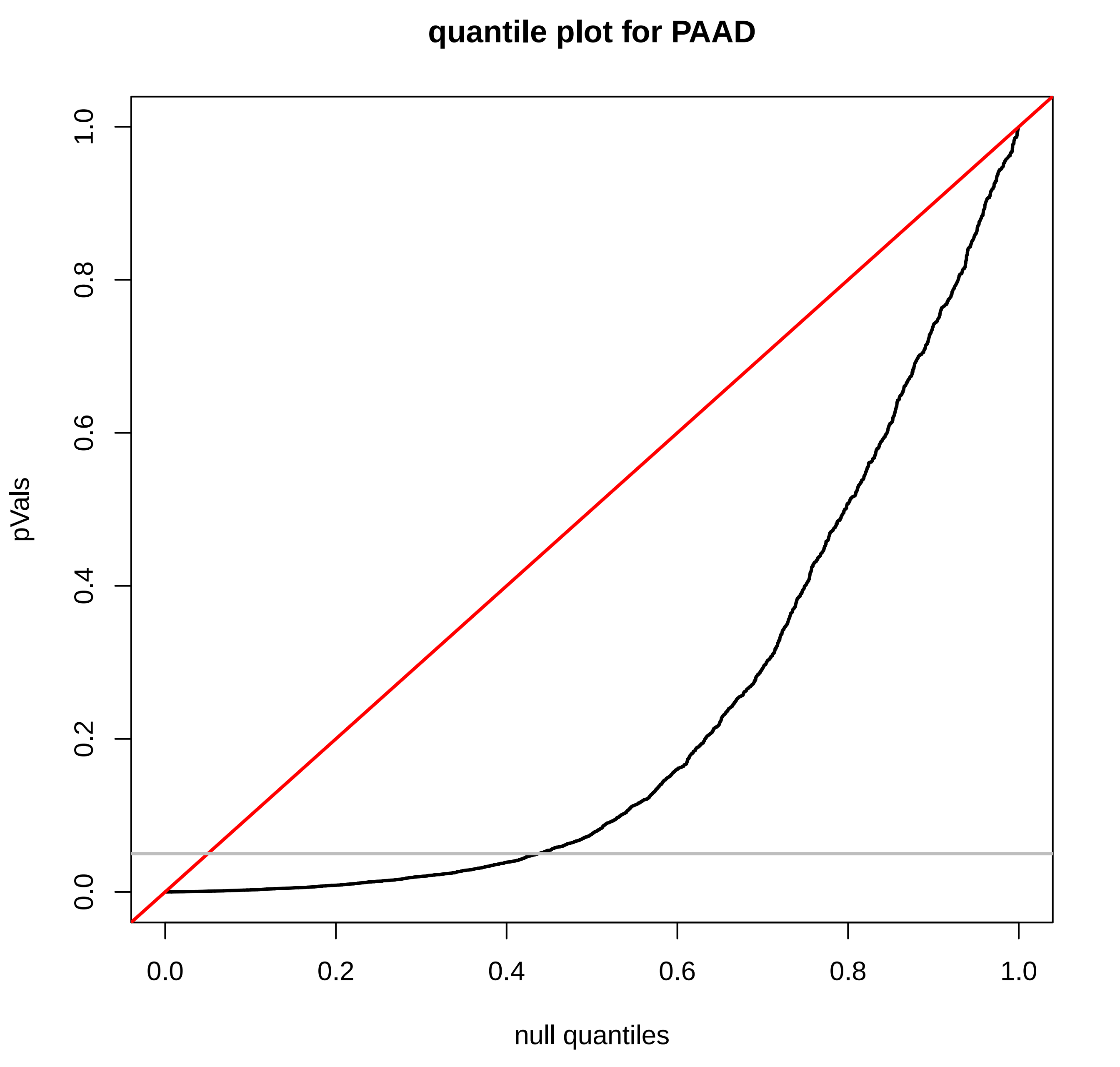
quantile plot for PAAD
pVals
null quantiles
0.0
0.2
0.4
0.6
0.8
1.0

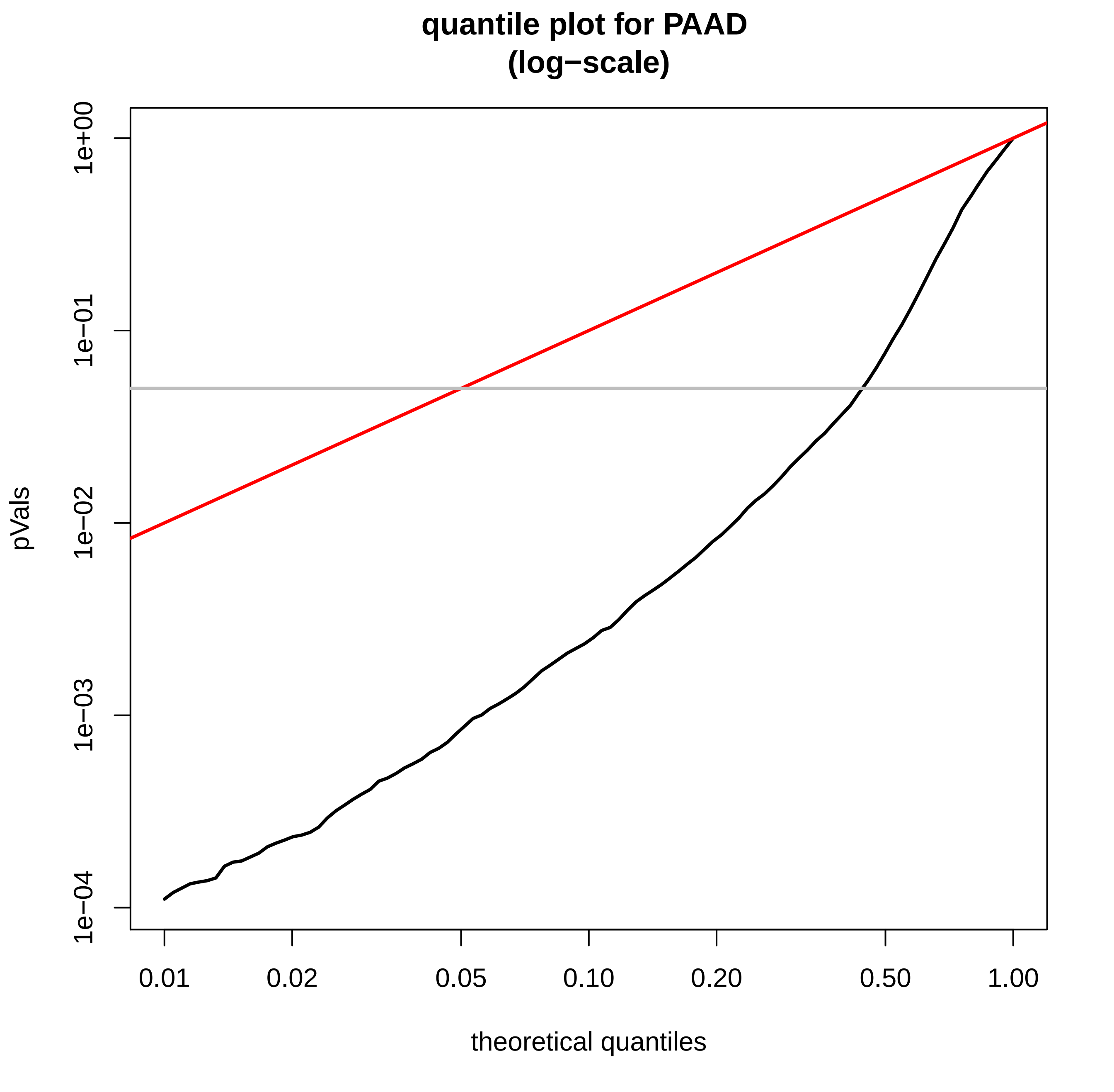
quantile plot for PAAD
(log−scale)
1e+00
1e−01
1e−02
1e−03
1e−04
pVals
0.01
0.02
0.05
0.10
0.20
0.50
1.00
theoretical quantiles

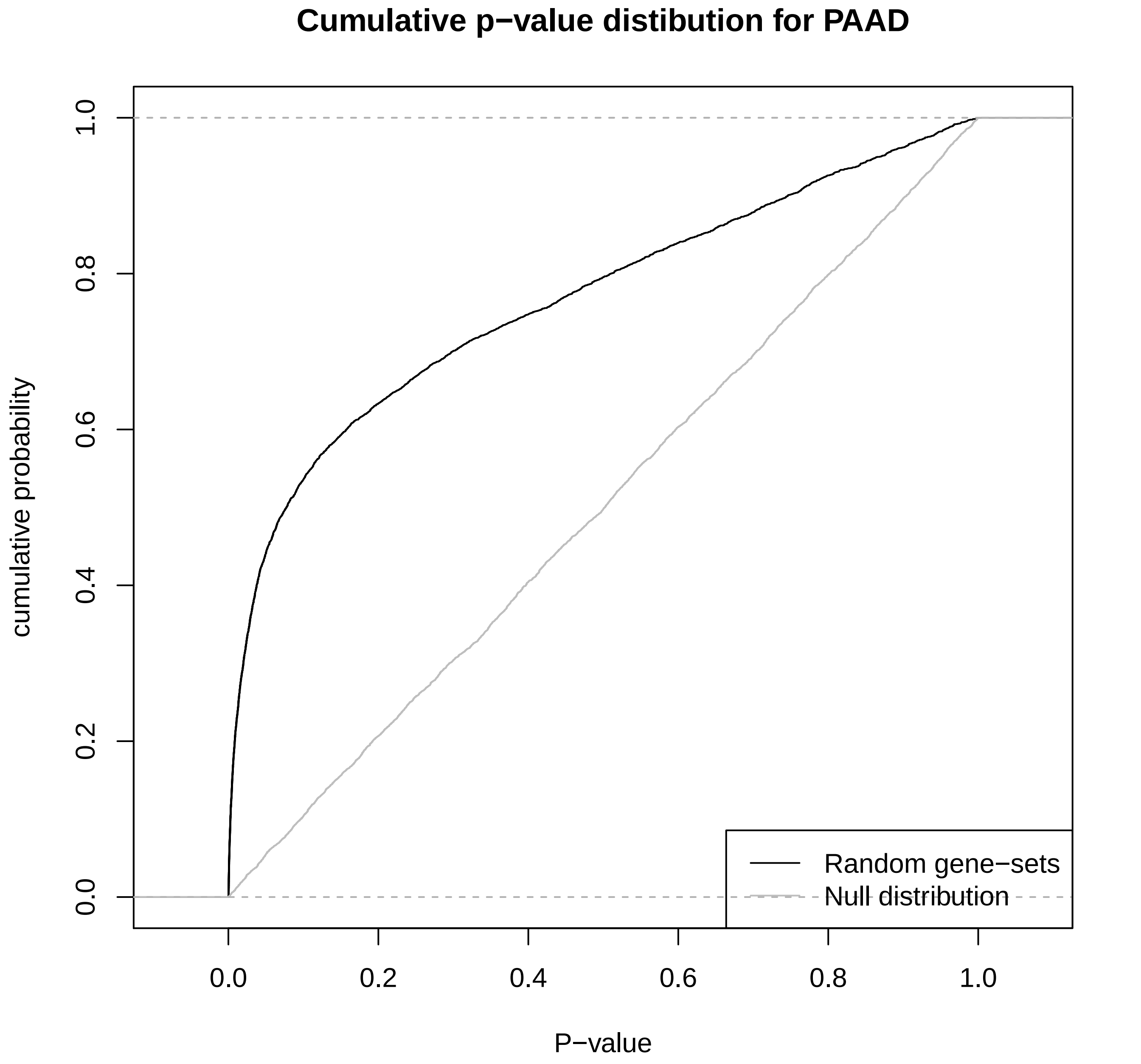
Cumulative p−value distibution for PAAD
cumulative probability
0.0
0.2
0.4
0.6
0.8
1.0
P−value
0.0
0.2
0.4
0.6
0.8
1.0
Random gene−sets
Null distribution

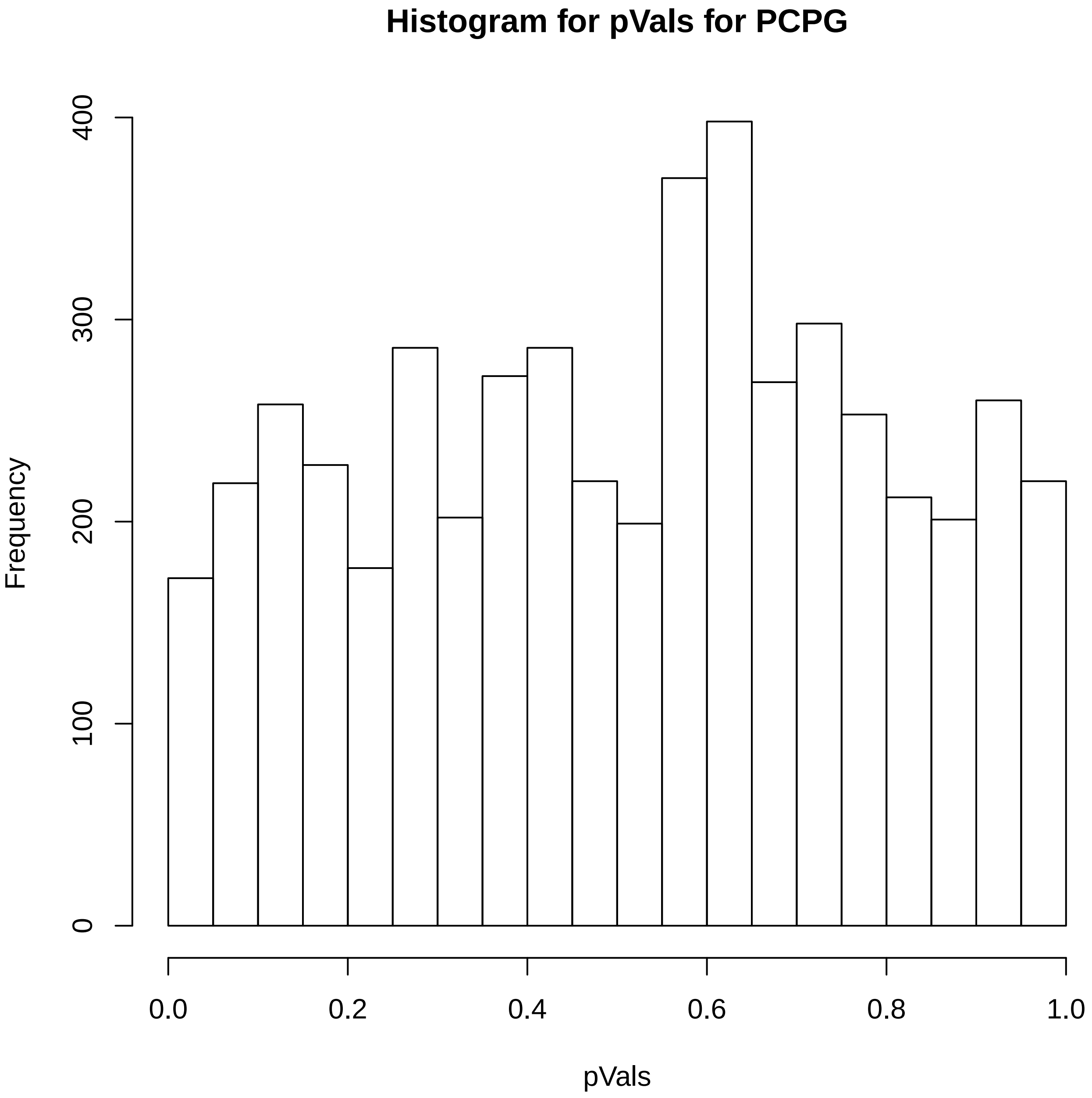
Histogram for pVals for PCPG
Frequency
0
100
200
300
400
0.0
0.2
0.4
0.6
0.8
1.0
pVals

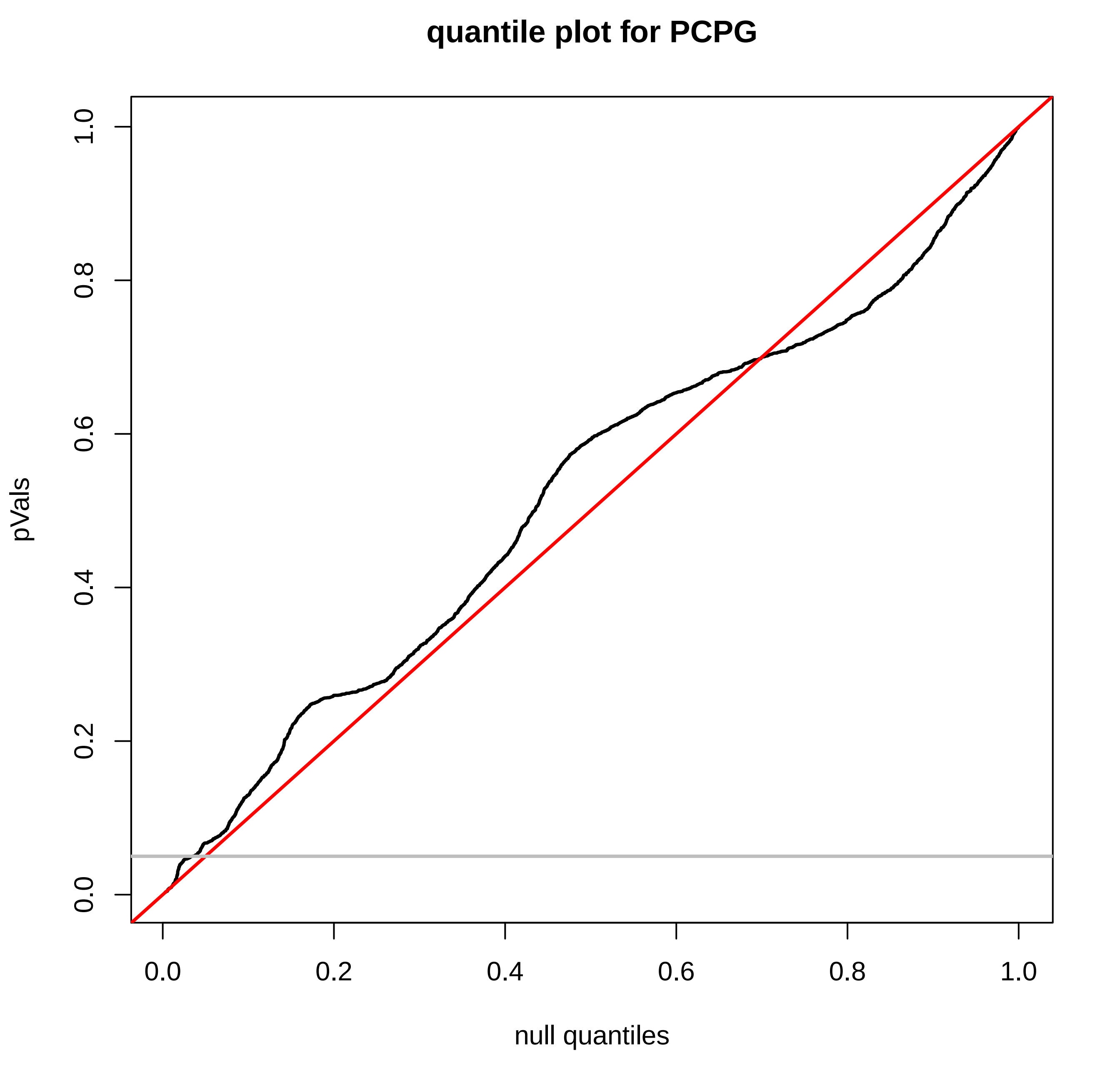

quantile plot for PCPG
pVals
null quantiles
0.0
0.2
0.4
0.6
0.8
1.0

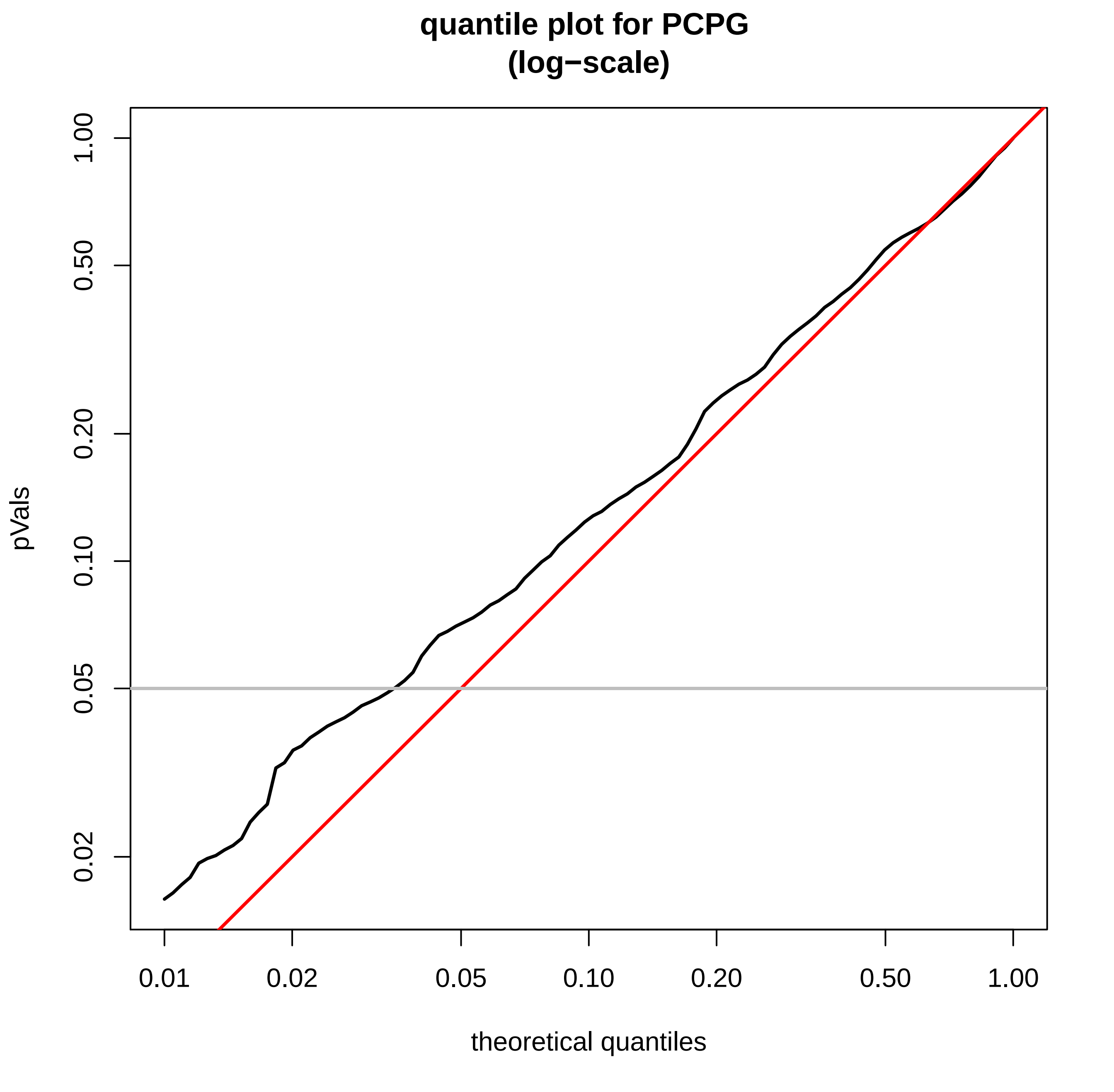
quantile plot for PCPG
(log−scale)
pVals
1.00
0.50
0.20
0.10
0.05
0.02
0.01
0.02
0.05
0.10
0.20
0.50
1.00
theoretical quantiles

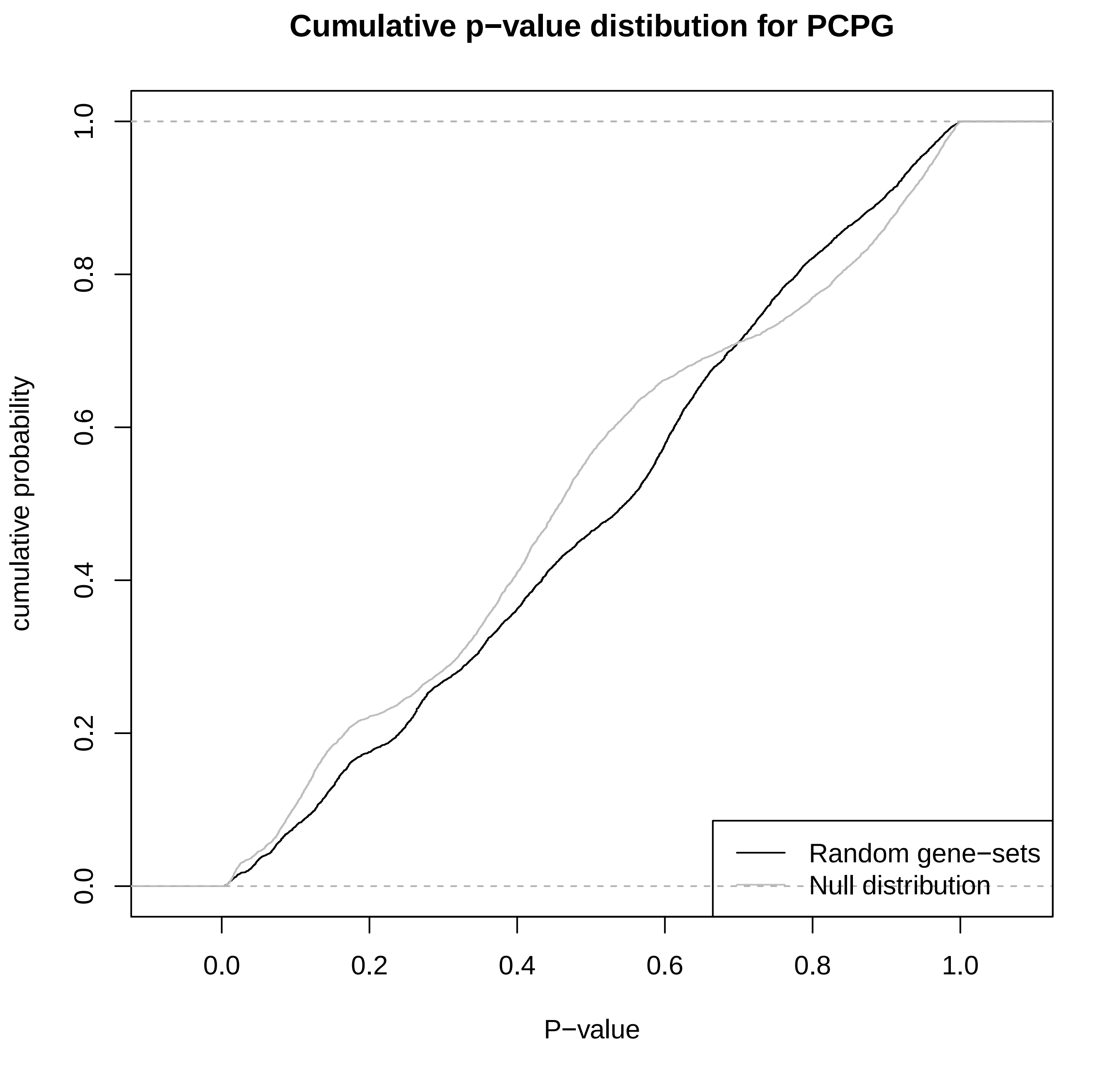

Cumulative p−value distibution for PCPG
cumulative probability
P−value
0.0
0.2
0.4
0.6
0.8
1.0
Random gene−sets
Null distribution

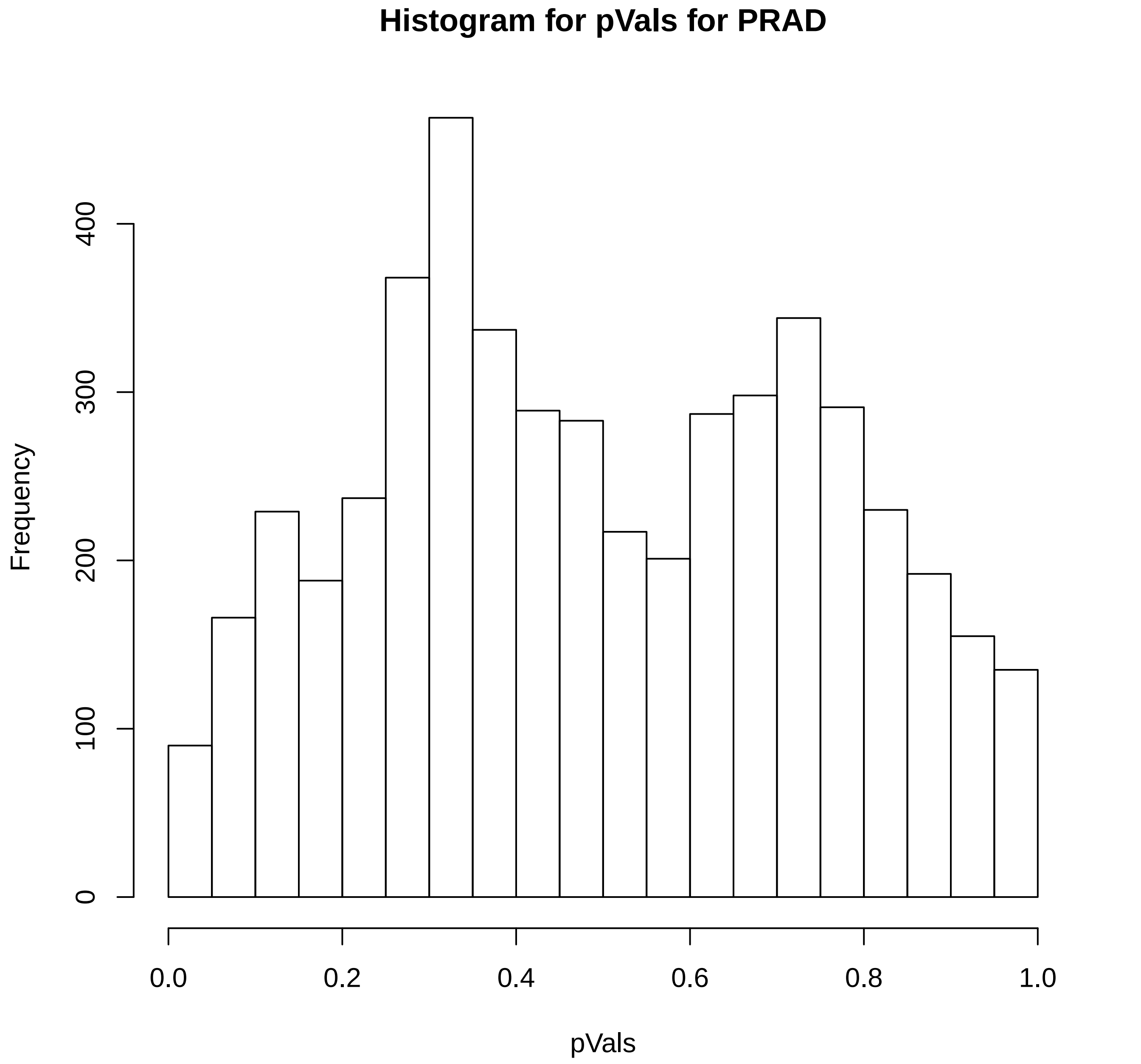
Histogram for pVals for PRAD
Frequency
0
100
200
300
400
0.0
0.2
0.4
0.6
0.8
1.0
pVals

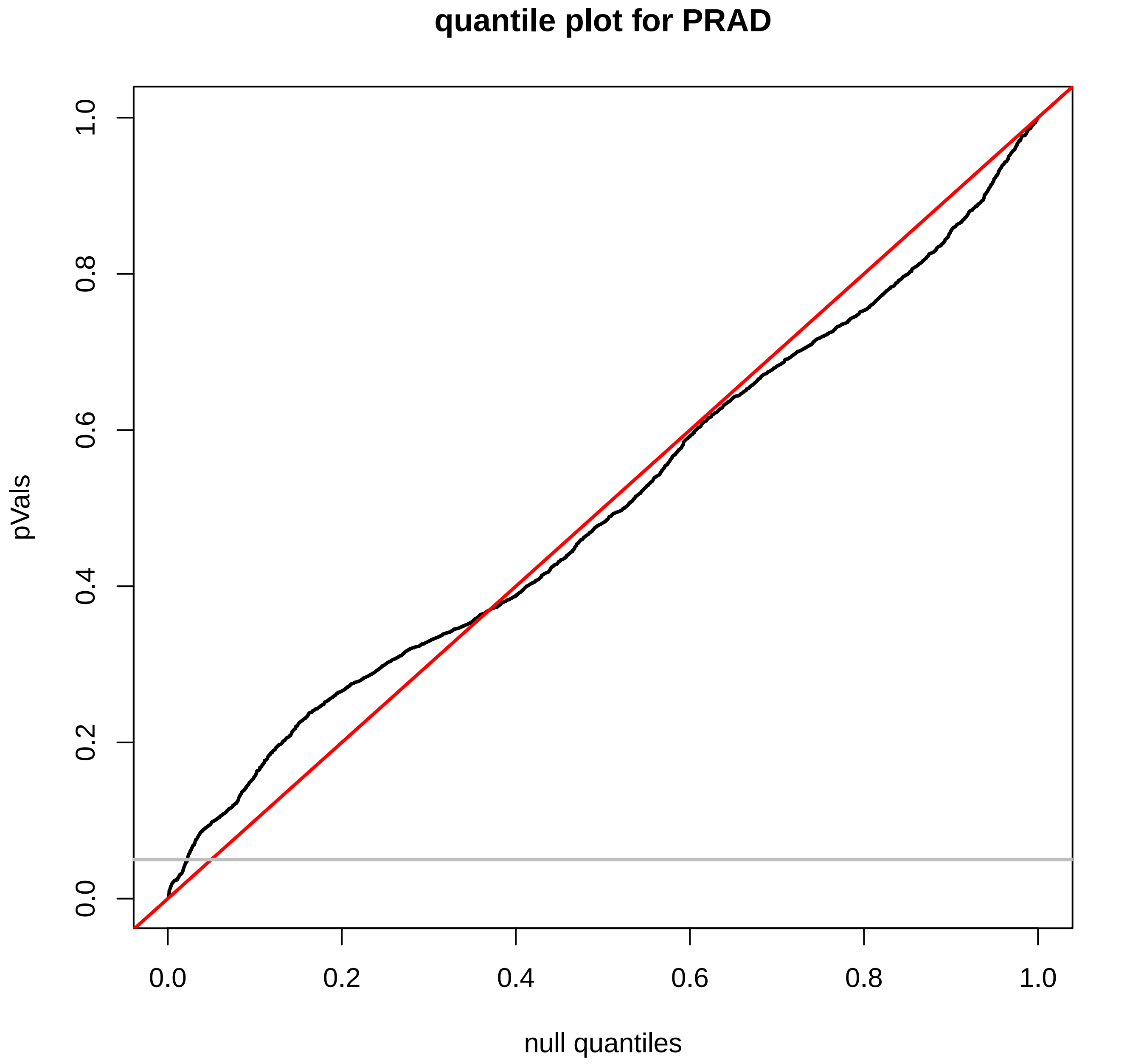
quantile plot for PRAD
pVals
null quantiles
0.0
0.2
0.4
0.6
0.8
1.0

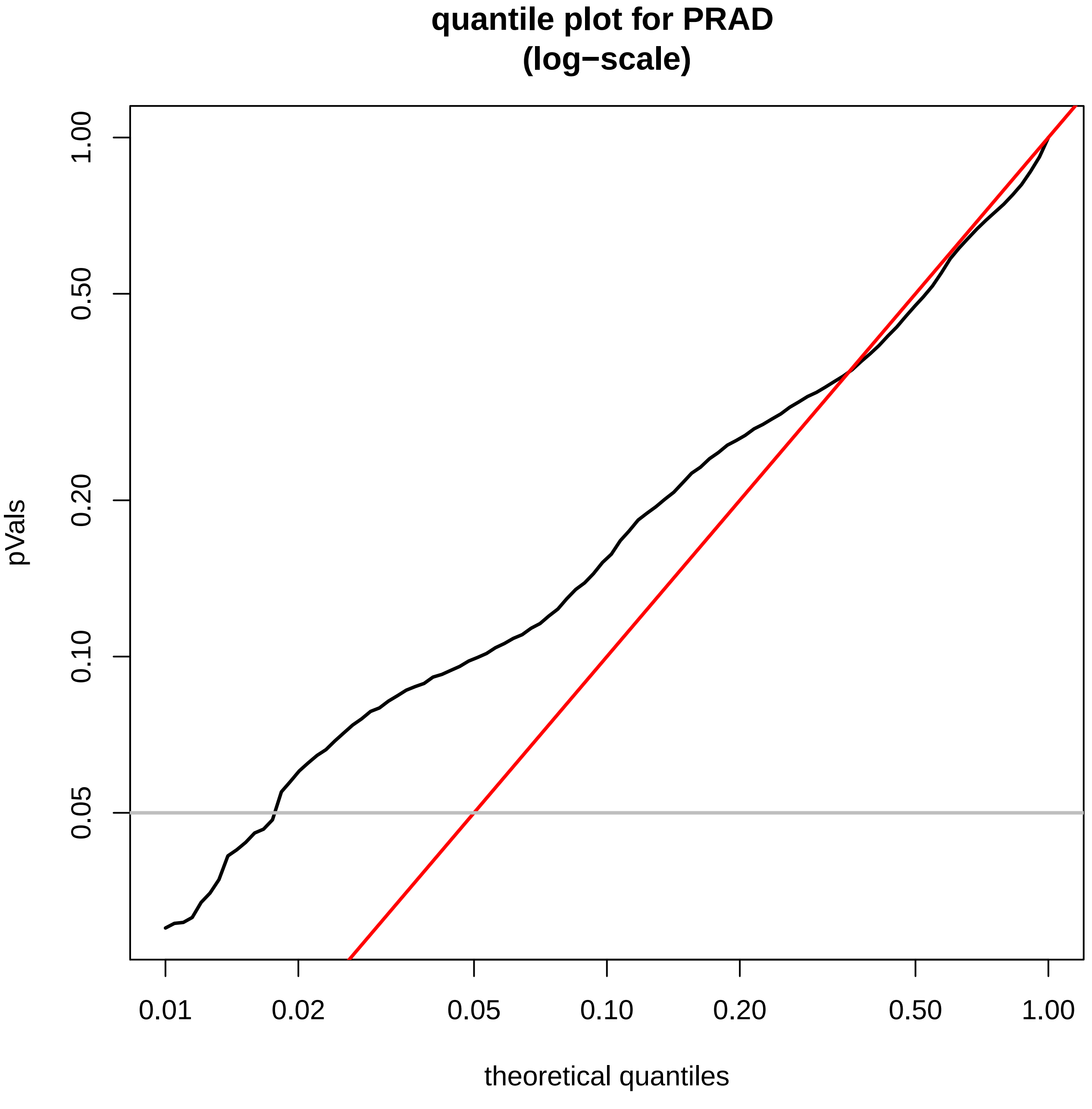
quantile plot for PRAD
(log−scale)
pVals
1.00
0.50
0.20
0.10
0.05
0.01
0.02
0.05
0.10
0.20
0.50
1.00
theoretical quantiles

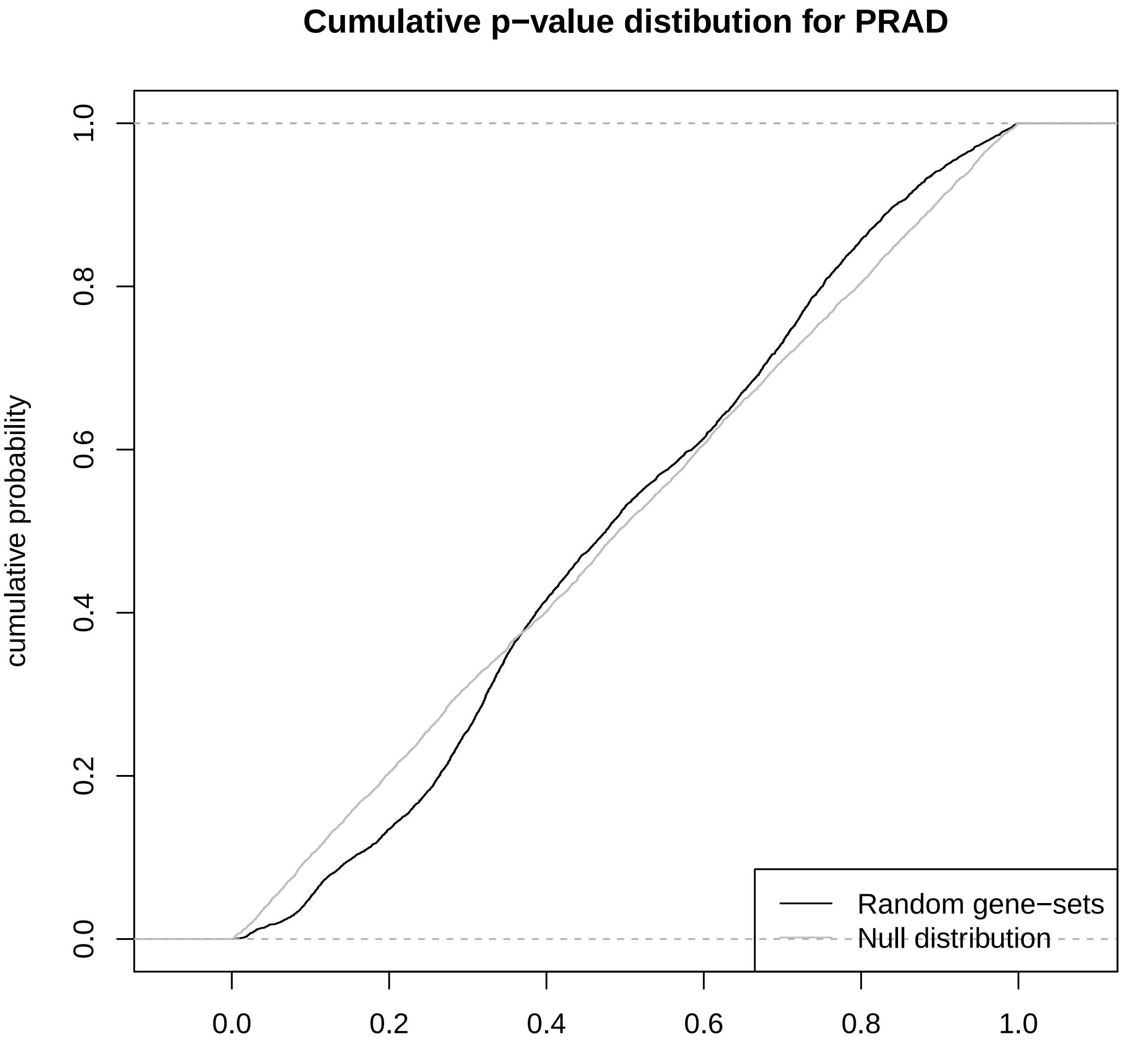
Cumulative p−value distibution for PRAD
cumulative probability
P−value
0.0
0.2
0.4
0.6
0.8
1.0
Random gene−sets
Null distribution

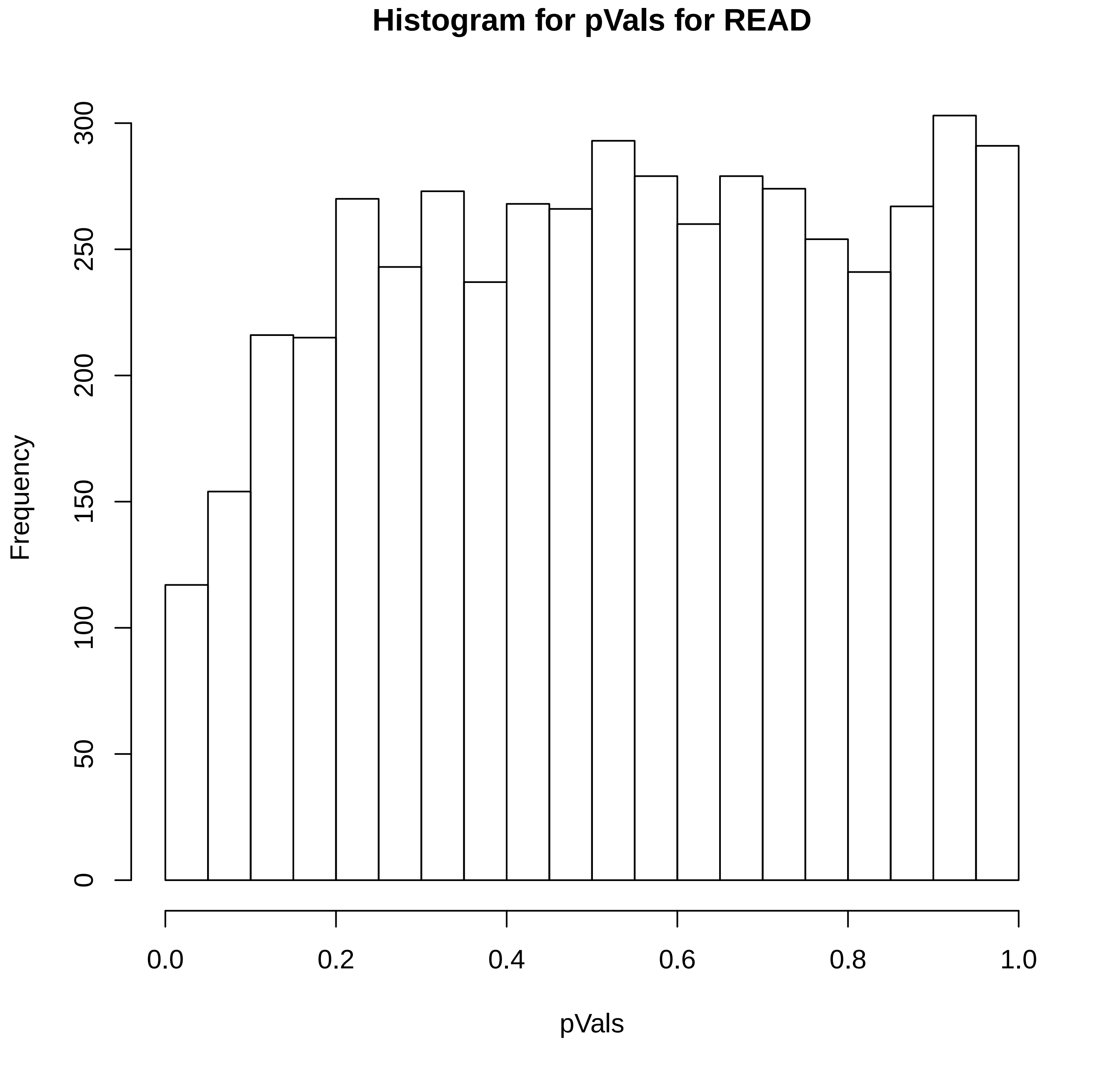
Histogram for pVals for READ
Frequency
0
50
100
150
200
250
300
0.0
0.2
0.4
0.6
0.8
1.0
pVals

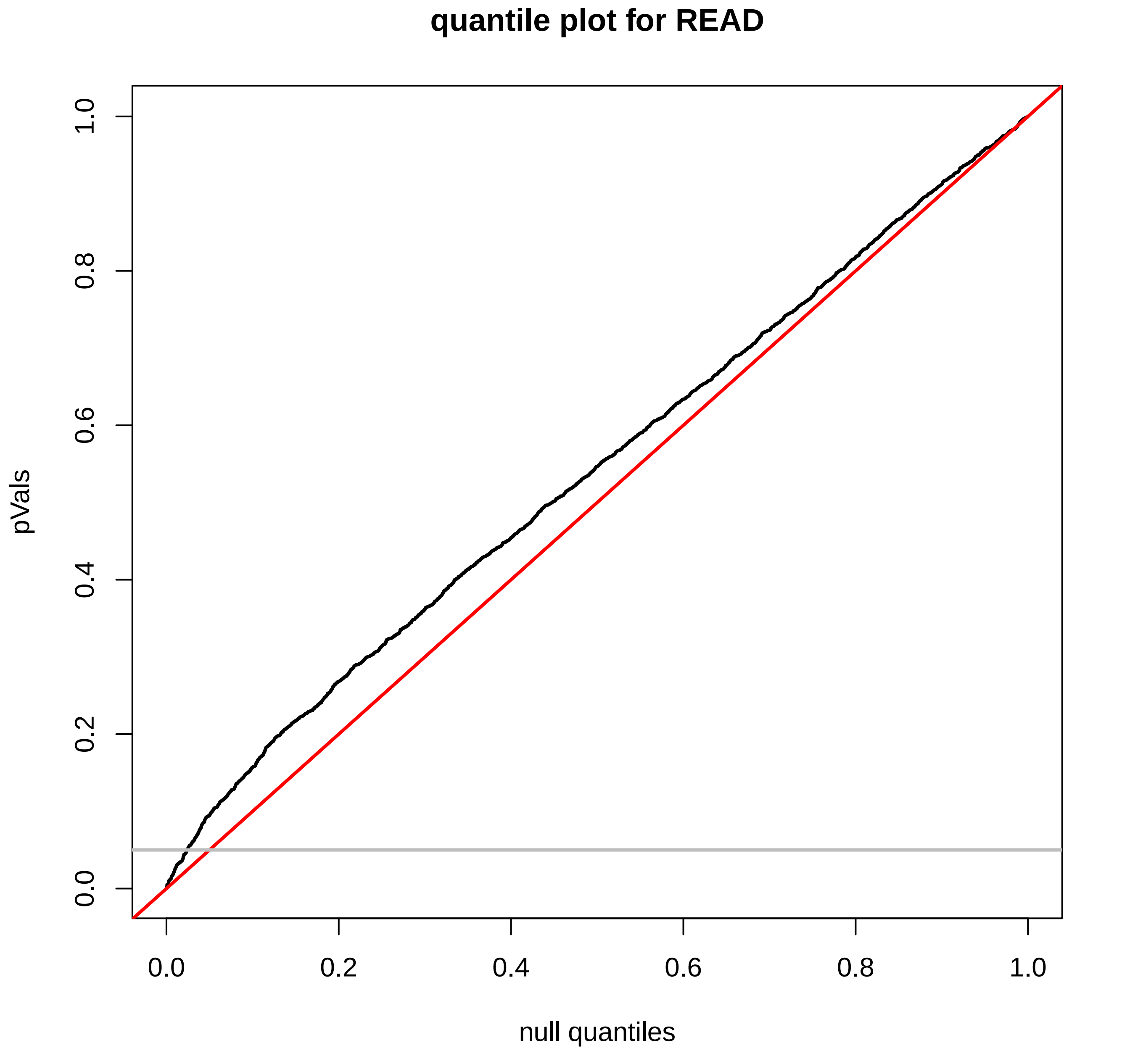
quantile plot for READ
pVals
null quantiles
0.0
0.2
0.4
0.6
0.8
1.0
0.0
0.2
0.4
0.6
0.8
1.0

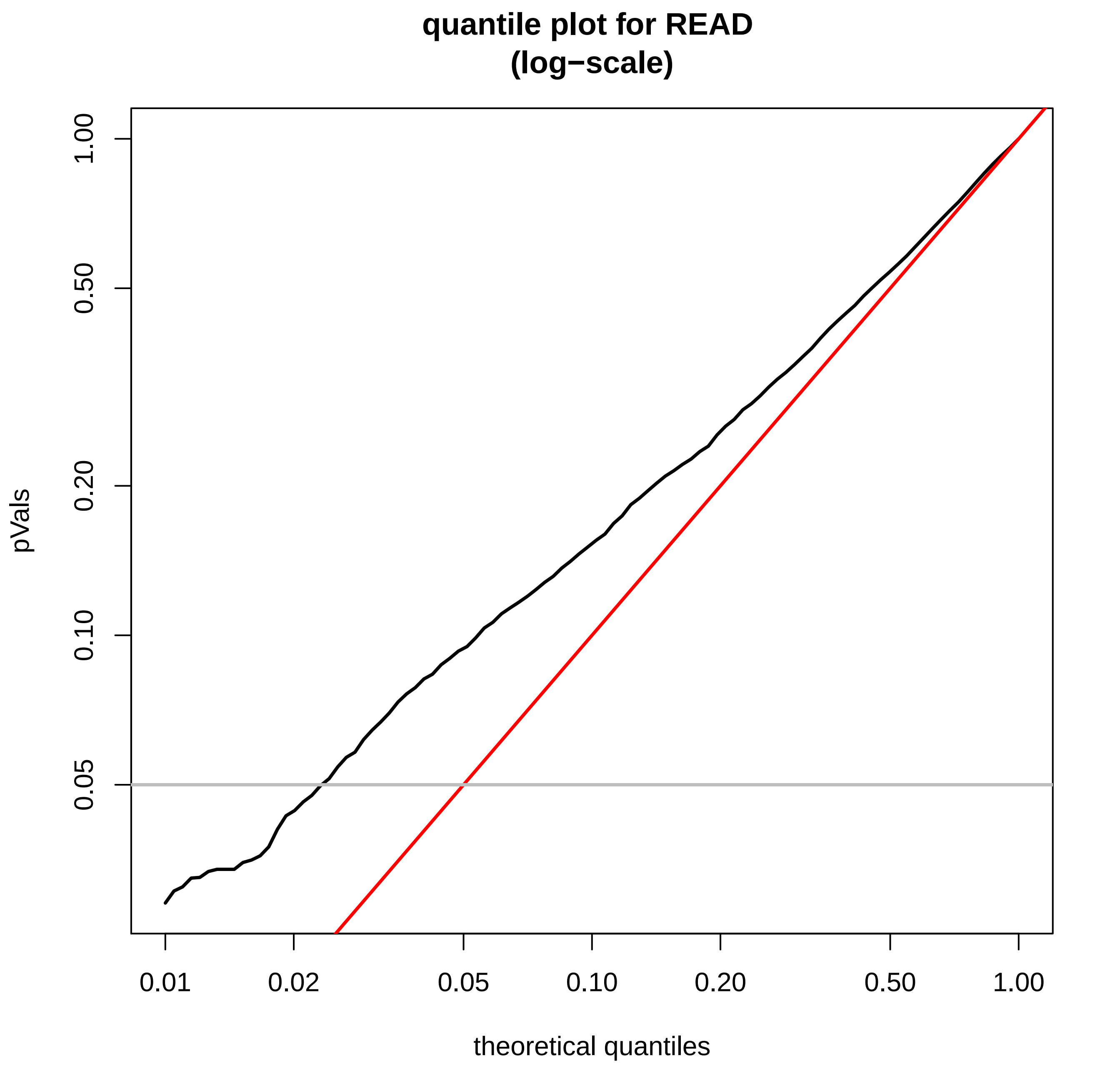
quantile plot for READ
(log−scale)
pVals
1.00
0.50
0.20
0.10
0.05
0.01
0.02
0.05
0.10
0.20
0.50
1.00
theoretical quantiles

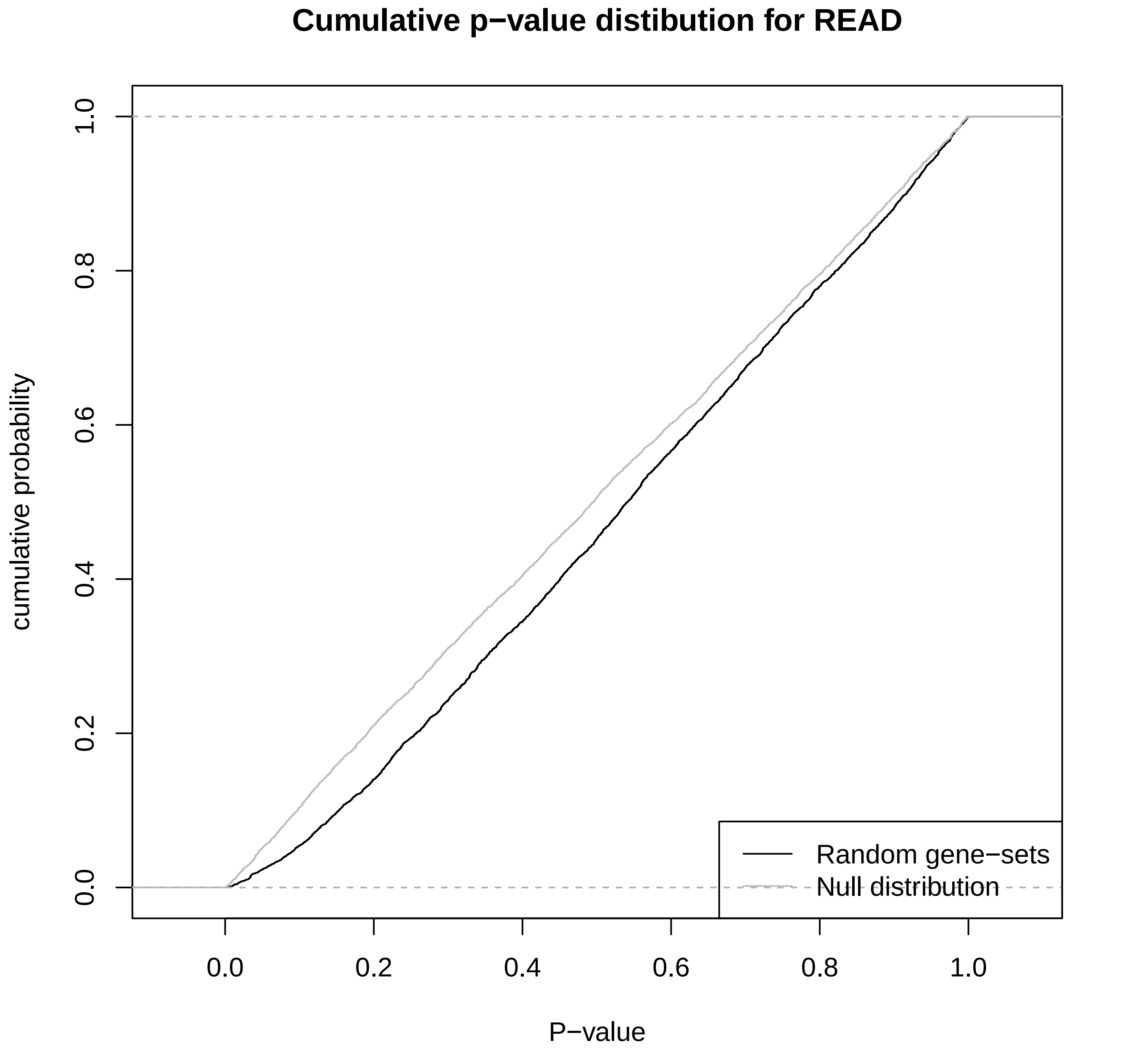

Cumulative p−value distibution for READ
cumulative probability
P−value
0.0
0.2
0.4
0.6
0.8
1.0
Random gene−sets
Null distribution

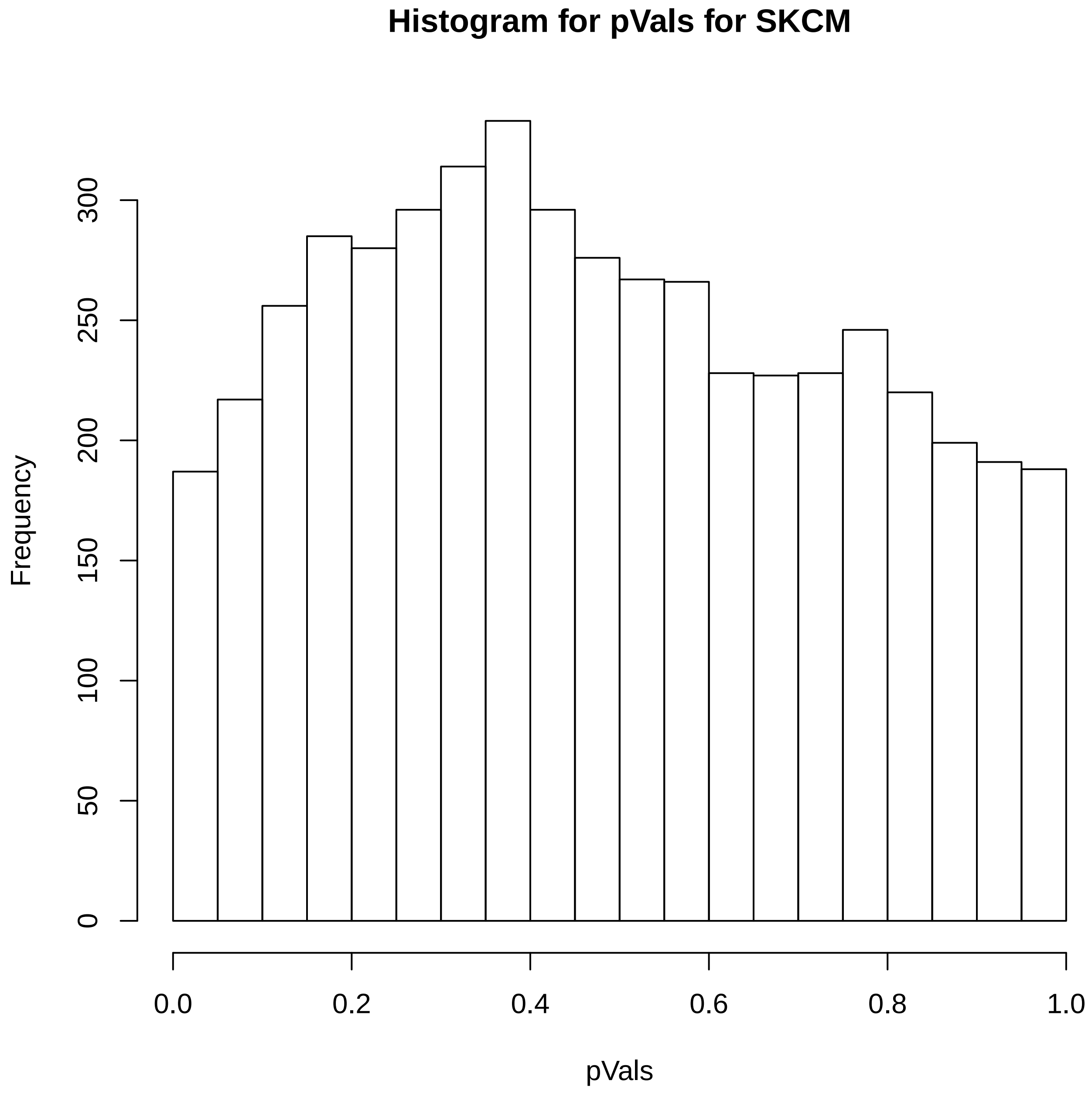
Histogram for pVals for SKCM
Frequency
0
50
100
150
200
250
300
0.0
0.2
0.4
0.6
0.8
1.0
pVals

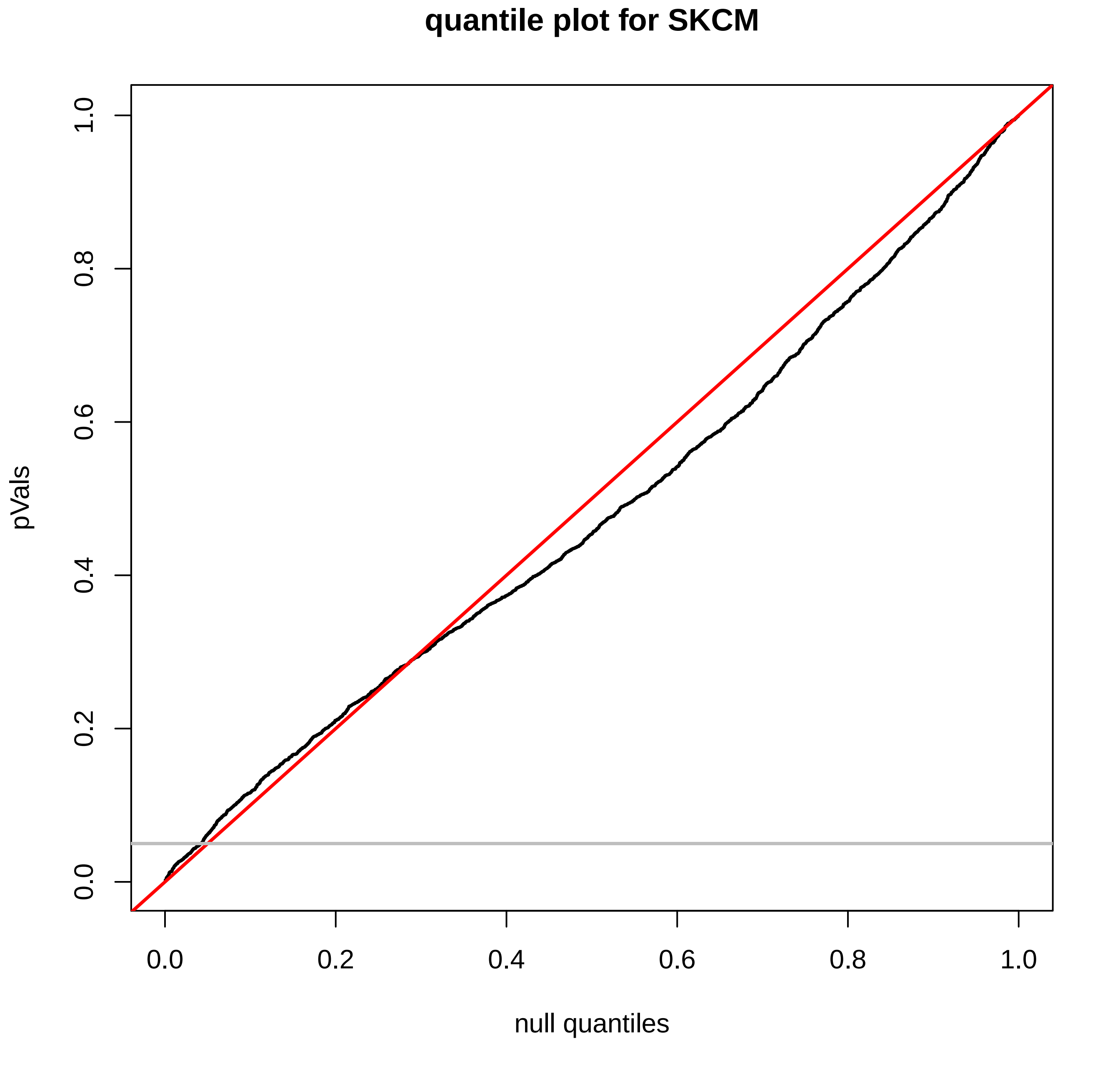
quantile plot for SKCM
pVals
null quantiles
0.0
0.2
0.4
0.6
0.8
1.0
0.0
0.2
0.4
0.6
0.8
1.0

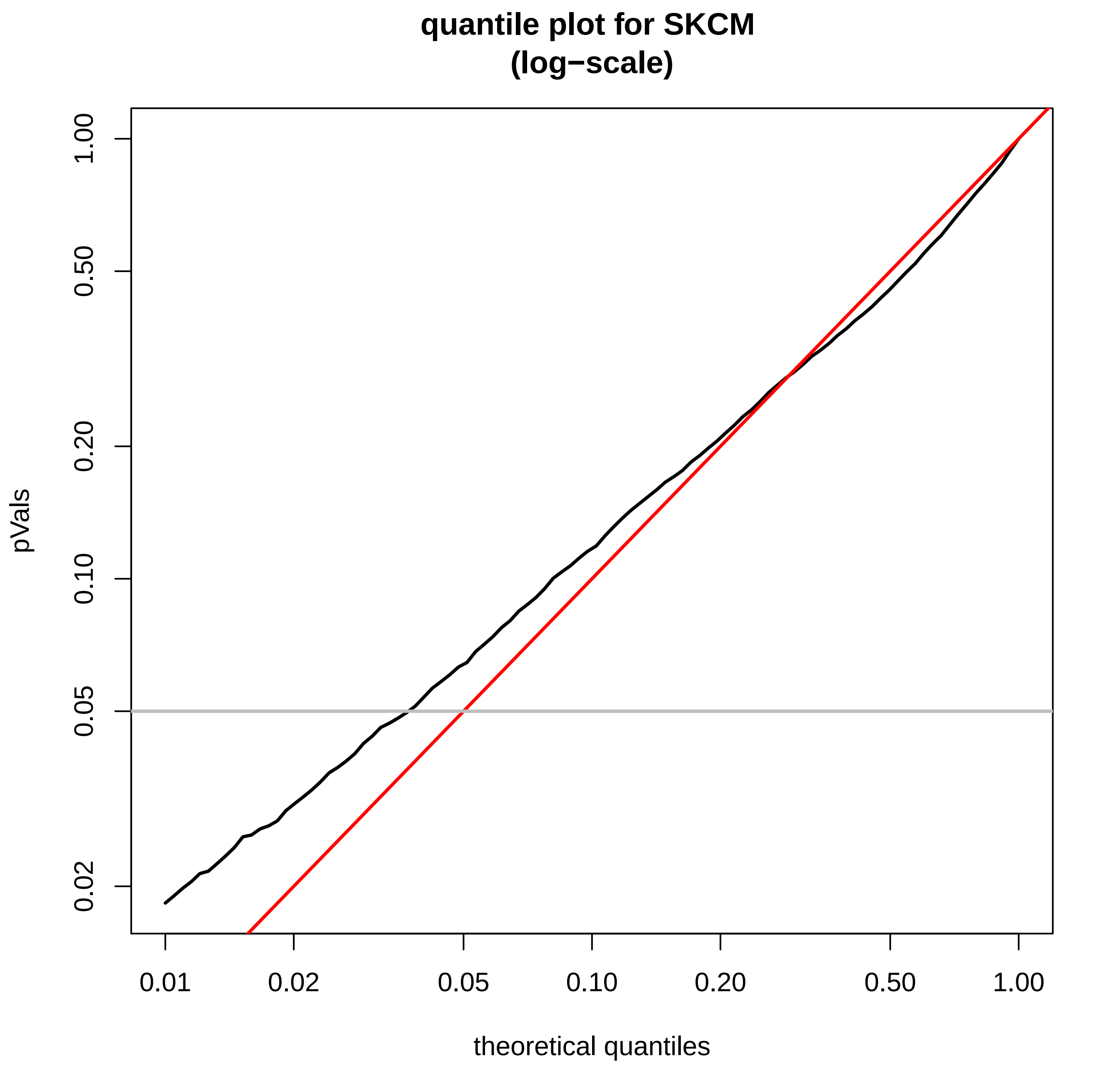
quantile plot for SKCM
(log−scale)
pVals
1.00
0.50
0.20
0.10
0.05
0.02
0.01
0.02
0.05
0.10
0.20
0.50
1.00
theoretical quantiles

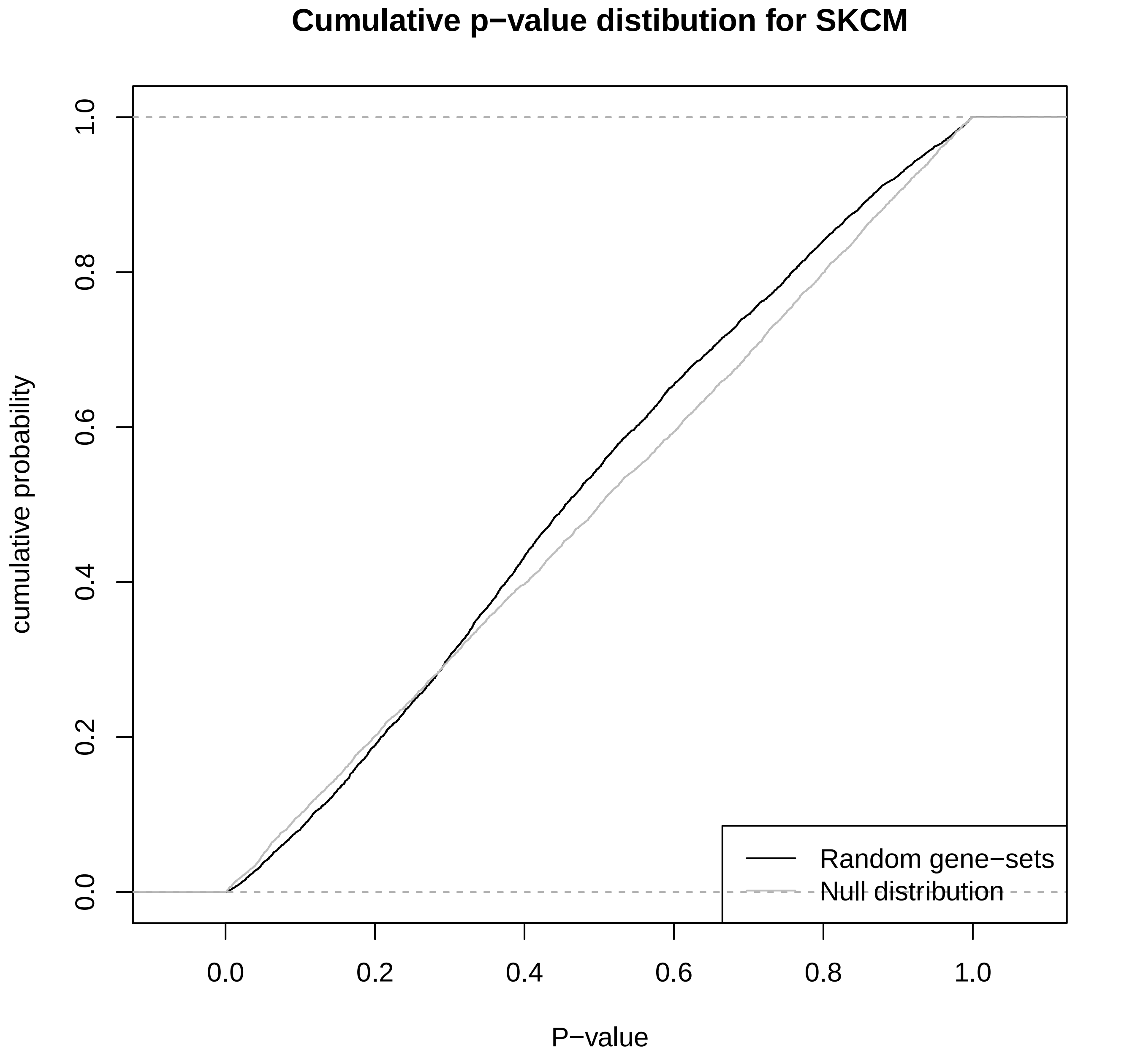

Cumulative p−value distibution for SKCM
cumulative probability
0.0
0.2
0.4
0.6
0.8
1.0
P−value
Random gene−sets
Null distribution

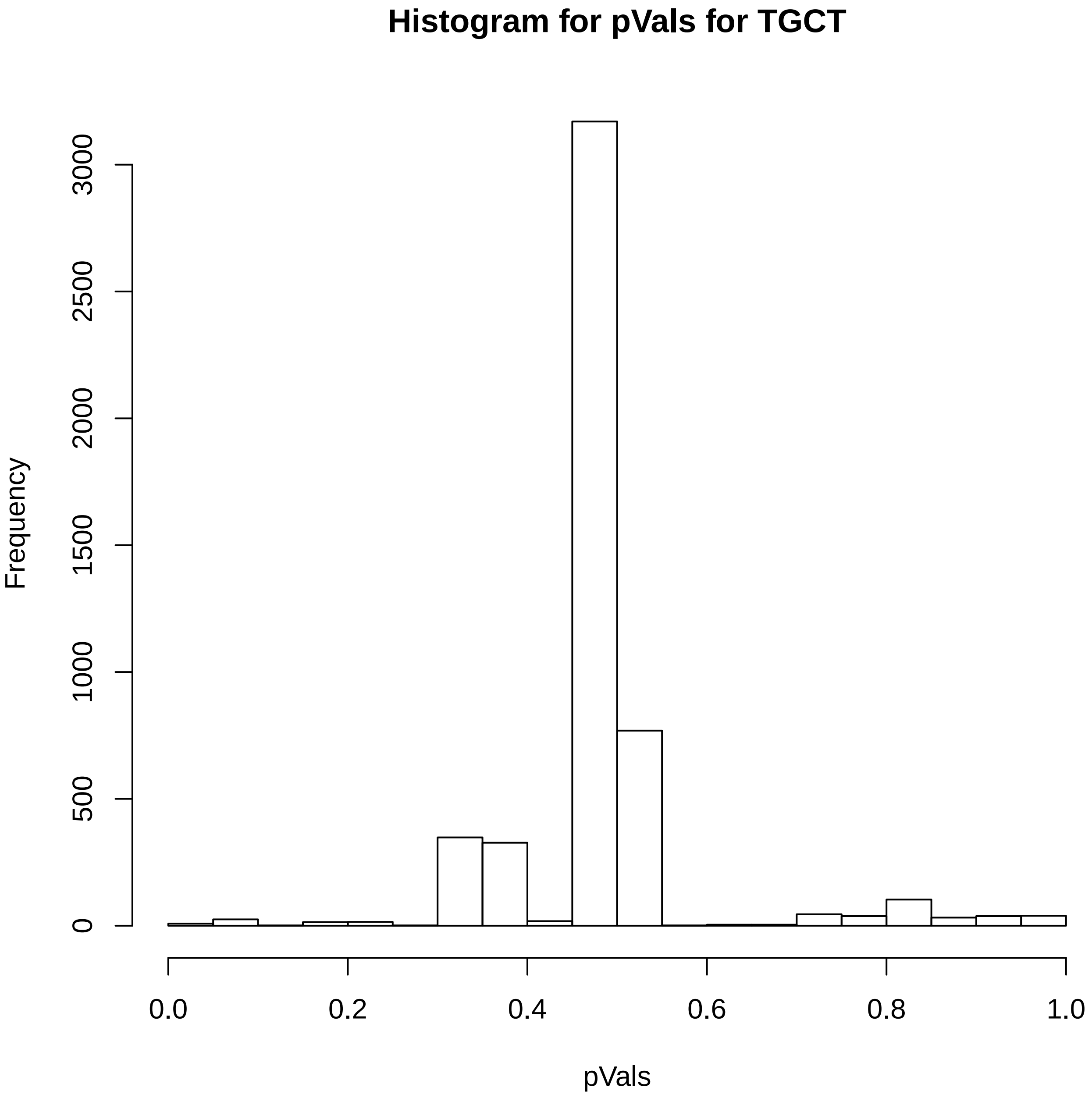
Histogram for pVals for TGCT
Frequency
0
500
1000
1500
2000
2500
3000
0.0
0.2
0.4
0.6
0.8
1.0
pVals

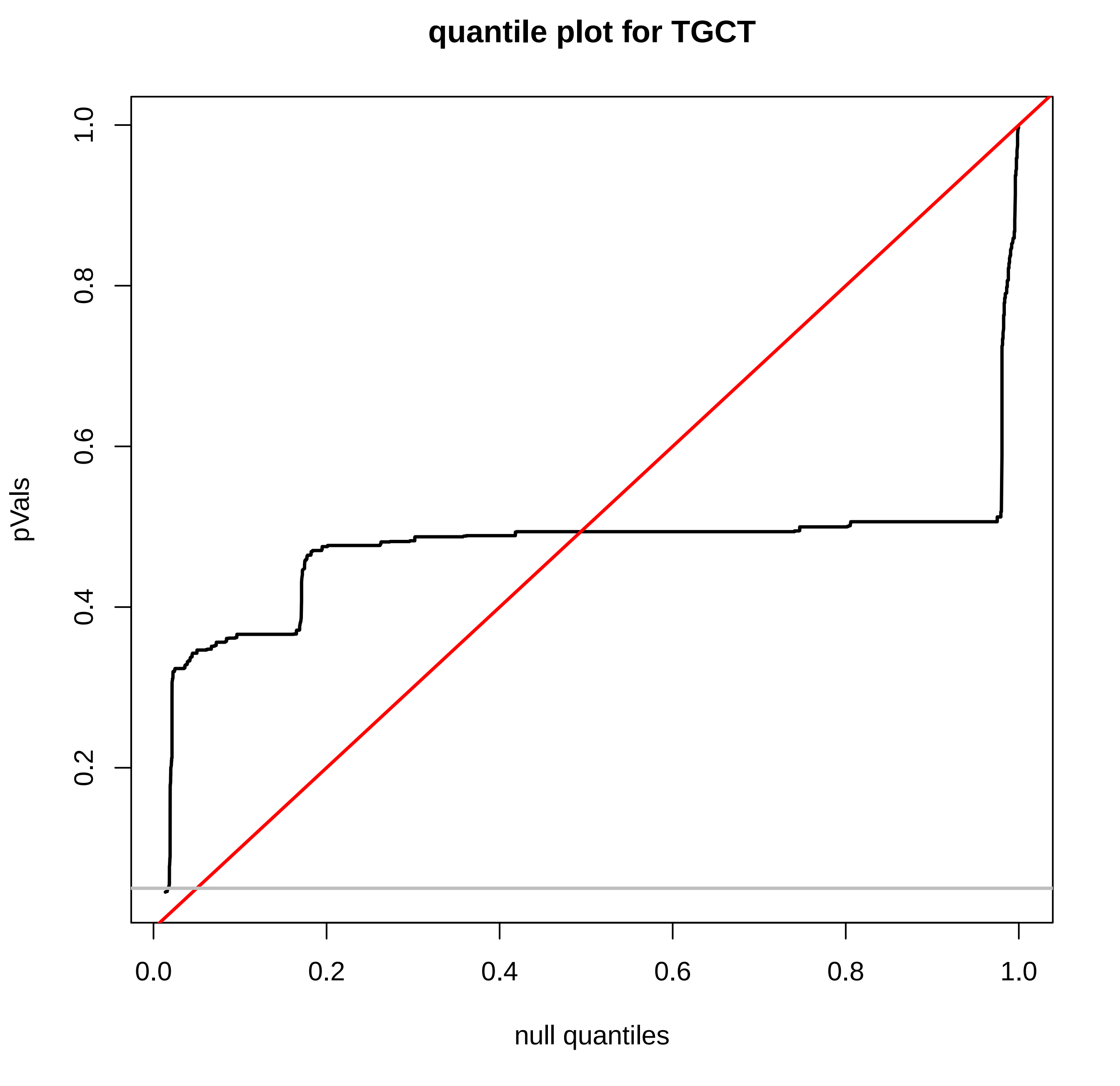
quantile plot for TGCT
pVals
null quantiles
0.0
0.2
0.4
0.6
0.8
1.0
0.2
0.4
0.6
0.8
1.0

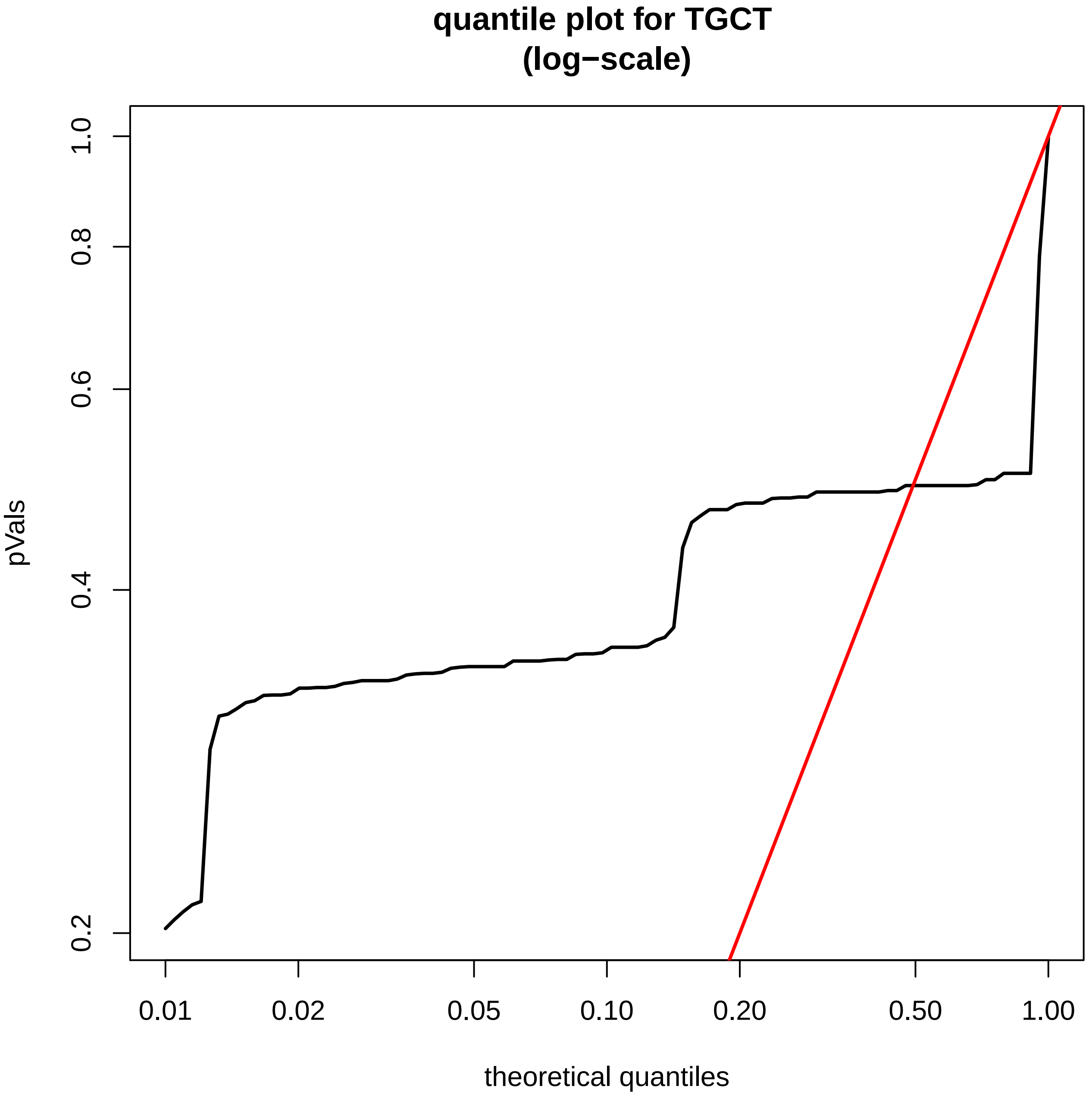
quantile plot for TGCT
(log−scale)
pVals
1.0
0.8
0.6
0.4
0.2
0.01
0.02
0.05
0.10
0.20
0.50
1.00
theoretical quantiles

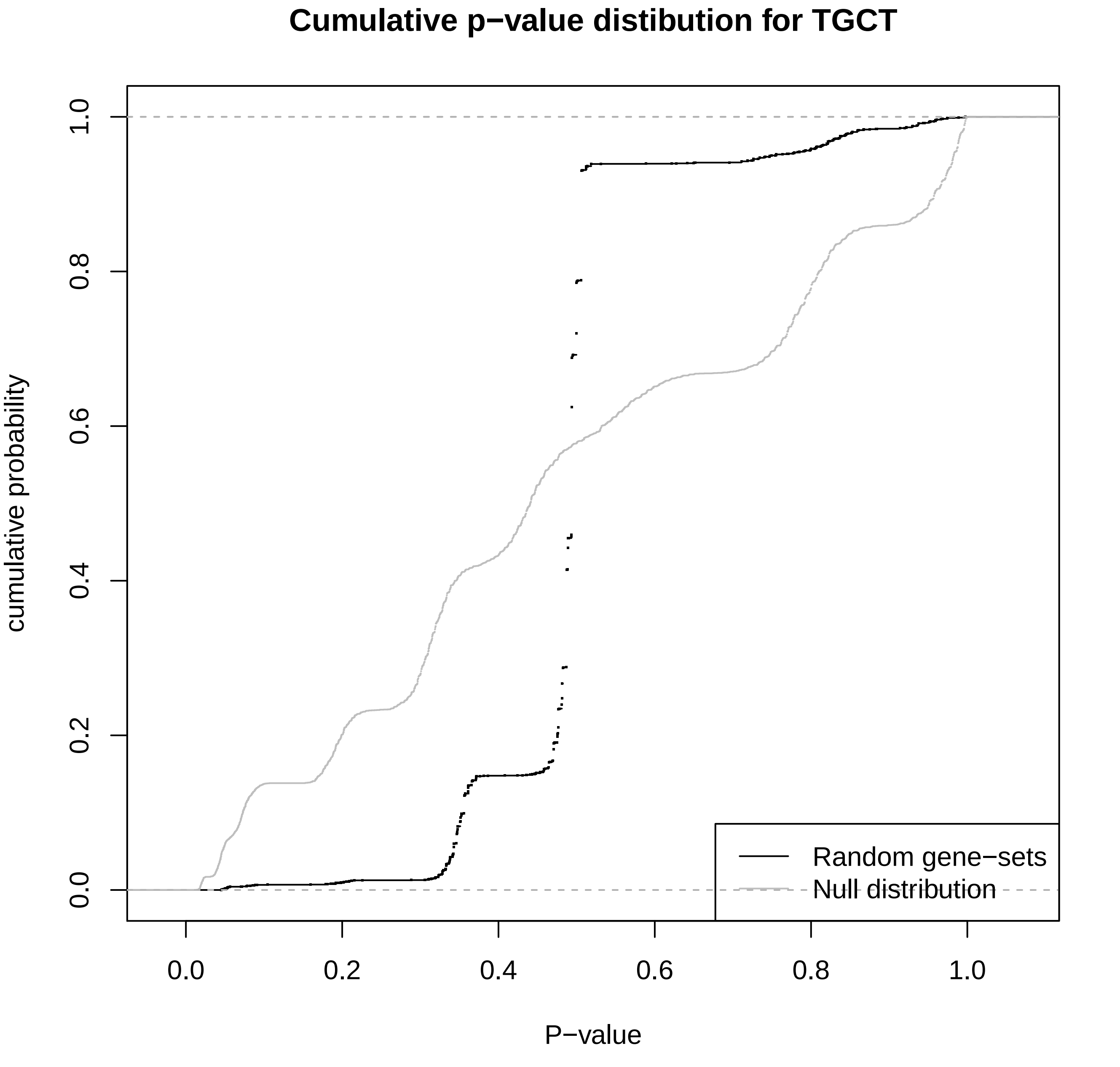

Cumulative p−value distibution for TGCT
cumulative probability
P−value
0.0
0.2
0.4
0.6
0.8
1.0
0.0
0.2
0.4
0.6
0.8
1.0
Random gene−sets
Null distribution

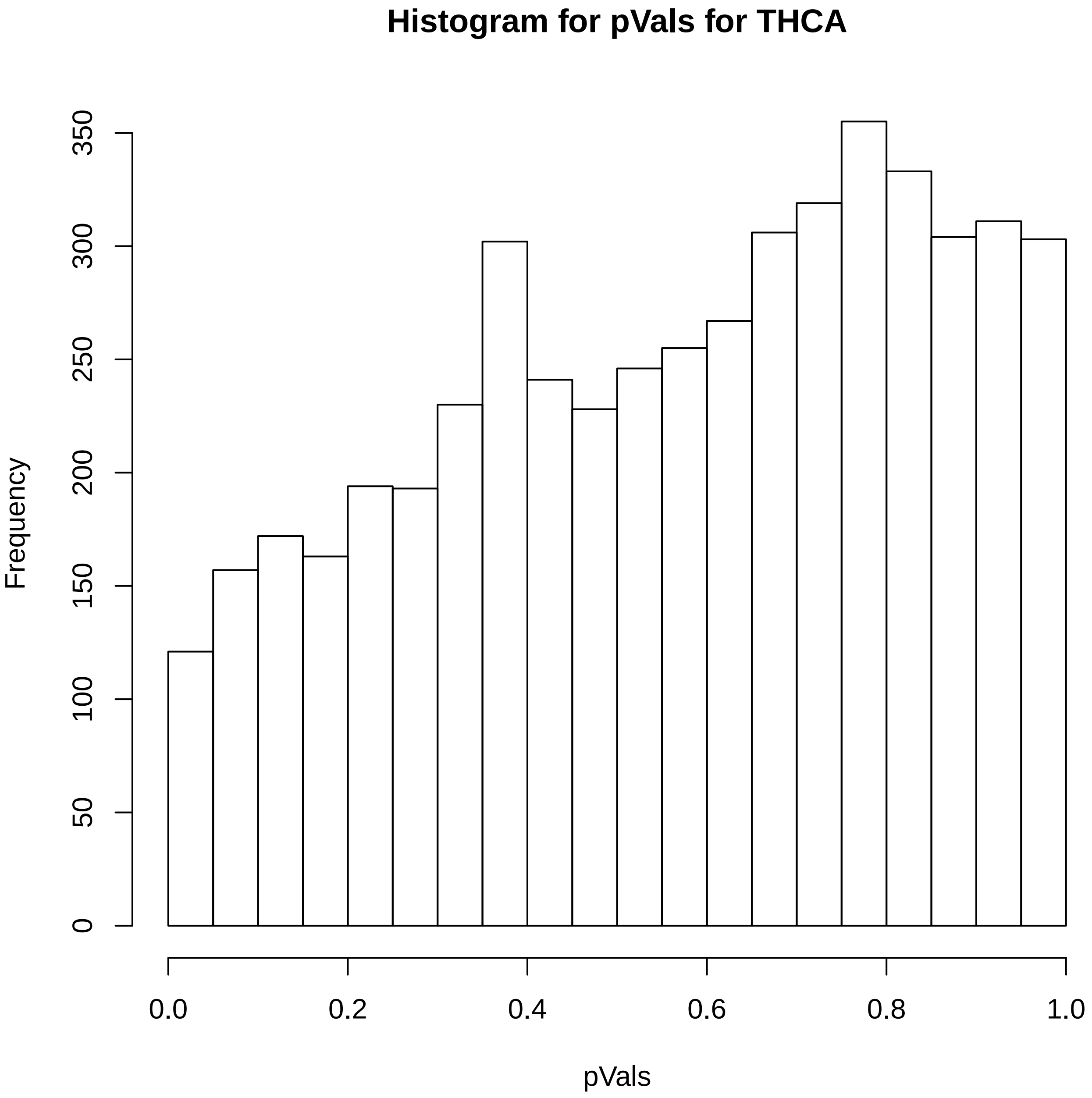
Histogram for pVals for THCA
Frequency
0
50
100
150
200
250
300
350
0.0
0.2
0.4
0.6
0.8
1.0
pVals

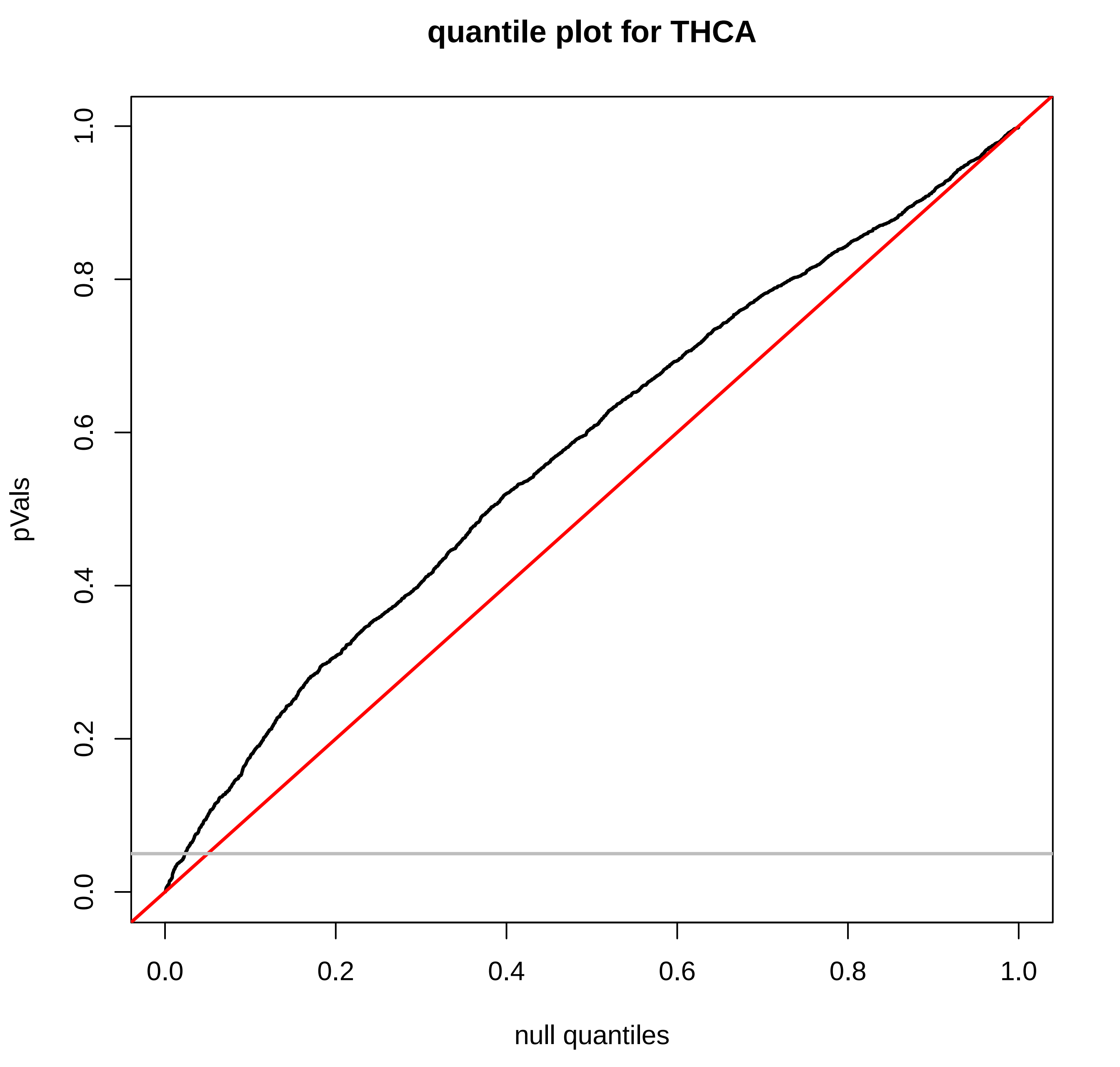
quantile plot for THCA
pVals
0.0
0.2
0.4
0.6
0.8
1.0
null quantiles
0.0
0.2
0.4
0.6
0.8
1.0

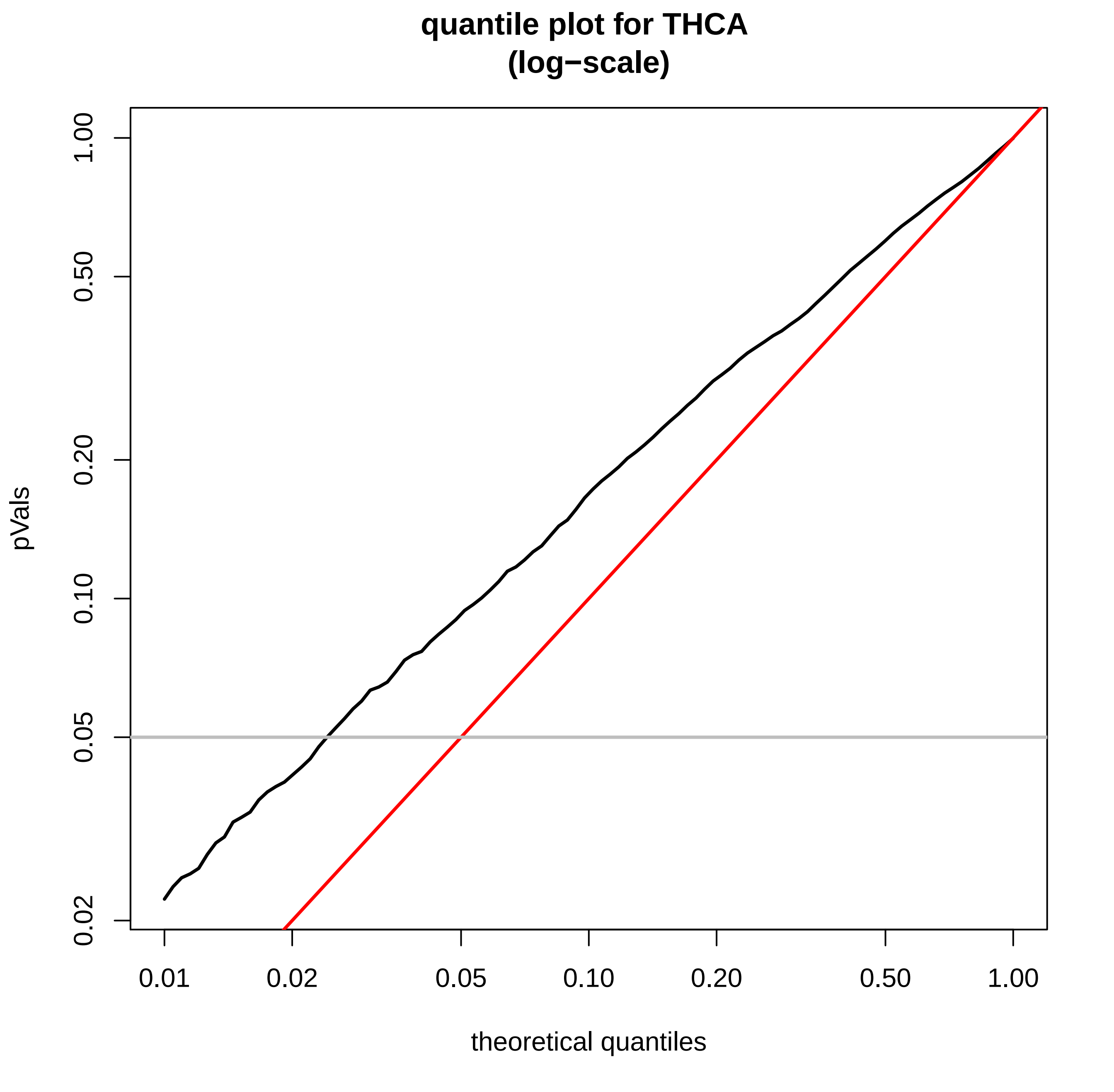
quantile plot for THCA
(log−scale)
pVals
1.00
0.50
0.20
0.10
0.05
0.02
0.01
0.02
0.05
0.10
0.20
0.50
1.00
theoretical quantiles

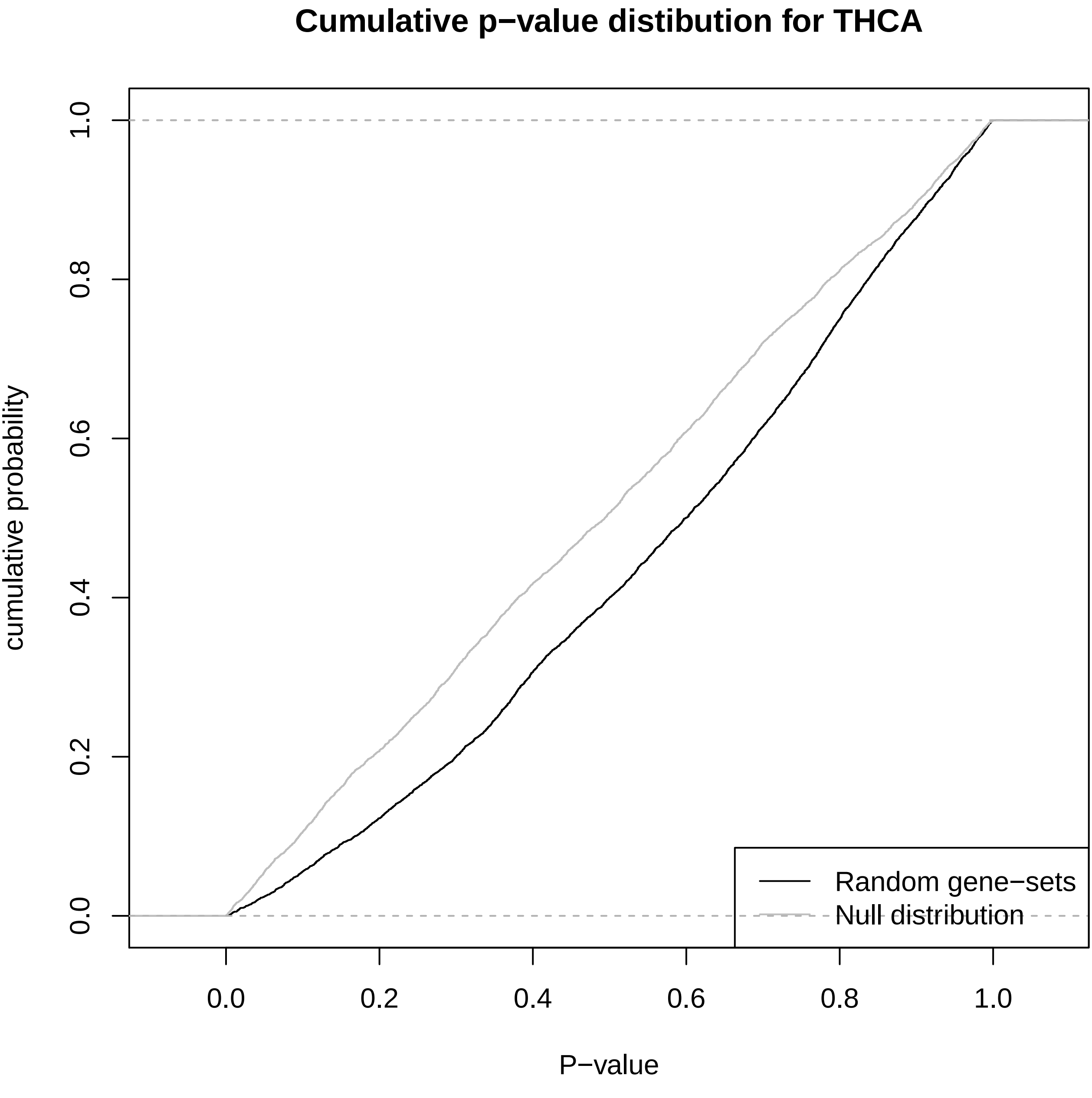
Cumulative p−value distibution for THCA
cumulative probability
P−value
0.0
0.2
0.4
0.6
0.8
1.0
Random gene−sets
Null distribution

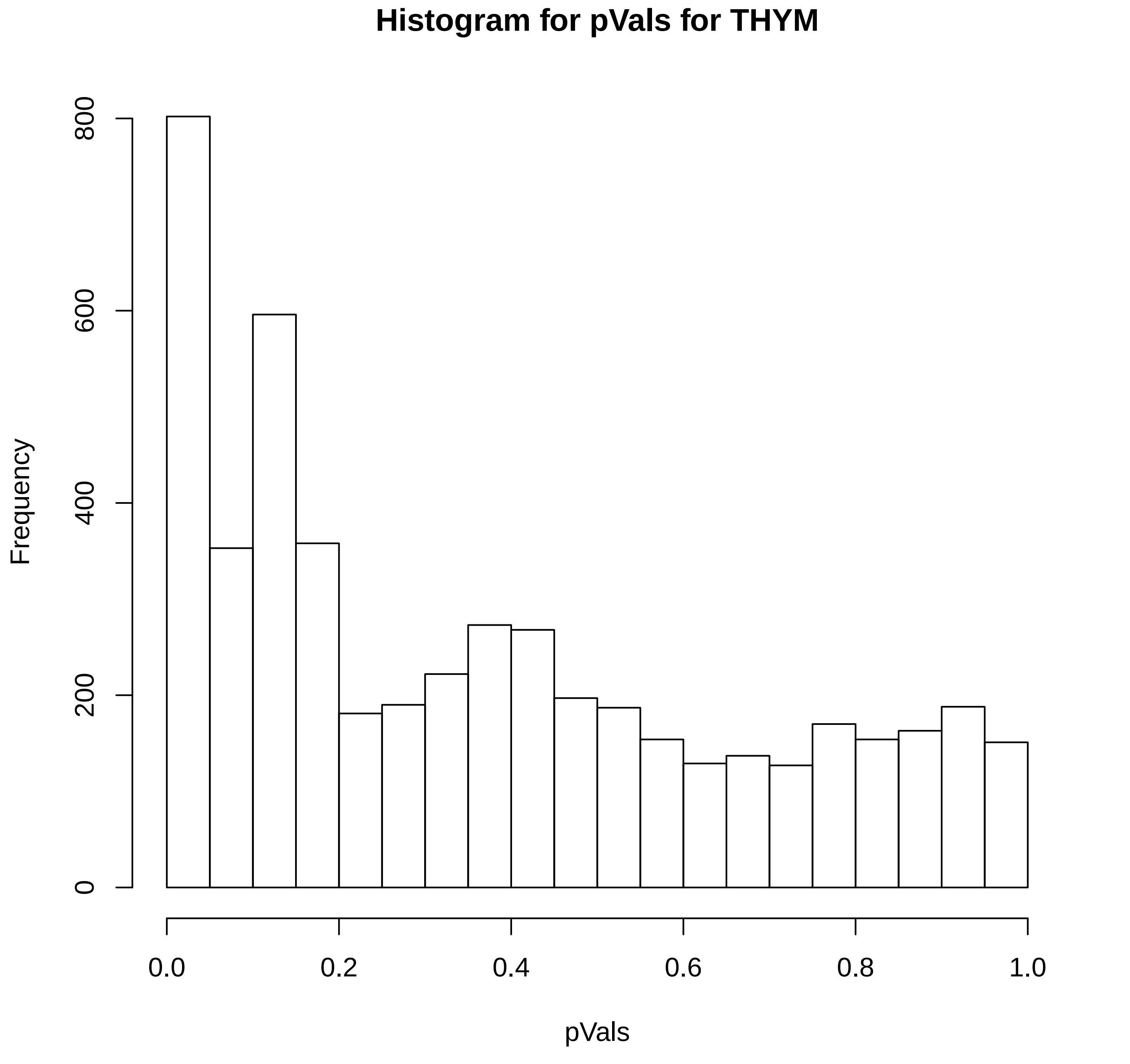

Histogram for pVals for THYM
Frequency
0
200
400
600
800
0.0
0.2
0.4
0.6
0.8
1.0
pVals

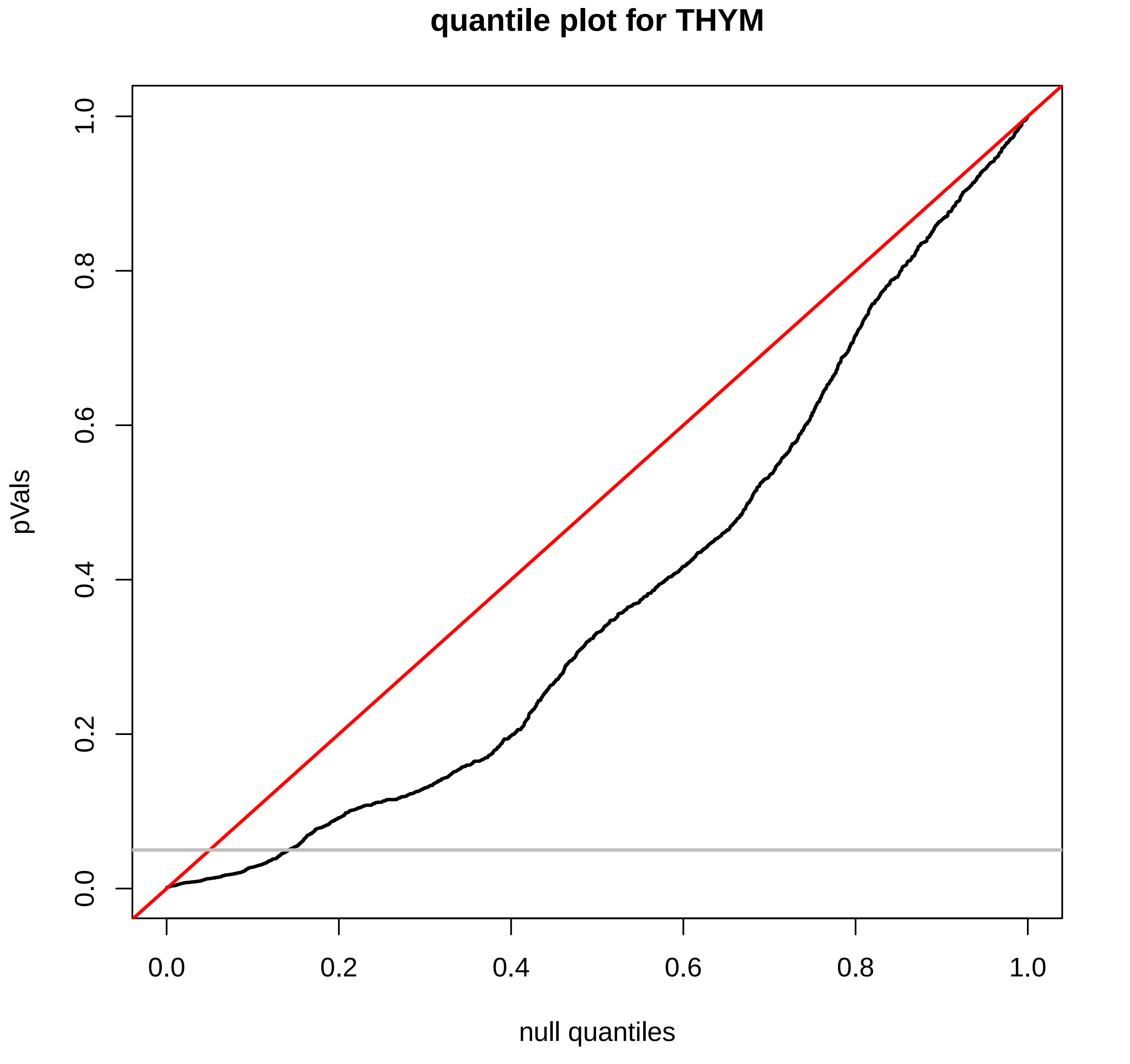
quantile plot for THYM
pVals
null quantiles
0.0
0.2
0.4
0.6
0.8
1.0
0.0
0.2
0.4
0.6
0.8
1.0

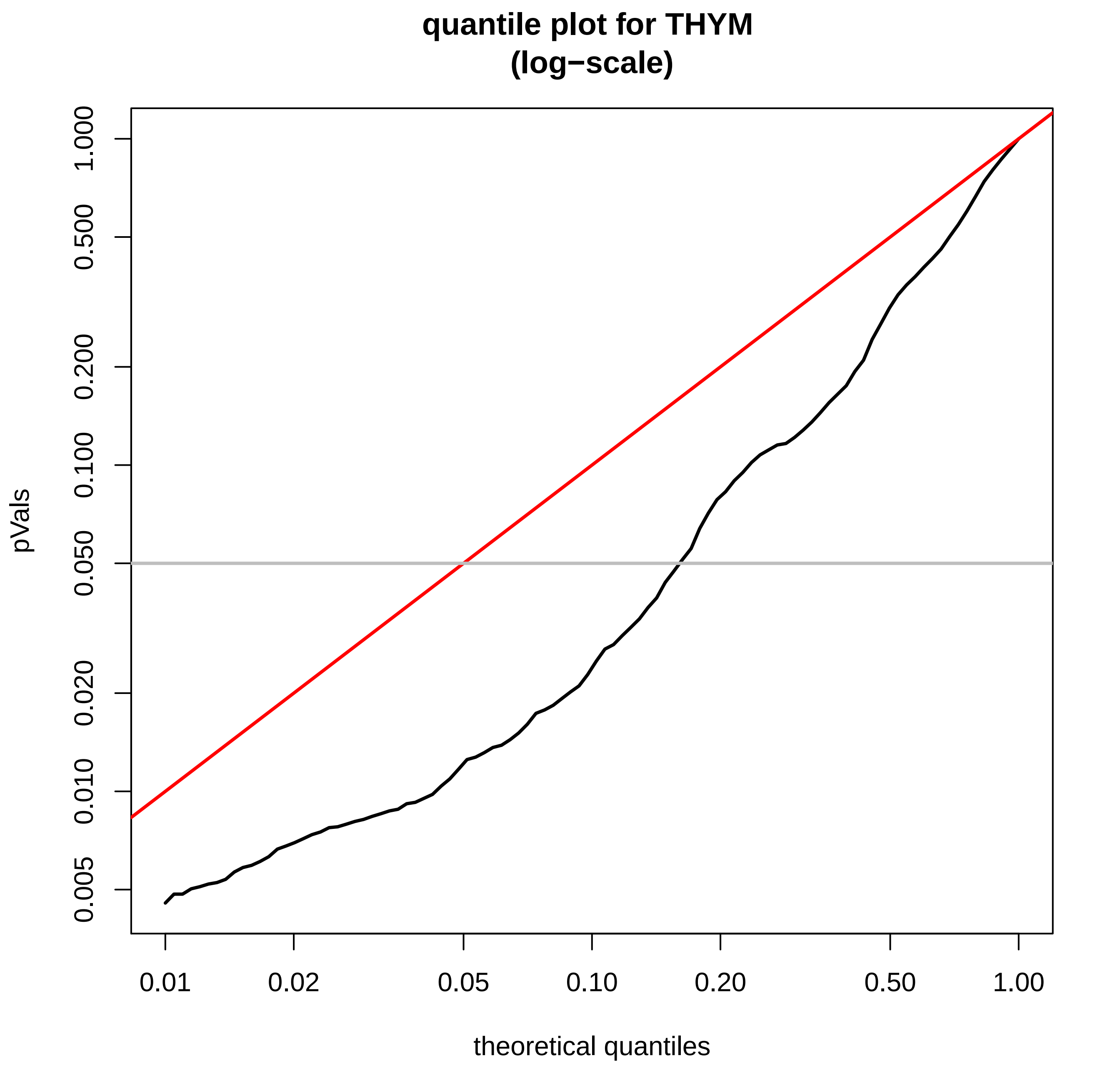
quantile plot for THYM
(log−scale)
pVals
1.000
0.500
0.200
0.100
0.050
0.020
0.010
0.005
0.01
0.02
0.05
0.10
0.20
0.50
1.00
theoretical quantiles

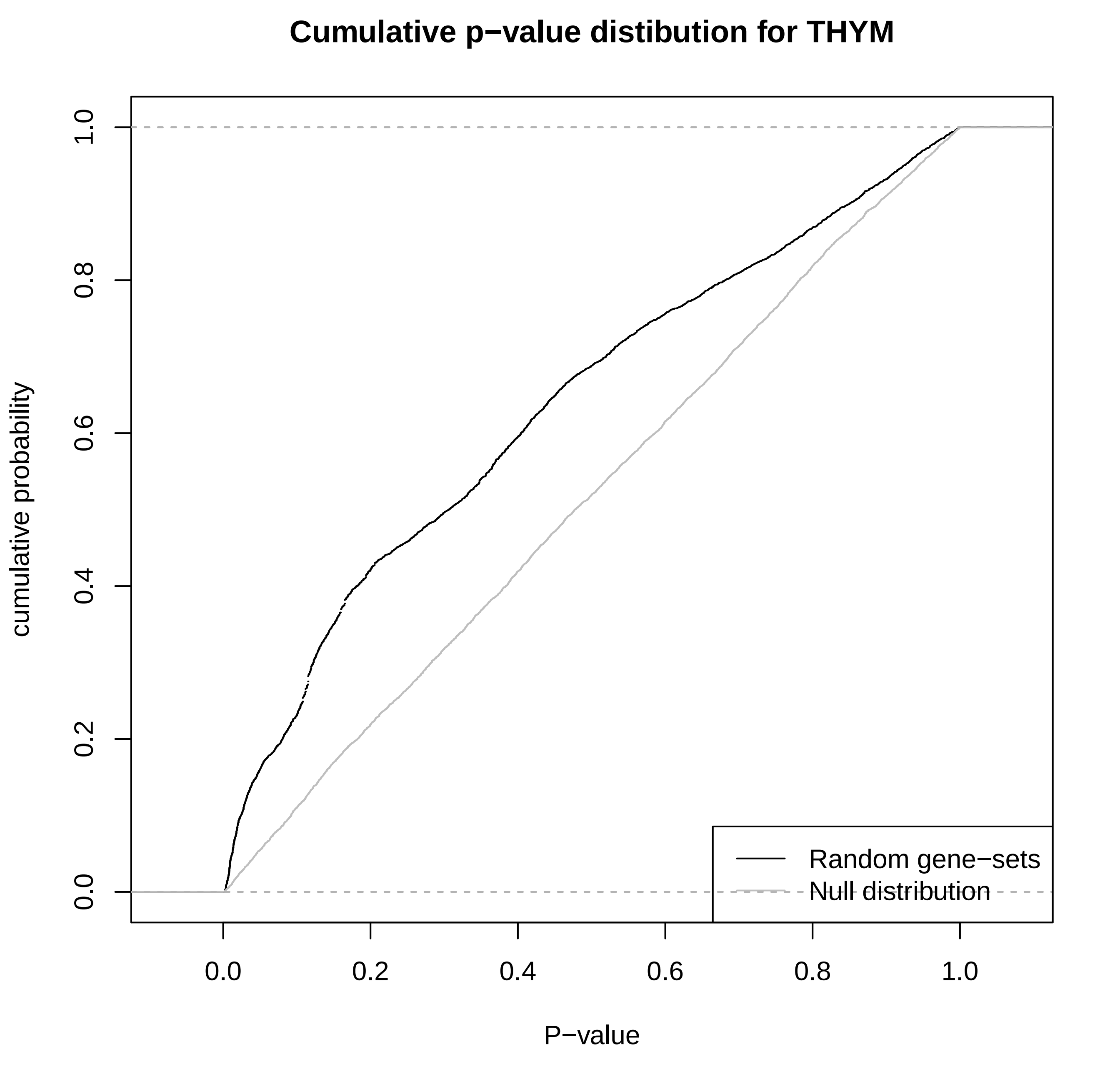

Cumulative p−value distibution for THYM
cumulative probability
0.0
0.2
0.4
0.6
0.8
1.0
P−value
0.0
0.2
0.4
0.6
0.8
1.0
Random gene−sets
Null distribution

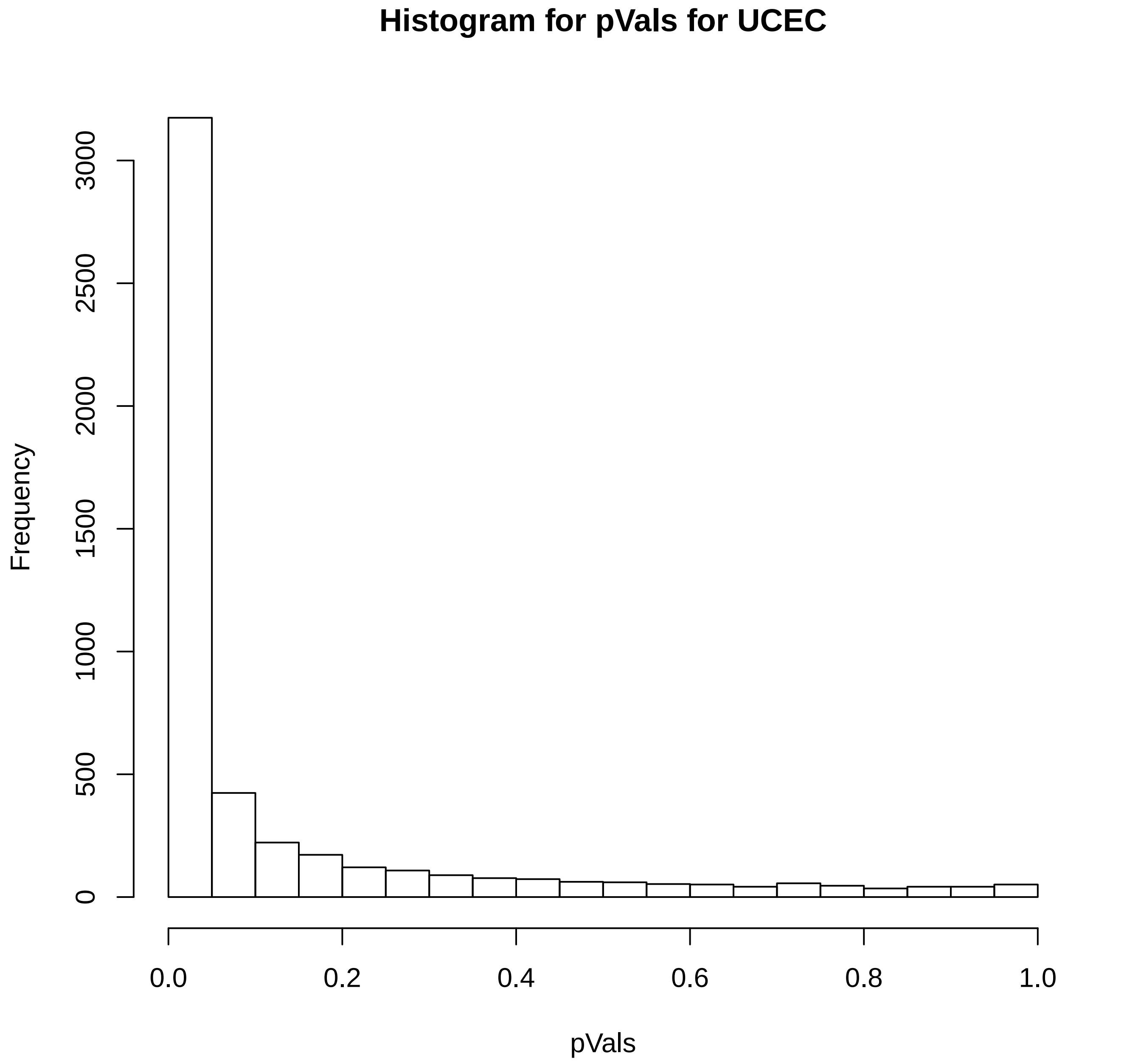

Histogram for pVals for UCEC
Frequency
0
500
1000
1500
2000
2500
3000
0.0
0.2
0.4
0.6
0.8
1.0
pVals

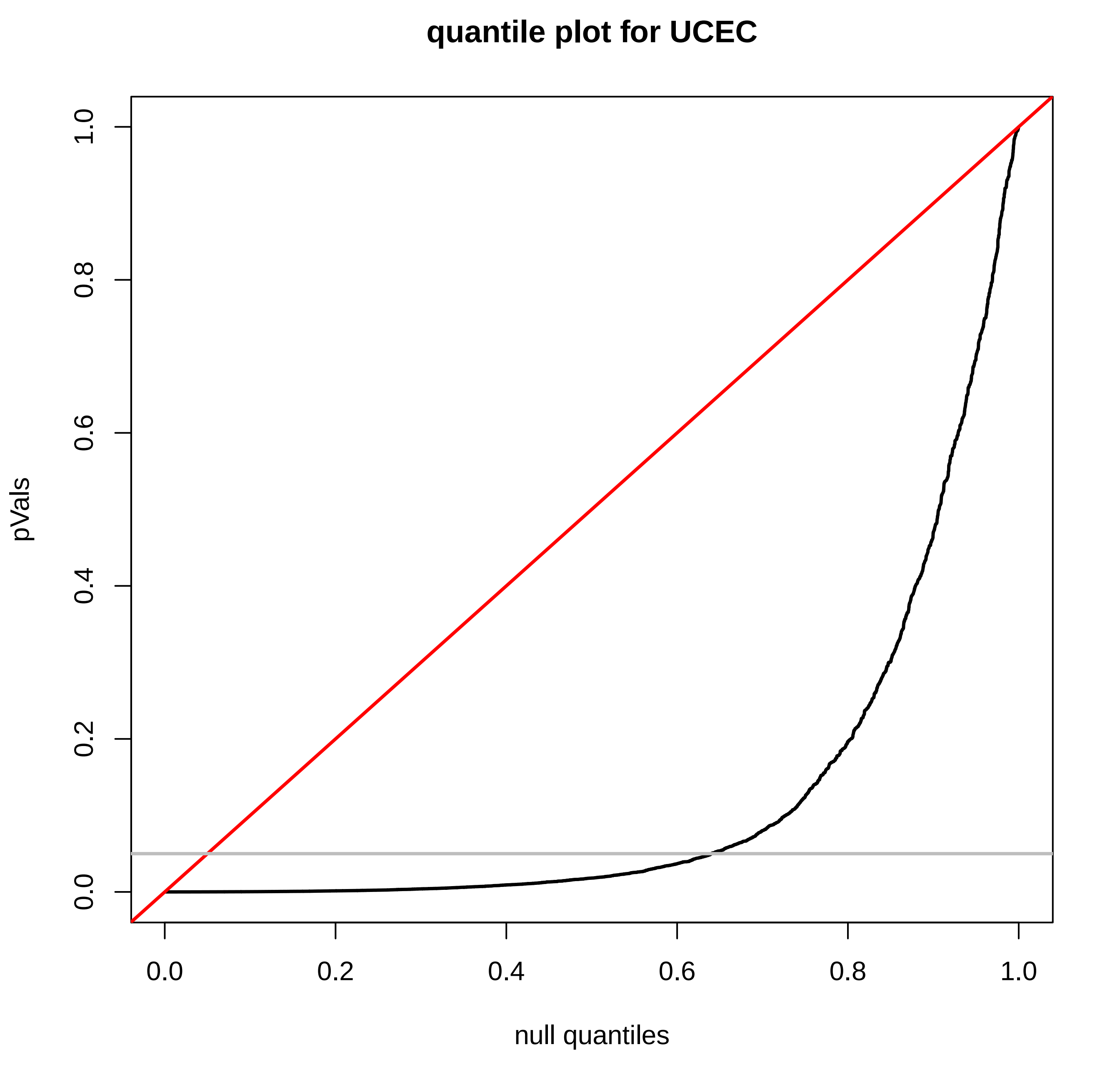

quantile plot for UCEC
pVals
null quantiles
0.0
0.2
0.4
0.6
0.8
1.0

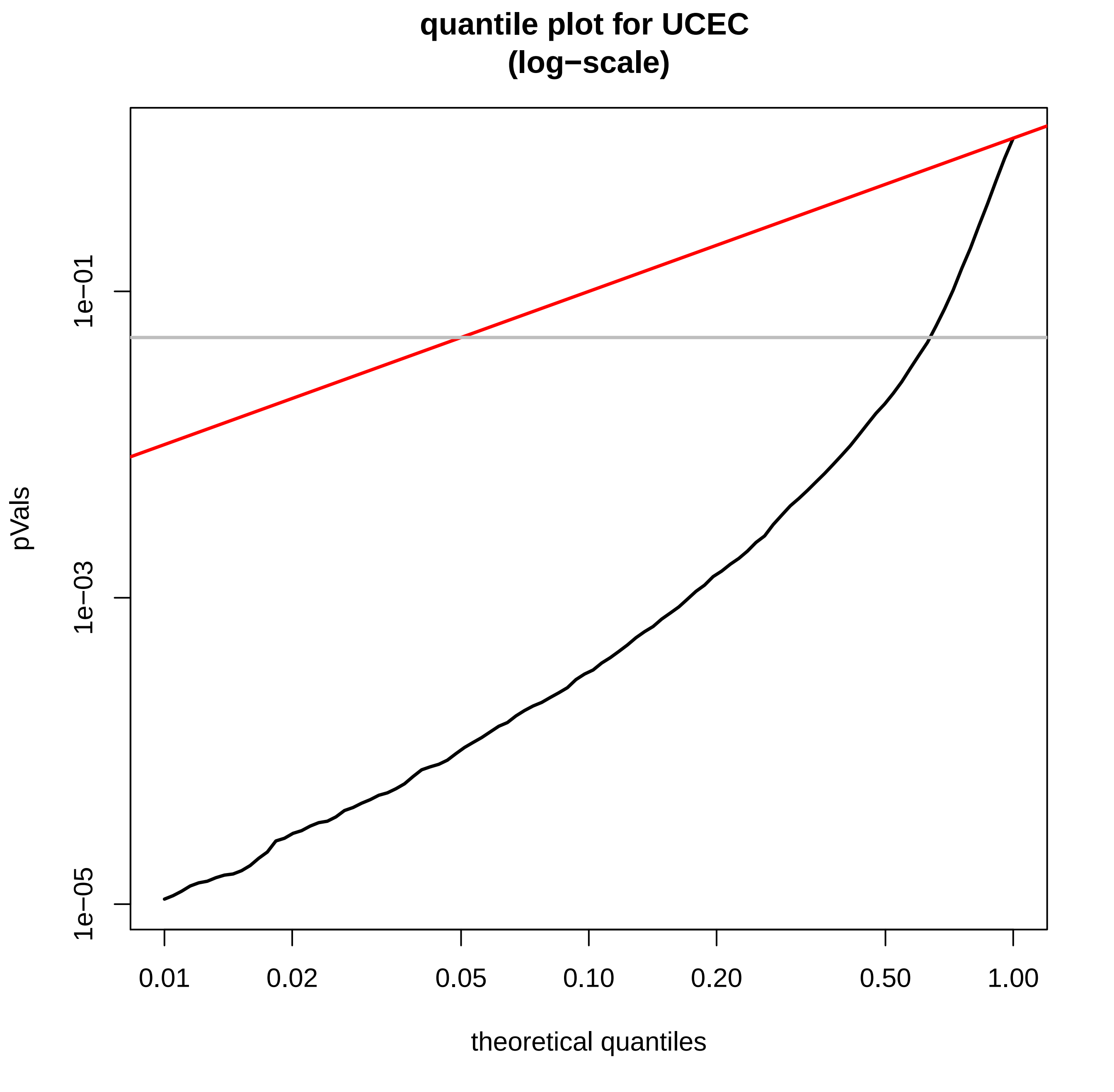
quantile plot for UCEC
(log−scale)
pVals
1e−01
1e−03
1e−05
0.01
0.02
0.05
0.10
0.20
0.50
1.00
theoretical quantiles

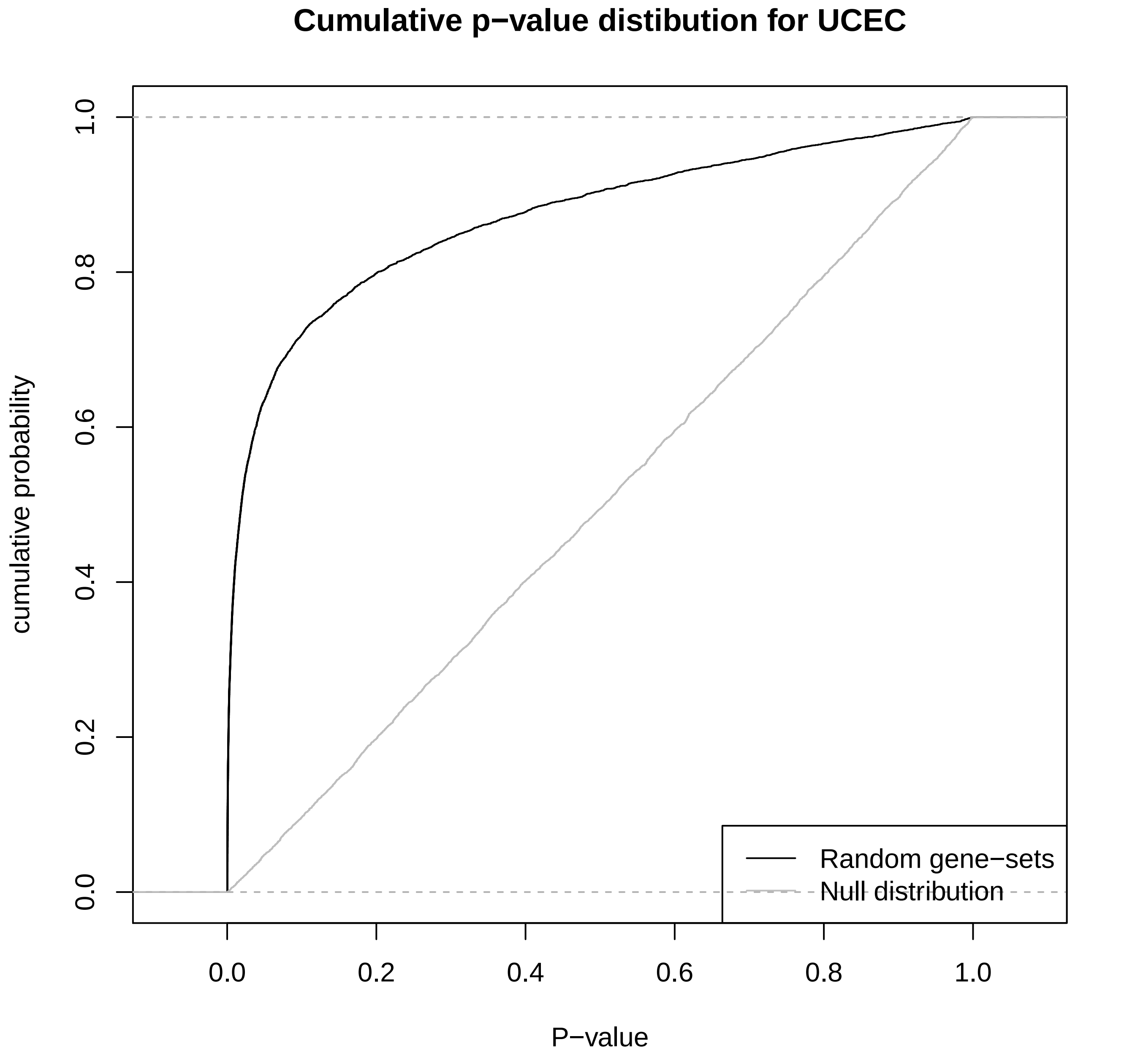
Cumulative p−value distibution for UCEC
cumulative probability
0.0
0.2
0.4
0.6
0.8
1.0
P−value
0.0
0.2
0.4
0.6
0.8
1.0
Random gene−sets
Null distribution

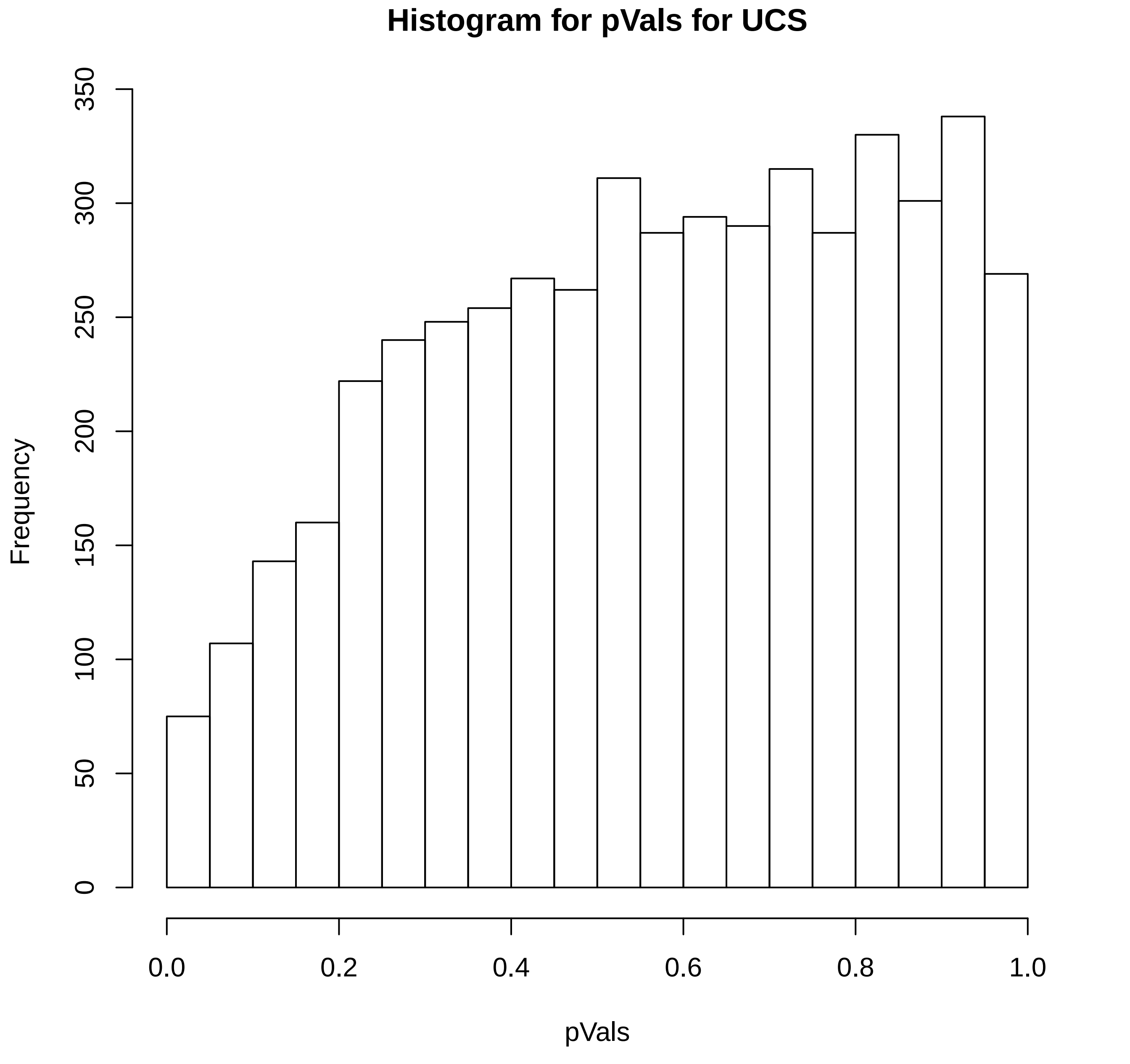
Histogram for pVals for UCS
Frequency
0
50
100
150
200
250
300
350
0.0
0.2
0.4
0.6
0.8
1.0
pVals

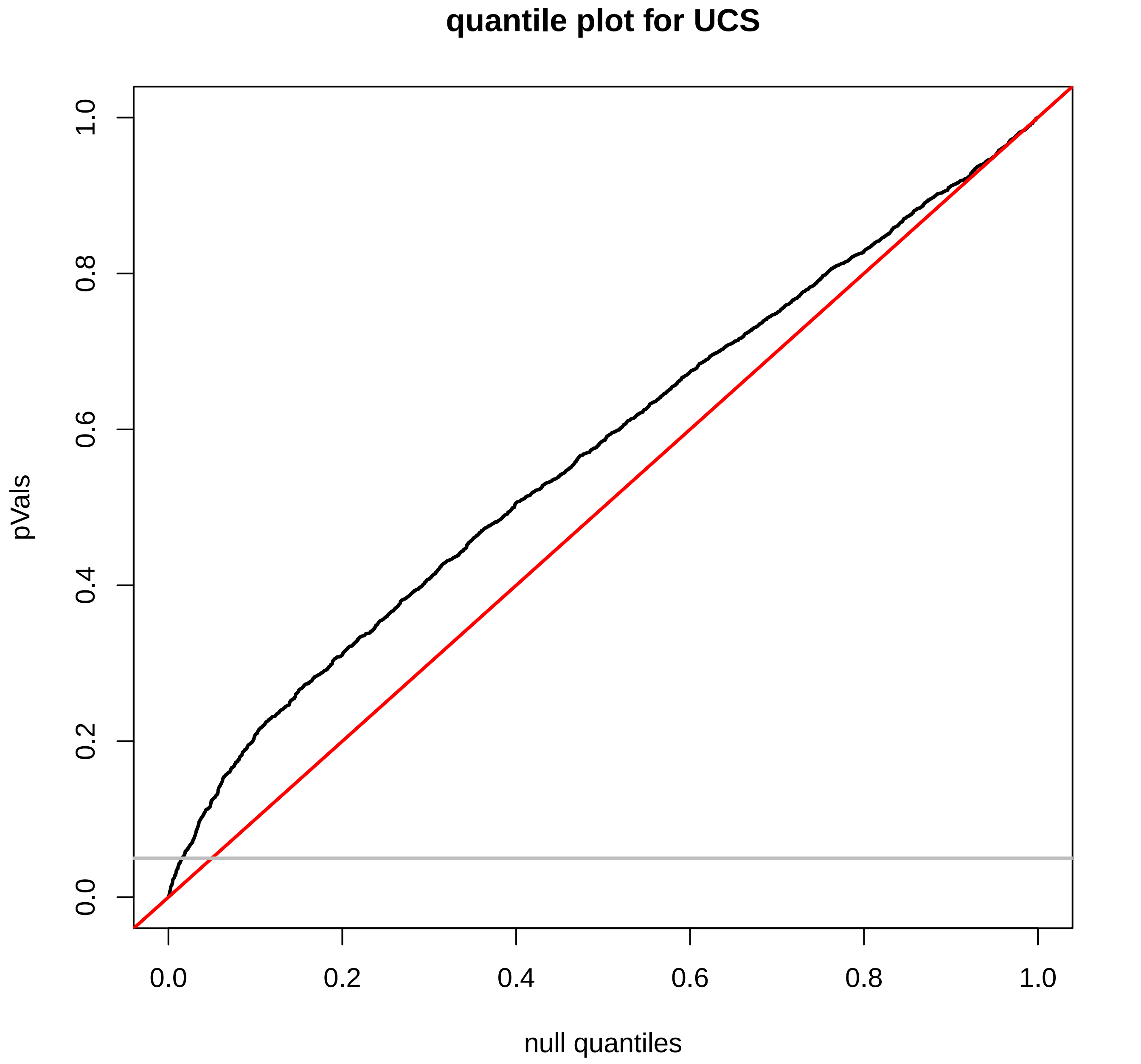
quantile plot for UCS
pVals
null quantiles
0.0
0.2
0.4
0.6
0.8
1.0
0.0
0.2
0.4
0.6
0.8
1.0

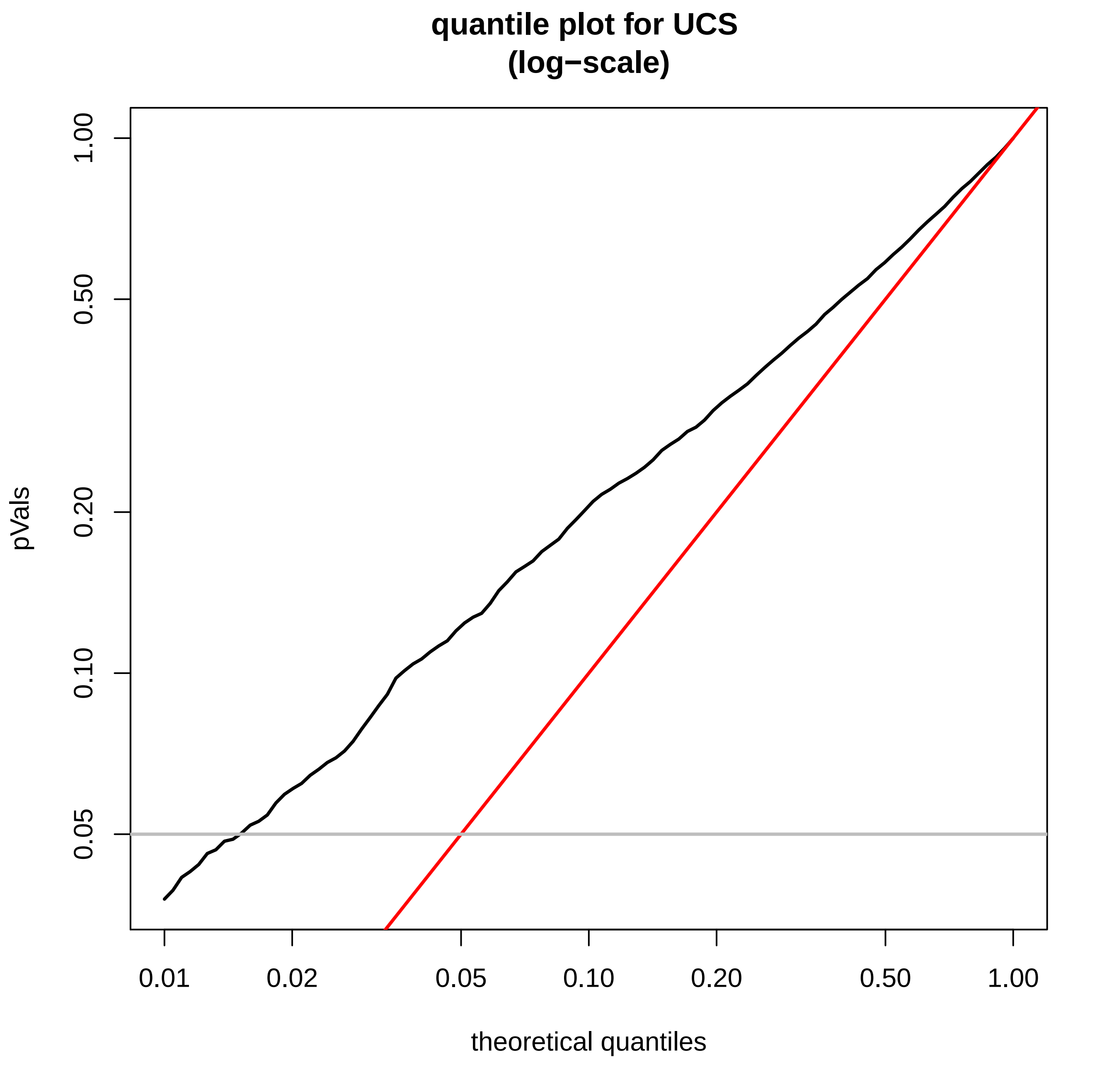
quantile plot for UCS
(log−scale)
pVals
1.00
0.50
0.20
0.10
0.05
0.01
0.02
0.05
0.10
0.20
0.50
1.00
theoretical quantiles

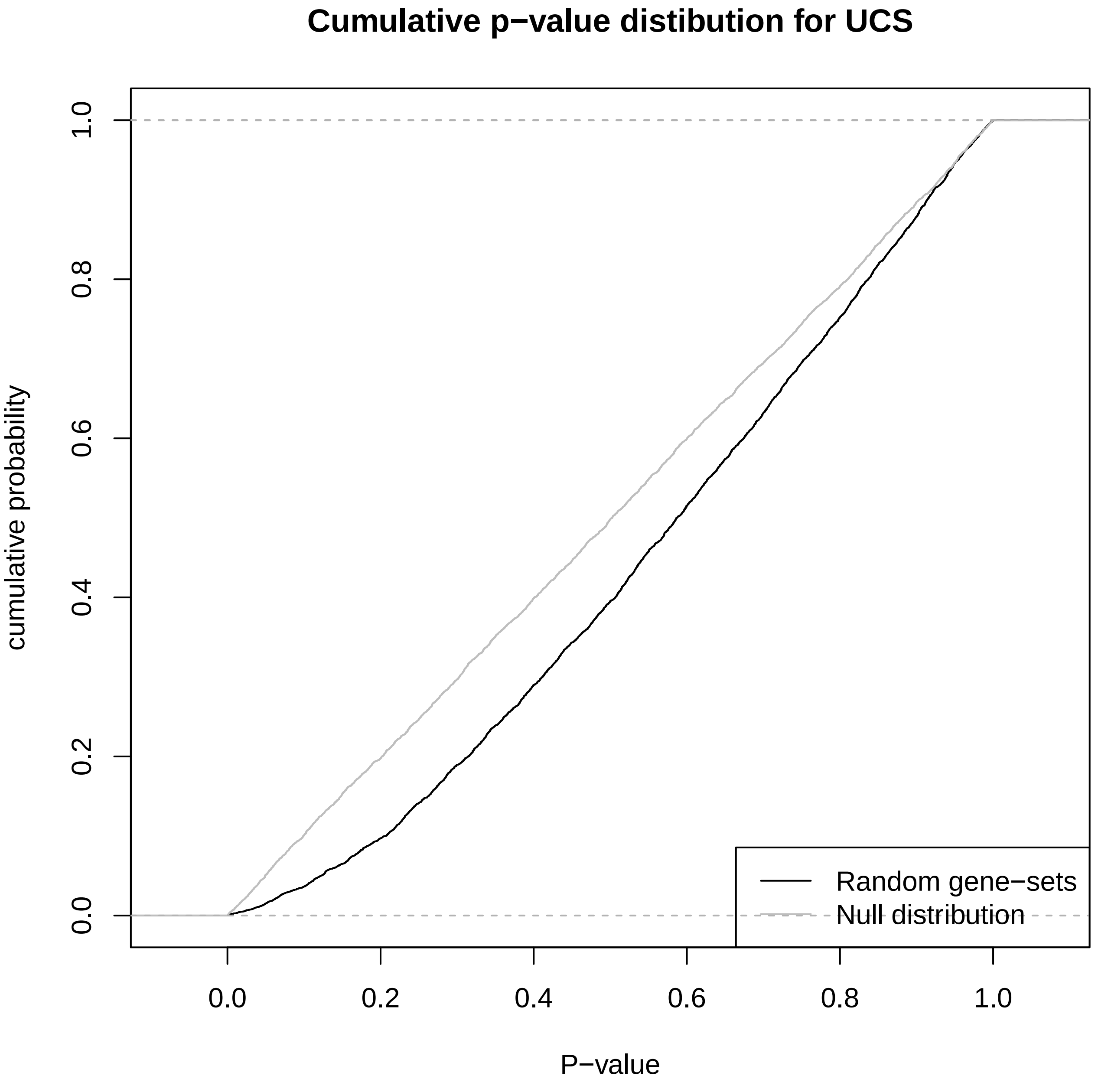

Cumulative p−value distibution for UCS
cumulative probability
P−value
0.0
0.2
0.4
0.6
0.8
1.0
Random gene−sets
Null distribution

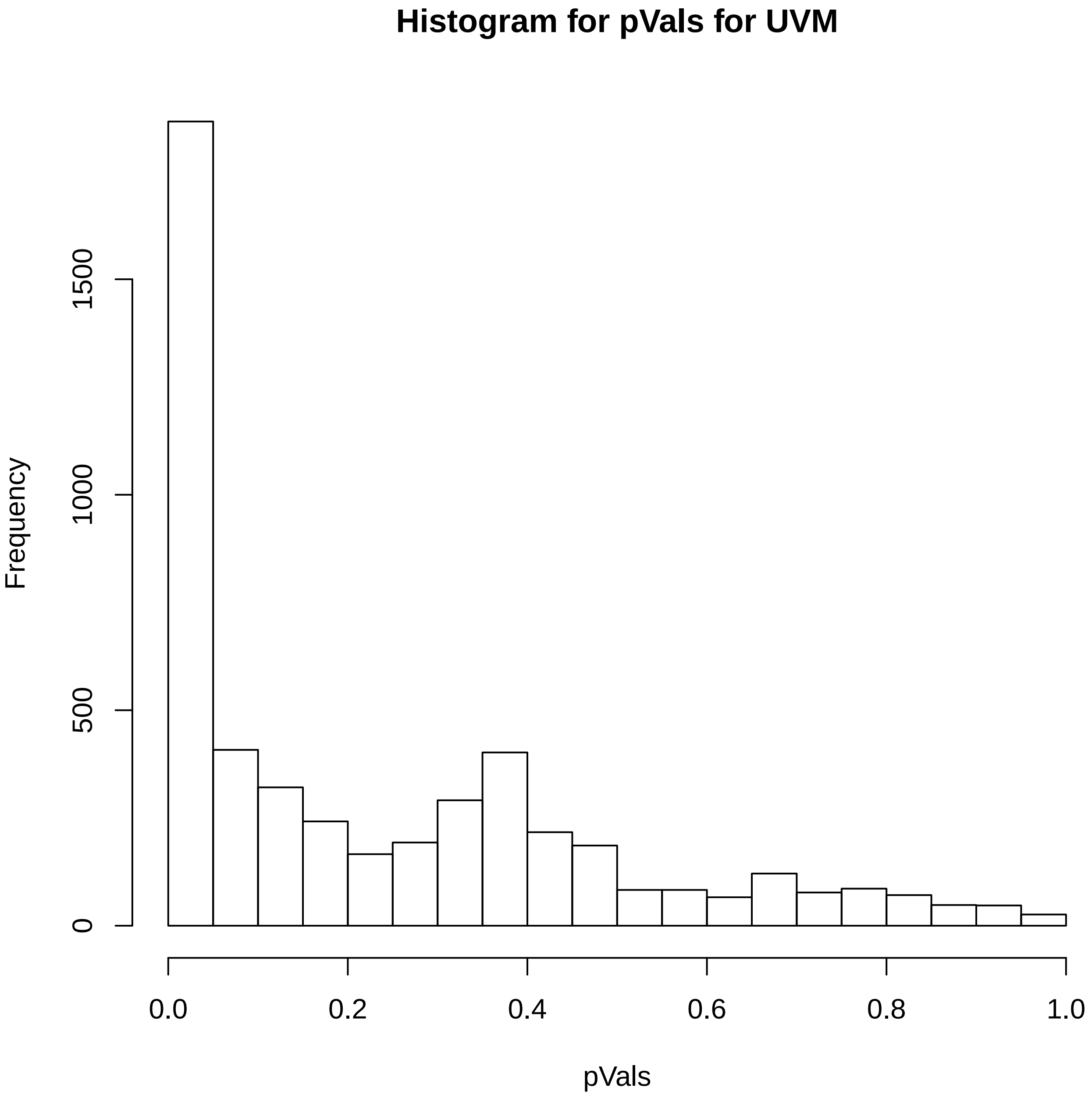
Histogram for pVals for UVM
Frequency
0
500
1000
1500
0.0
0.2
0.4
0.6
0.8
1.0
pVals

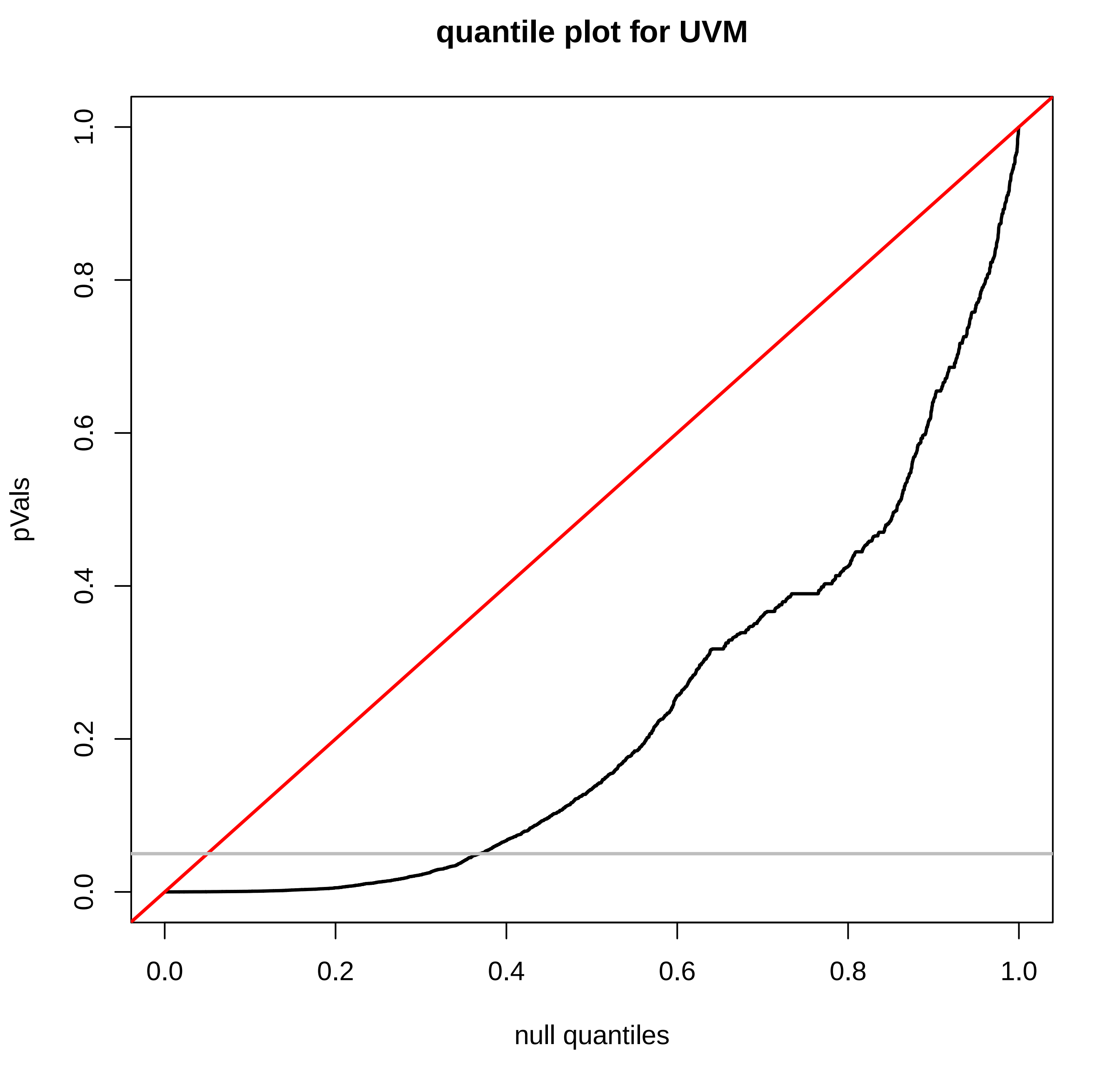
quantile plot for UVM
pVals
null quantiles
0.0
0.2
0.4
0.6
0.8
1.0

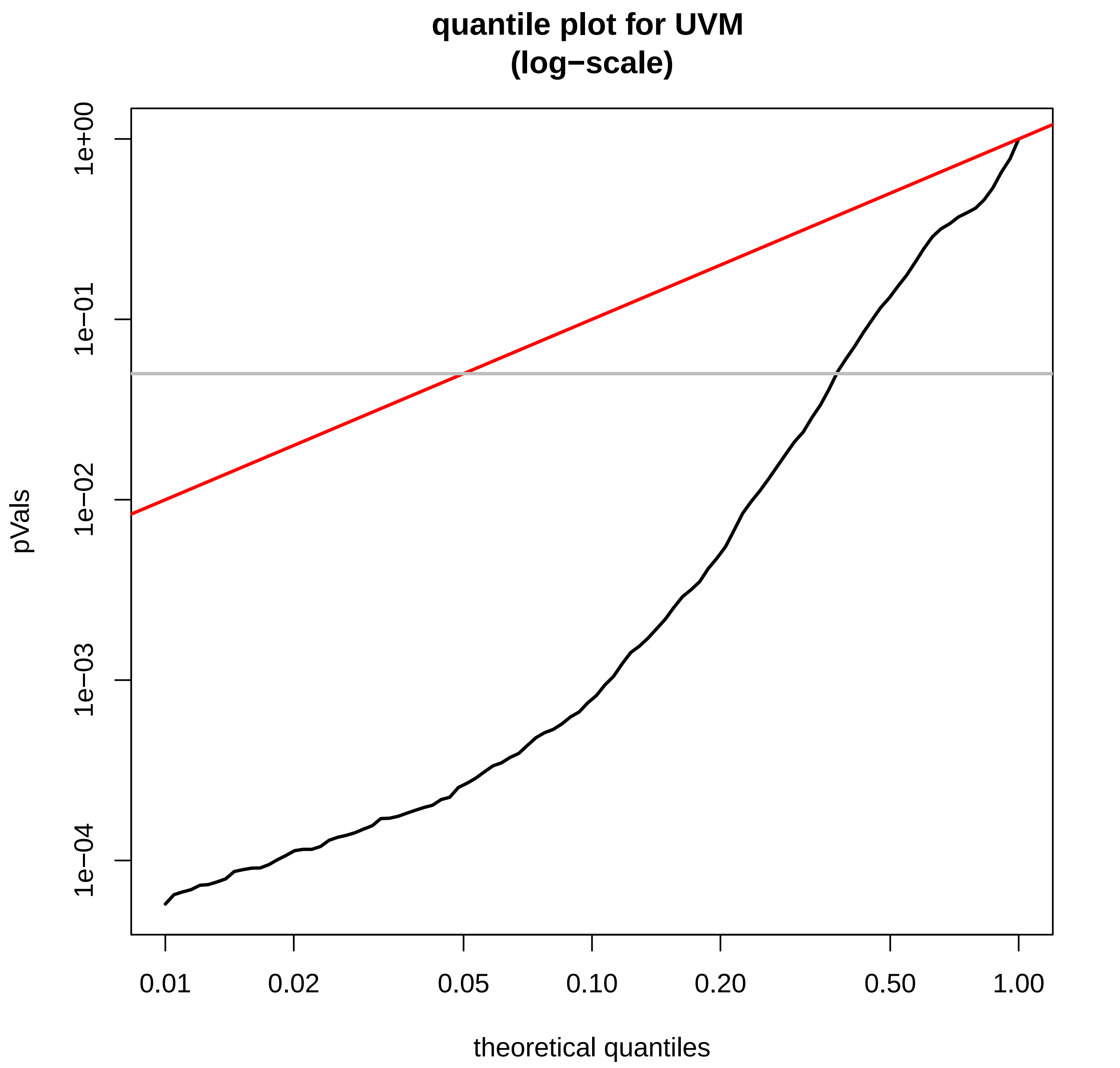
quantile plot for UVM
(log−scale)
1e+00
1e−01
1e−02
1e−03
1e−04
pVals
0.01
0.02
0.05
0.10
0.20
0.50
1.00
theoretical quantiles

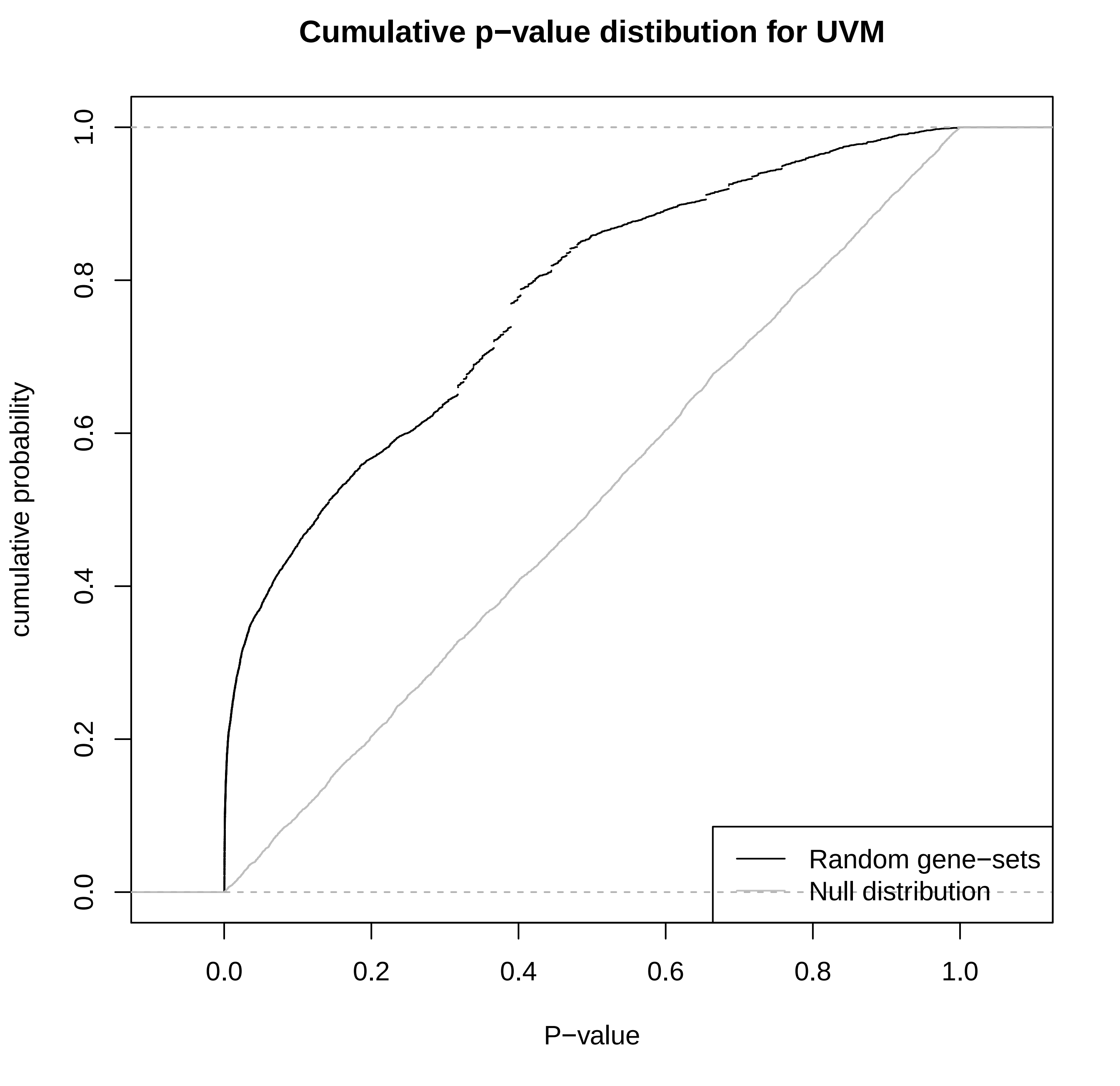
Cumulative p−value distibution for UVM
cumulative probability
0.0
0.2
0.4
0.6
0.8
1.0
P−value
0.0
0.2
0.4
0.6
0.8
1.0
Random gene−sets
Null distribution
