# Supplementary figures and images for: Association between expression of random gene sets and survival is evident in multiple cancer types and may be explained by sub-classification
Source: PLoS Comput Biol. 2018 Feb 22;14(2):e1006026. doi: 10.1371/journal.pcbi.1006026 (PMC5839591; doi:10.1371/journal.pcbi.1006026)

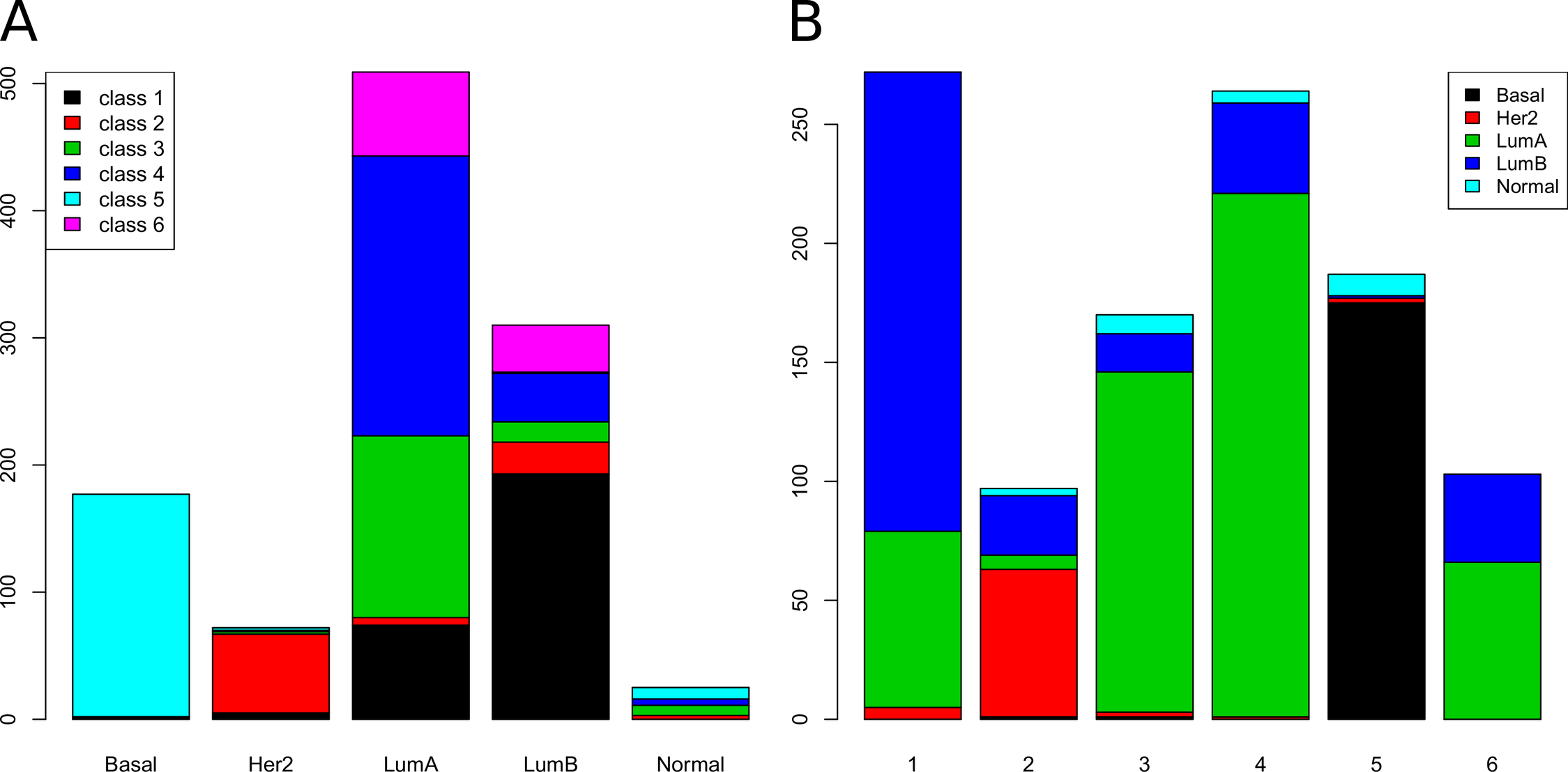

Supplement: S1 Fig — A. The proportion of each phenoClust cluster within the PAM50 sub-classes. The Basal class is composed almost solely of class 6, the HER2 subclass is mostly composed of class 2, the Luminal A subclass mostly consists of classes 3 and 4, and the Luminal B subclass is mostly composed of class 1. B. The reciprocal view of the proportion of PAM50 subclasses within each phenoClust cluster. Each of the phenoClust clusters consists mostly of a single PAM50 subclass. (TIF) [file pcbi.1006026.s002.tif]

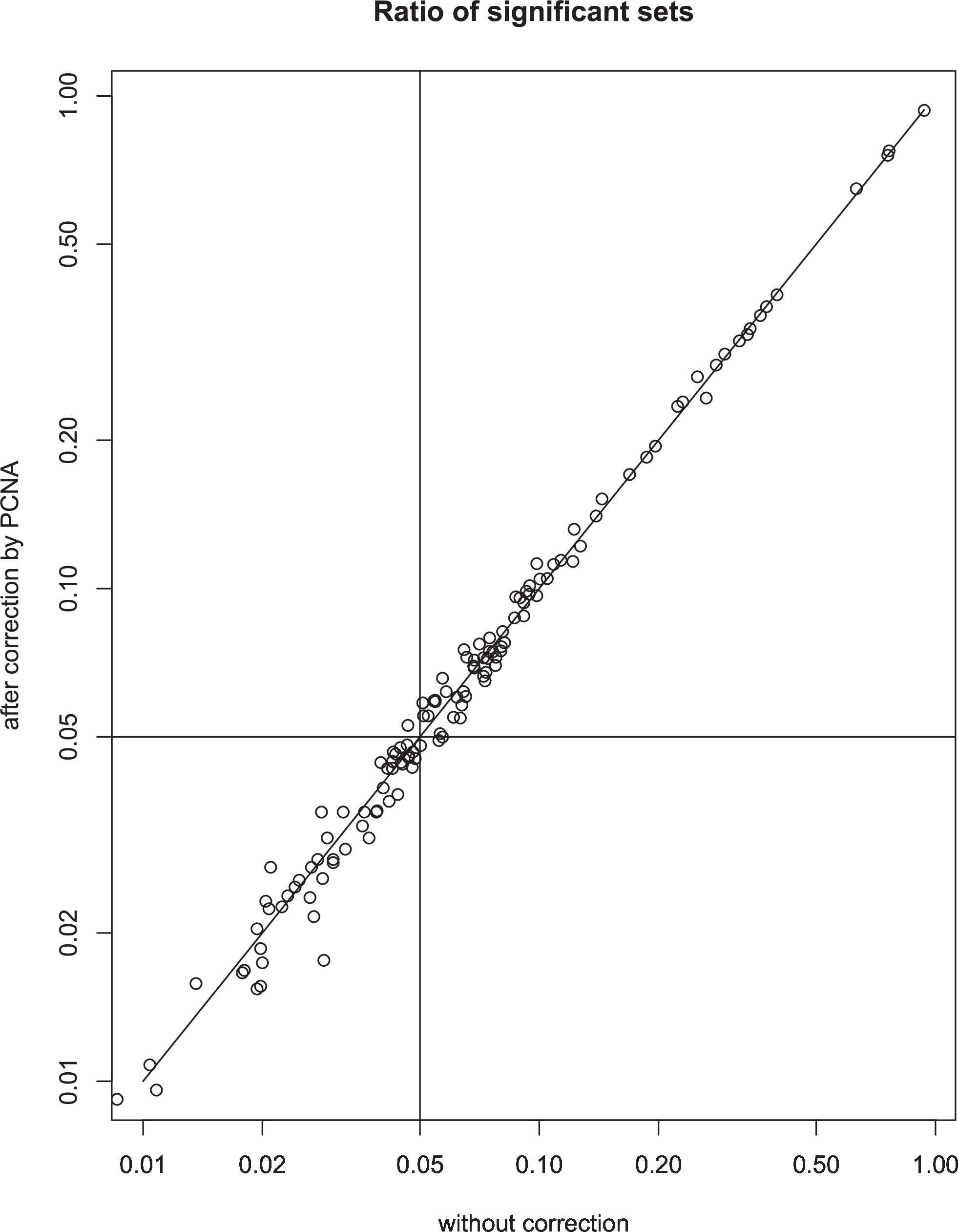

Supplement: S2 Fig — The x-axis represents the proportion of significant genes in each phenoClust cluster without adjusting for the PCNA signature, and the y-axis represents the proportion of significant genes in the same phenoClust clusters after adjusting for the PCNA signature. (TIF) [file pcbi.1006026.s003.tif]

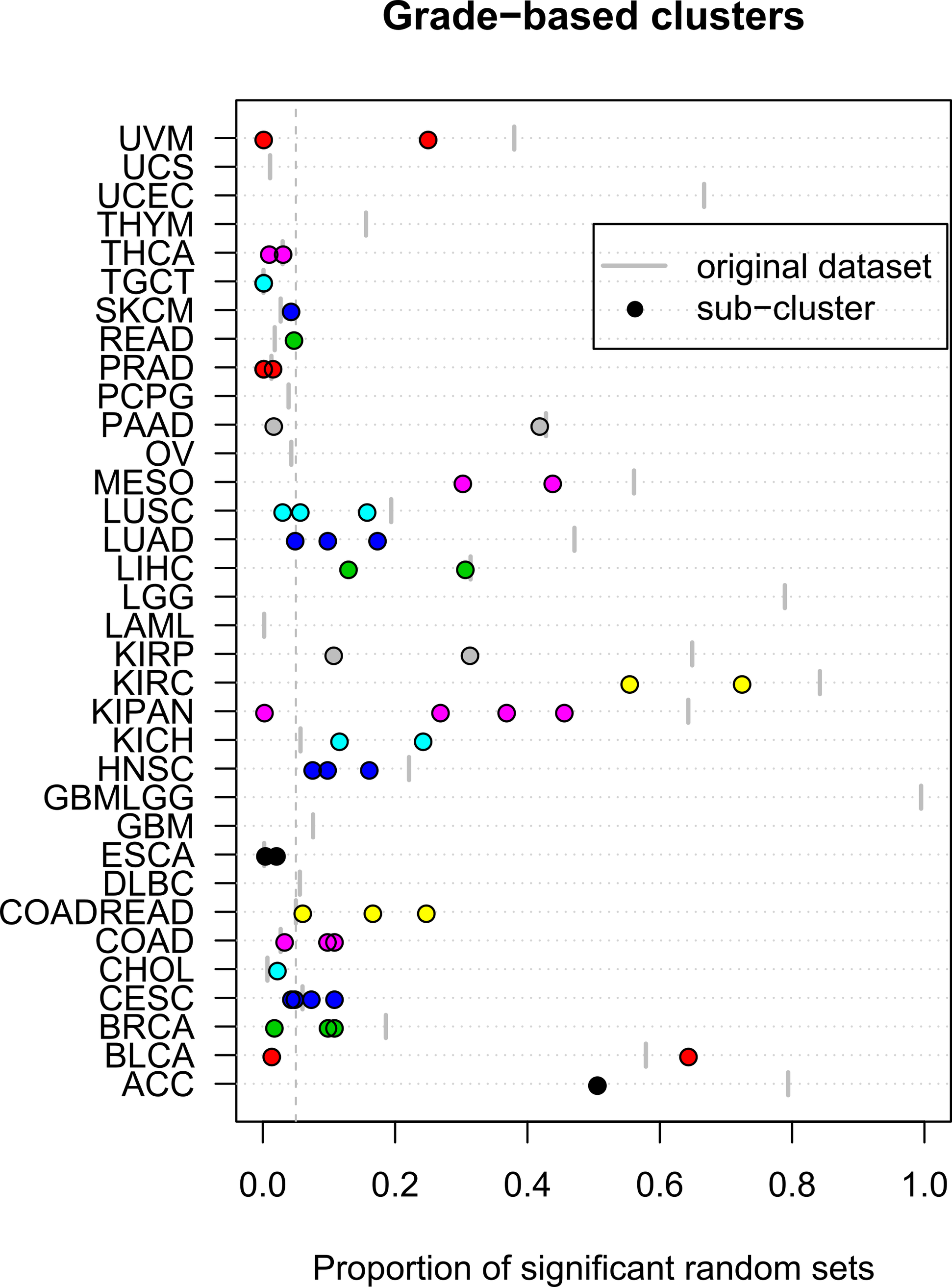

Supplement: S3 Fig — Each horizontal line represents a single TCGA dataset, where the location of each dot along the x axis represents the proportion of significant sets in a single cluster consisting of samples labeled with the same grade, and the location of the short vertical gray line indicates the proportion of significant sets in the complete dataset. Both proportions were calculated using random sets of size N = 64. Classes with less than 10 samples were not analyzed. The analysis was only performed for cancer types for which grade information is available in the clinical information provided by TCGA. (TIF) [file pcbi.1006026.s004.tif]
